# The molecular bases of floral scent evolution under artificial selection: insights from a transcriptome analysis in *Brassica rapa*

**DOI:** 10.1038/srep36966

**Published:** 2016-11-14

**Authors:** Jing Cai, Pengjuan Zu, Florian P. Schiestl

**Affiliations:** 1 Department of Systematic and Evolutionary Botany, University of Zürich, Zollikerstrasse 107, CH-8008 Zürich, Switzerland

## Abstract

In an artificial selection experiment using fast-cycling *Brassica rapa* plants it was recently shown that floral VOCs respond rapidly to selection for increased amounts. Here we carried out transcriptome analysis in these plants to explore the molecular bases of the augmentation in the artificially selected scent compound, phenylacetaldehyde (PAA), as well as other compounds that increased through pleiotropy. In the transcriptome data, we found up-regulation of genes likely underlying PAA synthesis, but also several genes of the shikimate pathway and the related phenylalanine metabolism. As phenylalanine is the precursor of many aromatic volatiles that showed increased emission, this result could explain some of the pleiotropic evolutionary responses. In addition, we found that ribosomal protein genes were up-regulated in “high” (high PAA amount) selection line plants, a mechanism that might further augment the effect of elevated gene expression at the proteomic level. Our study shows that selection on an individual trait can impose changes in the expression of several different genes, which could explain pleiotropic responses in the biosynthetic network of floral volatiles.

Natural selection is a fundamental concept underlying Darwinism and modern evolutionary theory. The molecular mechanisms of evolutionary change driven by natural selection have been investigated by many evolutionary biologists since the beginning of the molecular biology era. However, it is difficult to pinpoint the mechanism of adaptive evolution when studying wild populations unless selection and adaptive evolution is ongoing or happened only recently. Artificial selection experiments provide a solution to this problem as the selection process can be controlled. Humans have been applying artificial selection in the breeding of domesticated crops and animals since the onset of agricultural civilization, which has attracted many evolutionary biologists’ attention, including Darwin’s, as evidenced by his book “*The Variation of Animals and Plants under Domestication*”^1^. However, most domestication has happened quite a long time ago in human history (archaeological evidence of plant domestication has been dated to as early as 11,050 BC^2^) and an often complex breeding history makes it difficult to investigate the molecular mechanism underlying domestication. Artificial selection experiments circumvent this problem by focusing on the very early onset of evolutionary change. Previously, short term artificial selection experiments have been carried out in animals including fruit flies^3^,^4^,^5^, nematodes^6^ and fish^7^, but less often in plants^8^,^9^.

Flower scent is an important chemical trait in flowering plants, helping them to attract pollinators to enable sexual reproduction. Because floral scent is a signal to pollinators, it can impact plant ecological speciation through impacting both reproductive success and reproductive isolation^10^,^11^. However, the molecular bases of scent evolution remain largely unknown partially because the heavily studied plant models such as *Arabidopsis thaliana* and rice produce quite weak scent and do not rely on pollinators for sexual reproduction. Most of the few studies on the molecular bases of scent evolution were restricted to candidate genes in non-model plants^10^,^12^,^13^,^14^,^15^,^16^,^17^,^18^. *Brassica rapa* is a very good model for studying floral scent as it features strong scent emission and most populations are outcrossing and rely on insect pollinators for sexual reproduction. Fast cycling *Brassica rapa* (Wisconsin fast plants) is a strain with short life cycle and thus an ideal model for artificial selection studies (http://fastplants.org). Such an experiment has recently been carried out to assess the heritability of flower scent with four different scent compounds as selection targets^19^. In their study, three generations of artificial selection lead to significant scent divergence between “high” and “low” selection line plants. As the reference genome and many functional genomics tools are available for *Brassica rapa*, this system provides us with the possibility of a system-level exploration of the molecular mechanisms of scent evolution, instead of targeting individual genes. We focused our study on the high and low lines with the target compound phenylacetaldehyde, as it is one of the key pollinator-attractive volatile compounds in the flower scent of *Brassica rapa*^20^. We carried out transcriptome analysis on flowers of *Brassica rapa* fast plant lines selected for high and low PAA emission to address the question of the molecular bases of scent evolution in artificially selected plants at the transcriptome level.

## Material and Methods

### Study plants

Seeds of fast cycling *Brassica rapa* plants used in this study were collected from plants that had been selected for three generations with phenylacetaldehyde as the target compound according to procedures described in ref. 19. 50 seeds from ten individuals (5 seeds per each mother plant) of third generation plants for both “high” and “low” selection lines were sown out in standardized soil (Humuswerke Gebr. Patzer GmbH & Co.KG, http://www.einheitserde.de) in a phytotron under 24 hours fluorescent light at 22 °C, 60% relative humidity and were watered twice a day. One week after sown out, the seedlings were transferred into individual pots (7 cm*7 cm*8 cm) and kept under the same growth condition until the experiments were finished.

### Floral scent collection and analyses

Headspace collection of floral volatile organic compounds (VOCs) was carried out from 30 high line and 30 low line plants with a push-pull system when there around ten open flowers on each plant individual. The entire inflorescence was enclosed into cylindrical vessel made of glass previously silanized with Sigmacote (Sigma Aldrich, http://www.sigmaaldrich.com) to minimize VOC absorption to the glass. Two Teflon plates were used to close the open end with a small opening in the middle to hold the stem. The glass vessel had two holes, one through which charcoal-filtered air was pumped into the glass vessel (“push”) and one through which then scent-containing air was pulled out with a vacuum pump. The scent was collected at the “pull” port with glass tubes filled with absorbent (35 mg Tenax TA 60/80, Supelco, Sigma Aldrich, http://www.sigmaaldrich.com). The flow rate of push and pull were both set at 100 ml∙min^−1^. Floral scent was collected for three hours; one air control sample with the same settings but without a plant inside the glass vessel was collected for each batch. After scent sampling, the glass tubes with Tenax were removed from the system and immediately wrapped up with teflon tape. The samples were analyzed immediately or stored at −20 °C until analysis.

Analyses of floral scent was done using a gas chromatograph with a mass selective detector (GC-MSD; Agilent 6890 N, Agilent Technologies, http://www.agilent.com)) fitted with a thermal desorption system (Gerstel TDS/TDU, Gerstel, http://www.gerstel.com). Each glass tube was loaded and injected into the GC using a Gerstel MultiPurpose Sampler MPS. For thermal desorption, the temperature was programmed to start at 30 °C (hold for one minute) and increase to 240 °C (and held for one minute) at 60 °C min^−1^. The eluting volatiles from the TDS were collected and enriched at −150 °C at a cool injection system (CIS 4, Gerstel, http://www.gerstel.com). For injection, the CIS was heated to 150 °C at 16 °C s^−1^, then increased further from 150 °C to 250 °C at 12 °C s^−1^. The GC was equipped with an HP-5 capillary column (Agilent, 15 m length, 0.25mm diameter, 0.25 μm film thickness) and helium was used as the carrier gas with a constant flow of 2 ml min^−1^. The temperature of the GC oven was set to 50 °C (held for one minute) at first and then increased to 250 °C at 10 °C min^−1^. An Agilent 5975 Series MSD mass spectrometer was used to identify and quantify compounds. Chromatograms were analyzed with the ChemStation Enhanced Data Analysis program (Version E.01.00). The mass spectra obtained from the samples were matched with those of a reference collection (the National Institute of Standards and Technology (NIST) mass spectral library) for initial identification; then, retention times and mass spectra of all compounds included in the quantitative analyses were compared to those of synthetic reference standards. Peak areas of target ions were subsequently converted to compound quantity in to nanogram by applying calibration curves established for each compound. Quantitation of 16 floral scent compounds was done in the four individuals with the highest emission of phenylacetaldehyde (PAA) among all “high” line plants, and the four individuals with the lowest emission of PAA among all “low” line plants. As the program cannot always identify the peak correctly, manual integration was done when necessary.

### RNA extraction and library construction

After scent collection, the four individuals with the highest PAA emission, and the four individuals with the lowest PAA emission were used for RNAseq analyses. The flowers were collected and flash frozen in liquid nitrogen and stored at −80 °C until extraction. Frozen flowers were first homogenized with beads-beater and then used for RNA and DNA extraction. Total RNA was extracted using Trizol (Invitrogen) following the manufacturer’s instruction. The integrity of the RNA was then assessed using RNA nano chips on 2100 Bioanalyzer (Agilent Technologies, Inc). Then the following library construction and sequencing of the total RNA was done at Functional Genomic Center Zurich. The libraries were constructed with PolyA enrichment protocol using TruSeq Stranded mRNA library prep kit (Illumina) and then eight libraries were mixed to be sequenced in one lane on Hiseq2000 at single end for 100 bp. Totally, we got 212,759,422 reads from the eight libraries (on average 26,594,928 reads per sample). The raw reads were submitted to the NCBI under BioProject accession: PRJNA347876.

### Transcriptome data analyses

The Tophat-Cufflink protocol^21^ was used to quantify gene expression level and find genes with different expression level between high and low lines. First, quality control was done with the raw data using FastQC (http://www.bioinformatics.babraham.ac.uk/projects/fastqc/) with default settings. Then, filtered reads were mapped to the reference genome (GCF_000309985.1_Brapa_1.0 from NCBI ftp.ncbi.nih.gov/genomes/refseq/plant/Brassica_rapa/representative/GCF_000309985.1_Brapa_1.0/) with Bowtie^22^ implemented in TopHat. Finally we use Cuffdiff in the Cufflink package to find genes with different expression levels between high and low lines.

To find out whether there is a difference in the general pattern of expression profile of high line and low line, we carried out gene ontology (GO) enrichment test in genes with significantly different expression level between high lines and low lines. In addition, we also carried out gene set enrichment test for pathway analysis implemented in the R package of GAGE (Generally Applicable Gene-set Enrichment)^23^.

## Results and Discussion

### Volatiles with increased emission and transcriptome analysis

**Figure 1:**
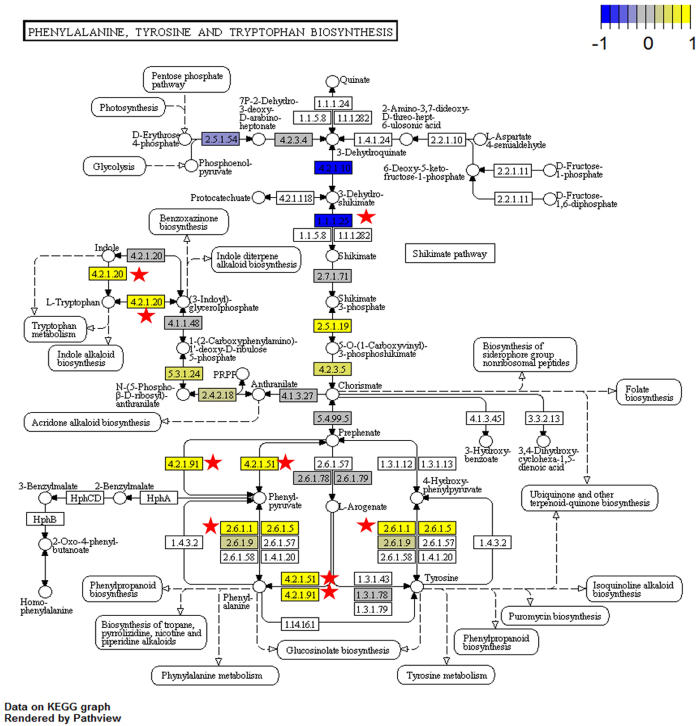
Log2 fold changes of gene expression level mapped onto the KEGG pathway module “phenylalanine, tyrosine and tryptophan biosynthesis” by R package “Pathview”. Phenylalanine is the precursor of phenylacetaldehyde in *Petunia*^30^. Most genes in the shikimate pathway and phenylalanine related pathway showed increased expression in high line plants. Red stars were used to label the reactions where significant expression changes were found in RNAseq data (q < 0.05).

Our data show that besides phenylacetaldehyde, several other VOCs were emitted in significantly higher amounts in the “high line” than in the “low line” plants (Table 1). Those increased VOCs include α-farnesene, 2-aminobenzaldehyde, indole, benzyl nitrile, methyl salicylate and methyl anthranilate. In addition, 2-phenylethanol also showed considerable emission in high line, but no emission in low line plants; nevertheless, there was no significant difference between high and low line plants for this compound, likely because of low sample size and variation in the high line plants. Many of these volatiles are known to attract pollinators^11^,^24^,^25^ and herbivores^26^,^27^,^28^; α-farnesene is also known to be repellent to ants^29^. Evolutionary change in non-selected VOCs can be explained by pleiotropy or close linkage between individual “scent genes” (linkage disequilibrium). These mechanisms were previously suggested to contribute to the evolution of scent bouquets^19^. The transcriptome data of the high and low selection line plants render us the possibility to unravel the molecular mechanisms of these phenomena. PAA synthesis was previously found to be catalyzed by PAA synthase (PAAS) with phenylalanine as substrate in *Petunia*^30^. After BLAST search, we identified five homologs of PAAS in the *Brassica rapa* genome annotated as tyrosine decarboxylase. Three of the genes showed significant up-regulation in the high lines while the other two showed no significant difference between high and low lines (Table 2). Thus, these three genes are probably the functional genes encoding the *PAAS* in *Brassica rapa*. In addition to the actual PAAS gene, several genes in the shikimate pathway which synthesizes phenylalanine as the substrate of PAA synthesis, also showed increased expression in high line plants. Specifically, genes in four of the six reactions from shikimate to phenylalanine showed increased expression (Fig. 1). On the contrary, there are only three reactions upstream of shikimate that showed decreased expression in two corresponding genes: 3-deoxy-D-arabino-heptulosonate 7-phosphate (*DAHP*) synthase and bifunctional 3-dehydroquinate dehydratase (*DHD*)–shikimate dehydrogenase (*SDH*). *DAHP* expression was shown to be induced upon wounding in Solanaceae^31^ and methyl jasmonate treatment in *Arabidopsis*^32^. However, we know little about the reason for the down-regulation of those genes; feedback of phenylalanine and other intermediate product accumulation in the plants may play a role in this response.

Benzyl nitrile is another compound for which phenylalanine is the precursor and was found to be co-upregulated with PAA. It has been shown in *Arabidopsis* that cytochrome *P450 CYP79A2* catalyzes the conversion of L-phenylalanine to phenylacetaldoxime^33^. Phenylacetaldoxime can then be conversed to benzyl nitrile by phenylacetaldoxime dehydratase. However, the later enzyme was up to now only found in a *Bacillus* strain^34^. Although benzyl nitrile can also originate from the hydrolyzation of glucosinolates, this reaction only occurs when myrosinase is activated by mechanical damage or herbivore attack which was not the case in our study. In the *Brassica rapa* genome, we identified two homologs of the gene *CYP79A2* catalyzing the first step from phenylalanine to phenylacetaldoxime (Table 2). We found that one of these genes showed significantly increased expression in the high lines while the other one showing quite low expression level in both high and low lines (Table 2). Thus, the first gene is probably the functional gene in the tissue assayed which catalyzes the initial step of the benzyl nitrile synthesis from phenylalanine.

The compound with the most similar pattern of co-upregulation with PAA in the high line plants is 2-phenylethanol. In the low line plants, 2-phenylethanol was not detected at all, similar to the selection target PAA, while considerable amount was found in high line plants. A 2-phenylacetaldehyde reductase was identified in tomato to catalyze the conversion of PAA to 2-phenylethanol (=phenylethyl alcohol)^35^. When searching the *Brassica rapa* genome using the protein sequence of 2-phenylacetaldehyde reductase gene as the query, we identified genes annotated as cinnamoyl-CoA reductase 1 and 2 gene family most similar to our query gene (see Supplementary Information). We checked the expression changes of all 13 members of the cinnamoyl-CoA reductase 1, and 7 members of the cinnamoyl-CoA reductase 2 gene family and only one gene (gene ID: 103846465) showed significant up-regulation in high line plants (Table 2). The gene 103846465 is a member of cinnamoyl-CoA reductase 2 gene family and could possibly encode a functional PAA synthase. However, a previous study in tomato also suggested that the synthesis of the substrate PAA is the limiting step in the synthesis of phenylethyl alcohol, because over-expression of 2-phenylacetaldehyde reductase in tomato does not necessarily lead to increased emission of phenylethyl alcohol^35^. The increased emission of phenylethyl alcohol in our high line plants is therefore also likely caused by increased substrate availability rather than increased expression of the gene catalyzing the final step in its synthesis.

Many other VOCs related to the shikimate pathway were also found to be emitted at higher levels in the high line plants, including indole and methyl anthranilate (Table 1). Indole is synthesized from chorismate which is an intermediate product of phenylalanine synthesis in the Shikimate pathway (http://www.genome.jp/kegg-bin/show_pathway?map00400). Genes catalyzing four of the eight reactions from shikimate to indole showed increased expression in our data (Fig. 1). Methyl anthranilate, which is probably synthesized via methylation of anthranilate, also showed higher emission rates in high lines. Anthranilate is again an intermediate product in the pathway from shikimate to indole.

The evolutionary change in the compounds discussed above are pleiotropic effects of selection on PAA. Interestingly, selection on PAA led to up-regulation of genes both directly involved in PAA synthesis as well as those upstream in the shikimate pathway, likely enhancing the availability of the substrate of PAA synthase, phenylalanine. Substrate availability is a well-known mechanism for regulation of scent emission^36^,^37^ and in our study probably one mechanism causing pleiotropic responses in floral scent compounds. One more possible mechanism contributing to pleiotropy is the upregulation of one (or more) transcription factor in high PAA lines, regulating transcription level of multiple target genes. However, due to current lack of experimental data on target promotors of transcription factors in *Brassica rapa*, we are not able to test this hypothesis with our dataset. To find out whether linkage disequilibrium plays a role in the evolution of the scent bouquet in high PAA line plants, we manually checked the genomic locations of all the 82 genes in the PAA pathway (see Supplementary Table S1). However, we couldn’t find any pair of neighboring genes (within 10 kbp distance) showing co-regulation in the PAA network except tandem duplicates in the same gene family. This shows that the genes controlling diverse, co-upregulated genes involved in the synthesis of aromatic compounds are not located spatially close to each other. Linkage is usually associated with spatial proximity and was suggested to evolve in traits comprising pollination syndromes in *Petunia*^38^. However, we cannot exclude the possibility that distant genes in the genome get close to each other due to spatial organization at three dimensional level.

### Increased transcription of ribosomal protein genes in high lines

**Figure 2:**
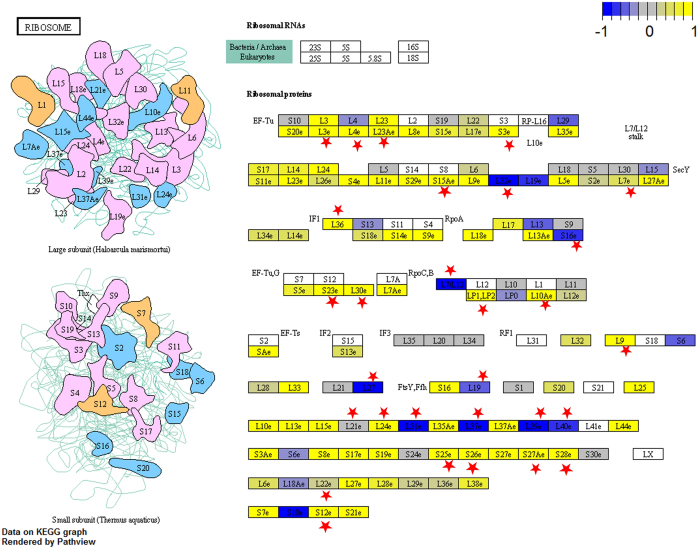
Log2 fold changes of gene expression level mapped onto the KEGG pathway module “ribosome” by R package “Pathview”. Most genes coding ribosomal proteins showed increased expression in high line plants. Red stars were used to label the reactions where significant expression changes were found in RNAseq data (q < 0.05).

In addition to scent genes and related pathways, our GO enrichment analysis in the genes with significantly different expression levels between high and low lines found that “structural constituent of ribosome” (GO:0003735, *P* = 1.4e-16), “translation” (GO:0006412, *P* = 1.3e-09) and “ribosome” (GO:0005840, *P* = 1.6e-14) were the top significantly over-represented GOs in the test of molecular function (MF), biological process (BP) and cellular component (CC), respectively. Those three over-represented GOs in their respective categories (MF, BP and CC) are actually referring to exactly the same molecular identity. All showed that the ribosomal protein genes as a group were significantly over-represented among all genes with increased expression level in high lines compared to low lines. In addition, our gene set enrichment test for pathway found that the KEGG pathway “ko03010 Ribosome” was the only significantly enriched pathway in genes with different expression level (*P* = 2.7e-6, q = 3.5e-4, Fig. 2). This result is consistent with the GO enrichment test and confirmed that ribosomal protein genes are the most prominent group of genes up-regulated in high PAA line compared to low line plants.

Ribosomes as the protein translation machinery are conserved in all organisms and play an important role in translating the mRNAs into functional proteins. The hypothetic consequence of up-regulation of ribosomal protein genes is higher throughput of protein translation with the same level of mRNA input. As ribosomal proteins have been taken as reference genes in many studies, little attention has been paid to the biological meaning of their up-regulation. Only until Thorezz *et al*. published their work on falsifying the validity of ribosomal proteins as reference genes, tissues with active proliferating or secreting cells were found to have elevated expression level in almost all ribosomal protein genes^39^. Higher efficiency in protein translation as a result of more ribosomal proteins available may be necessary for those tissues to maintain their function. Similarly, higher ribosomal protein gene expression may add another layer of gene regulation and might contribute to the (short term) scent evolution at the protein level, in addition of the up-regulation of many genes involved in scent metabolism at transcriptome level. In addition to translation, many redundant ribosomal protein genes in plants were found to exert extra-ribosomal functions during development, such as stress response and other physiological process^40^,^41^,^42^. Those up-regulated ribosomal proteins in high lines might also play a role other than translation in the evolution of high PAA line plants.

To avoid bias due to alignment and the statistical method, we re-analyze the transcriptome data with a different protocol (Subread-edgeR) and also mapped the differentially expressed genes in the KEGG pathways. The results (see Supplementary Figs S1 and S2 and Table S2) from the alternative protocol showed high consistency with the first protocol, indicating that our conclusions are independent of the computational method used.

In conclusion, we show that multiple molecular factors may contribute to floral scent evolution, at least in the short term, namely over three generations with artificial selection. Correlated expression pattern with VOC emissions were found in the transcriptome data including increased expression of genes related to the biosynthesis of scent compounds and their biosynthetic precursors, as well as increased activity of the ribosomal translation machinery were the possible main mechanisms of increased scent emission in our study. In future investigations, the role of some SNPs or indels selected in the high lines and methylation changes during scent evolution deserve more attention. Also, it would be interesting to contrast short term to long term evolutionary change to see whether quick adaptive responses to environmental fluctuation in plants are mediated by different molecular mechanisms than long term evolutionary changes, for example during speciation.

## Additional Information

**How to cite this article**: Cai, J. *et al*. The molecular bases of floral scent evolution under artificial selection: insights from a transcriptome analysis in *Brassica rapa*. *Sci. Rep*. **6**, 36966; doi: 10.1038/srep36966 (2016).

**Publisher’s note**: Springer Nature remains neutral with regard to jurisdictional claims in published maps and institutional affiliations.

## Supplementary Material

Supplementary Information

## Figures and Tables

**Table 1:** Results of the floral scent analysis of low- and high line *Brassica rapa* plants.

Volatile Compounds	L1	L2	L3	L4	H1	H2	H3	H4	*P*-value	Change Fold
*α-Farnesene*	22.89	19.37	12.22	29.72	61.52	73.70	69.93	57.05	**1**.**52E-04**	3.11E + 00
*Phenylacetaldehyde*	0.00	0.00	0.76	0.00	1299.68	1066.12	1427.14	1145.34	**6**.**01E-04**	6.50E + 03
*2-Aminobenzaldehyde*	11.00	3.14	31.50	83.61	1657.05	684.75	1554.58	1351.11	**9**.**65E-03**	4.06E + 01
*Indole*	12.31	12.09	37.08	52.26	774.52	334.06	759.55	535.03	**1**.**15E-02**	2.11E + 01
*Benzyl nitrile*	5.46	5.17	7.85	5.45	113.99	110.12	197.36	78.48	**1**.**83E-02**	2.09E + 01
*Methyl salicylate*	1.13	2.27	0.77	0.96	5.28	4.03	4.05	1.99	**2**.**46E-02**	2.99E + 00
*Methyl anthranilate*	16.76	11.62	28.65	52.54	325.24	73.40	295.26	198.75	**3**.**92E-02**	8.15E + 00
2-Phenylethanol*	0.00	0.00	0.00	0.00	99.75	147.19	53.64	23.81	5.75E-02	NA
Tridecane	0.48	0.36	0.30	0.00	0.00	0.00	0.00	0.00	6.83E-02	0.00E + 00
Limonene	21.78	13.16	17.54	12.98	29.94	23.33	17.66	52.61	1.53E-01	1.89E + 00
p-Anisaldehyde	0.25	0.00	0.06	0.04	0.00	0.00	0.00	0.00	2.14E-01	0.00E + 00
1-Butene-3-isothiocyanate	6.88	6.87	7.19	0.00	4.17	3.46	2.76	0.00	2.44E-01	4.96E-01
Z-3-Hexenyl acetate	10.30	4.86	3.41	5.77	13.81	6.49	7.16	7.37	2.92E-01	1.43E + 00
Methyl benzoate	6.14	14.72	10.29	5.73	21.94	4.10	17.69	13.74	2.94E-01	1.56E+00
Acetophenone	5.37	3.13	5.12	7.86	13.19	4.77	7.10	5.92	3.17E-01	1.44E + 00
Decanal	5.39	6.12	9.33	0.00	6.04	0.00	5.89	3.64	6.04E-01	7.47E-01
Tetradecane	3.11	3.28	1.82	2.63	3.78	2.25	1.83	1.72	6.07E-01	8.84E-01
Benzaldehyde	118.94	67.97	182.50	361.75	194.48	106.24	182.91	180.87	8.18E-01	9.09E-01

L1, L2, L3 and L4 denote the four individuals with lowest PAA emission in the low selection line while H1, H2, H3 and H4 denote the four individuals with the highest PAA emission in the high selection line. All values are in nanogram per inflorescence and 18 l sampled air. Unpaired t-tests were carried out between high line and low line plants for each compound. Significant P-values are given in bold (P < 0.05) and compounds with significant differences are given in italics. (*The P value of 2-phenylethanol is not significant due to large variation in high lines and relatively low sample size. However, the difference is striking considering the constant zero emission in low line plants).

**Table 2:** Candidate scent genes and their expression profile in high and low line plants.

gene id	genomic location	High line expression	Low line expression	log2(fold_ change)	t statistics	*P*_value	*q*_value	Significant
tyrosine_decarboxylase (*PAAS*)								
103828182	NC_024801.1:217015–219880	1.16E + 01	9.69E + 00	−2.57E-01	−5.23E-01	3.33E-01	7.14E-01	no
103828183	NC_024801.1:223788–228639	1.19E + 00	9.78E-01	−2.85E-01	−2.89E-01	5.98E-01	8.79E-01	no
103838003	NC_024803.1:5661975–5665055	4.61E + 01	2.09E + 00	−4.47E + 00	−1.05E + 01	5.00E-05	2.30E-03	yes
103842606	NC_024803.1:32424436–32427586	6.79E + 01	2.52E-01	−8.07E + 00	−8.76E+00	5.00E-05	2.30E-03	yes
103854232	NC_024795.1:4244022–4248394	1.18E + 02	4.45E + 01	−1.41E + 00	−3.19E + 00	5.00E-05	2.30E-03	yes
phenylalanine_N-monooxygenase (*P450 CYP79A2*)								
103846879	NC_024804.1:15250750–15252899	1.70E-02	0.00E + 00	NA	0.00E+00	1.00E + 00	1.00E + 00	no
103850577	NC_024796.1:2153108–2155365	6.91E + 01	2.36E + 00	−4.87E + 00	−8.12E + 00	5.00E-05	2.30E-03	yes
cinnamoyl-CoA reductase_1								
103865283	NC_024798.1:14938835–14940534	3.67E + 01	3.68E + 01	3.91E-03	8.54E-03	9.86E-01	9.97E-01	no
103868569	NC_024799.1:11229002–11230594	4.20E-01	1.38E + 00	1.71E + 00	1.37E + 00	2.25E-02	1.81E-01	no
103871720	NC_024800.1:3242697–3244612	1.43E + 00	3.14E + 00	1.13E + 00	1.26E + 00	3.67E-02	2.40E-01	no
103871721	NC_024800.1:3245173–3247064	2.20E + 01	2.44E + 01	1.50E-01	3.30E-01	5.42E-01	8.53E-01	no
103872395	NC_024800.1:6188947–6192033	1.76E + 01	2.16E + 01	2.94E-01	6.37E-01	2.50E-01	6.34E-01	no
103832863	NC_024802.1:2105426–2118132	1.02E + 00	2.97E + 00	1.54E + 00	9.03E-01	1.12E-01	4.33E-01	no
103840198	NC_024803.1:20479284–20480968	4.97E-01	1.01E + 00	1.02E + 00	7.62E-01	1.74E-01	5.37E-01	no
103842871	NC_024803.1:33483629–33486514	2.00E + 01	2.58E + 01	3.64E-01	8.02E-01	1.44E-01	4.91E-01	no
103843352	NC_024803.1:35358842–35361339	1.06E + 01	2.06E + 01	9.64E-01	1.86E + 00	8.00E-04	2.04E-02	yes
103845276	NC_024804.1:8411299–8412899	5.55E + 01	6.03E + 01	1.19E-01	2.72E-01	6.05E-01	8.82E-01	no
103845753	NC_024804.1:10740540–10742420	4.80E + 01	4.99E + 01	5.37E-02	1.20E-01	8.21E-01	9.58E-01	no
103852537	NC_024796.1:11146161–11147957	3.59E-01	3.71E-01	4.66E-02	0.00E + 00	1.00E + 00	1.00E + 00	no
103854003	NC_024796.1:20583329–20585096	1.52E + 01	1.24E + 01	−2.93E-01	−5.47E-01	3.29E-01	7.10E-01	no
cinnamoyl-CoA reductase_2								
103861967	NC_024797.1:27844289–27847356	22.7478	21.0823	−0.109689	−0.249812	0.64525	0.89601	no
103864503	NC_024798.1:10682605–10684927	23.8815	25.8379	0.113595	0.263578	0.63055	0.890483	no
103832110	NC_024801.1:21084209–21086311	0.705213	1.49623	1.0852	0.970637	0.0824	0.373585	no
103832452	NC_024801.1:22383799–22389243	0.216609	0.128428	−0.754128	0	1	1	no
103846465	NC_024804.1:13672550–13675499	38.9807	14.2729	−1.44948	−3.17679	5.00E-05	0.002297	yes
103850944	NC_024796.1:3554969–3560372	1.36395	1.17333	−0.217176	−0.209659	0.7009	0.919132	no
103853214	NC_024796.1:15647909–15653369	1.08E + 00	1.95E + 00	0.849172	0.284177	0.70735	0.921026	no

Expression level are measured in FPKM (Fragments Per Kilobase of transcript per Million mapped reads). *P* value of the significance of expression change and *P* value corrected with Benjamini-Hochberg correction (*q* value) are calculated with default models in Cuffdiff.
