# Supplementary material for: The molecular bases of floral scent evolution under artificial selection: insights from a transcriptome analysis in Brassica rapa
Source: Sci Rep. 2016 Nov 14;6:36966. doi: 10.1038/srep36966 (PMC5107913; doi:10.1038/srep36966)
Supplement: Supplementary Information [file srep36966-s1.pdf]

The molecular bases of floral scent evolution under artificial selection: insights from a transcriptome analysis in *Brassica rapa*

Jing Cai†, Pengjuan Zu, Florian, P. Schiestl*

Department of Systematic and Evolutionary Botany, University of Zürich

Address: Zollikerstrasse 107, CH-8008 Zürich, Switzerland

†Current address: State Key Laboratory of Quality Research in Chinese Medicine, Institute of Chinese Medical Sciences, University of Macau, Avenida da Universidade, Taipa, Macau SAR, China

*Corresponding author: florian.schiestl@systbot.uzh.ch.

SUPPLEMENTARY INFORMATION

Figure S1. Log2 fold changes of gene expression level calculated by alternative protocol mapped onto the KEGG pathway module "phenylalanine, tyrosine and tryptophan biosynthesis" by R package "Pathview". Most genes in the shikimate pathway and phenylalanine related pathway showed increased expression in high line plants.

Figure S2. Log2 fold changes of gene expression level calculated by alternative protocol mapped onto the KEGG pathway module "ribosome" by R package "Pathview". Most genes coding ribosomal proteins showed increased expression in high line plants.

Table S1. Genes in the KEGG Phenyalanine metabolic pathway and their expression profile in high and low line plants.

Table S2. Gene counts data and differential expression test result of all discussed genes from alternative protocol.

Results of Blast searches of interested genes in Table 2.

a. **Blast search of tyrosine_decarboxylase (PAAS)**
b. **Blast search of phenylalanine_N-monooxygenase (P450 CYP79A2)**
c. **Blast search of cinnamoyl-CoA reductase**

Figure S1

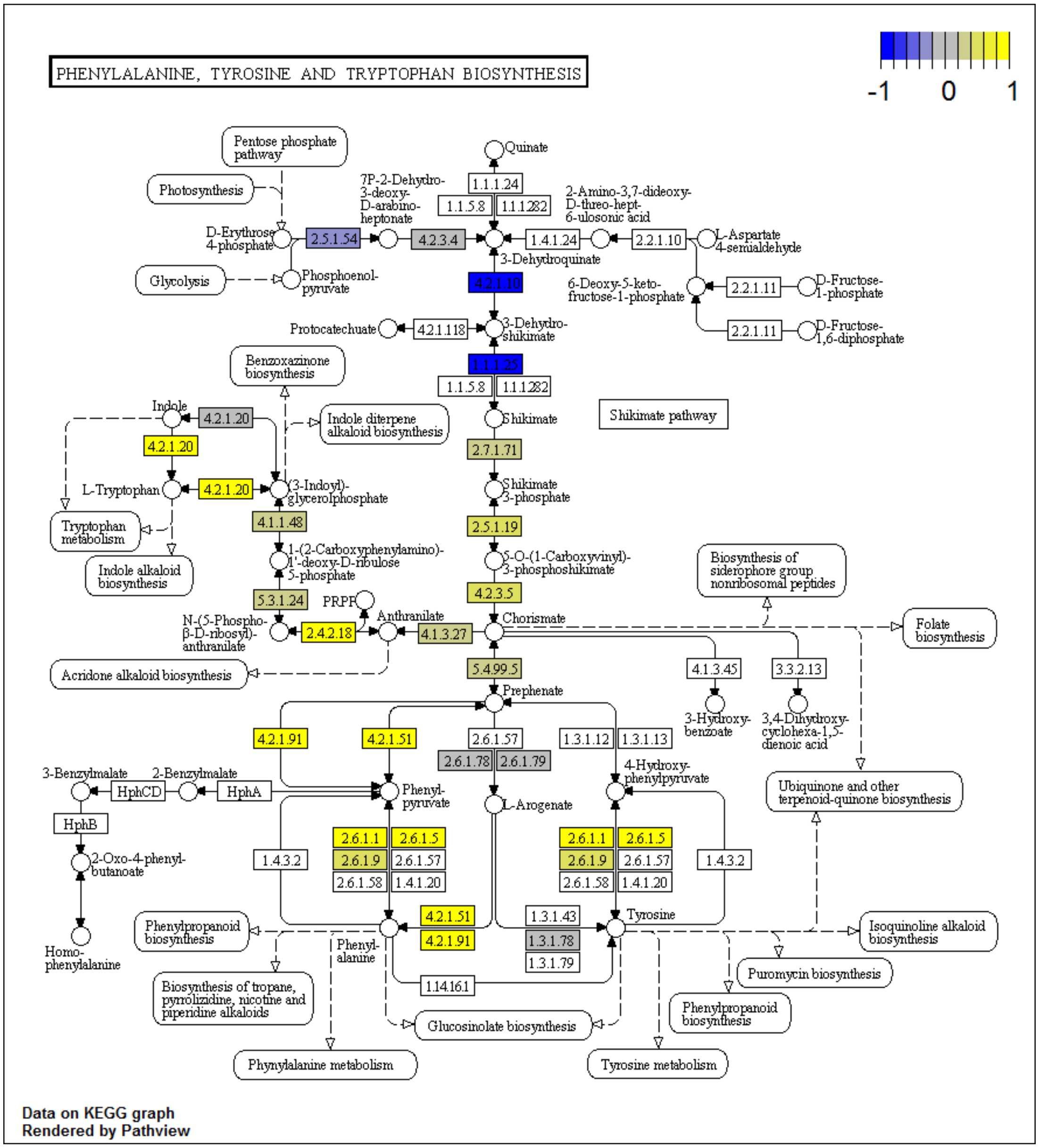

PHENYLALANINE, TYROSINE AND TRYPTOPHAN BIOSYNTHESIS
-1
0
1
Pentose phosphate pathway
Photosynthesis
Glycolysis
D-Erythrose 4-phosphate
2.5.1.54
7P-2-Dehydro-3-deoxy-D-arabino-heptonate
4.2.3.4
Phosphoenol-pyruvate
Quinate
1.1.1.24
1.1.5.8
1.1.1.282
2-Amino-3,7-dideoxy-D-threo-hept-6-ulosonic acid
1.4.1.24
2.2.1.10
L-Aspartate 4-semialdehyde
3-Dehydroquinate
4.2.1.10
6-Deoxy-5-keto-fructose-1-phosphate
2.2.1.11
D-Fructose-1-phosphate
2.2.1.11
D-Fructose-1,6-diphosphate
Protocatechuate
4.2.1.118
3-Dehydro-shikimate
Benzoxazinone biosynthesis
1.1.1.25
1.1.5.8
1.1.1.282
Shikimate
Shikimate pathway
Indole
4.2.1.20
Indole diterpene alkaloid biosynthesis
4.2.1.20
2.7.1.71
L-Tryptophan
4.2.1.20
(3-Indoyl)-glycerolphosphate
Shikimate 3-phosphate
Tryptophan metabolism
4.1.1.48
2.5.1.19
Indole alkaloid biosynthesis
1-(2-Carboxyphenylamino)-1'-deoxy-D-ribulose 5-phosphate
5-O-(1-Carboxyvinyl)-3-phosphoshikimate
Biosynthesis of siderophore group nonribosomal peptides
5.3.1.24
PRPP
4.2.3.5
N-(5-Phospho-β-D-ribosyl)-anthranilate
2.4.2.18
Anthranilate
4.1.3.27
Chorismate
Folate biosynthesis
Acridone alkaloid biosynthesis
5.4.99.5
4.1.3.45
3.3.2.13
Prephenate
3-Hydroxy-benzoate
3,4-Dihydroxy-cyclohexa-1,5-dienoic acid
4.2.1.91
4.2.1.51
2.6.1.57
1.3.1.12
1.3.1.13
2.6.1.78
2.6.1.79
3-Benzylmalate
2-Benzylmalate
HphCD
HphA
Phenyl-pyruvate
4-Hydroxy-phenylpyruvate
Ubiquinone and other terpenoid-quinone biosynthesis
L-Arogenate
HphB
2-Oxo-4-phenyl-butanoate
1.4.3.2
2.6.1.1
2.6.1.5
2.6.1.9
2.6.1.57
2.6.1.58
1.4.1.20
2.6.1.1
2.6.1.5
2.6.1.9
2.6.1.57
2.6.1.58
1.4.1.20
1.4.3.2
4.2.1.51
1.3.1.43
Tyrosine
Phenylpropanoid biosynthesis
4.2.1.91
1.3.1.78
Isoquinoline alkaloid biosynthesis
Homo-phenylalanine
Phenyl-alanine
1.3.1.79
Puromycin biosynthesis
Biosynthesis of tropane, pyrrolizidine, nicotine and piperidine alkaloids
1.14.16.1
Phenylpropanoid biosynthesis
Glucosinolate biosynthesis
Phynylalanine metabolism
Tyrosine metabolism
Data on KEGG graph
Rendered by Pathview

Figure S2

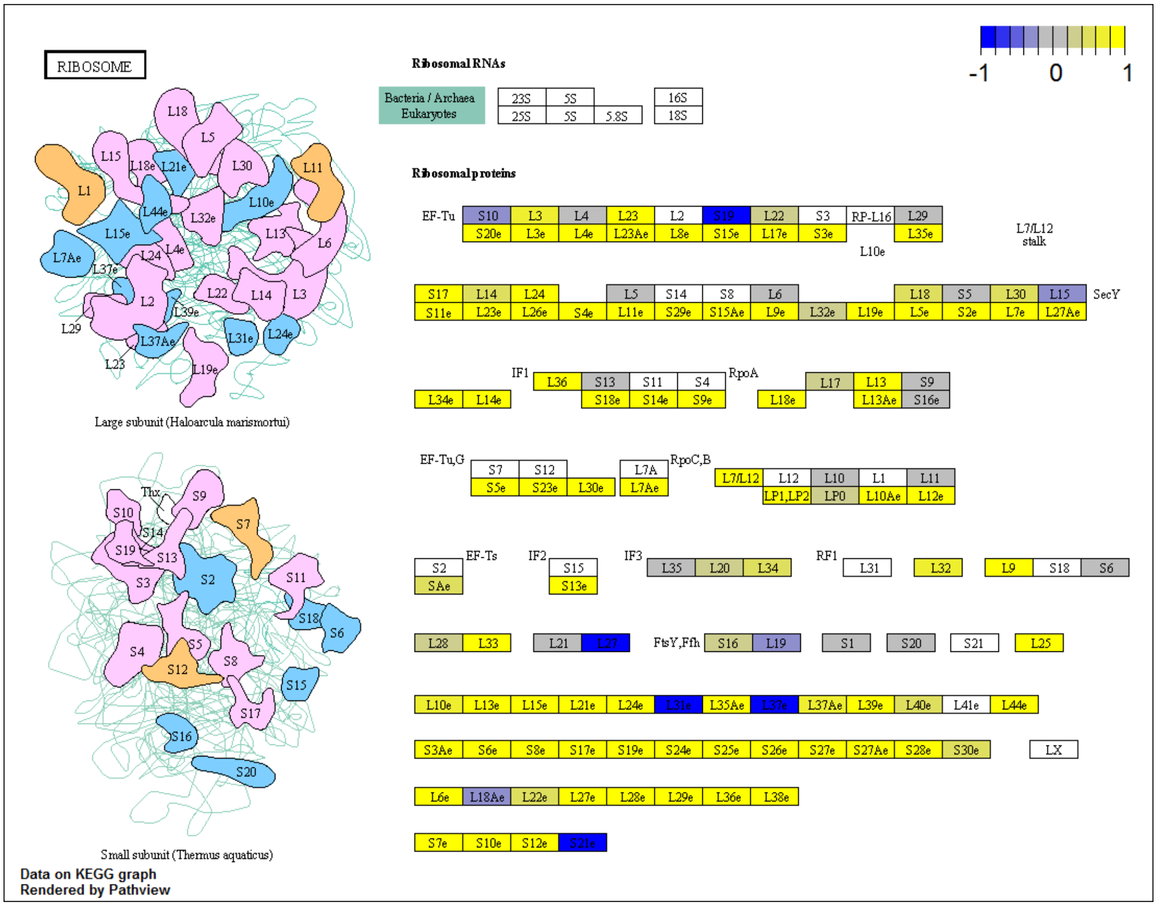
RIBOSOME
Ribosomal RNAs
Bacteria / Archaea
Eukaryotes
Ribosomal proteins
Large subunit (Haloarcula marismortui)
Small subunit (Thermus aquaticus)
-1 0 1
Data on KEGG graph
Rendered by Pathview

Table S1. Genes in the KEGG Phenylalanine metabolic pathway and their expression profile in high and low line plants. Expression level are measured in FPKM (Fragments Per Kilobase of transcript per Million mapped reads). P value of the significance of expression change and P value corrected with Benjamini-Hochberg correction (q value) are calculated with default models in Cuffdiff.

Functional annotation	KEGG annotation	Gene ID	Genomic location	High line expression	Low line expression	log2(fold change)	t statistics	P-value	q value
anthranilate phosphoribosyltransferase;	K00766 anthranilate phosphoribosyltransferase [EC:2.4.2.18] [KO:K00766] [EC:2.4.2.18]	103845884	NC_024804.1:11351364-11354059	2.33E+00	3.16E+00	4.40E-01	5.72E-01	2.89E-01	6.73E-01
anthranilate phosphoribosyltransferase;	K00766 anthranilate phosphoribosyltransferase [EC:2.4.2.18] [KO:K00766] [EC:2.4.2.18]	103851145	NC_024796.1:4387839-4390731	6.79E+00	3.88E+00	-8.09E-01	-2.15E+00	1.29E-02	1.31E-01
3-phosphoshikimate 1-carboxyvinyltransferase;	K00800 3-phosphoshikimate 1-carboxyvinyltransferase [EC:2.5.1.19] [KO:K00800] [EC:2.5.1.19]	103858170	NC_024797.1:10859740-10862284	5.62E+01	4.43E+01	-3.43E-01	-7.36E-01	1.70E-01	5.31E-01
3-phosphoshikimate 1-carboxyvinyltransferase;	K00800 3-phosphoshikimate 1-carboxyvinyltransferase [EC:2.5.1.19] [KO:K00800] [EC:2.5.1.19]	103866199	NC_024798.1:18531777-18534804	1.90E+01	1.34E+01	-5.02E-01	-1.10E+00	3.97E-02	2.52E-01
aspartate aminotransferase;	K00811 aspartate aminotransferase, chloroplastic [EC:2.6.1.1] [KO:K00811] [EC:2.6.1.1]	103851376	NC_024795.1:2777902-2780694	5.00E+01	2.50E+01	-9.99E-01	-2.42E+00	1.00E-04	4.08E-03
aspartate aminotransferase;	K00811 aspartate aminotransferase, chloroplastic [EC:2.6.1.1] [KO:K00811] [EC:2.6.1.1]	103862102	NC_024797.1:28544455-28547630	3.69E+01	4.85E+01	3.96E-01	9.43E-01	9.37E-02	3.99E-01

cystine lyase CORI3-like;	K00815 tyrosine aminotransferase [EC:2.6.1.5] [KO:K00815] [EC:2.6.1.5]	103833946	NC_024802.1:9197923-9204981	1.50E+01	2.08E+01	4.72E-01	9.74E-01	6.54E-02	3.32E-01
cystine lyase CORI3-like;	K00815 tyrosine aminotransferase [EC:2.6.1.5] [KO:K00815] [EC:2.6.1.5]	103834290	NC_024802.1:11988092-11992692	3.09E+00	4.89E+00	6.60E-01	8.96E-01	1.09E-01	4.28E-01
probable aminotransferase TAT4;	K00815 tyrosine aminotransferase [EC:2.6.1.5] [KO:K00815] [EC:2.6.1.5]	103841973	NC_024803.1:30050225-30052394	1.09E+01	1.73E+01	6.68E-01	1.27E+00	2.13E-02	1.75E-01
probable aminotransferase TAT3;	K00815 tyrosine aminotransferase [EC:2.6.1.5] [KO:K00815] [EC:2.6.1.5]	103842224	NC_024803.1:31145799-31149623	0.00E+00	5.70E-02	NA	0.00E+00	1.00E+00	1.00E+00
tyrosine aminotransferase-like;	K00815 tyrosine aminotransferase [EC:2.6.1.5] [KO:K00815] [EC:2.6.1.5]	103842591	NC_024803.1:32377789-32379657	1.77E+01	1.32E+01	-4.27E-01	-8.44E-01	1.22E-01	4.52E-01
probable aminotransferase TAT2;	K00815 tyrosine aminotransferase [EC:2.6.1.5] [KO:K00815] [EC:2.6.1.5]	103852000	NC_024796.1:8008508-8011128	2.13E+01	2.84E+01	4.14E-01	1.54E+00	8.03E-02	3.69E-01
probable aminotransferase TAT1;	K00815 tyrosine aminotransferase [EC:2.6.1.5] [KO:K00815] [EC:2.6.1.5]	103854450	NC_024795.1:4355026-4357143	8.22E-01	4.07E-02	-4.34E+00	-2.23E+00	1.48E-01	4.97E-01
probable aminotransferase TAT3;	K00815 tyrosine aminotransferase [EC:2.6.1.5] [KO:K00815] [EC:2.6.1.5]	103858362	NC_024797.1:11707883-11710300	3.69E-02	3.69E-02	-1.08E-03	0.00E+00	1.00E+00	1.00E+00
probable aminotransferase TAT4;	K00815 tyrosine aminotransferase [EC:2.6.1.5] [KO:K00815] [EC:2.6.1.5]	103861263	NC_024795.1:7341194-7343313	1.32E-01	2.78E-02	-2.24E+00	0.00E+00	1.00E+00	1.00E+00
cystine lyase CORI3-like;	K00815 tyrosine aminotransferase [EC:2.6.1.5] [KO:K00815] [EC:2.6.1.5]	103861272	NC_024795.1:7346252-7358519	3.22E+01	5.00E+01	6.36E-01	1.38E+00	1.24E-02	1.28E-01
probable aminotransferase TAT4;	K00815 tyrosine aminotransferase [EC:2.6.1.5] [KO:K00815] [EC:2.6.1.5]	103861408	NC_024797.1:25151783-25153948	4.72E-02	5.22E-02	1.45E-01	0.00E+00	1.00E+00	1.00E+00

cystine lyase CORI3-like;	K00815 tyrosine aminotransferase [EC:2.6.1.5] [KO:K00815] [EC:2.6.1.5]	103861409	NC_024797.1:25165731-25168280	9.23E-02	1.18E-02	-2.97E+00	0.00E+00	1.00E+00	1.00E+00
cystine lyase CORI3-like;	K00815 tyrosine aminotransferase [EC:2.6.1.5] [KO:K00815] [EC:2.6.1.5]	103861413	NC_024797.1:25190709-25195452	1.14E-02	0.00E+00	NA	0.00E+00	1.00E+00	1.00E+00
S-alkyl-thiohydroximate lyase SUR1-like;	K00815 tyrosine aminotransferase [EC:2.6.1.5] [KO:K00815] [EC:2.6.1.5]	103861830	NC_024797.1:26922948-26926716	6.05E+01	8.05E+01	4.13E-01	8.60E-01	1.17E-01	4.43E-01
S-alkyl-thiohydroximate lyase SUR1-like;	K00815 tyrosine aminotransferase [EC:2.6.1.5] [KO:K00815] [EC:2.6.1.5]	103862132	NC_024797.1:26902572-26908100	0.00E+00	0.00E+00	0.00E+00	0.00E+00	1.00E+00	1.00E+00
tyrosine aminotransferase;	K00815 tyrosine aminotransferase [EC:2.6.1.5] [KO:K00815] [EC:2.6.1.5]	103868301	NC_024799.1:9396505-9398613	2.00E+01	1.92E+01	-5.88E-02	-1.45E-01	8.13E-01	9.56E-01
histidinol-phosphate aminotransferase 1;	K00817 histidinol-phosphate aminotransferase [EC:2.6.1.9] [KO:K00817] [EC:2.6.1.9]	103832256	NC_024801.1:19423733-19426136	4.94E+00	4.63E+00	-9.13E-02	-1.99E-01	7.78E-01	9.46E-01
histidinol-phosphate aminotransferase 1;	K00817 histidinol-phosphate aminotransferase [EC:2.6.1.9] [KO:K00817] [EC:2.6.1.9]	103852737	NC_024796.1:12407799-12409953	1.31E+01	1.14E+01	-2.00E-01	-4.35E-01	4.30E-01	7.89E-01
shikimate kinase 2;	K00891 shikimate kinase [EC:2.7.1.71] [KO:K00891] [EC:2.7.1.71]	103850170	NC_024795.1:213493-215634	1.54E+01	2.10E+01	4.50E-01	9.86E-01	7.81E-02	3.65E-01
shikimate kinase 1;	K00891 shikimate kinase [EC:2.7.1.71] [KO:K00891] [EC:2.7.1.71]	103864307	NC_024798.1:9476707-9479255	7.78E+01	5.96E+01	-3.85E-01	-8.46E-01	1.27E-01	4.59E-01
indole-3-glycerol phosphate synthase;	K01609 indole-3-glycerol phosphate synthase [EC:4.1.1.48] [KO:K01609] [EC:4.1.1.48]	103860298	NC_024797.1:19872761-19875284	1.80E+01	1.47E+01	-2.86E-01	-5.86E-01	2.68E-01	6.53E-01

indole-3-glycerol phosphate synthase;	K01609 indole-3-glycerol phosphate synthase [EC:4.1.1.48] [KO:K01609] [EC:4.1.1.48]	103874936	NC_024800.1:21169936-21172146	1.74E+01	1.93E+01	1.54E-01	3.24E-01	5.41E-01	8.52E-01
phospho-2-dehydro-3-deoxyheptonate aldolase 1;	K01626 3-deoxy-7-phosphoheptulonate synthase [EC:2.5.1.54] [KO:K01626] [EC:2.5.1.54]	103832310	NC_024801.1:21846937-21849556	2.76E+01	2.29E+01	-2.67E-01	-5.84E-01	2.73E-01	6.57E-01
phospho-2-dehydro-3-deoxyheptonate aldolase 1;	K01626 3-deoxy-7-phosphoheptulonate synthase [EC:2.5.1.54] [KO:K01626] [EC:2.5.1.54]	103835703	NC_024802.1:18167445-18173199	2.12E+00	1.56E+00	-4.39E-01	-5.33E-01	3.09E-01	6.92E-01
phospho-2-dehydro-3-deoxyheptonate aldolase 2;	K01626 3-deoxy-7-phosphoheptulonate synthase [EC:2.5.1.54] [KO:K01626] [EC:2.5.1.54]	103846297	NC_024795.1:2123385-2126462	1.37E+01	4.36E+01	1.67E+00	2.95E+00	5.00E-05	2.30E-03
phospho-2-dehydro-3-deoxyheptonate aldolase 1;	K01626 3-deoxy-7-phosphoheptulonate synthase [EC:2.5.1.54] [KO:K01626] [EC:2.5.1.54]	103854257	NC_024795.1:386082-388339	3.11E+01	4.81E+01	6.32E-01	1.41E+00	9.25E-03	1.08E-01
phospho-2-dehydro-3-deoxyheptonate aldolase 1;	K01626 3-deoxy-7-phosphoheptulonate synthase [EC:2.5.1.54] [KO:K01626] [EC:2.5.1.54]	103869052	NC_024799.1:15582668-15585492	2.14E+02	8.58E+01	-1.32E+00	-2.21E+00	2.50E-04	8.46E-03
anthranilate synthase alpha subunit 1;	K01657 anthranilate synthase component I [EC:4.1.3.27] [KO:K01657] [EC:4.1.3.27]	103846914	NC_024804.1:15360328-15363453	4.13E+00	2.99E+00	-4.67E-01	-7.38E-01	1.71E-01	5.33E-01
anthranilate synthase alpha subunit 1;	K01657 anthranilate synthase component I [EC:4.1.3.27] [KO:K01657] [EC:4.1.3.27]	103855638	NC_024797.1:979359-982276	3.31E+01	3.28E+01	-1.29E-02	-2.79E-02	9.59E-01	9.91E-01

anthranilate synthase alpha subunit 2;	K01657 anthranilate synthase component I [EC:4.1.3.27] [KO:K01657] [EC:4.1.3.27]	103858269	NC_024797.1:11272671-11275602	6.44E+00	9.77E+00	6.00E-01	1.21E+00	2.82E-02	2.08E-01
anthranilate synthase beta subunit 1;	K01658 anthranilate synthase component II [EC:4.1.3.27] [KO:K01658] [EC:4.1.3.27]	103835440	NC_024802.1:17405757-17407789	2.25E+01	1.91E+01	-2.41E-01	-4.98E-01	3.47E-01	7.25E-01
anthranilate synthase beta subunit 1;	K01658 anthranilate synthase component II [EC:4.1.3.27] [KO:K01658] [EC:4.1.3.27]	103839088	NC_024803.1:12884411-12886461	6.27E+00	6.17E+00	-2.43E-02	-3.90E-02	9.43E-01	9.89E-01
tryptophan synthase alpha chain;	K01695 tryptophan synthase alpha chain [EC:4.2.1.20] [KO:K01695] [EC:4.2.1.20]	103836728	NC_024803.1:117707-120888	1.02E+01	1.02E+01	1.18E-02	1.17E-02	9.84E-01	9.97E-01
tryptophan synthase alpha chain;	K01695 tryptophan synthase alpha chain [EC:4.2.1.20] [KO:K01695] [EC:4.2.1.20]	103841367	NC_024803.1:27248533-27250508	1.85E+01	2.03E+01	1.33E-01	3.08E-01	6.01E-01	8.80E-01
tryptophan synthase beta chain 1-like;	K01696 tryptophan synthase beta chain [EC:4.2.1.20] [KO:K01696] [EC:4.2.1.20]	103836224	NC_024802.1:20191496-20195122	1.33E+02	5.34E+01	-1.32E+00	-2.52E+00	5.00E-05	2.30E-03
tryptophan synthase beta chain 1-like;	K01696 tryptophan synthase beta chain [EC:4.2.1.20] [KO:K01696] [EC:4.2.1.20]	103836225	NC_024802.1:20199937-20202903	1.85E+00	2.96E+00	6.75E-01	9.23E-01	1.06E-01	4.22E-01
tryptophan synthase beta chain 1-like;	K01696 tryptophan synthase beta chain [EC:4.2.1.20] [KO:K01696] [EC:4.2.1.20]	103837846	NC_024803.1:4881343-4890369	1.25E+00	8.41E-01	-5.74E-01	-6.26E-01	2.42E-01	6.26E-01

tryptophan synthase beta chain 1;	K01696 tryptophan synthase beta chain [EC:4.2.1.20] [KO:K01696] [EC:4.2.1.20]	103844934	NC_024804.1:6537356-6539498	1.24E+01	1.10E+01	-1.73E-01	-8.56E-01	4.59E-01	8.07E-01
tryptophan synthase beta chain 2;	K01696 tryptophan synthase beta chain [EC:4.2.1.20] [KO:K01696] [EC:4.2.1.20]	103861672	NC_024797.1:26455371-26457643	8.79E+00	6.72E+00	-3.88E-01	-7.90E-01	1.66E-01	5.25E-01
3-dehydroquinate synthase;	K01735 3-dehydroquinate synthase [EC:4.2.3.4] [KO:K01735] [EC:4.2.3.4]	103837671	NC_024803.1:4110034-4112146	2.79E+01	2.62E+01	-9.09E-02	-2.10E-01	7.08E-01	9.22E-01
chorismate synthase;	K01736 chorismate synthase [EC:4.2.3.5] [KO:K01736] [EC:4.2.3.5]	103832977	NC_024802.1:2957982-2960850	2.09E+01	2.36E+01	1.74E-01	4.20E-01	4.62E-01	8.09E-01
chorismate synthase;	K01736 chorismate synthase [EC:4.2.3.5] [KO:K01736] [EC:4.2.3.5]	103868667	NC_024799.1:12159341-12165326	4.35E+01	2.79E+01	-6.42E-01	-1.37E+00	1.02E-02	1.14E-01
N-(5'-phosphoribosyl)anthranilate isomerase 1;	K01817 phosphoribosyl-anthranilate isomerase [EC:5.3.1.24] [KO:K01817] [EC:5.3.1.24]	103850592	NC_024796.1:2217843-2219492	1.16E+01	8.16E+00	-5.03E-01	-9.29E-01	9.24E-02	3.96E-01
chorismate mutase 3;	K01850 chorismate mutase [EC:5.4.99.5] [KO:K01850] [EC:5.4.99.5]	103830932	NC_024801.1:16233520-16235877	1.39E+01	7.76E+00	-8.42E-01	-1.53E+00	9.30E-03	1.08E-01
chorismate mutase 3;	K01850 chorismate mutase [EC:5.4.99.5] [KO:K01850] [EC:5.4.99.5]	103831335	NC_024801.1:18335261-18339326	2.69E+00	2.39E+00	-1.75E-01	-1.39E-01	8.10E-01	9.55E-01
chorismate mutase 1;	K01850 chorismate mutase [EC:5.4.99.5] [KO:K01850] [EC:5.4.99.5]	103837205	NC_024803.1:2147284-2149276	1.90E+00	2.07E+00	1.23E-01	1.40E-01	7.90E-01	9.49E-01
chorismate mutase 2;	K01850 chorismate mutase [EC:5.4.99.5] [KO:K01850] [EC:5.4.99.5]	103850294	NC_024796.1:1078813-1080530	1.81E+00	3.32E+00	8.78E-01	1.07E+00	6.86E-02	3.41E-01

chorismate mutase 1;	K01850 chorismate mutase [EC:5.4.99.5] [KO:K01850] [EC:5.4.99.5]	103854148	NC_024796.1:21480354-21481623	0.00E+00	0.00E+00	0.00E+00	0.00E+00	1.00E+00	1.00E+00
chorismate mutase 1;	K01850 chorismate mutase [EC:5.4.99.5] [KO:K01850] [EC:5.4.99.5]	103875095	NC_024800.1:21871603-21873467	2.45E+01	2.35E+01	-6.13E-02	-1.38E-01	7.99E-01	9.51E-01
arogenate dehydratase/prephenate dehydratase 1;	K05359 arogenate/prephenate dehydratase [EC:4.2.1.91 4.2.1.51] [KO:K05359] [EC:4.2.1.51 4.2.1.91]	103836212	NC_024802.1:20103656-20120640	1.91E+00	2.55E+00	4.12E-01	1.80E-01	8.47E-01	9.64E-01
arogenate dehydratase/prephenate dehydratase 6;	K05359 arogenate/prephenate dehydratase [EC:4.2.1.91 4.2.1.51] [KO:K05359] [EC:4.2.1.51 4.2.1.91]	103843406	NC_024803.1:35579090-35580575	9.56E-01	1.14E+00	2.50E-01	2.33E-01	6.73E-01	9.09E-01
arogenate dehydratase/prephenate dehydratase 6;	K05359 arogenate/prephenate dehydratase [EC:4.2.1.91 4.2.1.51] [KO:K05359] [EC:4.2.1.51 4.2.1.91]	103843407	NC_024803.1:35590470-35591964	3.25E-01	7.72E-02	-2.07E+00	0.00E+00	1.00E+00	1.00E+00
arogenate dehydratase 5;	K05359 arogenate/prephenate dehydratase [EC:4.2.1.91 4.2.1.51] [KO:K05359] [EC:4.2.1.51 4.2.1.91]	103845522	NC_024804.1:9666783-9669018	4.53E+01	2.69E+01	-7.52E-01	-1.95E+00	2.35E-03	4.28E-02
arogenate dehydratase 5;	K05359 arogenate/prephenate dehydratase [EC:4.2.1.91 4.2.1.51] [KO:K05359] [EC:4.2.1.51 4.2.1.91]	103851454	NC_024796.1:5541181-5542974	1.32E+02	3.32E+01	-1.99E+00	-4.10E+00	5.00E-05	2.30E-03

arogenate dehydratase/prephenate dehydratase 2;	K05359 arogenate/prephenate dehydratase [EC:4.2.1.91 4.2.1.51] [KO:K05359] [EC:4.2.1.51 4.2.1.91]	103859235	NC_024797.1:15564557-15567159	1.84E+00	1.25E+00	-5.58E-01	-6.01E-01	2.56E-01	6.40E-01
arogenate dehydratase 3;	K05359 arogenate/prephenate dehydratase [EC:4.2.1.91 4.2.1.51] [KO:K05359] [EC:4.2.1.51 4.2.1.91]	103864806	NC_024798.1:12198428-12200050	8.95E+00	1.18E+01	4.06E-01	7.62E-01	1.64E-01	5.22E-01
arogenate dehydratase/prephenate dehydratase 2;	K05359 arogenate/prephenate dehydratase [EC:4.2.1.91 4.2.1.51] [KO:K05359] [EC:4.2.1.51 4.2.1.91]	103870648	NC_024799.1:22771086-22773673	1.44E+01	1.32E+01	-1.26E-01	-2.61E-01	6.22E-01	8.87E-01
arogenate dehydratase/prephenate dehydratase 1;	K05359 arogenate/prephenate dehydratase [EC:4.2.1.91 4.2.1.51] [KO:K05359] [EC:4.2.1.51 4.2.1.91]	103871931	NC_024800.1:4109051-4115117	8.28E-01	1.26E+00	6.03E-01	4.91E-01	3.71E-01	7.42E-01
arogenate dehydratase 4;	K05359 arogenate/prephenate dehydratase [EC:4.2.1.91 4.2.1.51] [KO:K05359] [EC:4.2.1.51 4.2.1.91]	103873609	NC_024800.1:13263916-13266942	1.69E+01	2.31E+01	4.50E-01	1.05E+00	7.21E-02	3.50E-01
tryptophan synthase beta chain 1-like;	K06001 tryptophan synthase beta chain [EC:4.2.1.20] [KO:K06001] [EC:4.2.1.20]	103850119	NC_024796.1:156932-159489	2.80E+00	2.45E+00	-1.94E-01	-2.70E-01	6.13E-01	8.85E-01
tryptophan synthase beta chain 1-like;	K06001 tryptophan synthase beta chain [EC:4.2.1.20] [KO:K06001] [EC:4.2.1.20]	103863791	NC_024798.1:6535471-6537941	4.42E+00	6.03E+00	4.49E-01	7.66E-01	1.76E-01	5.40E-01

bifunctional 3-dehydroquinate dehydratase/shikimate dehydrogenase;	K13832 3-dehydroquinate dehydratase / shikimate dehydrogenase [EC:4.2.1.10 1.1.1.25] [KO:K13832] [EC:1.1.1.25 4.2.1.10]	103849983	NC_024795.1:28233064-28236971	1.18E+01	1.56E+01	4.07E-01	8.78E-01	9.79E-02	4.06E-01
bifunctional 3-dehydroquinate dehydratase/shikimate dehydrogenase;	K13832 3-dehydroquinate dehydratase / shikimate dehydrogenase [EC:4.2.1.10 1.1.1.25] [KO:K13832] [EC:1.1.1.25 4.2.1.10]	103854474	NC_024796.1:23271850-23276196	1.77E+00	4.91E+00	1.47E+00	2.12E+00	3.00E-04	9.73E-03
bifunctional 3-dehydroquinate dehydratase/shikimate dehydrogenase;	K13832 3-dehydroquinate dehydratase / shikimate dehydrogenase [EC:4.2.1.10 1.1.1.25] [KO:K13832] [EC:1.1.1.25 4.2.1.10]	103859154	NC_024797.1:15248266-15251563	4.13E+00	5.59E+00	4.36E-01	7.69E-01	1.56E-01	5.11E-01
aspartate aminotransferase;	K14454 aspartate aminotransferase, cytoplasmic [EC:2.6.1.1] [KO:K14454] [EC:2.6.1.1]	103838043	NC_024803.1:5893646-5897611	1.06E+01	9.58E+00	-1.42E-01	-2.80E-01	6.23E-01	8.88E-01
aspartate aminotransferase;	K14454 aspartate aminotransferase, cytoplasmic [EC:2.6.1.1] [KO:K14454] [EC:2.6.1.1]	103838363	NC_024803.1:7620230-7623525	0.00E+00	0.00E+00	0.00E+00	0.00E+00	1.00E+00	1.00E+00
aspartate aminotransferase;	K14454 aspartate aminotransferase, cytoplasmic [EC:2.6.1.1] [KO:K14454] [EC:2.6.1.1]	103845742	NC_024804.1:10694564-10698271	6.25E+01	5.52E+01	-1.81E-01	-4.14E-01	4.52E-01	8.03E-01

aspartate aminotransferase;	K14454 aspartate aminotransferase, cytoplasmic [EC:2.6.1.1] [KO:K14454] [EC:2.6.1.1]	103848200	NW_008720769.1:838106-843700	3.32E-02	0.00E+00	NA	0.00E+00	1.00E+00	1.00E+00
aspartate aminotransferase;	K14454 aspartate aminotransferase, cytoplasmic [EC:2.6.1.1] [KO:K14454] [EC:2.6.1.1]	103851283	NC_024796.1:4808262-4811559	2.03E+01	1.21E+01	-7.42E-01	-1.52E+00	6.05E-03	8.24E-02
aspartate aminotransferase 3;	K14454 aspartate aminotransferase, cytoplasmic [EC:2.6.1.1] [KO:K14454] [EC:2.6.1.1]	103855915	NC_024797.1:2009114-2016889	8.28E+01	9.20E+01	1.51E-01	3.11E-01	5.87E-01	8.74E-01
aspartate aminotransferase;	K14455 aspartate aminotransferase, mitochondrial [EC:2.6.1.1] [KO:K14455] [EC:2.6.1.1]	103865090	NC_024798.1:13851500-13854557	7.50E+01	6.73E+01	-1.56E-01	-3.46E-01	5.19E-01	8.39E-01
aspartate aminotransferase;	K14455 aspartate aminotransferase, mitochondrial [EC:2.6.1.1] [KO:K14455] [EC:2.6.1.1]	103867922	NC_024799.1:7210962-7213496	2.78E+01	3.96E+01	5.07E-01	1.21E+00	3.18E-02	2.22E-01
arogenate dehydrogenase 2;	K15227 arogenate dehydrogenase (NADP+), plant [EC:1.3.1.78] [KO:K15227] [EC:1.3.1.78]	103842888	NC_024803.1:33562413-33563757	1.28E+01	1.27E+01	-1.37E-02	-4.07E-02	9.57E-01	9.91E-01
arogenate dehydrogenase 1;	K15227 arogenate dehydrogenase (NADP+), plant [EC:1.3.1.78] [KO:K15227] [EC:1.3.1.78]	103858107	NC_024797.1:10619264-10621358	8.06E+00	8.82E+00	1.31E-01	2.76E-01	6.11E-01	8.84E-01

bifunctional aspartate aminotransferase and glutamate/aspartate-prephenate aminotransferase;	K15849 bifunctional aspartate aminotransferase and glutamate/aspartate-prephenate aminotransferase [EC:2.6.1.1 2.6.1.78 2.6.1.79] [KO:K15849] [EC:2.6.1.79 2.6.1.78 2.6.1.1]	103837904	NC_024803.1:5173642-5176198	9.66E+00	1.02E+01	7.91E-02	1.53E-01	7.75E-01	9.45E-01
bifunctional aspartate aminotransferase and glutamate/aspartate-prephenate aminotransferase-like;	K15849 bifunctional aspartate aminotransferase and glutamate/aspartate-prephenate aminotransferase [EC:2.6.1.1 2.6.1.78 2.6.1.79] [KO:K15849] [EC:2.6.1.79 2.6.1.78 2.6.1.1]	103864337	NC_024798.1:9635097-9637360	1.02E+01	9.33E+00	-1.25E-01	-2.37E-01	6.56E-01	9.00E-01

Table S2. Gene counts data and differential expression test result of all discussed genes from alternative protocol. H1,H 2, H3, H4 stand for four high emission individuals, while L1, L2, L3, L4 stand for low emission individuals. logFC is the log fold change of gene expression in low over high emission plants. FDR is the false discovery rate corrected with multiple hypothesis test correction. When the counts number is too small(no more than 1 read per million in at least two samples), the statistic test results are left empty because we take those extremely low expression genes meaningless.

Gene ID	H1	H2	H3	H4	L1	L2	L3	L4	logFC	P value	FDR
tyrosine_decarboxylase (PAAS)											
103854232	3794	2446	2960	2466	1226	840	2818	1098	-1.14E+00	2.58E-05	2.58E-03
103828182	450	516	385	450	163	263	898	364	-4.57E-01	7.60E-02	4.33E-01
103828183	71	6	30	36	26	20	32	23	-4.48E-01	4.88E-01	8.39E-01
103838003	957	1729	2853	3358	44	74	258	128	-4.50E+00	3.12E-22	6.27E-19
103842606	3713	4730	3186	2805	7	28	24	8	-7.89E+00	1.64E-41	1.23E-37
phenylalanine_N-monooxygenase (P450CYP79A2)											
103850577	2982	2560	3036	2215	26	63	233	145	-4.92E+00	9.31E-40	5.60E-36
103846879	10	0	2	1	0	0	0	0			
cinnamoyl-CoA reductase 1											
103852537	1	7	18	0	0	2	0	23			
103854003	321	287	359	228	199	170	554	259	-2.57E-01	3.08E-01	7.34E-01
103865283	978	964	1166	901	783	640	1462	958	-2.07E-01	3.63E-01	7.71E-01
103868569	7	9	8	7	11	21	4	6			
103871720	9	12	68	46	10	33	313	170	1.39E+00	1.06E-01	5.00E-01
103871721	507	325	514	462	268	462	942	661	1.55E-01	3.80E-01	7.79E-01
103872395	477	390	435	505	267	384	707	515	-1.24E-01	4.40E-01	8.12E-01
103832863	17	25	29	19	20	60	34	101	1.13E+00	4.93E-02	3.54E-01

	103840198	11	1	22	8	9	30	13	18			
	103842871	423	482	595	648	449	563	1177	696	2.31E-01	2.63E-01	7.00E-01
	103843352	221	152	135	245	189	318	423	408	7.16E-01	5.16E-03	1.03E-01
	103845276	1372	937	1020	1419	830	873	2319	1442	8.11E-04	9.96E-01	1.00E+00
	103845753	1398	1168	1110	1232	581	1209	2648	1318	-3.53E-02	8.57E-01	9.75E-01
cinnamoyl-CoA reductase 2												
	103850944	69	23	29	13	7	35	52	38	-2.17E-01	7.14E-01	9.31E-01
	103853214	17	34	33	24	51	19	55	40	5.22E-01	3.29E-01	7.49E-01
	103861967	695	394	580	605	329	339	747	543	-3.66E-01	3.07E-02	2.78E-01
	103864503	816	588	553	785	385	520	1347	1088	4.34E-02	8.20E-01	9.66E-01
	103832110	8	20	17	36	28	30	67	25	6.97E-01	1.88E-01	6.23E-01
	103832452	3	2	3	3	0	0	0	3			
	103846465	1813	747	1690	820	170	707	1358	609	-1.10E+00	6.04E-03	1.13E-01
genes in KEGG phenylalanine metabolic pathway												
	103850170	398	296	326	302	245	421	649	383	2.13E-01	3.09E-01	7.35E-01
	103854257	1268	1453	1582	1256	1410	1719	5539	1900	6.15E-01	1.44E-02	1.89E-01
	103846297	243	268	271	1484	1026	1012	3186	1894	1.44E+00	1.29E-02	1.79E-01
	103851376	1360	1334	1450	1324	592	740	1568	333	-9.35E-01	9.11E-03	1.47E-01
	103854450	91	21	38	0	0	1	47	0	-2.43E+00	1.71E-01	6.01E-01
	103861263	0	2	2	5	0	0	1	2			
	103861272	443	704	663	992	1144	379	4724	1246	9.95E-01	4.38E-02	3.33E-01
	103849983	669	430	550	476	360	404	1121	755	8.55E-02	6.00E-01	8.88E-01
	103850119	56	68	110	155	64	55	138	91	-3.32E-01	3.94E-01	7.87E-01
	103850294	90	36	54	85	40	59	133	102	1.46E-01	6.39E-01	9.04E-01
	103850592	442	242	217	278	133	178	500	288	-3.41E-01	1.26E-01	5.35E-01
	103851145	292	188	190	284	126	90	146	240	-7.22E-01	3.62E-02	3.01E-01
	103851283	929	367	691	454	248	187	512	429	-9.64E-01	6.75E-04	2.75E-02
	103851454	7144	1843	4086	1851	320	712	3059	746	-2.01E+00	6.09E-05	4.93E-03
	103852000	908	721	611	1159	618	796	1833	1419	2.50E-01	2.13E-01	6.52E-01
	103852737	469	368	403	564	282	272	671	511	-2.37E-01	1.99E-01	6.36E-01

103854148	4	0	4	4	0	3	10	3			
103854474	104	62	63	94	100	63	323	440	1.25E+00	6.11E-03	1.14E-01
103855638	2007	991	1999	849	1022	1107	2101	1480	-1.51E-01	6.10E-01	8.92E-01
103855915	2874	3302	3014	3082	2283	2158	7385	4290	1.07E-01	6.22E-01	8.98E-01
103858107	368	267	412	337	205	462	667	380	1.45E-01	5.57E-01	8.68E-01
103858170	3273	1968	3594	2269	1318	2029	6725	2452	-1.47E-01	5.71E-01	8.74E-01
103858269	378	283	303	365	289	336	926	576	4.45E-01	3.75E-03	8.50E-02
103858362	1	0	1	1	1	0	1	1			
103859154	311	205	256	192	240	162	364	357	1.17E-01	6.74E-01	9.16E-01
103859235	63	48	58	116	21	48	69	37	-8.56E-01	3.09E-02	2.79E-01
103860298	778	402	287	687	351	238	782	406	-4.27E-01	1.73E-01	6.04E-01
103861408	2	1	0	2	0	1	3	1			
103861409	1	1	2	3	0	2	13	1			
103861413	1	0	0	0	0	0	0	0			
103861672	788	335	454	515	433	504	1032	590	1.66E-01	4.29E-01	8.06E-01
103862132	2	0	0	2	0	0	2	1			
103861830	1667	2421	1904	2816	2174	1597	4703	3023	1.80E-01	5.17E-01	8.50E-01
103862102	1246	1066	1001	1163	822	835	2614	1631	1.44E-01	3.90E-01	7.86E-01
103863791	127	108	108	179	125	146	318	127	2.88E-01	3.22E-01	7.45E-01
103864307	3311	1529	2826	1844	1063	1600	3374	1617	-4.94E-01	3.85E-02	3.11E-01
103864337	428	276	290	286	196	280	699	342	1.28E-02	9.53E-01	9.98E-01
103864806	279	182	199	249	253	211	592	300	4.05E-01	6.05E-02	3.87E-01
103865090	2119	1641	2029	1499	1323	973	3340	2379	-8.71E-02	7.00E-01	9.27E-01
103866199	767	622	688	600	352	348	1050	871	-2.76E-01	1.62E-01	5.88E-01
103867922	643	626	757	721	692	731	2016	1398	5.78E-01	7.49E-04	2.98E-02
103868301	462	396	359	809	313	316	831	670	-1.29E-01	6.31E-01	9.02E-01
103868667	1824	1047	1439	1503	565	762	2203	989	-6.17E-01	2.80E-04	1.48E-02
103869052	8859	6450	9849	5906	1984	2935	10886	3562	-1.04E+00	3.12E-04	1.59E-02
103870648	501	328	452	468	280	325	1018	477	6.51E-03	9.79E-01	1.00E+00
103871931	22	17	15	14	16	13	88	34	7.68E-01	9.26E-02	4.72E-01
103873609	361	389	465	403	330	456	565	527	1.17E-01	6.40E-01	9.05E-01
103874936	529	399	585	365	381	276	861	774	8.40E-02	7.49E-01	9.42E-01
103875095	722	527	729	746	431	580	1338	770	-1.53E-02	9.18E-01	9.91E-01

103830932	396	114	242	193	94	106	258	191	-6.97E-01	2.48E-02	2.48E-01
103831335	31	59	44	45	35	21	100	54	-7.48E-02	8.52E-01	9.74E-01
103832256	264	181	179	194	132	101	336	238	-2.31E-01	3.36E-01	7.54E-01
103832310	1203	489	1319	833	623	545	2241	803	-1.34E-01	6.59E-01	9.11E-01
103832977	1122	726	887	1044	569	877	1852	1136	2.93E-02	8.37E-01	9.71E-01
103833946	320	225	213	511	314	310	691	468	3.51E-01	2.00E-01	6.38E-01
103834290	663	470	390	902	716	517	1416	935	4.28E-01	1.19E-01	5.24E-01
103835440	581	384	561	546	264	310	916	588	-2.43E-01	1.35E-01	5.50E-01
103835703	77	74	103	98	52	41	80	60	-7.07E-01	3.45E-02	2.94E-01
103836212	76	40	61	63	36	61	174	71	2.40E-01	4.36E-01	8.11E-01
103836224	6019	4736	7158	5319	796	1666	3508	3336	-1.58E+00	7.72E-09	2.50E-06
103836225	60	65	97	145	63	129	268	128	4.36E-01	2.27E-01	6.67E-01
103836728	315	144	198	195	161	147	344	280	-8.35E-03	9.89E-01	1.00E+00
103837205	57	43	54	65	53	54	115	74	2.76E-01	3.18E-01	7.42E-01
103837671	1126	781	957	891	746	559	1697	1062	-6.84E-02	7.24E-01	9.35E-01
103837846	77	56	68	74	18	57	128	32	-5.24E-01	1.93E-01	6.28E-01
103837904	370	242	335	315	154	264	679	354	-6.21E-02	7.34E-01	9.38E-01
103838043	326	241	322	291	246	143	504	322	-1.34E-01	6.07E-01	8.91E-01
103838363	0	0	0	0	0	0	0	0			
103839088	154	126	162	137	94	104	340	154	-1.58E-02	9.42E-01	9.96E-01
103841367	773	537	587	801	525	444	1319	666	-5.91E-02	7.61E-01	9.47E-01
103841973	260	170	107	292	245	172	478	310	4.09E-01	2.10E-01	6.49E-01
103842224	0	0	0	1	1	1	0	0			
103842591	419	373	269	348	194	132	517	329	-5.11E-01	4.89E-02	3.53E-01
103842888	578	440	462	462	292	341	965	544	-1.03E-01	5.02E-01	8.44E-01
103843406	18	22	28	14	5	25	29	30	-1.05E-01	8.66E-01	9.77E-01
103843407	0	15	4	3	3	0	9	0			
103844934	882	682	666	875	502	579	1492	795	-9.80E-02	5.17E-01	8.50E-01
103845522	1181	1156	1814	1106	395	596	1692	780	-9.05E-01	1.79E-04	1.08E-02
103845742	2759	1656	2376	1970	1178	1122	3151	1932	-4.45E-01	1.21E-02	1.72E-01
103845884	168	127	143	179	110	97	218	149	-2.49E-01	3.15E-01	7.39E-01
103846914	223	129	223	152	107	82	325	191	-2.92E-01	2.87E-01	7.20E-01
103848200	1	0	0	2	0	1	1	0			

a. Blast search of tyrosine_decarboxylase (PAAS)

```
BLASTP 2.5.0+
Reference: Stephen F. Altschul, Thomas L. Madden, Alejandro
A. Schaffer, Jinghui Zhang, Zheng Zhang, Webb Miller, and
David J. Lipman (1997), "Gapped BLAST and PSI-BLAST: a new
generation of protein database search programs", Nucleic
Acids Res. 25:3389-3402.


Reference for compositional score matrix adjustment: Stephen
F. Altschul, John C. Wootton, E. Michael Gertz, Richa
Agarwala, Aleksandr Morgulis, Alejandro A. Schaffer, and
Yi-Kuo Yu (2005) "Protein database searches using
compositionally adjusted substitution matrices", FEBS J.
272:5101-5109.


RID: WZ1TSDAX01R


Database: NCBI Protein Reference Sequences
           67,628,579 sequences; 25,829,655,146 total letters
Query= Query_155969 gi|18491209|gb|AAL69507.1| putative tyrosine
decarboxylase [Arabidopsis thaliana]

Length=490


                                                                      Score     E
Sequences producing significant alignments:                          (Bits)  Value

XP_009112658.1  PREDICTED: tyrosine decarboxylase 1 [Brassica ...  929     0.0
XP_009117497.1  PREDICTED: tyrosine decarboxylase 1-like [Bras...  927     0.0
XP_009102040.1  PREDICTED: tyrosine decarboxylase 1-like [Bras...  898     0.0
XP_009102041.1  PREDICTED: tyrosine decarboxylase 1-like [Bras...  887     0.0
XP_009129414.1  PREDICTED: probable tyrosine decarboxylase 2 [...  727     0.0

ALIGNMENTS
>XP_009112658.1 PREDICTED: tyrosine decarboxylase 1 [Brassica rapa]
Length=490

 Score = 929 bits (2401),  Expect = 0.0, Method: Compositional matrix adjust.
 Identities = 440/490 (90%), Positives = 465/490 (95%), Gaps = 0/490 (0%)

Query  1    MENGSGKVLKPMDSEQLREYGHLMVDFIADYYKTIEDFPVLSQVQPGYLHKLLPDSAPDH  60
            MENGS  VLKPMDSEQLREYGH MVDFIADYYKTIE FPVLSQVQPGYLH LLPDSAPDH
Sbjct  1    MENGSRHVLKPMDSEQLREYGHRMVDFIADYYKTIESFPVLSQVQPGYLHNLLPDSAPDH  60

Query  61   PETLDQVLDDVRAKILPGVTHWQSPSFFAYYPSNSSVAGFLGEMLSAGLGIVGFSWVTSP  120
            PET++QVLDDV+ KILPGVTHWQSP+FFAYYPSNSSVAGFLGEMLSAG+GIVGFSWVTSP
Sbjct  61   PETVEQVLDDVKTKILPGVTHWQSPNFFAYYPSNSSVAGFLGEMLSAGVGIVGFSWVTSP  120

Query  121  AATELEMIVLDWVAKLLNLPEQFMSKGNGGGVIQGSASEAVLVVLIAARDKVLRSVGKNA  180
            AATELEMIVLDW+AKLLNLPE F+SKGNGGGVIQGSASEA+LVV+IAARDKVLRS GKN+
Sbjct  121  AATELEMIVLDWLAKLLNLPEHFLSKGNGGGVIQGSASEAILVVMIAARDKVLRSAGKNS  180

Query  181  LEKLVVYSSDQTHSALQKACQIAGIHPENCRVLTTDSSTNYALRPESLQEAVSRDLEAGL  240
            L KLVVYSSDQTHSALQKACQIAGIHPENCRVL  DSSTNYALRPE LQEAVSRDLEAGL
Sbjct  181  LGKLVVYSSDQTHSALQKACQIAGIHPENCRVLKADSSTNYALRPELLQEAVSRDLEAGL  240

Query  241  IPFFLCANVGTTSSTAVDPLAALGKIANSNGIWFHVDAAYAGSACICPEYRQYIDGVETA  300
            IPFFLC NVGTTSS AVDPLAALGKIA SN IWFHVDAAYAGSACICPEYRQYIDGVETA
Sbjct  241  IPFFLCGNVGTTSSAAVDPLAALGKIAKSNEIWFHVDAAYAGSACICPEYRQYIDGVETA  300

Query  301  DSFNMNAHKWFLTNFDCSLLWVKDQDSLTLALSTNPEFLKNKASQANLVVDYKDWQIPLG  360
            DSFNMNAHKWFLTNFDCSLLWVKDQ +LT ALSTNPEFLKNKASQANLVVDYKDWQIPLG
Sbjct  301  DSFNMNAHKWFLTNFDCSLLWVKDQHALTEALSTNPEFLKNKASQANLVVDYKDWQIPLG  360
```

```
Query  361  RRFRSLKLWMVLRLYGSETLKSYIRNHIKLAKEFEQLVSQDPNFEIVTPRIFALVCFRLV  420
            RRFRSLKLWMVLRLYG+ETLK+YIRNHIKLAK+ EQLVSQDPNFE++TPRIF+LVCFR+V
Sbjct  361  RRFRSLKLWMVLRLYGAETLKNYIRNHIKLAKDLEQLVSQDPNFEVITPRIFSLVCFRIV  420

Query  421  PVKDEEKKCNNRNRELLDAVNSSGKLFMSHTALSGKIVLRCAIGAPLTEEKHVKEAWKII  480
            P  ++EKKCN+RN ELL+AVNSSGKLF+SHTALSGKIVLRCAIGAPLTEEKHVKE WK+I
Sbjct  421  PTDNDEKKCNSRNLELLEAVNSSGKLFISHTALSGKIVLRCAIGAPLTEEKHVKETWKVI  480

Query  481  QEEASYLLHK  490
            QE+ SYLL K
Sbjct  481  QEKVSYLLRK  490


>XP_009117497.1 PREDICTED: tyrosine decarboxylase 1-like [Brassica rapa]
Length=490

 Score = 927 bits (2397),  Expect = 0.0, Method: Compositional matrix adjust.
 Identities = 440/490 (90%), Positives = 465/490 (95%), Gaps = 0/490 (0%)

Query  1    MENGSGKVLKPMDSEQLREYGHLMVDFIADYYKTIEDFPVLSQVQPGYLHKLLPDSAPDH  60
            MENGS  VLKPMDSEQLREYGH MVDFIADYYKTIE FPVLSQVQPGYLH LLPDSAPD
Sbjct  1    MENGSRNVLKPMDSEQLREYGHRMVDFIADYYKTIETFPVLSQVQPGYLHNLLPDSAPDQ  60

Query  61   PETLDQVLDDVRAKILPGVTHWQSPSFFAYYPSNSSVAGFLGEMLSAGLGIVGFSWVTSP  120
            PET++QVLDDV+ KILPG+THWQSP+F+AYYPSNSSVAGFLGEMLSAGLGIVGFSWVTSP
Sbjct  61   PETVEQVLDDVKTKILPGITHWQSPTFYAYYPSNSSVAGFLGEMLSAGLGIVGFSWVTSP  120

Query  121  AATELEMIVLDWVAKLLNLPEQFMSKGNGGGVIQGSASEAVLVVLIAARDKVLRSVGKNA  180
            AATELEMIVLDW+AKLLNLPEQF+SKGNGGGVIQGSASEA+LVV+I AR+KVLR VGKNA
Sbjct  121  AATELEMIVLDWLAKLLNLPEQFLSKGNGGGVIQGSASEAILVVMIGAREKVLRRVGKNA  180

Query  181  LEKLVVYSSDQTHSALQKACQIAGIHPENCRVLTTDSSTNYALRPESLQEAVSRDLEAGL  240
            L KLVVYSSDQTHSALQKACQIAGIHPENCRVL  DSSTNYALRPE LQEAVS+D+EAGL
Sbjct  181  LGKLVVYSSDQTHSALQKACQIAGIHPENCRVLKADSSTNYALRPELLQEAVSKDIEAGL  240

Query  241  IPFFLCANVGTTSSTAVDPLAALGKIANSNGIWFHVDAAYAGSACICPEYRQYIDGVETA  300
            IPFFLC NVGTTSSTAVDPLAALGKIA SN IWFHVDAAYAGSACICPEYRQYIDGVETA
Sbjct  241  IPFFLCGNVGTTSSTAVDPLAALGKIAKSNEIWFHVDAAYAGSACICPEYRQYIDGVETA  300

Query  301  DSFNMNAHKWFLTNFDCSLLWVKDQDSLTLALSTNPEFLKNKASQANLVVDYKDWQIPLG  360
            DSFNMNAHKWFLTNFDCSLLWVKDQ  LT ALSTNPEFLKNKASQANLVVDYKDWQIPLG
Sbjct  301  DSFNMNAHKWFLTNFDCSLLWVKDQYVLTEALSTNPEFLKNKASQANLVVDYKDWQIPLG  360

Query  361  RRFRSLKLWMVLRLYGSETLKSYIRNHIKLAKEFEQLVSQDPNFEIVTPRIFALVCFRLV  420
            RRFRSLKLWMVLRLYG+ETLKSYIRNHIKLAK+ EQLVSQDPNFE+VTPRIF+LVCFR++
Sbjct  361  RRFRSLKLWMVLRLYGAETLKSYIRNHIKLAKDLEQLVSQDPNFEVVTPRIFSLVCFRIL  420

Query  421  PVKDEEKKCNNRNRELLDAVNSSGKLFMSHTALSGKIVLRCAIGAPLTEEKHVKEAWKII  480
            PV ++EK+CNNRNR LLDAVNSSGKLF+SHTALSGKIVLRCAIGAPLTEE+HVKE WK+I
Sbjct  421  PVDNDEKECNNRNRNLLDAVNSSGKLFLSHTALSGKIVLRCAIGAPLTEERHVKETWKVI  480

Query  481  QEEASYLLHK  490
            QEEAS LL K
Sbjct  481  QEEASRLLGK  490


>XP_009102040.1 PREDICTED: tyrosine decarboxylase 1-like [Brassica rapa]
Length=494

 Score = 898 bits (2321),  Expect = 0.0, Method: Compositional matrix adjust.
 Identities = 429/489 (88%), Positives = 455/489 (93%), Gaps = 1/489 (0%)

Query  1    MENGS-GKVLKPMDSEQLREYGHLMVDFIADYYKTIEDFPVLSQVQPGYLHKLLPDSAPD  59
            MENGS  K LKPMDSEQLREYGH MVDFIADYYKTIE FPVLSQVQPGYLH LLPDSAPD
Sbjct  1    MENGSSNKALKPMDSEQLREYGHRMVDFIADYYKTIETFPVLSQVQPGYLHNLLPDSAPD  60

Query  60   HPETLDQVLDDVRAKILPGVTHWQSPSFFAYYPSNSSVAGFLGEMLSAGLGIVGFSWVTS  119
             PETL+QVLDDV+ KILPGVTHWQSPSFFAYYP+NSSVAGFLGEMLSA L IVGFSWV+S
Sbjct  61   QPETLEQVLDDVKEKILPGVTHWQSPSFFAYYPANSSVAGFLGEMLSAALNIVGFSWVSS  120
```

```
Query  120  PAATELEMIVLDWVAKLLNLPEQFMSKGNGGGVIQGSASEAVLVVLIAARDKVLRSVGKN  179
            PAATELEMIVLDW AKLLNLPEQF+S+GNGGGVIQG+ASEA+LVV+IAARDKVLRS+GK
Sbjct  121  PAATELEMIVLDWFAKLLNLPEQFLSRGNGGGVIQGTASEAILVVMIAARDKVLRSLGKK  180

Query  180  ALEKLVVYSSDQTHSALQKACQIAGIHPENCRVLTTDSSTNYALRPESLQEAVSRDLEAG  239
            ALEKLVVYSSDQTHS+L KACQIAGIH ENCR+L TDSSTNYALRPESLQEAVS DLEAG
Sbjct  181  ALEKLVVYSSDQTHSSLLKACQIAGIHLENCRMLKTDSSTNYALRPESLQEAVSGDLEAG  240

Query  240  LIPFFLCANVGTTSSTAVDPLAALGKIANSNGIWFHVDAAYAGSACICPEYRQYIDGVET  299
            LIPFFLC  VGTTSSTAVDPLA LGKIA SN +WFHVDAAYAGSACICPEYRQYIDGVET
Sbjct  241  LIPFFLCGTVGTTSSTAVDPLAELGKIAKSNEMWFHVDAAYAGSACICPEYRQYIDGVET  300

Query  300  ADSFNMNAHKWFLTNFDCSLLWVKDQDSLTLALSTNPEFLKNKASQANLVVDYKDWQIPL  359
            ADSFNMNAHKWFLTNFDCSLLWVKD+ +LT ALSTNPEFLKNKASQANLVVDYKDWQIPL
Sbjct  301  ADSFNMNAHKWFLTNFDCSLLWVKDRYALTEALSTNPEFLKNKASQANLVVDYKDWQIPL  360

Query  360  GRRFRSLKLWMVLRLYGSETLKSYIRNHIKLAKEFEQLVSQDPNFEIVTPRIFALVCFRL  419
            GRRFRSLKLWMVLRLYG+ETLKSYIRNHIKLAK+ EQLVSQDPNFE+VTPRIF+LVCFR+
Sbjct  361  GRRFRSLKLWMVLRLYGAETLKSYIRNHIKLAKDLEQLVSQDPNFEVVTPRIFSLVCFRI  420

Query  420  VPVKDEEKKCNNRNRELLDAVNSSGKLFMSHTALSGKIVLRCAIGAPLTEEKHVKEAWKI  479
              V ++EK CNN NR LLDAVNSSGKLF+SHT LSGK VLR AIGAPLTEEKHV +AWK+
Sbjct  421  AAVDNDEKTCNNLNRSLLDAVNSSGKLFISHTTLSGKFVLRLAIGAPLTEEKHVMDAWKV  480

Query  480  IQEEASYLL  488
            IQEEAS+LL
Sbjct  481  IQEEASFLL  489


>XP_009102041.1 PREDICTED: tyrosine decarboxylase 1-like [Brassica rapa]
Length=491

 Score = 887 bits (2292),  Expect = 0.0, Method: Compositional matrix adjust.
 Identities = 421/491 (86%), Positives = 453/491 (92%), Gaps = 1/491 (0%)

Query  1    MENG-SGKVLKPMDSEQLREYGHLMVDFIADYYKTIEDFPVLSQVQPGYLHKLLPDSAPD  59
            MENG S   LKPMDSEQLREYGH MVDF+ADYYKTIE FPVLSQVQPGYLH LLP+SAPD
Sbjct  1    MENGRSNNALKPMDSEQLREYGHRMVDFVADYYKTIETFPVLSQVQPGYLHNLLPESAPD  60

Query  60   HPETLDQVLDDVRAKILPGVTHWQSPSFFAYYPSNSSVAGFLGEMLSAGLGIVGFSWVTS  119
            HPET++QVLDDV+AKILPGVTHWQSPSFFAY+P N S+AGFLGEMLSAGL  + FSWV S
Sbjct  61   HPETVEQVLDDVKAKILPGVTHWQSPSFFAYFPINGSIAGFLGEMLSAGLNTMSFSWVAS  120

Query  120  PAATELEMIVLDWVAKLLNLPEQFMSKGNGGGVIQGSASEAVLVVLIAARDKVLRSVGKN  179
            PAATELE++V+DWVAKLLNLPEQF+SKGNGGGVIQGSA EA+LVV+IAARDKVLRSVGK
Sbjct  121  PAATELEIVVVDWVAKLLNLPEQFLSKGNGGGVIQGSACEAILVVMIAARDKVLRSVGKK  180

Query  180  ALEKLVVYSSDQTHSALQKACQIAGIHPENCRVLTTDSSTNYALRPESLQEAVSRDLEAG  239
            ALEKLVVYSSDQTHS+LQKACQ+AGIH ENCRVL TDSSTNYALRPESLQEAVS+DLEAG
Sbjct  181  ALEKLVVYSSDQTHSSLQKACQLAGIHLENCRVLKTDSSTNYALRPESLQEAVSKDLEAG  240

Query  240  LIPFFLCANVGTTSSTAVDPLAALGKIANSNGIWFHVDAAYAGSACICPEYRQYIDGVET  299
            LIPFFLC  VGTTSSTAVDPLAALGKIA SN +WFH+DAAYAGSACICPEYRQYIDGVET
Sbjct  241  LIPFFLCGTVGTTSSTAVDPLAALGKIAKSNEMWFHIDAAYAGSACICPEYRQYIDGVET  300

Query  300  ADSFNMNAHKWFLTNFDCSLLWVKDQDSLTLALSTNPEFLKNKASQANLVVDYKDWQIPL  359
            ADSF+MNAHKW LTNF+CSLLWVKDQ +LT ALSTNPE+LKNKASQANLVVDYKDWQIPL
Sbjct  301  ADSFDMNAHKWLLTNFECSLLWVKDQSALTEALSTNPEYLKNKASQANLVVDYKDWQIPL  360

Query  360  GRRFRSLKLWMVLRLYGSETLKSYIRNHIKLAKEFEQLVSQDPNFEIVTPRIFALVCFRL  419
            GRRFRSLKLWMVLRLYG+E LKSYIRNHIKLAK FEQLVS+DPNFE+VTPRIF+LVCFR+
Sbjct  361  GRRFRSLKLWMVLRLYGAENLKSYIRNHIKLAKIFEQLVSKDPNFEVVTPRIFSLVCFRI  420

Query  420  VPVKDEEKKCNNRNRELLDAVNSSGKLFMSHTALSGKIVLRCAIGAPLTEEKHVKEAWKI  479
              V ++EKKCNN NR LLDAVNSSGKL  SHT LS K VLR AIGAPLTEEKHVKEAWK+
Sbjct  421  AAVDNDEKKCNNLNRSLLDAVNSSGKLLFSHTILSEKFVLRFAIGAPLTEEKHVKEAWKV  480

Query  480  IQEEASYLLHK  490
```

```
            IQEEAS+LL K
Sbjct  481  IQEEASFLLSK  491


>XP_009129414.1 PREDICTED: probable tyrosine decarboxylase 2 [Brassica rapa]
Length=556

 Score = 727 bits (1876),  Expect = 0.0, Method: Compositional matrix adjust.
 Identities = 340/498 (68%), Positives = 409/498 (82%), Gaps = 11/498 (2%)

Query  3    NGSGKV-------LKPMDSEQLREYGHLMVDFIADYYKTIED----FPVLSQVQPGYLHK  51
            NG+GKV       +KPMDSE LRE GH+MVDFIADYYK +ED    FPVLSQVQPGYL
Sbjct  55   NGNGKVNGEKMKKMKPMDSEMLREQGHIMVDFIADYYKNLEDSPQDFPVLSQVQPGYLRD  114

Query  52   LLPDSAPDHPETLDQVLDDVRAKILPGVTHWQSPSFFAYYPSNSSVAGFLGEMLSAGLGI  111
            +LPDSAPDHPE L ++L DV  KI+PG+THWQSPS+FAYY S++SVAGFLGEML+AGL +
Sbjct  115  ILPDSAPDHPEPLKELLHDVSKKIIPGLTHWQSPSYFAYYASSTSVAGFLGEMLNAGLSV  174

Query  112  VGFSWVTSPAATELEMIVLDWVAKLLNLPEQFMSKGNGGGVIQGSASEAVLVVLIAARDK  171
            VGF+W+TSPAATELE+IVLDW+AKLL LP+ F+S G GGGVIQG+  EAVLVV++AARD+
Sbjct  175  VGFTWLTSPAATELEVIVLDWLAKLLQLPDHFLSTGRGGGVIQGTGCEAVLVVVLAARDR  234

Query  172  VLRSVGKNALEKLVVYSSDQTHSALQKACQIAGIHPENCRVLTTDSSTNYALRPESLQEA  231
            +++  GKN+L +LVVY+SDQTHS+ +KAC I GIH EN R+L TDSSTNY + P+SL+EA
Sbjct  235  IMKKAGKNSLSQLVVYASDQTHSSFRKACLIGGIHEENIRLLKTDSSTNYGMPPKSLEEA  294

Query  232  VSRDLEAGLIPFFLCANVGTTSSTAVDPLAALGKIANSNGIWFHVDAAYAGSACICPEYR  291
            +S DL  G IPFF+CA VGTTSS AVDPL  LGKIA S GIW HVDAAYAG+ACICPE+R
Sbjct  295  ISSDLAKGFIPFFICATVGTTSSAAVDPLVPLGKIAKSYGIWMHVDAAYAGNACICPEFR  354

Query  292  QYIDGVETADSFNMNAHKWFLTNFDCSLLWVKDQDSLTLALSTNPEFLKNKASQANLVVD  351
            ++IDGVE ADSFN+NAHKW   N  CS LWVKD+ SL  AL TNPE+L+ K S+ + VV+
Sbjct  355  KHIDGVENADSFNINAHKWLFANQTCSPLWVKDRYSLIDALKTNPEYLEYKVSKRDEVVN  414

Query  352  YKDWQIPLGRRFRSLKLWMVLRLYGSETLKSYIRNHIKLAKEFEQLVSQDPNFEIVTPRI  411
            YKDWQI L RRFRSLKLWMVLRLYG+E L+++IR+H+ LAK FE  V+QD +FE+VT R
Sbjct  415  YKDWQISLSRRFRSLKLWMVLRLYGAENLRNFIRDHVNLAKNFEDYVAQDAHFEVVTTRY  474

Query  412  FALVCFRLVPVKDEEKKCNNRNRELLDAVNSSGKLFMSHTALSGKIVLRCAIGAPLTEEK  471
            F+LVCFRL PV  +E KCN RNRELL  VNS+GK+F+SHTALSGK +LR A+GAPLTEEK
Sbjct  475  FSLVCFRLAPVDGDEDKCNERNRELLATVNSTGKIFISHTALSGKFILRFAVGAPLTEEK  534

Query  472  HVKEAWKIIQEEASYLLH  489
            HV EAW+IIQ+ A+  +H
Sbjct  535  HVTEAWRIIQKHATEFIH  552


  Database: NCBI Protein Reference Sequences
    Posted date:  Sep 1, 2016  7:11 AM
  Number of letters in database: 19,254,814
  Number of sequences in database:  46,093

Lambda      K        H
   0.319    0.134    0.400
Gapped
Lambda      K        H
   0.267   0.0410    0.140
Matrix: BLOSUM62
Gap Penalties: Existence: 11, Extension: 1
Number of Sequences: 46093
Number of Hits to DB: 453
Number of extensions: 0
Number of successful extensions: 0
Number of sequences better than 100: 0
Number of HSP's better than 100 without gapping: 0
Number of HSP's gapped: 0
Number of HSP's successfully gapped: 0
Length of query: 490
Length of database: 19254814
```

```
Length adjustment: 106
Effective length of query: 384
Effective length of database: 14368956
Effective search space: 5517679104
Effective search space used: 5517679104
T: 21
A: 40
X1: 16 (7.4 bits)
X2: 38 (14.6 bits)
X3: 64 (24.7 bits)
S1: 41 (20.4 bits)
S2: 55 (25.8 bits)
ka-blk-alpha gapped: 1.9
ka-blk-alpha ungapped: 0.7916
ka-blk-alpha_v gapped: 42.6028
ka-blk-alpha_v ungapped: 4.96466
ka-blk-sigma gapped: 43.6362
```

b. Blast search of phenylalanine_N-monooxygenase (P450 CYP79A2)

BLASTP 2.5.0+
Reference: Stephen F. Altschul, Thomas L. Madden, Alejandro A. Schaffer, Jinghui Zhang, Zheng Zhang, Webb Miller, and David J. Lipman (1997), "Gapped BLAST and PSI-BLAST: a new generation of protein database search programs", Nucleic Acids Res. 25:3389-3402.

Reference for compositional score matrix adjustment: Stephen F. Altschul, John C. Wootton, E. Michael Gertz, Richa Agarwala, Aleksandr Morgulis, Alejandro A. Schaffer, and Yi-Kuo Yu (2005) "Protein database searches using compositionally adjusted substitution matrices", FEBS J. 272:5101-5109.

RID: WZ24DCFS014

Database: NCBI Protein Reference Sequences
67,628,579 sequences; 25,829,655,146 total letters
Query= Query_184487 gi|13878375|sp|Q9FLC8.1|C79A2_ARATH RecName: Full=Phenylalanine N-monooxygenase; AltName: Full=Cytochrome P450 79A2; AltName: Full=Phenylalanine N-hydroxylase

Length=529

Sequences producing significant alignments:		Score (Bits)	E Value
XP_009125594.1	PREDICTED: phenylalanine N-monooxygenase-like ...	922	0.0
XP_009122135.1	PREDICTED: phenylalanine N-monooxygenase [Bras...	890	0.0
XP_009140345.1	PREDICTED: tryptophan N-monooxygenase 2 [Brass...	554	0.0
XP_009129057.1	PREDICTED: cytochrome P450 79B1 [Brassica rapa]	550	0.0
XP_009138546.1	PREDICTED: cytochrome P450 79B1-like [Brassica...	543	0.0
XP_009142023.1	PREDICTED: dihomomethionine N-hydroxylase-like...	451	3e-154
XP_009132654.1	PREDICTED: dihomomethionine N-hydroxylase [Bra...	442	1e-150
XP_009149073.1	PREDICTED: dihomomethionine N-hydroxylase-like...	400	3e-134
XP_009144065.1	PREDICTED: dihomomethionine N-hydroxylase-like...	395	1e-131
XP_009119683.1	PREDICTED: cytochrome P450 703A2 [Brassica rapa]	301	4e-96
XP_009118885.1	PREDICTED: LOW QUALITY PROTEIN: cytochrome P45...	289	2e-91
XP_009122411.1	PREDICTED: flavonoid 3'-monooxygenase [Brassic...	263	2e-81
XP_009141948.1	PREDICTED: LOW QUALITY PROTEIN: cytochrome P45...	229	1e-68
XP_009136876.1	PREDICTED: cytochrome P450 81D11-like [Brassic...	223	2e-66
XP_009133464.1	PREDICTED: cytochrome P450 98A3 [Brassica rapa]	223	2e-66
XP_009101692.1	PREDICTED: LOW QUALITY PROTEIN: cytochrome P45...	223	6e-66
XP_009138414.1	PREDICTED: cytochrome P450 84A1-like [Brassica...	222	7e-66
XP_009109920.1	PREDICTED: cytochrome P450 82C4 [Brassica rapa]	219	2e-64
XP_009142348.1	PREDICTED: cytochrome P450 76C4-like [Brassica...	216	1e-63
XP_009108580.1	PREDICTED: LOW QUALITY PROTEIN: geraniol 8-hyd...	215	3e-63
XP_009142346.1	PREDICTED: cytochrome P450 76C4-like [Brassica...	214	8e-63
XP_009103435.1	PREDICTED: cytochrome P450 81D1-like [Brassica...	213	1e-62
XP_009134258.1	PREDICTED: cytochrome P450 76C2-like [Brassica...	213	3e-62
XP_009129359.1	PREDICTED: cytochrome P450 81D11-like [Brassic...	211	6e-62
XP_009150750.1	PREDICTED: cytochrome P450 84A1-like [Brassica...	211	7e-62
XP_009112368.1	PREDICTED: cytochrome P450 81D1-like [Brassica...	211	9e-62
XP_009136071.1	PREDICTED: geraniol 8-hydroxylase [Brassica rapa]	209	5e-61
XP_009151853.1	PREDICTED: cytochrome P450 81D11 [Brassica rapa]	208	8e-61
XP_009114628.1	PREDICTED: cytochrome P450 76C1-like [Brassica...	209	8e-61
XP_009129360.1	PREDICTED: cytochrome P450 81D11-like [Brassic...	208	9e-61
XP_009144655.1	PREDICTED: cytochrome P450 81D1 [Brassica rapa]	207	2e-60
XP_009111606.1	PREDICTED: cytochrome P450 81D11 [Brassica rapa]	206	4e-60
XP_009142712.1	PREDICTED: cytochrome P450 98A3-like [Brassica...	206	4e-60
XP_009144487.1	PREDICTED: geraniol 8-hydroxylase-like [Brassi...	206	6e-60
XP_009137274.1	PREDICTED: geraniol 8-hydroxylase-like [Brassi...	206	1e-59
XP_009145966.1	PREDICTED: cytochrome P450 84A1 [Brassica rapa]	204	5e-59

XP_009136332.1	PREDICTED: cytochrome P450 81F1 [Brassica rapa]	203	9e-59
XP_009117291.1	PREDICTED: cytochrome P450 81D11 [Brassica rapa]	202	1e-58
XP_009134259.1	PREDICTED: cytochrome P450 76C2-like isoform X...	202	2e-58
XP_009103432.1	PREDICTED: cytochrome P450 81D1-like isoform X...	202	2e-58
XP_009142349.1	PREDICTED: cytochrome P450 76C3-like [Brassica...	202	2e-58
XP_009116769.1	PREDICTED: cytochrome P450 76C3 [Brassica rapa]	201	3e-58
XP_009102613.1	PREDICTED: cytochrome P450 76C4-like [Brassica...	201	5e-58
XP_009133990.1	PREDICTED: cytochrome P450 81D11-like [Brassic...	201	6e-58
XP_009136438.1	PREDICTED: cytochrome P450 81F1-like [Brassica...	200	1e-57
XP_009142347.1	PREDICTED: cytochrome P450 76C2-like [Brassica...	200	1e-57
XP_009129560.1	PREDICTED: cytochrome P450 71B23-like [Brassic...	199	2e-57
XP_009138490.1	PREDICTED: cytochrome P450 81D11-like [Brassic...	199	2e-57
XP_009101907.1	PREDICTED: cytochrome P450 84A1-like [Brassica...	199	3e-57
XP_009137862.1	PREDICTED: cytochrome P450 CYP82D47-like [Bras...	199	6e-57
XP_009125667.1	PREDICTED: 3,9-dihydroxypterocarpan 6A-monooxy...	198	8e-57
XP_009151334.1	PREDICTED: cytochrome P450 71A23-like [Brassic...	197	1e-56
XP_009126851.1	PREDICTED: cytochrome P450 81F1-like [Brassica...	197	1e-56
XP_009136105.1	PREDICTED: cytochrome P450 82G1 [Brassica rapa]	197	1e-56
XP_009148545.1	PREDICTED: cytochrome P450 71B7-like [Brassica...	197	2e-56
XP_009120246.1	PREDICTED: cytochrome P450 81F1 [Brassica rapa]	196	3e-56
XP_009150729.1	PREDICTED: cytochrome P450 71B7-like [Brassica...	196	4e-56
XP_009132142.1	PREDICTED: cytochrome P450 81F1-like [Brassica...	195	8e-56
XP_009152069.1	PREDICTED: cytochrome P450 71B23 [Brassica rapa]	195	8e-56
XP_009136992.1	PREDICTED: cytochrome P450 81D11 [Brassica rapa]	194	9e-56
XP_009138491.1	PREDICTED: cytochrome P450 81D11-like [Brassic...	194	2e-55
XP_009116856.1	PREDICTED: cytochrome P450 78A9 [Brassica rapa]	193	8e-55
XP_009110601.1	PREDICTED: cytochrome P450 71B2-like [Brassica...	191	2e-54
XP_009138745.1	PREDICTED: LOW QUALITY PROTEIN: cytochrome P45...	192	2e-54
XP_009118796.1	PREDICTED: cytochrome P450 78A6-like [Brassica...	192	3e-54
XP_009109368.1	PREDICTED: cytochrome P450 81F1-like [Brassica...	191	3e-54
XP_009118025.1	PREDICTED: cytochrome P450 71B7 [Brassica rapa]	191	4e-54
XP_009132738.1	PREDICTED: trans-cinnamate 4-monooxygenase-lik...	190	4e-54
XP_009107632.1	PREDICTED: cytochrome P450 83B1 [Brassica rapa]	189	1e-53
XP_009138804.1	PREDICTED: cytochrome P450 76C4-like [Brassica...	189	1e-53
XP_009138803.1	PREDICTED: cytochrome P450 76C4-like [Brassica...	188	3e-53
XP_009148546.1	PREDICTED: cytochrome P450 71B7-like [Brassica...	187	5e-53
XP_009143151.1	PREDICTED: cytochrome P450 76C3-like [Brassica...	187	5e-53
XP_009150199.1	PREDICTED: cytochrome P450 71A26 [Brassica rapa]	186	2e-52
XP_009118033.1	PREDICTED: cytochrome P450 71B2-like [Brassica...	186	3e-52
XP_009129563.1	PREDICTED: LOW QUALITY PROTEIN: cytochrome P45...	186	3e-52
NP_001288861.1	trans-cinnamate 4-monooxygenase [Brassica rapa]	185	5e-52
XP_009142434.1	PREDICTED: cytochrome P450 78A6 [Brassica rapa]	185	5e-52
XP_009140526.1	PREDICTED: cytochrome P450 705A5-like [Brassic...	184	2e-51
XP_009109371.1	PREDICTED: cytochrome P450 81F1-like [Brassica...	183	2e-51
XP_009136769.1	PREDICTED: cytochrome P450 81F1 [Brassica rapa]	182	4e-51
XP_009103856.1	PREDICTED: cytochrome P450 71B5 [Brassica rapa]	182	4e-51
XP_009140379.1	PREDICTED: cytochrome P450 71B2-like [Brassica...	182	5e-51
XP_009110600.1	PREDICTED: cytochrome P450 71B2-like [Brassica...	181	1e-50
NP_001306250.1	cytochrome P450 81F1-like [Brassica rapa]	181	1e-50
XP_009137084.1	PREDICTED: cytochrome P450 81D1 [Brassica rapa]	181	2e-50
XP_009119678.1	PREDICTED: cytochrome P450 78A6 [Brassica rapa]	181	3e-50
XP_009126913.1	PREDICTED: cytochrome P450 71B2-like [Brassica...	180	4e-50
XP_009103433.1	PREDICTED: cytochrome P450 81D1-like isoform X...	179	4e-50
XP_009145675.1	PREDICTED: cytochrome P450 705A20-like [Brassi...	179	5e-50
XP_009104755.1	PREDICTED: cytochrome P450 98A8-like [Brassica...	179	6e-50
XP_009141067.1	PREDICTED: trans-cinnamate 4-monooxygenase-lik...	179	6e-50
XP_009152073.1	PREDICTED: cytochrome P450 71B19-like [Brassic...	179	7e-50
XP_009142048.1	PREDICTED: 3,9-dihydroxypterocarpan 6A-monooxy...	179	9e-50
XP_009152068.1	PREDICTED: cytochrome P450 71B3-like [Brassica...	179	1e-49
XP_009121600.1	PREDICTED: cytochrome P450 71B4-like [Brassica...	178	2e-49
XP_009128027.1	PREDICTED: cytochrome P450 78A5-like [Brassica...	179	2e-49
XP_009151311.1	PREDICTED: cytochrome P450 71A23-like [Brassic...	177	2e-49
XP_009111243.1	PREDICTED: cytochrome P450 705A20-like [Brassi...	177	4e-49
XP_009142616.1	PREDICTED: cytochrome P450 78A6-like [Brassica...	177	4e-49

ALIGNMENTS
>XP_009125594.1 PREDICTED: phenylalanine N-monooxygenase-like [Brassica rapa]
Length=532

```
 Score = 922 bits (2383),  Expect = 0.0, Method: Compositional matrix adjust.
 Identities = 441/532 (83%), Positives = 486/532 (91%), Gaps = 3/532 (1%)

Query  1    MLDSTPMLAFIIGLLLLALTMK-RKEKKKTMLI-SPTRNLSLPPGPKSWPLIGNLPEILG  58
            MLDST +LAF++G L++ALTMK RKE KK +L+ S TRNL LPPGPKSWPL+GNLPEIL
Sbjct  1    MLDSTSLLAFLLGFLIVALTMKKRKEPKKNVLMTSHTRNLLLPPGPKSWPLVGNLPEILW  60

Query  59   RNKPVFRWIHSLMKELNTDIACIRLANTHVIPVTSPRIAREILKKQDSVFATRPLTMGTE  118
            R +PVFRWIH++M+ELNTDIACI LANT+VIPVTSPRIAREILKKQDS+FATRPLTMGTE
Sbjct  61   RKRPVFRWIHAIMEELNTDIACIPLANTNVIPVTSPRIAREILKKQDSIFATRPLTMGTE  120

Query  119  YCSRGYLTVAVEPQGEQWKKMRRVVASHVTSKKSFQMMLQKRTEEADNLVRYINNRSVKN  178
            YCSRGYLT+AVEPQGEQWKKMRRVVASHVTS+KSF+  L+KRTEEADNLVRYINN  VKN
Sbjct  121  YCSRGYLTIAVEPQGEQWKKMRRVVASHVTSQKSFKWTLEKRTEEADNLVRYINNLCVKN  180

Query  179  RGNAFVVIDLRLAVRQYSGNVARKMMFGIRHFGKGSEDGSGPGLEEIEHVESLFTVLTHL  238
            +GN F VIDLRL VRQYSGNVAR M+FG+RHFGKGSEDG GPG EEIEHV+SLFTV+TH+
Sbjct  181  QGNGFEVIDLRLVVRQYSGNVARNMLFGVRHFGKGSEDGFGPGFEEIEHVDSLFTVVTHI  240

Query  239  YAFALSDYVPWLRFLDLEGHEKVVSNAMRNVSKYNDPFVDERLMQWRNGKMKEPQDFLDM  298
            YAFALSDYVPWLRFLDLEGHEKVVS AMRN+SKYND FVD+RL QWR+GKMKEPQDFLDM
Sbjct  241  YAFALSDYVPWLRFLDLEGHEKVVSGAMRNISKYNDSFVDQRLEQWRDGKMKEPQDFLDM  300

Query  299  FIIAKDTDGKPTLSDEEIKAQVT-ELMLATVDNPSNAAEWGMAEMINEPSIMQKAVEEID  357
            FI+AKDT+G P LSDEEIKAQV  ELMLATVDNPSNAAEW MAEMIN+P+IMQKAVEEID
Sbjct  301  FILAKDTNGNPALSDEEIKAQVRYELMLATVDNPSNAAEWAMAEMINQPNIMQKAVEEID  360

Query  358  RVVGKDRLVIESDLPNLNYVKACVKEAFRLHPVAPFNLPHMSTTDTVVDGYFIPKGSHVL  417
            RVVGKDR V+ESD+ NLNYVKACVKEAFRLHPVAPFNL HMST D VVDGYFIPKGSHVL
Sbjct  361  RVVGKDRFVLESDISNLNYVKACVKEAFRLHPVAPFNLTHMSTADAVVDGYFIPKGSHVL  420

Query  418  ISRMGIGRNPSVWDKPHKFDPERHLSTNTCVDLNESDLNIISFSAGRRGCMGVDIGSAMT  477
            ISR+GIGRNP+VWDKP KFDPERH+  N  V+LN+ DLNIISFSAGRRGCMG +IGSAMT
Sbjct  421  ISRLGIGRNPNVWDKPLKFDPERHMGNNKNVELNDPDLNIISFSAGRRGCMGSNIGSAMT  480

Query  478  YMLLARLIQGFTWLPVPGKNKIDISESKNDLFMAKPLYAVATPRLAPHVYPT  529
            YMLLARLIQGFTW  V G++KIDISESK+DLFMAKPL+A+ATPRLAP +Y T
Sbjct  481  YMLLARLIQGFTWSSVHGEDKIDISESKSDLFMAKPLHAIATPRLAPQIYST  532


>XP_009122135.1 PREDICTED: phenylalanine N-monooxygenase [Brassica rapa]
Length=525

 Score = 890 bits (2299),  Expect = 0.0, Method: Compositional matrix adjust.
 Identities = 436/528 (83%), Positives = 476/528 (90%), Gaps = 6/528 (1%)

Query  1    MLDSTPMLAFIIGLLLLALTMKRKEKKKTMLI-SPTRNLSLPPGPKSWPLIGNLPEILGR  59
            MLDST +LAF +G L+L  T+KRKE KKT+L+ S TRNL LPPGPK WP+IGNLPEIL R
Sbjct  1    MLDSTSLLAFFLGFLILTFTVKRKESKKTVLVTSYTRNLPLPPGPKPWPIIGNLPEILWR  60

Query  60   NKPVFRWIHSLMKELNTDIACIRLANTHVIPVTSPRIAREILKKQDSVFATRPLTMGTEY  119
            NKPVFRWIHSLM+ELNTDIACIRLANTHVIPVTSPRIAREILKKQDS+FATRPLTMGTEY
Sbjct  61   NKPVFRWIHSLMEELNTDIACIRLANTHVIPVTSPRIAREILKKQDSIFATRPLTMGTEY  120

Query  120  CSRGYLTVAVEPQGEQWKKMRRVVASHVTSKKSFQMMLQKRTEEADNLVRYINNRSVKNR  179
             SRGYLT+AVEPQGEQWKKMRRVVASHVT +KSF+  L++RTEEADNLVRYIN     +
Sbjct  121  SSRGYLTIAVEPQGEQWKKMRRVVASHVTCQKSFRWTLEQRTEEADNLVRYINKLCKGSE  180

Query  180  GNAFVVIDLRLAVRQYSGNVARKMMFGIRHFGKGSEDGSGPGLEEIEHVESLFTVLTHLY  239
            G     ID+R  VRQYSGNVARKM+FG+RHFGKGSEDGSGPG EEIEHV+SLF V+THLY
Sbjct  181  G-----IDVRHVVRQYSGNVARKMLFGVRHFGKGSEDGSGPGFEEIEHVDSLFKVVTHLY  235

Query  240  AFALSDYVPWLRFLDLEGHEKVVSNAMRNVSKYNDPFVDERLMQWRNGKMKEPQDFLDMF  299
            AFALSDYVPWLRFLDLEGHEKVVS AMRNVSKYNDP VD+RL  WRNGKM EPQDFLDM
Sbjct  236  AFALSDYVPWLRFLDLEGHEKVVSGAMRNVSKYNDPLVDKRLDLWRNGKMNEPQDFLDML  295

Query  300  IIAKDTDGKPTLSDEEIKAQVTELMLATVDNPSNAAEWGMAEMINEPSIMQKAVEEIDRV  359
            I+AKDT+GKP LSDEEIKAQVTELMLATVDNPSNA EW MAEMINEP+IM+KAVEE+DR+
Sbjct  296  ILAKDTNGKPALSDEEIKAQVTELMLATVDNPSNAVEWAMAEMINEPNIMKKAVEEVDRI  355
```

```
Query  360  VGKDRLVIESDLPNLNYVKACVKEAFRLHPVAPFNLPHMSTTDTVVDGYFIPKGSHVLIS  419
            VGKDRLV+ESDLPNLNY+KACVKEAFRLHPVAPFNLPHMST D VVDGYFIPKGSHVLIS
Sbjct  356  VGKDRLVLESDLPNLNYLKACVKEAFRLHPVAPFNLPHMSTADAVVDGYFIPKGSHVLIS  415

Query  420  RMGIGRNPSVWDKPHKFDPERHLSTNTCVDLNESDLNIISFSAGRRGCMGVDIGSAMTYM  479
            R+GIGRNP+VWDKP KFDPERHL  N  V+LN+ DLNIISFSAGRRGCMG +IGSAMTYM
Sbjct  416  RLGIGRNPNVWDKPLKFDPERHLGDNKNVELNDPDLNIISFSAGRRGCMGSNIGSAMTYM  475

Query  480  LLARLIQGFTWLPVPGKNKIDISESKNDLFMAKPLYAVATPRLAPHVY  527
            LLARLIQGFTW  +PG++K+DI+ESK DLFMAKPL+AV TPRLAPHVY
Sbjct  476  LLARLIQGFTWSSMPGEDKVDITESKTDLFMAKPLHAVGTPRLAPHVY  523


>XP_009140345.1 PREDICTED: tryptophan N-monooxygenase 2 [Brassica rapa]
Length=543

 Score = 554 bits (1427),  Expect = 0.0, Method: Compositional matrix adjust.
 Identities = 268/522 (51%), Positives = 363/522 (70%), Gaps = 10/522 (2%)

Query  8    LAFIIGLLLLALTMKRKEKKKTMLISPTRNLS-LPPGPKSWPLIGNLPEILGRNKPVFRW  66
            L  +  L++++L M  K+ K +   S  + L  LPPGP  +P++G LP +L +N+PVFRW
Sbjct  28   LTTLQALVVISLLMIIKKIKSS---SHNKKLHPLPPGPSGFPIVGMLPAML-KNRPVFRW  83

Query  67   IHSLMKELNTDIACIRLANTHVIPVTSPRIAREILKKQDSVFATRPLTMGTEYCSRGYLT  126
            +HSLMKELNT+IAC+RL NTHVIPVT P+IAREI K+QD++FA+RPLT   +  S GY T
Sbjct  84   LHSLMKELNTEIACVRLGNTHVIPVTCPKIAREIFKQQDALFASRPLTYAQKILSNGYKT  143

Query  127  VAVEPQGEQWKKMRRVVASHVTSKKSFQMMLQKRTEEADNLVRYINNRSVKNRGNAFVVI  186
              + P GEQ+KKMR+V+ + +      + +   R EE D+L  ++ N  VKN       +
Sbjct  144  CVITPFGEQFKKMRKVIMTEIVCPARHRWLHDNRAEETDHLTAWLYNM-VKNSEP----V  198

Query  187  DLRLAVRQYSGNVARKMMFGIRHFGKGSEDGSGPGLEEIEHVESLFTVLTHLYAFALSDY  246
            DLR   R Y GN  +++MFG R F + ++   GP +E+IEH+E++F  L   +AF +SDY
Sbjct  199  DLRFVTRHYCGNAIKRLMFGTRTFSEKTKTDGGPTMEDIEHMEAMFEGLGFTFAFCVSDY  258

Query  247  VPWLRFLDLEGHEKVVSNAMRNVSKYNDPFVDERLMQWRNGKMKEPQDFLDMFIIAKDTD  306
            +P L  LDL GHEK++  A   + KY+DP +DER+  WR GK  + +DFLD+FI  KD D
Sbjct  259  LPMLTGLDLNGHEKIMREASAIMDKYHDPIIDERMKMWREGKRTQIEDFLDIFISIKDAD  318

Query  307  GKPTLSDEEIKAQVTELMLATVDNPSNAAEWGMAEMINEPSIMQKAVEEIDRVVGKDRLV  366
            G P L+ +EIK  + EL++A  DNPSNAAEW MAEMIN+P I+QKA+EEI+RVVGK+RLV
Sbjct  319  GHPLLTADEIKPTIKELVMAAPDNPSNAAEWAMAEMINKPEILQKAMEEIERVVGKERLV  378

Query  367  IESDLPNLNYVKACVKEAFRLHPVAPFNLPHMSTTDTVVDGYFIPKGSHVLISRMGIGRN  426
             ESD+P LNY+KA ++E FRLHPVA FNLPH++ +DT V GY IPKGS VL+SR G+GRN
Sbjct  379  QESDIPKLNYLKAIIRETFRLHPVAAFNLPHVALSDTTVAGYHIPKGSQVLLSRYGLGRN  438

Query  427  PSVWDKPHKFDPERHLSTNTCVDLNESDLNIISFSAGRRGCMGVDIGSAMTYMLLARLIQ  486
            P VW  P  F PERHL+    V L E+DL  ISFS G+RGC    +G+A+T M+LARL+Q
Sbjct  439  PKVWSDPLSFKPERHLNECLEVTLTENDLRFISFSTGKRGCAAPALGTAITVMMLARLLQ  498

Query  487  GFTWLPVPGKNKIDISESKNDLFMAKPLYAVATPRLAPHVYP  528
            GF W    G+ ++++ ES +D+F+AKPL  V   RL+  +YP
Sbjct  499  GFKWKLAGGETRVELMESSHDMFLAKPLVMVGELRLSEELYP  540


>XP_009129057.1 PREDICTED: cytochrome P450 79B1 [Brassica rapa]
Length=540

 Score = 550 bits (1416),  Expect = 0.0, Method: Compositional matrix adjust.
 Identities = 260/502 (52%), Positives = 350/502 (70%), Gaps = 6/502 (1%)

Query  28   KTMLISPTRNLSLPPGPKSWPLIGNLPEILGRNKPVFRWIHSLMKELNTDIACIRLANTH  87
            K +  +  + LSLPPGP  WP+IG +P +L +++PVFRW+HS+MK+LNT+IAC+RL NTH
Sbjct  43   KKVFTTDKKKLSLPPGPTGWPIIGMVPTML-KSRPVFRWLHSIMKQLNTEIACVRLGNTH  101

Query  88   VIPVTSPRIAREILKKQDSVFATRPLTMGTEYCSRGYLTVAVEPQGEQWKKMRRVVASHV  147
            VI VT P+IAREILK+QD++FA+RP+T      S GY T  + P GEQ+KKMR+VV + +
```

```
Sbjct  102  VITVTCPKIAREILKQQDALFASRPMTYAQNVLSNGYKTCVITPFGEQFKKMRKVVMTEL  161

Query  148  TSKKSFQMMLQKRTEEADNLVRYINNRSVKNRGNAFVVIDLRLAVRQYSGNVARKMMFGI  207
                  + + QKR EE D+L  ++ N  VKN G+    +D R   R Y GN  +K+MFG
Sbjct  162  VCPARHRWLHQKRAEENDHLTAWVYNL-VKNSGS----VDFRFVTRHYCGNAIKKLMFGT  216

Query  208  RHFGKGSEDGSGPGLEEIEHVESLFTVLTHLYAFALSDYVPWLRFLDLEGHEKVVSNAMR  267
            R F + +    GP  E+IEH+E++F  L   ++F +SDY+P L  LDL GHEK++ ++
Sbjct  217  RTFSENTAPDGGPTAEDIEHMEAMFEALGFTFSFCISDYLPMLTGLDLNGHEKIMRDSSA  276

Query  268  NVSKYNDPFVDERLMQWRNGKMKEPQDFLDMFIIAKDTDGKPTLSDEEIKAQVTELMLAT  327
             + KY+DP VD R+  WR GK  + +DFLD+FI  KD  G P L+ +EIK  + EL++A
Sbjct  277  IMDKYHDPIVDARIKMWREGKRTQIEDFLDIFISIKDEQGNPLLTADEIKPTIKELVMAA  336

Query  328  VDNPSNAAEWGMAEMINEPSIMQKAVEEIDRVVGKDRLVIESDLPNLNYVKACVKEAFRL  387
             DNPSNA EW MAEM+N+P I+ KA+EEIDRVVGK+RLV ESD+P LNYVKA ++EAFRL
Sbjct  337  PDNPSNAVEWAMAEMVNKPEILHKAMEEIDRVVGKERLVQESDIPKLNYVKAILREAFRL  396

Query  388  HPVAPFNLPHMSTTDTVVDGYFIPKGSHVLISRMGIGRNPSVWDKPHKFDPERHLSTNTC  447
            HPVA FNLPH++ +D  V GY IPKGS VL+SR G+GRNP VW  P  F PERHL+  +
Sbjct  397  HPVAAFNLPHVALSDATVAGYHIPKGSQVLLSRYGLGRNPKVWADPLSFKPERHLNECSE  456

Query  448  VDLNESDLNIISFSAGRRGCMGVDIGSAMTYMLLARLIQGFTWLPVPGKNKIDISESKND  507
            V L E+DL  ISFS G+RGC    +G+A+T M+LARL+QGFT      + ++++ ES +D
Sbjct  457  VTLTENDLRFISFSTGKRGCAAPALGTALTTMMLARLLQGFTSKLPENETRVELMESSHD  516

Query  508  LFMAKPLYAVATPRLAPHVYPT  529
            +F+AKPL  V   RL   +YPT
Sbjct  517  MFLAKPLVMVGELRLPEQLYPT  538


>XP_009138546.1 PREDICTED: cytochrome P450 79B1-like [Brassica rapa]
Length=541

 Score = 543 bits (1400),  Expect = 0.0, Method: Compositional matrix adjust.
 Identities = 261/526 (50%), Positives = 363/526 (69%), Gaps = 15/526 (3%)

Query  5    TPMLAFIIGLLLLALTMKRKEKKKTMLISPTRN-LSLPPGPKSWPLIGNLPEILGRNKPV  63
            T + AF+   L++ L        K M+ +P +  L LPPGP  WP+IG +P +L +++PV
Sbjct  28   TTLQAFVAITLVMLL--------KKMITNPNKKKLYLPPGPIGWPIIGMIPAML-KSRPV  78

Query  64   FRWIHSLMKELNTDIACIRLANTHVIPVTSPRIAREILKKQDSVFATRPLTMGTEYCSRG  123
            FRW+HS+MK+LNT+IAC+RL NT+VI VT P+IARE+LK+QD++FA+RP+T      S G
Sbjct  79   FRWLHSIMKQLNTEIACVRLGNTNVITVTCPKIAREVLKQQDALFASRPMTYAQNVLSNG  138

Query  124  YLTVAVEPQGEQWKKMRRVVASHVTSKKSFQMMLQKRTEEADNLVRYINNRSVKNRGNAF  183
            Y T  + P GEQ+KKMR+VV + +      + + QKR EE D+L  ++ N  VKN G+
Sbjct  139  YKTCVITPFGEQFKKMRKVVMTELVCPARHRWLHQKRAEENDHLTAWVYNM-VKNSGS--  195

Query  184  VVIDLRLAVRQYSGNVARKMMFGIRHFGKGSEDGSGPGLEEIEHVESLFTVLTHLYAFAL  243
              +D R   R Y GN  +K+MFG R F + +    GP  E+ +H++++F  L   +AF +
Sbjct  196  --VDFRFVTRHYCGNAIKKLMFGTRTFSENTAADGGPTAEDSDHMDAMFEALGFTFAFCI  253

Query  244  SDYVPWLRFLDLEGHEKVVSNAMRNVSKYNDPFVDERLMQWRNGKMKEPQDFLDMFIIAK  303
            SDY+P L  LDL GHEK++ ++   + KY+DP +D R+  W+ GK  + +DFLD+FI  K
Sbjct  254  SDYLPMLTGLDLNGHEKIMRDSSAIMDKYHDPIIDGRIKMWKEGKRTQIEDFLDIFISIK  313

Query  304  DTDGKPTLSDEEIKAQVTELMLATVDNPSNAAEWGMAEMINEPSIMQKAVEEIDRVVGKD  363
            D +G P L+ +EIK  + EL++A  DNPSNA EW MAEM+N+P I++KA+EEIDRVVGK+
Sbjct  314  DEEGNPLLTADEIKPTIKELVMAAPDNPSNAVEWAMAEMVNKPEILRKAMEEIDRVVGKE  373

Query  364  RLVIESDLPNLNYVKACVKEAFRLHPVAPFNLPHMSTTDTVVDGYFIPKGSHVLISRMGI  423
            R+V ESD+P LN VKA ++EAFRLHPVA FNLPH++ +DT V GY IPKGS VL+SR G+
Sbjct  374  RIVQESDIPKLNNVKAILREAFRLHPVAAFNLPHVALSDTTVAGYHIPKGSQVLLSRYGL  433

Query  424  GRNPSVWDKPHKFDPERHLSTNTCVDLNESDLNIISFSAGRRGCMGVDIGSAMTYMLLAR  483
            GRNP VW  P  F PERHL+  + V L E+DL  ISFS G+RGC    +G+A+T M+LAR
Sbjct  434  GRNPKVWTDPLSFKPERHLNECSEVTLTENDLRFISFSTGKRGCAAPALGTALTTMMLAR  493
```

```
Query  484  LIQGFTWLPVPGKNKIDISESKNDLFMAKPLYAVATPRLAPHVYPT  529
            L+QGFTW     + ++++ ES +D+F+AKPL  V   RL  H+YPT
Sbjct  494  LLQGFTWKLPENETRVELMESSHDMFLAKPLVMVGELRLPEHLYPT  539


>XP_009142023.1 PREDICTED: dihomomethionine N-hydroxylase-like [Brassica rapa]
Length=529

 Score = 451 bits (1160),  Expect = 3e-154, Method: Compositional matrix adjust.
 Identities = 220/495 (44%), Positives = 316/495 (64%), Gaps = 11/495 (2%)

Query  39   SLPPGPKSWPLIGNLPEILGRNKPVFRWIHSLMKELNTDIACIRLANTHVIPVTSPRIAR  98
             LPPGP  WP IGNL +++ +N+P   WIH +MK++ T+IAC R A  HVI VTS  IAR
Sbjct  37   QLPPGPTRWPFIGNLLQMV-KNRPTHLWIHRVMKDMQTEIACFRFAGVHVITVTSSEIAR  95

Query  99   EILKKQDSVFATRPLTMGTEYCSRGYLTVAVEPQGEQWKKMRRVVASHVTSKKSFQMMLQ  158
            E+L+++D   A R  +  +   S GY  +   P GE WK M++V+ + + S  +   +
Sbjct  96   EVLREKDEALADRADSYSSNLISHGYKDIIFSPYGESWKLMKKVMVTKLMSPSTLNKI--  153

Query  159  KRTEEADNLVRYINNRSVKNRGNAFVVIDLRLAVRQYSGNVARKMMFGIRHFGKGSEDGS  218
             RT EADN+V YI N  +    +   ++++R     YS  V  +MMFG RHF + +EDG
Sbjct  154  DRTLEADNIVTYIFN--LCRLQSTIKLVNVRDVALTYSHAVMMRMMFGQRHFEEPAEDG-  210

Query  219  GPGLEEIEHVESLFTVLTHLYAFALSDYVPWLRFLDLEGHEKVVSNAMRNVSKYNDPFVD  278
            G G +E EH+++++  +   ++  +S+Y+ +LR  +++G E  +  A+  +++ NDP +
Sbjct  211  GLGRKEREHMDAIYQAIDCFFSSNISNYLSFLRGWNIDGEEAKLREAVDIINRCNDPIIH  270

Query  279  ERLMQWRNGKMKEPQ-DFLDMFIIAKDTDGKPTLSDEEIKAQVTELMLATVDNPSNAAEW  337
            ER+  WRN   KE + D+LD  I  KD  G P  + +EI+AQ   + +AT+DN  N  EW
Sbjct  271  ERMHLWRNKSGKETEEDWLDTLITLKDDQGMPLFTLDEIRAQCKNINVATIDNTMNNVEW  330

Query  338  GMAEMINEPSIMQKAVEEIDRVVGKDRLVIESDLPNLNYVKACVKEAFRLHPVAPFNLPH  397
             +AEM+N P IM+KA  E+D +VGKDRLV ESD+P LNY+KAC +E+FRLHP   F  PH
Sbjct  331  TIAEMLNHPEIMEKATNELDMIVGKDRLVQESDIPQLNYIKACSRESFRLHPANAFMPPH  390

Query  398  MSTTDTVVDGYFIPKGSHVLISRMGIGRNPSVWDKPHKFDPERHLSTNT----CVDLNES  453
             +  +T + GYFIPKGS + +SR+G+GRNP +WD+P  F PERHL         V L E
Sbjct  391  GAIENTTLAGYFIPKGSQIFVSRLGLGRNPKIWDEPEAFKPERHLYDRARDPMGVTLMEP  450

Query  454  DLNIISFSAGRRGCMGVDIGSAMTYMLLARLIQGFTWLPVPGKNKIDISESKNDLFMAKP  513
            D+  + FS GRR C G  IG++MT MLLARL+QGF W   PG ++I++  ++++LFMAKP
Sbjct  451  DMRFVIFSTGRRACAGTKIGASMTIMLLARLLQGFEWTRPPGTSQIELVSAESNLFMAKP  510

Query  514  LYAVATPRLAPHVYP  528
            L A   PRLAPH+YP
Sbjct  511  LVASVKPRLAPHLYP  525


>XP_009132654.1 PREDICTED: dihomomethionine N-hydroxylase [Brassica rapa]
Length=530

 Score = 442 bits (1136),  Expect = 1e-150, Method: Compositional matrix adjust.
 Identities = 221/492 (45%), Positives = 319/492 (65%), Gaps = 13/492 (3%)

Query  41   PPGPKSWPLIGNLPEILGRNKPVFRWIHSLMKELNTDIACIRLANTHVIPVTSPRIAREI  100
            PP P   P+IGNL  +L RN+P  +WI  +M ++ TDIAC R    HVI +TS  IARE+
Sbjct  41   PPCPPGIPMIGNLVGML-RNRPTTKWIVRVMNDMKTDIACFRFGRVHVIAITSDEIAREV  99

Query  101  LKKQDSVFATRPLTMGTEYCSRGYLTVAVEPQGEQWKKMRRVVASHVTSKKSFQMMLQKR  160
            +K++DSVFA RP +   EY S GY  V  +  GE+  KM++V+ S + S K+  ++   R
Sbjct  100  VKEKDSVFADRPDSYSAEYISCGYKGVVFDEYGERQMKMKKVMTSELMSTKALDLLRDVR  159

Query  161  TEEADNLVRYINNRSVKNRGNAFVVIDLRLAVRQYSGNVARKMMFGIRHFGKGSEDGS-G  219
              E+DNL+ Y+ N  +  +G    ++++R  V  ++ +V  +++FG +HF + ++DGS G
Sbjct  160  NLESDNLLAYVLN--LYKKGG---LVNVRDIVCTHTHSVKMRLLFGRKHFKETTKDGSLG  214

Query  220  PGLEEIEHVESLFTVLTHLYAFALSDYVPWLRFLDLEGHEKVVSNAMRNVSKYNDPFVDE  279
            P   E EH++++F  L   ++F ++DY    R  +L+G E V+  A+  ++KYN   +DE
Sbjct  215  P--MEKEHLDAIFKALDCFFSFYIADYYSLFRGWNLQGEEVVLREAVDVIAKYNKMIIDE  272
```

```
Query  280   RLMQWR---NGKMKEPQDFLDMFIIAKDTDGKPTLSDEEIKAQVTELMLATVDNPSNAAE  336
             ++  WR   +     P+D+LD+    KD  GKP L+ EEI     +L +  +DN  N  E
Sbjct  273   KIDLWRKNCDANKNVPKDWLDILFTLKDDKGKPLLTPEEITHLSIDLDVVGIDNAVNVIE  332

Query  337   WGMAEMINEPSIMQKAVEEIDRVVGKDRLVIESDLPNLNYVKACVKEAFRLHPVAPFNLP  396
             W +AEM+N+  I+++AVEEIDRVVGKDRLV ESD+P LNYVKAC++E  RLHP  PF +P
Sbjct  333   WTLAEMLNQREILEQAVEEIDRVVGKDRLVQESDVPKLNYVKACIRETLRLHPTNPFLVP  392

Query  397   HMSTTDTVVDGYFIPKGSHVLISRMGIGRNPSVWDKPHKFDPERHLSTNTCVDLNESDLN  456
             HM+  DT + GYF+PKGSH+L+SR GIGRNP  WD+P  + PERHL+ +  V L E D+
Sbjct  393   HMARQDTTLAGYFVPKGSHILVSRPGIGRNPKTWDEPLIYKPERHLTGDE-VMLTEPDMR  451

Query  457   IISFSAGRRGCMGVDIGSAMTYMLLARLIQGFTWLPVPGKNKIDISESKNDLFMAKPLYA  516
             ++SF  GRRGC+G  +G+ M   LL RL+QGF W   P   K+++ ESK +LFMAKPL A
Sbjct  452   LVSFGTGRRGCVGTKLGTYMIVTLLGRLLQGFDWTLPPKTAKVELVESKENLFMAKPLLA  511

Query  517   VATPRLAPHVYP  528
                PRL P++YP
Sbjct  512   CVEPRLDPNMYP  523


>XP_009149073.1 PREDICTED: dihomomethionine N-hydroxylase-like [Brassica rapa]
Length=540

 Score = 400 bits (1028),  Expect = 3e-134, Method: Compositional matrix adjust.
 Identities = 214/532 (40%), Positives = 313/532 (59%), Gaps = 23/532 (4%)

Query  7     MLAFIIGLL---LLALTMKRKEKKKTMLISPTRNLSLPPGPKSWPLIGNLPEILGRNKPV  63
             +L FI+ +    LL   + R  K K       R+  LPPGP  WP++GNLPE++   +P
Sbjct  18    LLVFILSMASISLLGRILSRPTKTKD------RSRQLPPGPPGWPILGNLPELM-TRPR  70

Query  64    FRWIHSLMKELNTDIACIRLANTHVIPVTSPRIAREILKKQDSVFATRPLTMGTEYCSRG  123
              ++I   +K    +IAC   A TH I + S  IARE LK++D+ FA RP
Sbjct  71    HKYIDIALKGQKPEIACFNFAGTHAIVINSDEIAREALKERDADFADRPNLFNMRTIGGN  130

Query  124   YLTVAVEPQGEQWKKMRRVVASHVTSKKSFQMMLQKRTEEADNLVRYINNRSVKNRGNAF  183
             + ++   P GEQ+ KM+RV+ + + S K+  M++  RT EADNL+ Y+   S+  R
Sbjct  131   HKSMGNSPYGEQFMKMKRVITTEIMSVKTLNMLVAARTVEADNLLAYL--LSMYKRSE--  186

Query  184   VVIDLRLAVRQYSGNVARKMMFGIRHFGKGS--EDGSGPGLEEIEHVESLFTVLTHLYAF  241
                D+R   R Y   V  +++FG RH  K +   D    G  E +H++++F  L  L +F
Sbjct  187   -TADVREFSRVYGYAVTMRLLFGRRHITKENVFSDEGRLGQAEKDHLDAIFETLNCLPSF  245

Query  242   ALSDYVP-WLRFLDLEGHEKVVSNAMRNVSKYNDPFVDERLMQWRN-GKMKEPQDFLDMF  299
             + +DY+  W R  +++G E+ V      V  YN+P +DER+  WR  G     +D++D F
Sbjct  246   SPADYLEKWFRGWNIDGQEERVVMYCNKVRSYNNPIIDERVELWREKGGKAAVEDWIDTF  305

Query  300   IIAKDTDGKPTLSDEEIKAQVTELMLATVDNPSNAAEWGMAEMINEPSIMQKAVEEIDRV  359
             I  KD +GK  ++ +E+KAQ  E  +A +DNP+N  EW +AEM+  P I++KA++E+D V
Sbjct  306   ITLKDENGKYYITPDEVKAQCVEFCIAAIDNPANNMEWTLAEMLKNPEILKKALKELDEV  365

Query  360   VGKDRLVIESDLPNLNYVKACVKEAFRLHPVAPFNLPHMSTTDTVVDGYFIPKGSHVLIS  419
             VG+DRLV ESD+PNLNY+KAC +E FR+HP A +   H++  DT + GYFIPKGSH+ +
Sbjct  366   VGRDRLVQESDIPNLNYLKACCRETFRIHPSAHYVPTHVARQDTTLGGYFIPKGSHIHVG  425

Query  420   RMGIGRNPSVWDKPHKFDPERHLSTNTC---VDLNESDLNIISFSAGRRGCMGVDIGSAM  476
             R GIGR+  VW  P  + PERHL  +     + L ES+L  +SF  GRRGC+GV +G+ M
Sbjct  426   RPGIGRSSKVWKDPLVYKPERHLEGDGISKELSLVESELRFVSFGTGRRGCVGVKVGTIM  485

Query  477   TYMLLARLIQGFTWLPVPGKNKIDISESKNDLFMAKPLYAVATPRLAPHVYP  528
               ++LAR +Q F W   PG   + + E  + L MAKPL     PRLAP++YP
Sbjct  486   MVIMLARFLQAFNWKLHPGYGPLSLEED-DALLMAKPLLLSVEPRLAPNLYP  536


>XP_009144065.1 PREDICTED: dihomomethionine N-hydroxylase-like [Brassica rapa]
Length=579

 Score = 395 bits (1015),  Expect = 1e-131, Method: Compositional matrix adjust.
```

```
 Identities = 205/523 (39%), Positives = 297/523 (57%), Gaps = 57/523 (11%)

Query  60   NKPVFRWIHSLMKELNTDIACIRLANTHVIPVTSPRIAREILKKQDSVFATRPLTMGTEY  119
            N+P  +WIH +M+ + T+IAC R A  HVI VTS  IARE+L+ +D   A R      +
Sbjct  56   NRPAHQWIHRVMEAMETEIACFRFAGVHVIVVTSSEIAREVLRAKDKALADRAEAYSIKL  115

Query  120  CSRGYLTVAVEPQGEQWKKMRRVVASHVTSKKSFQMMLQKR-------------------  160
             S GY  V+    GE+WK  ++V+ + + S  +       R
Sbjct  116  ISHGYKGVSFSSYGERWKLAKKVMVTKLLSSATLNKTTSDRLHRLSVVTRRFSFRIEPTI  175

Query  161  ------------------------------TEEADNLVRYINNRSVKNRGNAFVVIDLRL  190
                                          T EADN+V Y+ N  +   G+    +++R
Sbjct  176  SGNADGKEGNAPETHGTRNGTHGDVGKVVITVEADNIVTYVYN--ICQSGSVTKPVNVRD  233

Query  191  AVRQYSGNVARKMMFGIRHFGKGSEDGSGPGLEEIEHVESLFTVLTHLYAFALSDYVPWL  250
                YS  V  +M+FG RHF K ++DG G G +E EH+++++  L  L+ F ++DY+P+L
Sbjct  234  VALTYSHAVMMRMLFGQRHFDKPAKDG-GLGPKEREHMDAIYRALDCLFGFTVADYLPFL  292

Query  251  RFLDLEGHEKVVSNAMRNVSKYNDPFVDERLMQWRNGKMKEPQ-DFLDMFIIAKDTDGKP  309
            R  ++EG EK V  A+  +++ NDP + ER+  WR    KE + D+LD+ I  KD  G
Sbjct  293  RGWNVEGEEKDVREAVDIINRCNDPIICERMHLWREKGGKETEEDWLDILITQKDDQGMY  352

Query  310  TLSDEEIKAQVTELMLATVDNPSNAAEWGMAEMINEPSIMQKAVEEIDRVVGKDRLVIES  369
              + E+I+AQ  ++ +AT+DN  N  EW +AEM+N   I++KA+EE++ +VGKDRL+ ES
Sbjct  353  LFTFEDIRAQCKDVNVATIDNTMNTVEWTIAEMLNHQEILEKAIEELNTIVGKDRLIQES  412

Query  370  DLPNLNYVKACVKEAFRLHPVAPFNLPHMSTTDTVVDGYFIPKGSHVLISRMGIGRNPSV  429
            D+P LNY+KAC KE+FRLHP   F  PH +  DT + GYF+PKGS +L+SR G+GRNP
Sbjct  413  DIPQLNYIKACCKESFRLHPPNAFLPPHGAREDTTLAGYFVPKGSQILVSRPGLGRNPKT  472

Query  430  WDKPHKFDPERHLSTNT----CVDLNESDLNIISFSAGRRGCMGVDIGSAMTYMLLARLI  485
            W++P  F PERHL  +      V L E D+  + F  GRRGC G  +G+ M  MLLARL+
Sbjct  473  WEEPDAFKPERHLVDHARNPVDVTLMEPDMRFVVFGTGRRGCAGPKLGATMIVMLLARLL  532

Query  486  QGFTWLPVPGKNKIDISESKNDLFMAKPLYAVATPRLAPHVYP  528
            QGF W    G +++++  +  +LFMAKPL A A PRLAP +YP
Sbjct  533  QGFEWTLQAGASQVELIAANTNLFMAKPLLASAKPRLAPGLYP  575


>XP_009119683.1 PREDICTED: cytochrome P450 703A2 [Brassica rapa]
Length=522

 Score = 301 bits (772),  Expect = 4e-96, Method: Compositional matrix adjust.
 Identities = 180/499 (36%), Positives = 267/499 (54%), Gaps = 29/499 (6%)

Query  40   LPPGPKSWPLIGNLPEILGRNKPVFRWIHSLMKELNTD---IACIRLANTHVIPVTSPRI  96
            LPPGP  WP++GNL ++     P+    H  M  L +    +  +RL N   I    P
Sbjct  33   LPPGPPRWPILGNLLQL----GPL---PHRDMAALCSKYGPLVYLRLGNIDAITTNDPET  85

Query  97   AREILKKQDSVFATRPLTMGTEYCSRGYLTVAVEPQGEQWKKMRRVVASHVTSKKSFQMM  156
             REIL +QD VFA+RP T+   + + G   VA+ P G  WK+MRR+   H+ + K  +
Sbjct  86   IREILFRQDDVFASRPKTLAAVHLAYGCGDVALAPMGPHWKRMRRICMEHLLTTKRLESF  145

Query  157  LQKRTEEADNLVRYINNRSVKNRGNAFVVIDLRLAVRQYSGNVARKMMFGIRHFGKGSED  216
              +R EEA  L++       V  R       I+LR  +  +S N   +M+ G + FG GS
Sbjct  146  TSQRAEEAQYLIQ-----DVCKRAECGKPINLREVLGAFSMNNVTRMLLGKQFFGPGSVV  200

Query  217  GSGPGLEEIEHVESLFTVLTHLYAFALSDYVPWLRFLDLEGHEKVVSNAMRNVSKYNDPF  276
            G+    E +     LF +L  +Y   L DY+P+ R++D  G EK + +  + V K++
Sbjct  201  GAKEAQEFMHITHKLFRLLGVIY---LGDYLPFWRWVDPYGCEKEMRDVEKRVDKFHTKI  257

Query  277  VDERLMQWRNGKMKEPQ---DFLDMFIIAKDTDGKPTLSDEEIKAQVTELMLATVDNPSN  333
            ++E     R  + K  +   DF+D+ +     +GK  + D EIKA + +++ A  D  +
Sbjct  258  IEEHRRAKREKEDKNIEGDMDFVDVLLSLPGENGKEHMDDVEIKALIQDMIAAATDTSAV  317

Query  334  AAEWGMAEMINEPSIMQKAVEEIDRVVGKDRLVIESDLPNLNYVKACVKEAFRLHPVAPF  393
              EW MAE+I +P +M+K  EE+D VVG +R+V E+DL +LNY++  V+E FR+HP  PF
Sbjct  318  TNEWAMAEVIKQPRVMRKIQEELDNVVGSNRMVNETDLVHLNYLRCVVRETFRMHPAGPF  377
```

```
Query  394   NLPHMSTTDTVVDGYFIPKGSHVLISRMGIGRNPSVWDKP-HKFDPERHLSTNTC--VDL  450
              +PH S   T ++GY+IP  + V I+  G+GRN SVW     +F PERH   +    V++
Sbjct  378   LIPHESVRPTTINGYYIPAKTRVFINTHGLGRNTSVWTTDIEEFRPERHWPVDGSGRVEI  437

Query  451   NES-DLNIISFSAGRRGCMGVDIGSAMTYMLLARLIQGFTWLPVPGKNKIDISESKN-DL  508
             +   D  I+ FSAG+R C G  +G  M  M LARL   F W        ID  E     +
Sbjct  438   SHGPDYKILPFSAGKRKCPGAPLGVTMVLMALARLFHCFDWT---TPEDIDTVEVYGMTM  494

Query  509   FMAKPLYAVATPRLAPHVY  527
               AKPL+A+A PRLA H+Y
Sbjct  495   PKAKPLWALAKPRLAAHLY  513


>XP_009118885.1 PREDICTED: LOW QUALITY PROTEIN: cytochrome P450 703A2-like
[Brassica
rapa]
Length=508

 Score = 289 bits (740),  Expect = 2e-91, Method: Compositional matrix adjust.
 Identities = 181/529 (34%), Positives = 271/529 (51%), Gaps = 44/529 (8%)

Query  7     MLAF-IIGLLLLALTMKRKEKKKTMLISPTRNLSLPPGPKSWPLIGNLPEILGRNKPVFR  65
             +LAF +  +L L + + R  K     +S  +    PPGP  WP++GNL ++     P+
Sbjct  4     LLAFSLFAILSLNVLLWRWLK-----VSACKTQKFPPGPPRWPILGNLLQL----GPL--  52

Query  66    WIHSLMKELNTDIACIRLANTHVIPVTSPRIAREILKKQDSVFATRPLTMGTEYCSRGYL  125
               H  M  L           N   I    P   REIL +QD VF++RP T+   + + G
Sbjct  53    -PHRDMARLCDKYG---XGNVDAITTNDPDTIREILFRQDDVFSSRPKTLAAVHLAYGCG  108

Query  126   TVAVEPQGEQWKKMRRVVASHVTSKKSFQMMLQKRTEEADNLVRYINNRSVKNRGNAFVV  185
              VA+ P G  WK+MRR+   H+ + K  +    +R EE+  L+        V  R     +
Sbjct  109   DVALAPMGPHWKRMRRICMEHLLTTKRLESFTAQRAEESCYLIA-----DVYKRAECGKL  163

Query  186   IDLRLAVRQYSGNVARKMMFGIRHFGKGSEDGSGPGLEEIEHVESLFTVLTHLYAFALSD  245
             ++LR  +  +S N   +M+ G + FG GS  G     E +     LF +L  +Y   L D
Sbjct  164   VNLREVLGAFSMNNVTRMLLGKQFFGPGSVVGPKEAQEFMHITHKLFRLLGVIY---LGD  220

Query  246   YVPWLRFLDLEGHEKVVSNAMRNVSKYNDPFVDERLMQWR---------NGKMKEPQDFL  296
             Y+P+ R++D  G EK + +  + V K++   +DE     R          NG+M    DF+
Sbjct  221   YLPFWRWVDPYGCEKEMKDVEKRVDKFHTKIMDEHRRAKREREDNTKNNNGEM----DFV  276

Query  297   DMFIIAKDTDGKPTLSDEEIKAQVTELMLATVDNPSNAAEWGMAEMINEPSIMQKAVEEI  356
             D+ +     +GK  + D EIKA + +++ A  D  +   EW MAE+I +P +M+K  EE+
Sbjct  277   DVLLSLPGENGKEHMDDVEIKALIQDMIAAATDTSAVTNEWAMAEVIKQPRVMRKIQEEL  336

Query  357   DRVVGKDRLVIESDLPNLNYVKACVKEAFRLHPVAPFNLPHMSTTDTVVDGYFIPKGSHV  416
             D +VG  R+V ESDL +LNY++  V+E FR+HPV PF +PH      T V+GY+IP  + V
Sbjct  337   DSIVGSKRMVDESDLVHLNYLRCVVRETFRMHPVGPFLIPHEFVRPTTVNGYYIPAKTRV  396

Query  417   LISRMGIGRNPSVWDKPHKFDPERHLSTNTC--VDLNE-SDLNIISFSAGRRGCMGVDIG  473
              I+  G+GRN  +WD   +F PERH   +    V+++  +D  I+ FSAG+R C G  +G
Sbjct  397   FINTHGLGRNTKIWDDTDEFRPERHWPVDGSGRVEISHGADYKILPFSAGKRKCPGAPLG  456

Query  474   SAMTYMLLARLIQGFTWLPVPGKNKIDISESKN-DLFMAKPLYAVATPR  521
               M  M LARL   F W        ID  E     +  AKPL+A+A PR
Sbjct  457   VTMVLMALARLFHCFDWT---SPENIDTLEVYGMTMPKAKPLWALAKPR  502


>XP_009122411.1 PREDICTED: flavonoid 3'-monooxygenase [Brassica rapa]
Length=511

 Score = 263 bits (672),  Expect = 2e-81, Method: Compositional matrix adjust.
 Identities = 170/510 (33%), Positives = 254/510 (50%), Gaps = 34/510 (7%)

Query  30    MLISPTRNLSLPPGPKSWPLIGNLPEILGRNKPVFRWIHSLMKELNTDIACIRLANTHVI  89
             +++S  RN  LPPGP  WP+IGNLP +     P      + M      I  +RL    V+
Sbjct  19    LVLSRRRNNRLPPGPNPWPIIGNLPHM----GPKPHQTLAAMVTTYGPILHLRLGFADVV  74

Query  90    PVTSPRIAREILKKQDSVFATRPLTMGTEYCSRGYLTVAVEPQGEQWKKMRRVVASHVTS  149
```

```
               S  +A + LK  D+ FA+RP   G ++ +  Y  +   P G++W+ +R++ + H+ S
Sbjct  75   VAASKSVAEQFLKVHDANFASRPPNSGAKHMAYNYQDLVFAPYGQRWMLRKISSVHLFS  134

Query  150  KKSFQMMLQKRTEEADNLVRYINNRSVKNRGNAFVVIDLRLAVRQYSGNVARKMMFGIRH  209
             K+ +     R EE   L+R +   + K        ++L   V     N   + M G R
Sbjct  135  AKALEDFKHVRQEEVGTLMRELARANTKP-------VNLGQLVNMCVLNALGREMIGRRL  187

Query  210  FGKGSEDGSGPGLEEIEHVESLFTVLTHLYA-FALSDYVPWLRFLDLEGHEKVVSNAMRN  268
            FG  ++         + E   S+ T +  L   F + D+VP L  LDL+G    V+  M+
Sbjct  188  FGADAD-------HKAEEFRSMVTEMMALAGVFNIGDFVPALDCLDLQG----VAGKMKR  236

Query  269  VSKYNDPFVDERLMQ---WRNGKMKEPQDFLDMFIIAKDTD---GKPTLSDEEIKAQVTE  322
            + K  D F+   L +    +NG+ ++  D L   I  K TD      TL+D EIKA +
Sbjct  237  LHKRFDAFLSSILEEHEAMKNGQDQKHTDMLSTLISLKGTDFDGEGGTLTDTEIKALLLN  296

Query  323  LMLATVDNPSNAAEWGMAEMINEPSIMQKAVEEIDRVVGKDRLVIESDLPNLNYVKACVK  382
            +  A  D  ++  +W +AE+I  P IM+KA EE+D VVG+ R + ESDL  L Y++A +K
Sbjct  297  MFTAGTDTSASTVDWAIAELIRHPEIMRKAQEELDSVVGRGRPINESDLSQLPYLQAVIK  356

Query  383  EAFRLHPVAPFNLPHMSTTDTVVDGYFIPKGSHVLISRMGIGRNPSVWDKPHKFDPERHL  442
            E FRLHP  P +LPH+++    ++GY IPKGS +L +   I R+P  W  P  F PER L
Sbjct  357  ENFRLHPPTPLSLPHIASESCEINGYHIPKGSTLLTNIWAIARDPDQWSDPLTFRPERFL  416

Query  443  --STNTCVDLNESDLNIISFSAGRRGCMGVDIGSAMTYMLLARLIQGFTWLPVPG--KNK  498
                   VD+  +D  +I F AGRR C G+ +G     +L A L+ GF W    G    K
Sbjct  417  PGGEKAGVDVKGNDFELIPFGAGRRICAGLSLGLRTIQLLTATLVHGFEWELAGGVTPEK  476

Query  499  IDISESKN-DLFMAKPLYAVATPRLAPHVY  527
            +++ E+    L  A PL     PRL    Y
Sbjct  477  LNMEETYGITLQRAVPLVVHPKPRLDRSAY  506


>XP_009141948.1 PREDICTED: LOW QUALITY PROTEIN: cytochrome P450 98A3-like
[Brassica
rapa]
Length=508

 Score = 229 bits (584),  Expect = 1e-68, Method: Compositional matrix adjust.
 Identities = 159/503 (32%), Positives = 247/503 (49%), Gaps = 39/503 (8%)

Query  38   LSLPPGPKSWPLIGNLPEILGRNKPV-FRWIHSLMKELNTDIACIRLANTHVIPVTSPRI  96
               PPGP+  P++GNL +I    KPV FR  +   +     I  + + +   + V+S  +
Sbjct  25   FKFPPGPRPKPIVGNLNDI----KPVRFRCYYEWAQTYGP-IISVWIGSILNVVVSSAEL  79

Query  97   AREILKKQDSVFATRPLTMGTEYCSRGYLTVAVEPQGEQWKKMRRVVASHVTSKKSFQMM  156
            A+ +LK+ D   A R     TE  SR    +     G  + K+R+V    + + K  + +
Sbjct  80   AKVVLKEHDQKLADRHRNRSTEAFSRNGQDLIWADYGAHYVKVRKVCTXELFTPKRLESL  139

Query  157  LQKRTEEADNLV----RYINNRSVKNRGNAFVVIDLRLAVRQYSGNVA----RKMMFGIR  208
               R +E   +V    R  N    K +G         L +R+Y G VA     ++ FG R
Sbjct  140  RPIREDEVTAMVESVFRDCNVPENKTKG---------LQLRKYLGAVAFNNITRLAFGKR  190

Query  209  HF-GKGSEDGSGPGLEEIEHVESLFTVLTHLYAFALSDYVPWLRFLDLEGHEKVVSNAMR  267
                +G  D  G     +E    +   L    + ++++++PWLR+L     EK  +
Sbjct  191  FVNAEGVMDEQG-----LEFKAIVSNGLKLGASLSIAEHIPWLRWL-FPADEKAFAKHGA  244

Query  268  NVSKYNDPFVDERLMQWRNGKMKEPQDFLDMFIIAKDTDGKPTLSDEEIKAQVTELMLAT  327
                    ++E  +  R       Q F+D  +  KD   +  LS++ I   + +++ A
Sbjct  245  RRDLLTRAIMEEHTLA-RQKSSGAKQHFVDALLTLKD---QYDLSEDTIIGLLWDMITAG  300

Query  328  VDNPSNAAEWGMAEMINEPSIMQKAVEEIDRVVGKDRLVIESDLPNLNYVKACVKEAFRL  387
            +D  +  AEWGMAEMI  P + QK  EE DRV+G+DR++ E D   L Y++  VKE+FRL
Sbjct  301  MDTTAITAEWGMAEMIKNPRVQQKVQEEFDRVIGRDRVLTEPDFSRLPYLQCVVKESFRL  360

Query  388  HPVAPFNLPHMSTTDTVVDGYFIPKGSHVLISRMGIGRNPSVWDKPHKFDPERHLSTNTC  447
            HP  P  LPH S  D  + GY IPKGS+V ++   + R+PSVW+ P +F PER L  +
Sbjct  361  HPPTPLMLPHRSNADVKIGGYDIPKGSNVHVNVWAVARDPSVWENPLEFRPERFLEED--  418

Query  448  VDLNESDLNIISFSAGRRGCMGVDIGSAMTYMLLARLIQGFTWLPVPGK--NKIDISESK  505
```

```
              VD+   D  ++ F AGRR C G  +G  +   ++  L+  F W P  G    +ID+SE+
Sbjct  419  VDMKGHDFRLLPFGAGRRVCPGAQLGINLVTSMMGHLLHHFVWTPPQGTKPEEIDMSENP  478

Query  506  NDL-FMAKPLYAVATPRLAPHVY  527
              + +M  P+ AVATPRL   +Y
Sbjct  479  GLVTYMRVPVQAVATPRLPSELY  501


>XP_009136876.1 PREDICTED: cytochrome P450 81D11-like [Brassica rapa]
Length=503

 Score = 223 bits (569),  Expect = 2e-66, Method: Compositional matrix adjust.
 Identities = 160/521 (31%), Positives = 252/521 (48%), Gaps = 41/521 (8%)

Query  5    TPMLAFIIGLLLLALTMKRKEKKKTMLISPTRNLSLPPGPK-SWPLIGNLPEILGRNKPV  63
            T  L F++  L+  +   R +K+K           LPP P  + P+IG+L  +     P+
Sbjct  8    TFTLIFVVLTLIFFIVTNRTKKRKP---------KLPPSPPFALPVIGHLRLL---KPPL  55

Query  64   FRWIHSLMKELN-TDIACIRLANTHVIPVTSPRIAREILKKQDSVFATRPLTMGTEYCSR  122
             R  +S+ + L    I  +RL +  V  V+S  IA E   K D V A RP T+ ++Y S
Sbjct  56   HRVFYSISQSLGGAPIFSLRLGSRLVFVVSSHSIAEECFTKNDVVLANRPNTIASKYVSY  115

Query  123  GYLTVAVEPQGEQWKKMRRVVASHVTSKKSFQMMLQKRTEEADNLVRYINNRSVKNRGNA  182
             + T+   P GE W+ +RR+ A  + S       L  R +E    VR +  R  +N
Sbjct  116  DHTTMVTAPYGEHWRNLRRIGAVEIFSAHRLNKFLSIRQDE----VRRLIVRLSRNSSYE  171

Query  183  FVVIDLRLAVRQYSGNVARKMMFGIRHFGKGSEDGSGPGLEEIEHVESLFTVLTHLY-AF  241
            F  +++       + N   +M+ G R++G  SE+ S     E + V  L   L  ++ A
Sbjct  172  FAKVEINSMFSDLTFNNIIRMVAGKRYYGDVSEENS-----EAKLVRQLIADLMSIFGAG  226

Query  242  ALSDYVPWLRFLDLEGHEKVVSNAMRNVSKYNDPFVDERLMQWRNGKMKEPQDFLDMFII  301
              +DYVP LR++   G EK V         ++    VDER    R  K K     +D  +
Sbjct  227  NAADYVPILRWVT--GFEKRVKELGGRFDEFLQGLVDER----RAAKEK-GNTMIDHLLS  279

Query  302  AKDTDGKPTLSDEEIKAQVTELMLATVDNPSNAAEWGMAEMINEPSIMQKAVEEIDRVVG  361
             ++T      +D  IK  +  L+LA  D  +   EW ++ ++N P  ++KA EEID +G
Sbjct  280  LQETQ-PGYYTDRTIKGTILSLILAGTDTSAVTLEWALSSLLNHPEKLRKAREEIDCKIG  338

Query  362  KDRLVIESDLPNLNYVKACVKEAFRLHPVAPFNLPHMSTTDTVVDGYFIPKGSHVLISRM  421
             DRLV ESD+ NL  ++  V E  RL+P  P  +PH+++ D  V GY +P+G+ +L++
Sbjct  339  LDRLVEESDISNLPCLQNIVSETLRLYPAGPLMVPHVASEDCKVGGYDMPQGTTLLVNLW  398

Query  422  GIGRNPSVWDKPHKFDPERHLSTNTCVDLNESDLNIISFSAGRRGCMGVDIGSAMTYMLL  481
             + R+P +WD P  F PER          L       ++F  GRR C G  +   +  + L
Sbjct  399  AMHRDPQLWDDPETFKPERFEKEGEAHKL-------MTFGLGRRACPGSGLAQRLVSLTL  451

Query  482  ARLIQGFTWLPVPGKNKIDISESKNDLF-MAKPLYAVATPR  521
            A LIQ F W  + G+ ++D++E+       A+PL A+ T R
Sbjct  452  ASLIQCFEWERI-GEEEVDMTEAGGATMPKARPLVAMCTAR  491


>XP_009133464.1 PREDICTED: cytochrome P450 98A3 [Brassica rapa]
Length=508

 Score = 223 bits (569),  Expect = 2e-66, Method: Compositional matrix adjust.
 Identities = 157/501 (31%), Positives = 247/501 (49%), Gaps = 35/501 (7%)

Query  38   LSLPPGPKSWPLIGNLPEILGRNKPV-FRWIHSLMKELNTDIACIRLANTHVIPVTSPRI  96
              LPPGP+  P++GNL +I    KPV FR  +   +     I  + + +   + V+S  +
Sbjct  25   FKLPPGPRPKPIVGNLYDI----KPVRFRCYYEWAQTYGP-IISVWIGSILNVVVSSAEL  79

Query  97   AREILKKQDSVFATRPLTMGTEYCSRGYLTVAVEPQGEQWKKMRRVVASHVTSKKSFQMM  156
            A+E+LK+ D   A R     TE  SR    +     G  + K+R+V    + + K  + +
Sbjct  80   AKEVLKEHDQKLADRHRNRSTEAFSRNGQDLIWADYGPHYVKVRKVCTLELFTPKRLESL  139

Query  157  LQKRTEEADNLVRYI--NNRSVKNRGNAFVVIDLRLAVRQYSGNVA----RKMMFGIRHF  210
               R +E   +V  +  +    +NR           L +R+Y G VA     ++ FG R
Sbjct  140  RPIREDEVTAMVESVFRDCNLPENRVKG-------LQLRKYLGAVALNNITRLAFGKRFM  192
```

```
Query  211  -GKGSEDGSGPGLEEIEHVESLFTVLTHLYAFALSDYVPWLRFLDLEGHEKVVSNAMRNV  269
              +G  D  G     +E    +   L    + ++++++PWLR++     EK  +
Sbjct  193  NAEGVMDEQG-----LEFKAIVSNGLNLGASLSIAEHIPWLRWM-FPADEKAFAKHGARR  246

Query  270  SKYNDPFVDERLMQWRNGKMKEPQDFLDMFIIAKDTDGKPTLSDEEIKAQVTELMLATVD  329
                   ++E  +  R       Q F+D  +   KD   +  LS++ I   + +++ A  +D
Sbjct  247  DILTRAIMEEHTLA-RQKSSGAKQHFVDALLTLKD---QYDLSEDTIIGLLWDMITAGMD  302

Query  330  NPSNAAEWGMAEMINEPSIMQKAVEEIDRVVGKDRLVIESDLPNLNYVKACVKEAFRLHP  389
              +  AEW MAEMI  P + QK  EE DRVVG DR+V E D   L Y++  VKE+FRLHP
Sbjct  303  TTAITAEWAMAEMIKNPRVQQKVQEEFDRVVGLDRVVTEPDFSRLPYLQCVVKESFRLHP  362

Query  390  VAPFNLPHMSTTDTVVDGYFIPKGSHVLISRMGIGRNPSVWDKPHKFDPERHLSTNTCVD  449
              P  LPH S     + GY IPKGS+V ++   + R+P+VW  P +F PER L  +  VD
Sbjct  363  PTPLMLPHRSNAHVKIGGYDIPKGSNVHVNVWAVARDPAVWKNPLEFRPERFLEED--VD  420

Query  450  LNESDLNIISFSAGRRGCMGVDIGSAMTYMLLARLIQGFTWLPVPGK--NKIDISESKND  507
            +   D  ++ F AGRR C G  +G  +   +++ L+  F W P  G   ++ID+SE+
Sbjct  421  MKGHDFRLLPFGAGRRVCPGAQLGINLVTSMMSHLLHHFVWTPPQGTKLDEIDMSENPGL  480

Query  508  L-FMAKPLYAVATPRLAPHVY  527
            + +M  P+ AVATPRL   +Y
Sbjct  481  VTYMRVPVQAVATPRLPSDLY  501


>XP_009101692.1 PREDICTED: LOW QUALITY PROTEIN: cytochrome P450 93A3 [Brassica
rapa]
Length=518

 Score = 223 bits (567),  Expect = 6e-66, Method: Compositional matrix adjust.
 Identities = 157/487 (32%), Positives = 251/487 (52%), Gaps = 32/487 (7%)

Query  49   LIGNLPEILGRNKP-VFRWIHSLMKELNTDIACIRLANTHVIPVTSPRIAREILKKQDSV  107
            +IGNLP +    KP +  +   L KE +  I  + L +   I V+S  +AREIL+  D +
Sbjct  55   IIGNLPFL----KPELHTYFQGLAKE-HGPIFKLWLGSKLAIVVSSSEVAREILRTNDVI  109

Query  108  FATRPLTMGTEYCSRGYLTVAVEPQGEQWKKMRRVVASHVTSKKSFQMMLQKRTEEADNL  167
            FA   +       + G + +A  P G +W+ +R++  + + S       +  R  E
Sbjct  110  FANHDVPAVALINTYGGIDIAWSPYGPRWRMLRKLCVNKILSNVRLDSSVGLRRGETRRT  169

Query  168  VRYINNR----SVKNRGNAFVVIDLRLAVRQYSGNVARKMMFGIRHFGKGSEDGSGPGLE  223
            VRY+ ++    S  N G    V+ L         NV  +M++G        E+    G E
Sbjct  170  VRYLADQARAGSQLNLGEQIFVMIL---------NVVTQMLWGAT---VEEEEREIVGAE  217

Query  224  EIEHVESLFTVLTHLYAFALSDYVPWLRFLDLEGHEKVVSNAMRNVSKYNDPFVDERLMQ  283
             IE V+ +  +L       +SD+ P L   DL+G  K +    + + +  D  +++RL
Sbjct  218  FIELVQEMNDLLM---VPNISDFFPALNRFDLQGLAKRMRGLAQRLDRLFDRVINQRL-G  273

Query  284  WRNGKMKEPQDFLDMFIIAKDT-DGKPTLSDEEIKAQVTELMLATVDNPSNAAEWGMAEM  342
               G   + +DFL++ +  KD  DG+  L+  ++KA +  ++L   D+  +  E+ MAE+
Sbjct  274  VDKGSEGKGEDFLEVLLKIKDEEDGQTNLNMNDVKALLMNMVLGGTDSSLHVIEFAMAEL  333

Query  343  INEPSIMQKAVEEIDRVVGKDRLVIESDLPNLNYVKACVKEAFRLHPVAPFNLPHMSTTD  402
            IN+P IM++A +E+D VVGKD++V ES +P L Y+ A +KE  RLH VAP  +PH  +
Sbjct  334  INKPDIMKRAQQELDEVVGKDKIVEESHIPXLPYILAIMKETLRLHMVAPLLIPHRPSQT  393

Query  403  TVVDGYFIPKGSHVLISRMGIGRNPSVWDKPHKFDPERHLSTNTCVDLNESDLNIISFSA  462
            TVV G+ IPK S V I+   I RNP+VW+ P +FDP R L  +   D N +D N I F A
Sbjct  394  TVVGGFTIPKDSKVFINVWAIHRNPNVWENPLEFDPNRFL--DKSYDFNGNDFNYIPFGA  451

Query  463  GRRGCMGVDIGSAMTYMLLARLIQGFTWLPVPGKNKIDISESKN-DLFMAKPLYAVATPR  521
            GRR C+G+ +G  +    +A L+  F W  +P   ++++ E     L +  PL      R
Sbjct  452  GRRICVGMAMGERIVLYNIATLLHSFDW-KLPRGERMEVEEKFGIALKLKNPLLTTPVLR  510

Query  522  LA-PHVY  527
            L+ P++Y
Sbjct  511  LSDPNLY  517
```

```
>XP_009138414.1 PREDICTED: cytochrome P450 84A1-like [Brassica rapa]
Length=519

 Score = 222 bits (566),  Expect = 7e-66, Method: Compositional matrix adjust.
 Identities = 150/543 (28%), Positives = 261/543 (48%), Gaps = 60/543 (11%)

Query  1    MLDSTPMLAFIIGLLLLALTMKRKEKKKTMLISPTRNLSLPPGPKSWPLIGNLPEILGRN  60
            +LD T  +  I+ L +    + R+ +            S PPGP+ WP+IGN+   L  +
Sbjct  12   VLDPTTAILIIVSLFIFIGVITRRRR------------SYPPGPRGWPIIGNM---LMMD  56

Query  61   KPVFRWIHSLMKELNTDIACIRLANTHVIPVTSPRIAREILKKQDSVFATRPLTMGTEYC  120
            +   R + +L K+    +  +R+   H+  V+SP +AR++L+ QDS+F+ RP T+   Y
Sbjct  57   QLTHRGLANLAKKYG-GLCHLRMGFLHMYAVSSPDVARQVLQVQDSIFSNRPATIAISYL  115

Query  121  SRGYLTVAVEPQGEQWKKMRRVVASHVTSKKSFQMMLQKRTEEADNLVRYINNRSVKNRG  180
            +     +A    G  W++MR+V    V S+K  +     R +E D ++R +++    N G
Sbjct  116  TYDRADMAFAHYGPFWRQMRKVCVMKVFSRKRAESWASVR-DEVDKMIRSVSS----NVG  170

Query  181  NAFVVIDLRLAVRQYSGNVARKMMFGIRHFGKGSEDGSGPGLEEIEHVESLFTVLTHLYA  240
             +  V +   A+ +   N+  +  FG        E G    +  ++    LF       A
Sbjct  171  KSINVGEQIFALTR---NITYRAAFG-----SACEKGQDEFIRILQEFSKLFG------A  216

Query  241  FALSDYVPWLRFLDLEGHEKVVSNAMRNVSKYNDPFVDERLMQWRNGKMKEP--------  292
            F ++D++P+  ++D +G  K +  A  ++  + D  +DE + +  N    +
Sbjct  217  FNVADFIPYFGWIDPQGISKRLVKARNDLDGFIDDIIDEHMKKKENQNTVDDGDVGDTDM  276

Query  293  -QDFLDMF-----IIAKDTDGKPT--LSDEEIKAQVTELMLATVDNPSNAAEWGMAEMIN  344
              D L  +     ++++  D + +  L+ + IKA + ++M    +  ++A EW + E++
Sbjct  277  VDDLLAFYSEEAKLVSETMDLQNSIKLTRDNIKAIIMDVMFGGTETVASAIEWALTELLR  336

Query  345  EPSIMQKAVEEIDRVVGKDRLVIESDLPNLNYVKACVKEAFRLHPVAPFNLPHMSTTDTV  404
             P  +++  +E+  VVG DR V ESD+  L ++K  +KE  RLHP  P  L H +  DT
Sbjct  337  SPEDLKRVQQELAEVVGLDRRVEESDIEKLTFLKCTLKETLRLHPPIPL-LLHETAEDTE  395

Query  405  VDGYFIPKGSHVLISRMGIGRNPSVWDKPHKFDPERHLSTNTCVDLNESDLNIISFSAGR  464
            +DGYF+PK S V+I+   IGR+P  W     F P R L      D   S+   I F +GR
Sbjct  396  IDGYFVPKKSRVMINAFAIGRDPKSWPDAETFRPSRFLEPGVA-DFKGSNFEFIPFGSGR  454

Query  465  RGCMGVDIGSAMTYMLLARLIQGFTWLPVPGKNKIDISESKNDLF-----MAKPLYAVAT  519
            R C G+ +G     + +A ++  FTW    G    ++    ND+F      A  L+AV +
Sbjct  455  RSCPGMQLGLYALELAVAHILHCFTWKLPDGMKASEL--DMNDVFGLTAPKATRLFAVPS  512

Query  520  PRL  522
             RL
Sbjct  513  TRL  515


>XP_009109920.1 PREDICTED: cytochrome P450 82C4 [Brassica rapa]
Length=525

 Score = 219 bits (557),  Expect = 2e-64, Method: Compositional matrix adjust.
 Identities = 135/494 (27%), Positives = 243/494 (49%), Gaps = 11/494 (2%)

Query  41   PPGPKSWPLIGNLPEILGRNKPVFRWIHSLMKELNTDIACIRLANTHVIPVTSPRIAREI  100
            P    +WP+IG+L  + G+ + ++R +  +       ++ +RL +     V+S  +A+E
Sbjct  33   PEPSGAWPIIGHLHLLGGKEQLLYRTLGEMANRYGPAMS-LRLGSNEAFVVSSFEVAKEC  91

Query  101  LKKQDSVFATRPLTMGTEYCSRGYLTVAVEPQGEQWKKMRRVVASHVTSKKSFQMMLQKR  160
                D   A+RP+T   ++    Y      P    W++MR++    + S +  QM+   R
Sbjct  92   FTVNDKALASRPMTAAAKHMGYNYAVFGFAPYSSFWREMRKIATVELLSNRRLQMLKHVR  151

Query  161  TEEADNLVRYINNRSVKNRGNAFVVIDLRLAVRQYSGNVARKMMFGIRHFGKGSEDGSGP  220
              E    V+ + +  VK  G+  +++DL+  +   + N+  +M+ G R+FG GS   +
Sbjct  152  VSEISMGVKDLYSLWVKKGGSEPIIVDLKRWLEDMTLNMIVRMVAGKRYFGGGST--TPE  209

Query  221  GLEEIEHVESLFTVLTHLYA-FALSDYVPWLRFLDLEGHEKVVSNAMRNVSKYNDPFVDE  279
              EE    +   T   HL   F +SD  P L + DL+GHEK +      +    + +++
Sbjct  210  DTEEARQCQKAITKFFHLIGIFTMSDAFPTLGWFDLQGHEKEMKKTGSELDVILERWIEN  269
```

```
Query  280  RLMQWR-NGKMKEPQDFLDMFIIAKDTDGKPTL---SDEEIKAQVTELMLATVDNPSNAA  335
               Q + +G  +   DF+D+ +   +      L   ++  IK+    L+L   D  ++
Sbjct  270  HRQQRKVSGNKENDSDFIDVMLSLAEQGKLSHLQYDANTSIKSTCLALILGGSDTTASTL  329

Query  336  EWGMAEMINEPSIMQKAVEEIDRVVGKDRLVIESDLPNLNYVKACVKEAFRLHPVAPFNL  395
             W +A ++N   +++KA +EID  VG DR V +SD+ NL Y++A +KE  RL+P  P
Sbjct  330  TWAIALLLNNKDMLKKAQDEIDLQVGTDRNVEDSDIENLVYLQAIIKETLRLYPAGPLLG  389

Query  396  PHMSTTDTVVDGYFIPKGSHVLISRMGIGRNPSVWDKPHKFDPERHLSTNTC-VDLNESD  454
            P  +  D  V GY +  G+ ++++   I R+P VW +P++F PER L+      D+   +
Sbjct  390  PREAMEDCTVAGYHVACGTRLIVNVWKIQRDPKVWVEPNEFRPERFLTGEAKEFDVRGQN  449

Query  455  LNIISFSAGRRGCMGVDIGSAMTYMLLARLIQGFTWLPVPGKNKIDISESKN-DLFMAKP  513
              +I F +GRR C G  +   + ++ LAR +  F  +       +D+SES    +  A P
Sbjct  450  FELIPFGSGRRSCPGSSLAMQVLHLGLARFLHSFD-VKTDLDVAVDMSESPGLTIPKATP  508

Query  514  LYAVATPRLAPHVY  527
            L  + +PRL  H++
Sbjct  509  LEVMISPRLEKHLH  522


>XP_009142348.1 PREDICTED: cytochrome P450 76C4-like [Brassica rapa]
Length=513

 Score = 216 bits (550),  Expect = 1e-63, Method: Compositional matrix adjust.
 Identities = 143/520 (28%), Positives = 260/520 (50%), Gaps = 36/520 (7%)

Query  10   FIIGLLLLA----LTMKRKEKKKTMLISPTRNLSLPPGPKSWPLIGNLPEILGRNKPVFR  65
            F++G  +L+    ++  R  ++K++  + T     PPGP    +IGN+  ++G++    R
Sbjct  10   FLLGCFILSCFFIISTTRSRRRKSLTAAAT-----PPGPPRQLIIGNI-NLVGKDP--HR  61

Query  66   WIHSLMKELNTDIACIRLANTHVIPVTSPRIAREILKKQDSVFATRPLTMGTEYCSRGYL  125
                L K     +  ++L   + + +TSP  ARE+L+  D V + R         +
Sbjct  62   SFADLSKTYGP-VMSLKLGFLNTVVITSPEAAREVLRTHDQVLSNRGSNNSINSINHQEF  120

Query  126  TVAVEPQGE-QWKKMRRVVASHVTSKKSFQMMLQKRTEEADNLVRYINNRSVKNRGNAFV  184
            ++   P    +W+ +R++ A+ + S +  +     R ++  +L+ +++  S +
Sbjct  121  SLVWAPSSSLRWRLLRKLSATLLFSPQRMEATKALRMKKVKDLISFMSESSEREEA----  176

Query  185  VIDLRLAVRQYSGNVARKMMFGIRHFGKGSEDGSGPGLEEIEHV-ESLFTVLTHLYAFAL  243
             +D+  A+   + N+   ++F +       + GS   L+E+    +++  V+  +
Sbjct  177  -VDISHALFTTTLNIISNILFSV-------DLGSYDPLKELNGFKDTVIGVMEAIGNPDA  228

Query  244  SDYVPWLRFLDLEGHEKVVSNAMRNVSKYNDPFVDERLMQW---RNGKMKEPQDFLDMFI  300
            ++Y P+LRFLDL+G+ K + + +  + K    F+D ++ +     N K     DF+D  +
Sbjct  229  ANYFPFLRFLDLQGNSKKMKDNIERLFKVFRGFIDAKIAEQSLRNNPKDVTDSDFVDALL  288

Query  301  IAKDTDGKPTLSDEEIKAQVTELMLATVDNPSNAAEWGMAEMINEPSIMQKAVEEIDRVV  360
               + DG   L+  +I+  +++L  A  D  S+  EW MAE+++ P  M+KA  EID V+
Sbjct  289  HLTEGDGA-ELNTNDIEHFLSDLFTAGADTSSSTVEWAMAELLSNPKTMEKAQAEIDHVI  347

Query  361  GKDRLVIESDLPNLNYVKACVKEAFRLHPVAPFNLPHMSTTDTVVDGYFIPKGSHVLISR  420
            G+  +V ESD+  L Y++A VKE FR+HP AP  +P  +  D  V G+ +PK + VL++
Sbjct  348  GQKGIVQESDISELPYLQAVVKETFRMHPAAPLLVPRKAEADVEVLGFMVPKDTQVLVNV  407

Query  421  MGIGRNPSVWDKPHKFDPERHLSTNTCVDLNESDLNIISFSAGRRGCMGVDIGSAMTYML  480
              IGR+PSVW  P +F+PER L   T  D+   D  +  F  GRR C G+ +      ++
Sbjct  408  WAIGRDPSVWKNPTRFEPERFLGKET--DVKGRDYELTPFGGGRRICPGLPLAVKTVNLM  465

Query  481  LARLIQGFTWLPVPGKNKIDISESKN---DLFMAKPLYAV  517
            LA L+  F W    G N  D+   +     L    PL+A+
Sbjct  466  LASLLYSFDWKLPNGINPEDLDMDETFGLTLHKTNPLHAI  505


>XP_009108580.1 PREDICTED: LOW QUALITY PROTEIN: geraniol 8-hydroxylase-like
[Brassica
rapa]
Length=524
```

```
 Score = 215 bits (548),  Expect = 3e-63, Method: Compositional matrix adjust.
 Identities = 145/485 (30%), Positives = 246/485 (51%), Gaps = 28/485 (6%)

Query  49   LIGNLPEILGRNKPVFRWIHSLMKELNTDIACIRLANTHVIPVTSPRIAREILKKQDSVF  108
            ++GNLP +   N  +       + + +  + I L +   I ++SP +ARE+LK  D  F
Sbjct  60   IVGNLPFL---NSDILHTQFQALTQKHGPLMKIHLGSKLAIVISSPYMAREVLKTHDITF  116

Query  109  ATRPLTMGTEYCSRGYLTVAVEPQGEQWKKMRRVVASHVTSKKSFQMMLQKRTEEADNLV  168
            A   L    +  + G   +   P G  W+++R++    + +  + +     R EE    V
Sbjct  117  ANHDLPEVGKINTYGGEDILWSPYGTHWRRLRKLCVMKMFTTPTLEASYSTRREETRQTV  176

Query  169  RYINNRSVKNRGNAFVVIDLRLAVRQYSGNVARKMMFGIRHFGKGSEDGSGPGLEEIEHV  228
             Y++  +   R  A V +  ++ +  +  NV  +MM+G    G   E+ +  G E    +
Sbjct  177  VYMSEMA---RDGAPVNLGEQIFLSIF--NVVTRMMWGATVEG---EERTSLGNE----L  224

Query  229  ESLFTVLTHLYAFA-LSDYVPWLRFLDLEGHEKVVSNAMRNVSKYNDPF--VDERLMQWR  285
            ++L + ++ +      SD+ PW    D +G   +V     +V K +  F  V E  ++
Sbjct  225  KTLISDISDIEGIQNYSDFFPWFARFDFQG---LVKQMKVHVKKLDLLFDRVMESHVKMV  281

Query  286  NGKMKEPQDFLDMFIIAKDTDGKPTLSDEEIKAQVTELMLATVDNPSNAAEWGMAEMINE  345
              K +E +DFL   I  KD D K  LS   +K+ + +++L  VD   NA+E+ MAE+++
Sbjct  282  GKKSEEEEDFLQYLIRVKDEDEKAPLSLTHVKSLLMDMVLGGVDTSVNASEFAMAEIVSR  341

Query  346  PSIMQKAVEEIDRVVGKDRLVIESDLPNLNYVKACVKEAFRLHPVAPFNLPHMSTTDTVV  405
            P + +K  +E+D+VVG D +V ES LP L Y++A +KE  RLHP  P  +PH ++  +VV
Sbjct  342  PEVFKKIRQELDQVVGTDSIVEESHLPKLPYLQAVMKETLRLHPTLPLLVPHRNSETSVV  401

Query  406  DGYFIPKGSHVLISRMGIGRNPSVWDKPHKFDPERHLSTNTCVDLNESDLNIISFSAGRR  465
             GY +PK S + I+   I R+P  WD+P++F PER L  +  +D N  D   + F +GRR
Sbjct  402  AGYTVPKDSKIFINVWAIHRDPKHWDEPNEFKPERFLENS--LDFNGGDFKYLPFGSGRR  459

Query  466  GCMGVDIGSAMTYMLLARLIQGFTWLPVPGKNKIDISESKNDLF--MAKPLYAVATPRLA  523
             C  +++   +    +A L+  F W+  P   K ++ E K  L   +  PL A+  PRL+
Sbjct  460  ICAAINMAERLVLFNIASLLHSFDWV-APKGQKFEV-EXKFGLVLKLKSPLVAIPVPRLS  517

Query  524  -PHVY  527
             P +Y
Sbjct  518  DPKLY  522


>XP_009142346.1 PREDICTED: cytochrome P450 76C4-like [Brassica rapa]
Length=511

 Score = 214 bits (544),  Expect = 8e-63, Method: Compositional matrix adjust.
 Identities = 135/489 (28%), Positives = 238/489 (49%), Gaps = 32/489 (7%)

Query  7    MLAFIIGLLLLALTMKRKEKKKTMLISPTRNLSLPPGPKSWPLIGNLPEILGRNKPVFRW  66
               FI+   L+  T++ +         S     +LPPGP   P+IGN+  ++G+N P   +
Sbjct  12   FFCFILSCFLIFTTVRSRR-------SSHGAAALPPGPPRLPIIGNM-HLVGKN-PHRSF  62

Query  67   IHSLMKELNTDIACIRLANTHVIPVTSPRIAREILKKQDSVFATRPLTMGTEYCSRGYLT  126
             H  + E    +  ++L   + + + SP  ARE+L+ QD + + R              +
Sbjct  63   AH--LSETYGPVMSLKLGCLNTVVIASPNAAREVLRTQDQILSGRYWNEAVRSIDHHSFS  120

Query  127  VA-VEPQGEQWKKMRRVVASHVTSKKSFQMMLQKRTEEADNLVRYINNRSVKNRGNAFVV  185
            VA + P    W+ +R++  +H+ S +  +      R ++   L+ +++  S +
Sbjct  121  VAWLHPSSPLWRLLRKISVTHLFSPQRIEATQALRMKKVQELITFVSECSDREEA-----  175

Query  186  IDLRLAVRQYSGNVARKMMFGIRHFGKGSEDGSGPGLEEIEHVESLFTVLTHLYAFALSD  245
            +D+  A    + N+   ++F I      S   S       +  + +  V+       L+D
Sbjct  176  VDISRASFVTALNIISNILFSIDLGNYDSRKSS-------DFQDMVIGVMESAGNTDLAD  228

Query  246  YVPWLRFLDLEGHEKVVSNAMRNVSKYNDPFVDERL----MQWRNGKMKEPQDFLDMFII  301
            + P++RFLD++G  K   +    + +      D+R+    +Q  +  +   +DFLD  I
Sbjct  229  FFPFMRFLDVQGTRKKFKDCSERLFRAFRRLYDDRIKGNSLQTEDKDVSS-KDFLDALID  287

Query  302  AKDTDGKPTLSDEEIKAQVTELMLATVDNPSNAAEWGMAEMINEPSIMQKAVEEIDRVVG  361
                D +  L+  +I+  + +L  A  D  S+  EW M E++  P  M K  +EID V+G
Sbjct  288  LNQGD-EAELNMYQIEHLLLDLFSAGTDTNSSTVEWAMTELLQNPKAMTKVQDEIDSVIG  346
```

```
Query  362  KDRLVIESDLPNLNYVKACVKEAFRLHPVAPFNLPHMSTTDTVVDGYFIPKGSHVLISRM  421
            ++ +V ESD+  L Y++A VKE FRLHP AP  LP  + TD  + G+ +PK + VL++
Sbjct  347  QNGVVQESDISQLPYLQAVVKETFRLHPAAPLLLPRKAETDVEILGFLVPKDTQVLVNVW  406

Query  422  GIGRNPSVWDKPHKFDPERHLSTNTCVDLNESDLNIISFSAGRRGCMGVDIGSAMTYMLL  481
             +GR+PS W+ P++F+PER L  +T  D+   D  +  F AGRR C G+ +      ++L
Sbjct  407  AVGRDPSTWENPNRFEPERFLGKDT--DVKGKDYELTPFGAGRRICPGLPLAVKTVPLML  464

Query  482  ARLIQGFTW  490
            A L+  F W
Sbjct  465  ASLLYSFDW  473


>XP_009103435.1 PREDICTED: cytochrome P450 81D1-like [Brassica rapa]
Length=500

 Score = 213 bits (543),  Expect = 1e-62, Method: Compositional matrix adjust.
 Identities = 147/519 (28%), Positives = 245/519 (47%), Gaps = 34/519 (7%)

Query  8    LAFIIGLLLLALTMKRKEKKKTMLISPTRNLSLPPGPKSWPLIGNLPEILGRNKPVFRWI  67
             A ++G +  A  + R +            L++PP P  +P+IG+L  +     PV R +
Sbjct  7    FALVVGFIAAAFYLFRSK------------LNVPPSPIGFPVIGHLHLL---KDPVHRCL  51

Query  68   HSLMKELNTDIACIRLANTHVIPVTSPRIAREILKKQ-DSVFATRPLTMGTEYCSRGYLT  126
            H L + L   +  ++L +   + VTS   A E L  + D VFA RP++   +Y +
Sbjct  52   HDLSRNLGP-VFSLKLGSCRAVVVTSASAAEEFLSHENDVVFANRPISTLGKYVAYNNSI  110

Query  127  VAVEPQGEQWKKMRRVVASHVTSKKSFQMMLQKRTEEADNLVRYINNRSVKNRGNAFVVI  186
            V+V P G+ W+ +RR+    + S    +  L+ R +E  +LV+ I+  +    G+  V +
Sbjct  111  VSVSPYGDHWRNLRRICTVEIFSAARLKESLEIRRDEVRSLVQTIHKATTSGGGDNSVRV  170

Query  187  DLRLAVRQYSGNVARKMMFGIRHFGKGSEDGSGPGLEEIEHVESLFTVLTHLYAFALSDY  246
            +LR  +  ++ NV  + + G R++G+ + +      E  E +   F +    YA    D+
Sbjct  171  ELRPLLSGFTLNVLMRTVAGKRYYGEDNAEAK----EVRELISETFELAGCTYA---GDF  223

Query  247  VPWLRFLDLEGHEKVVSNAMRNVSKYNDPFVDERLMQWRNGKMKEPQDFLDMFIIAKDTD  306
            +P L+  D  G+   V      + K+    VDE   +   GK +     +   +  +D+
Sbjct  224  LPILKLFDYNGYVTRVKTLGSKLDKFLQELVDEH--RGNRGKTEFKNTVITHLLTLQDSQ  281

Query  307  GKPTLSDEEIKAQVTELMLATVDNPSNAAEWGMAEMINEPSIMQKAVEEIDRVVG-KDRL  365
             +   +DE IK  V  +++A  D  +   EW MA ++  P ++ KA  E++ VV  K RL
Sbjct  282  PE-YYTDEIIKGLVMVMIVAGTDTTAVTLEWAMANLVKYPDVLAKAKTELNNVVSSKGRL  340

Query  366  VIESDLPNLNYVKACVKEAFRLHPVAPFNLPHMSTTDTVVDGYFIPKGSHVLISRMGIGR  425
            + ESD  N  Y+   + E  RL+P  P  +PH S+ D  V GY IP+G+ + I+   I R
Sbjct  341  MEESDTSNCTYLNNVISETLRLYPAGPMLVPHESSVDCKVAGYDIPRGTWLFINAWAIQR  400

Query  426  NPSVWDKPHKFDPERHLSTNTCVDLNESDLNIISFSAGRRGCMGVDIGSAMTYMLLARLI  485
            +P  WD+P  F PER  S     +L       + F  GRR C G+ +   +    L  LI
Sbjct  401  DPKEWDEPEAFKPERFDSE----ELKTHHGKFLPFGIGRRACPGMGLAQIVLSSALGSLI  456

Query  486  QGFTWLPVPGKNKIDISESKNDLFMAKPLYAVATPRLAP  524
            Q F W     +  +D+SE    L M K +  VA  + +P
Sbjct  457  QCFDW-ERDDEMAVDMSEGTG-LTMPKAVPLVAKCKSSP  493


>XP_009134258.1 PREDICTED: cytochrome P450 76C2-like [Brassica rapa]
Length=516

 Score = 213 bits (541),  Expect = 3e-62, Method: Compositional matrix adjust.
 Identities = 142/495 (29%), Positives = 245/495 (49%), Gaps = 46/495 (9%)

Query  49   LIGNLPEILGRNKPVFRWIHSLMKELNTD---IACIRLANTHVIPVTSPRIAREILKKQD  105
            ++GNLP +    KP    +H+  ++L  +   I  + L +   + V +P +AREILK+QD
Sbjct  51   IVGNLPFL----KPD---LHTYFRDLAQEYGPIFKLNLGSKLTVVVNTPSLAREILKEQD  103

Query  106  SVFATRPLTMGTEYCSRGYLTVAVEPQGEQWKKMRRVVASHVTSKKSFQMMLQKRTEEAD  165
              F+ R + +     + G L +   P   +W+ +R+V    + ++K+   + + R +E
```

```
Sbjct  104  INFSNRDVPLTARAITYGGLDIVWLPYSAEWRMLRKVCVLKLLNRKTLDSIYELRRKEIR  163

Query  166  NLVRYINNRSVK----NRGNAFVVIDLRLAVRQYSGNVARKMMFGIRHFGKGSEDGSGPG  221
               R++  +S +    N G+   V  + L +    G   +
Sbjct  164  ERTRFLYEKSQEEAAVNVGDQLFVTMMNLTINMLWGGSVKA------------------  204

Query  222  LEEIEHVESLFTVLTH-----LYAFALSDYVPWLRFLDLEGHEKVVSNAMRNVSKYNDPF  276
             EE+E V + F V+       L    +SD+ PWL   DL+G  K +    R ++   D
Sbjct  205  -EEMESVGTEFKVVVSEITRLLGEPNISDFFPWLARFDLQGLLKQMRVCSRELNAIFDGA  263

Query  277  VDERLMQWRNGKMKEPQDFLDMFIIAKDTDGKPT--LSDEEIKAQVTELMLATVDNPSNA  334
            + E++ +  +    E +DFL   +  KD +      ++   +KA + ++++   D  +N
Sbjct  264  I-EKMPKLESKDDGECKDFLQQLMKLKDQEANSQVPITVNHVKAVLADMVIGGTDTSTNT  322

Query  335  AEWGMAEMINEPSIMQKAVEEIDRVVGKDRLVIESDLPNLNYVKACVKEAFRLHPVAPFN  394
             E+ MAE+I  P  M++A +E+D VVGKD +V ES +  L Y+ A +KE  RL+P  P
Sbjct  323  IEFAMAELIKNPESMKRAQQELDEVVGKDNIVEESHITRLPYIVAIMKETLRLYPTVPLL  382

Query  395  LPHMSTTDTVVDGYFIPKGSHVLISRMGIGRNPSVWDKPHKFDPERHLSTNTCVDLNESD  454
            +PH      VV GY +PK + + I+   I R+P+VW+ P+KF PER L   +C D + +D
Sbjct  383  VPHRPAETAVVGGYTVPKDTKIFINVWSIQRDPNVWENPNKFRPERFLDKKSC-DFHGTD  441

Query  455  LNIISFSAGRRGCMGVDIGSAMTYMLLARLIQGFTWLPVPGKNKIDISESKNDLFMAK-P  513
             + + F +GRR C GV +   M    LA L+  F W  VP  +++++ +    +   K P
Sbjct  442  YSFLPFGSGRRICAGVALAERMVQYTLATLLHSFDW-KVPEGHELNVEDKFGIVLKLKNP  500

Query  514  LYAVATPRLA-PHVY  527
            L A+  PRL+ P++Y
Sbjct  501  LIAMPFPRLSDPNLY  515


>XP_009129359.1 PREDICTED: cytochrome P450 81D11-like [Brassica rapa]
Length=511

 Score = 211 bits (538),  Expect = 6e-62, Method: Compositional matrix adjust.
 Identities = 149/494 (30%), Positives = 241/494 (49%), Gaps = 31/494 (6%)

Query  36   RNLSLPPGPK-SWPLIGNLPEILGRNKPVFRWIHSLMKELN-TDIACIRLANTHVIPVTS  93
            R L+LPP P   +P+IG+L  +     P+ R   SL K L    I  +RL    V  V+S
Sbjct  35   RKLNLPPSPAWPYPIIGHLHLL---KLPLQRSFLSLSKSLGGASIFSLRLGTRLVYVVSS  91

Query  94   PRIAREILKKQDSVFATRPLTMGTEYCSRGYLTVAVEPQGEQWKKMRRVVASHVTSKKSF  153
              +A E   K D VFA RP  +  ++      T+     G+ W+ +RR+ A  + S
Sbjct  92   HSVAEECFTKNDVVFANRPEFLFAKHIGYNSSTMVSAAYGDSWRNLRRIGAIEIFSSIRI  151

Query  154  QMMLQKRTEEADNLVRYINNRSVKNRGNAFVVIDLRLAVRQYSGNVARKMMFGIRHFGKG  213
               L  R +E   L+  ++  S++        ++L       + N   +M+ G R++G G
Sbjct  152  DSFLSIRKDEIRRLILCLSKNSLQEPAK----VELGSLFMGLTINNIIRMLAGKRYYGDG  207

Query  214  SEDGSGPGLEEIEHVESLFT-VLTHLYAFALSDYVPWLRFLDLEGHEKVVSNAMRNVSKY  272
            +ED      +E +HV  L   V+    A   +DY P L +L    +EK V      V ++
Sbjct  208  TEDD-----DESKHVRDLIAEVIAAGGAGNAADYFPILCWLT--DYEKRVKKLGGRVDEF  260

Query  273  NDPFVDERLMQWRNGKMKEPQDFLDMFIIAKDTDGKPTLSDEEIKAQVTELMLATVDNPS  332
                VDE+    R  K+K     +D  +  ++T  +   +D  IK  +  ++LA  D  +
Sbjct  261  LQSLVDEK----RAEKVK-GSTMIDRLLSLQETQPE-YYTDVIIKGIIEVMILAGTDTSA  314

Query  333  NAAEWGMAEMINEPSIMQKAVEEIDRVVGKDRLVIESDLPNLNYVKACVKEAFRLHPVAP  392
               EW M+ ++N P +++KA  EID  +G DRL+ E D+  L Y+++ V E  RL+PV P
Sbjct  315  ATLEWAMSNLLNHPEVLKKAKTEIDEQIGLDRLIEEQDIVKLPYLQSIVAETLRLYPVVP  374

Query  393  FNLPHMSTTDTVVDGYFIPKGSHVLISRMGIGRNPSVWDKPHKFDPERHLSTNTCVDLNE  452
              LPHM++ D +V GY +P+ + +L++   I R+P +W++P KF PER        +
Sbjct  375  MLLPHMASKDCMVAGYDVPRKTILLVNVWAIHRDPKMWEEPEKFKPERF-------EKEG  427

Query  453  SDLNIISFSAGRRGCMGVDIGSAMTYMLLARLIQGFTWLPVPGKNKIDISESKNDLFMAK  512
             D  ++SF  GRR C G+ +G  +  + L  L+Q F W    G+  +D++E+   + M K
Sbjct  428  EDKKLMSFGMGRRACPGLGLGQRLVTLALGSLVQCFEW-ERTGEEYVDMTEAAKGITMHK  486
```

```
Query  513  PLYAVATPRLAPHV  526
                 A  R  P V
Sbjct  487  STSLEAMCRTRPIV  500


>XP_009150750.1 PREDICTED: cytochrome P450 84A1-like [Brassica rapa]
Length=514

 Score = 211 bits (538),  Expect = 7e-62, Method: Compositional matrix adjust.
 Identities = 153/540 (28%), Positives = 252/540 (47%), Gaps = 49/540 (9%)

Query  1    MLDSTPMLAFIIGLLLLALTMKRKEKKKTMLISPTRNLSLPPGPKSWPLIGNLPEILGRN  60
            ++D TP +  I   LL+ +           LIS       PPGPK  P+IGN+   L  +
Sbjct  12   VIDPTPSVLLITISLLVVV----------YLISQWFKPLFPPGPKGLPVIGNM---LMMD  58

Query  61   KPVFRWIHSLMKELNTDIACIRLANTHVIPVTSPRIAREILKKQDSVFATRPLTMGTEYC  120
            +     +  L  +    +  +R+   HV  +TSP +AR++L+ QD  F+ RP+T+   Y
Sbjct  59   QLTHHGLAKLAHKYG-GLFHLRMGFRHVFAITSPDVARQVLQVQDVSFSNRPVTVAINYL  117

Query  121  SRGYLTVAVEPQGEQWKKMRRVVASHVTSKKSFQMMLQKRTEEADNLVRYINNRSVK--N  178
            +     +A  P G  W++MR+V    V S+K  +     R EE +N+VR +++   K  N
Sbjct  118  TYDLADMAFAPYGPFWRQMRKVCVMKVFSRKRTESWASVR-EEVNNMVRSLSSNVGKPVN  176

Query  179  RGNAFVVIDLRLAVRQYSGNVARKMMFGIRHFGKGSEDGSGPGLEEIEHVESLFTVLTHL  238
             G     +   +  R                FG   E      +  ++    LF
Sbjct  177  VGELIFTLTRNITYRA--------------AFGAACETEQDEFIRILQEFSKLFG-----  217

Query  239  YAFALSDYVPWLRFLDLEGHEKVVSNAMRNVSKYNDPFVDERLMQWRNGKMKEPQDFLDM  298
             AF ++DY+P+L + DL+G  K +  A  ++  + D  +DE + +     + E  D +D+
Sbjct  218  -AFNIADYIPFLGWFDLQGINKRLVKARNDLDGFIDEVIDEHMKKTETVNVDEDTDMVDV  276

Query  299  FIIAKDTDGKPT-------LSDEEIKAQVTELMLATVDNPSNAAEWGMAEMINEPSIMQK  351
             +     D           L+ + IKA V ++M    +  ++  EW + E++  P+ +++
Sbjct  277  LLAFYSEDSSTNRNKNTVKLTRDNIKALVMDVMFGGTETMASGIEWALTELLRNPAELKR  336

Query  352  AVEEIDRVVGKDRLVIESDLPNLNYVKACVKEAFRLHPVAPFNLPHMSTTDTVVDGYFIP  411
              +E+  VVG DR V ++ L  L ++K  +KE  RLHP  P  L H +  DT + G+ +P
Sbjct  337  LQQELTEVVGLDRRVDDTHLEQLTFLKCTLKETMRLHPPIPLIL-HEAIEDTKLQGFSVP  395

Query  412  KGSHVLISRMGIGRNPSVWDKPHKFDPERHLSTNTCVDLNESDLNIISFSAGRRGCMGVD  471
            KGS ++I+   I R+P +W  P  F P R +      D   ++   I F AGRR C G+
Sbjct  396  KGSRLMINAFAIARDPKLWVDPEAFRPSRFMEPGMP-DFMGTNFEFIPFGAGRRSCPGMQ  454

Query  472  IGSAMTYMLLARLIQGFTW-LPVPGK-NKIDISESKN-DLFMAKPLYAVATPRLAPHVYP  528
            +G     + +A +I  FTW LP   K +++D+S+        A  L AV   RL   VYP
Sbjct  455  LGLYAMEVAVANIIHCFTWKLPDGMKPSELDMSDVMGLTAPRATRLIAVPDTRLIGPVYP  514


>XP_009112368.1 PREDICTED: cytochrome P450 81D1-like [Brassica rapa]
Length=499

 Score = 211 bits (537),  Expect = 9e-62, Method: Compositional matrix adjust.
 Identities = 142/489 (29%), Positives = 237/489 (48%), Gaps = 25/489 (5%)

Query  39   SLPPGPKSWPLIGNLPEILGRNKPVFRWIHSLMKELNTDIACIRLANTHVIPVTSPRIAR  98
            +LPP P  +P+IG+L  +    +PV R +  L + L   +  ++L +   + VTS   A
Sbjct  26   NLPPTPVGFPVIGHLHLL---KEPVHRCLRDLSQNLGP-VFSLKLGSCRAVVVTSASAAE  81

Query  99   EILKKQ-DSVFATRPLTMGTEYCSRGYLTVAVEPQGEQWKKMRRVVASHVTSKKSFQMML  157
            E L  Q D VFA RP++    Y       V   P GE W+ +RR+    + S    +
Sbjct  82   EFLTHQNDIVFANRPISTLGVYVGYNNTVVTAAPYGEHWRNLRRICTLEIFSAARLKESF  141

Query  158  QKRTEEADNLVRYINNRSVKNRGNAFVVIDLRLAVRQYSGNVARKMMFGIRHFGKGSEDG  217
            + R +E   L+R I+  +    G++FV ++LR  +   + N+  +M+ G R++G+ +E+
Sbjct  142  EIRRDEVRALLRTIHTETTLG-GDSFVRLELRPLLSGLTFNIVMRMVAGKRYYGEANEEA  200

Query  218  SGPGLEEI-EHVESLFTVLTHLYAFALSDYVPWLRFLDLEGHEKVVSNAMRNVSKYNDPF  276
                 EE+ E +   F V    Y   + D++P L+ LD +G+ K        +  +
Sbjct  201  -----EEVRELISEAFEVGGFTY---VGDFLPILKLLDFDGYVKRGKRLGSKLDVFLQKL  252
```

```
Query  277  VDERLMQWRNGKMKEPQDFLDMFIIAKDTDGKPTLSDEEIKAQVTELMLATVDNPSNAAE  336
            VDE   +   GK +     +   +  +++  +   +DE IK  V  +++A  D  +   E
Sbjct  253  VDEH--RQNRGKTEFKNTMITHLLTLQESQHE-YYTDEIIKGLVLVMLVAGTDTTAVTLE  309

Query  337  WGMAEMINEPSIMQKAVEEIDRVVGKD-RLVIESDLPNLNYVKACVKEAFRLHPVAPFNL  395
            W MA ++N P  + K   E++ VV ++ RL+ ESD+    Y+   + E  RL+P AP  +
Sbjct  310  WAMANLLNHPEALMKTKTELNAVVSREGRLMEESDMGACTYLNNVISETLRLYPAAPLLV  369

Query  396  PHMSTTDTVVDGYFIPKGSHVLISRMGIGRNPSVWDKPHKFDPERHLSTNTCVDLNESDL  455
            PH S+ D  V GY IP+G+ + ++   I R+P VWD+P  F PER  +  +
Sbjct  370  PHASSDDCKVAGYDIPRGTWLFVNAWAIQRDPKVWDEPETFKPERFGNEES----KTQHW  425

Query  456  NIISFSAGRRGCMGVDIGSAMTYMLLARLIQGFTWLPVPGKNKIDISESKNDLFMAKPLY  515
              I F  GRR C G+ +   +  + L  LIQ F W        +D+SE +  L M K +
Sbjct  426  KFIPFGMGRRACPGMGLAHLVLGLTLGSLIQCFDW-ARDADVAVDMSEGRG-LTMPKAVR  483

Query  516  AVATPRLAP  524
             VA  + +P
Sbjct  484  LVAKCKSSP  492
```

>XP_009136071.1 PREDICTED: geraniol 8-hydroxylase [Brassica rapa]
Length=524

Score = 209 bits (533), Expect = 5e-61, Method: Compositional matrix adjust.
Identities = 142/482 (29%), Positives = 242/482 (50%), Gaps = 22/482 (5%)

```
Query  49   LIGNLPEILGRNKPVFRWIHSLMKELNTDIACIRLANTHVIPVTSPRIAREILKKQDSVF  108
            ++GNLP +   N  +       + + +  +  I L +   I V+SP +ARE+LK  D  F
Sbjct  60   IVGNLPFL---NSDILHTQFQALTQKHGPLLKIHLGSQLAIVVSSPDMAREVLKTHDITF  116

Query  109  ATRPLTMGTEYCSRGYLTVAVEPQGEQWKKMRRVVASHVTSKKSFQMMLQKRTEEADNLV  168
            A   L    +  + G   +   P G  W+++R++    + +  + +     R EE    V
Sbjct  117  ANHDLPEVGKINTYGGEDILWSPYGTHWRRLRKLCVMKMFTTPTLEASYSTRREETRQTV  176

Query  169  RYINNRSVKNRGNAFVVIDLRLAVRQYSGNVARKMMFGIRHFGKGSEDGSGPGLEEIEHV  228
             Y++  +   R  + V +  ++ +  +  NV  +MM+G    G   E+ +  G E    +
Sbjct  177  VYMSEMA---RDGSPVNLGEQIFLSIF--NVVTRMMWGATVEG---EERTSLGNE----L  224

Query  229  ESLFTVLTHLYAFA-LSDYVPWLRFLDLEGHEKVVSNAMRNVSKYNDPFVDERLMQWRNG  287
            ++L + ++ +      SD+ P     D +G  K +   ++ +    D  V E  ++
Sbjct  225  KTLISDISDIEGIQNYSDFFPMFARFDFQGLVKKMKVHVKKLDLLFDR-VMESHVKMVGK  283

Query  288  KMKEPQDFLDMFIIAKDTDGKPTLSDEEIKAQVTELMLATVDNPSNAAEWGMAEMINEPS  347
            K +E +DFL   I  KD D K  LS   +K+ + +++L  VD   NA+E+ MAE+++ P
Sbjct  284  KSEEEEDFLQYLIRVKDDDEKAPLSLTHVKSLLMDMVLGGVDTSVNASEFAMAEIVSRPE  343

Query  348  IMQKAVEEIDRVVGKDRLVIESDLPNLNYVKACVKEAFRLHPVAPFNLPHMSTTDTVVDG  407
            + +K  +E+D+VVGKD +V ES LP L Y++A +KE  RLHP  P  +PH ++  +VV G
Sbjct  344  VFKKIRQELDQVVGKDSIVEESHLPKLPYLQAVMKETLRLHPTLPLLVPHRNSETSVVAG  403

Query  408  YFIPKGSHVLISRMGIGRNPSVWDKPHKFDPERHLSTNTCVDLNESDLNIISFSAGRRGC  467
            Y +PK S + I+   I R+P  WD+P++F PER L  +  +D N  D   + F +GRR C
Sbjct  404  YTVPKDSKIFINVWAIHRDPKHWDEPNEFKPERFLENS--LDFNGGDFKYLPFGSGRRIC  461

Query  468  MGVDIGSAMTYMLLARLIQGFTWLPVPGKNKIDISESKNDLFMAK-PLYAVATPRLA-PH  525
              +++   +    +A L   F W   P   K ++ E    +   K PL A+  PRL+ P
Sbjct  462  AAINMAERLVLFNIASLFHSFDW-KAPKGQKFEVEEKFGLVLKLKSPLVAIPVPRLSDPK  520

Query  526  VY  527
            +Y
Sbjct  521  LY  522
```

>XP_009151853.1 PREDICTED: cytochrome P450 81D11 [Brassica rapa]
Length=499

Score = 208 bits (530), Expect = 8e-61, Method: Compositional matrix adjust.

```
 Identities = 144/491 (29%), Positives = 236/491 (48%), Gaps = 33/491 (7%)

Query  36   RNLSLPPGPK-SWPLIGNLPEILGRNKPVFRWIHSLMKELN-TDIACIRLANTHVIPVTS  93
            R L+LPP P  S+P+IG+L  +     P+ R   SL K L    I  +RL    V  V+S
Sbjct  24   RKLNLPPSPAWSFPVIGHLHLL---KMPLQRSFLSLSKSLGGASIFSLRLGYRLVFVVSS  80

Query  94   PRIAREILKKQDSVFATRPLTMGTEYCSRGYLTVAVEPQGEQWKKMRRVVASHVTSKKSF  153
              +A E   K D VFA RP  +  ++       T+   P G+ W+ +RR+    + S
Sbjct  81   HAVAEECFTKNDIVFANRPEFIFAKHIGYNCTTMVSSPYGDNWRNLRRIGTIEIFSSFRL  140

Query  154  QMMLQKRTEEADNLVRYINNRSVKNRGNAFVVIDLRLAVRQYSGNVARKMMFGIRHFGKG  213
               L  R +E   L+  ++    KN  +    +++       + N   +M+ G R +G G
Sbjct  141  DSFLSIRKDEIRRLIFCLS----KNSQHESAKVEMGSLFMSLTLNNIIRMLSGKRFYGDG  196

Query  214  SEDGSGPGLEEIEHVESLFT-VLTHLYAFALSDYVPWLRFLDLEGHEKVVSNAMRNVSKY  272
            +ED +     E  HV  L    +    A   +DY P L ++    +EK V      V K+
Sbjct  197  TEDDN-----EARHVRQLIADAVYSADAGNAADYFPILCWI--TDYEKRVKKLAGRVDKF  249

Query  273  NDPFVDERLMQWRNGKMKEPQDFLDMFIIAKDTDGKPTLSDEEIKAQVTELMLATVDNPS  332
                VDE+  +   G        +D  +  ++T      +D  IK  +  ++LA  D  +
Sbjct  250  LQSLVDEKRAEKEKGNT-----VIDRLLALQETQ-PDYYTDAIIKGIIVVMILAGTDTSA  303

Query  333  NAAEWGMAEMINEPSIMQKAVEEIDRVVGKDRLVIESDLPNLNYVKACVKEAFRLHPVAP  392
               +W M+ ++N P +++KA  EID  +G DRL+   D+  L Y+++ V E  RL+PV P
Sbjct  304  ATLDWAMSNLLNHPEVLKKAKTEIDEQIGSDRLIEGQDIVKLPYLQSIVSETLRLYPVVP  363

Query  393  FNLPHMSTTDTVVDGYFIPKGSHVLISRMGIGRNPSVWDKPHKFDPERHLSTNTCVDLNE  452
              +PHM++ D +V GY +P+G+++L++   I R+P +W++P KF PER        +
Sbjct  364  MLVPHMASEDCMVAGYDVPRGTNLLVNVWAIHRDPEMWEEPEKFKPER-------FEKEG  416

Query  453  SDLNIISFSAGRRGCMGVDIGSAMTYMLLARLIQGFTWLPVPGKNKIDISESKNDLFM--  510
             D  ++ F  GRR C G+ +   +  + L  L+Q F W    G+  +D+SE +  + +
Sbjct  417  EDKKLMPFGMGRRACPGLGLALRLVTLALGSLVQCFEW-ERAGEEYVDMSEDEKGITLRR  475

Query  511  AKPLYAVATPR  521
            A  L AV  PR
Sbjct  476  ATLLEAVCRPR  486


>XP_009114628.1 PREDICTED: cytochrome P450 76C1-like [Brassica rapa]
Length=515

 Score = 209 bits (531),  Expect = 8e-61, Method: Compositional matrix adjust.
 Identities = 139/486 (29%), Positives = 235/486 (48%), Gaps = 37/486 (8%)

Query  49   LIGNLPEILGRNKPVFRWIHSLMKELNTDIACIRLANTHVIPVTSPRIAREILKKQDSVF  108
            ++GNLP +       F    + + + +  I  ++L +   + V SP +AREILK QD  F
Sbjct  50   IVGNLPFLDADLHTYF----TTIAQKHGPIFKLKLGSKLTVVVNSPSLAREILKDQDINF  105

Query  109  ATRPLTMGTEYCSRGYLTVAVEPQGEQWKKMRRVVASHVTSKKSFQMMLQKRTEEADNLV  168
            + R + +     + G L +   P G +W+ +R++  + + S+K+ +   + R +E
Sbjct  106  SNRDVPLTGRVATYGGLDIVWLPYGAEWRILRKLCVTKLLSRKTLESFYELRRQEVRERT  165

Query  169  RYINNRSVKNRGNAFVVIDLRLAVRQYSGNVARKMMFGIRHFGKGSEDGSGPGLEEIEHV  228
            R++  +S +                + + NV  ++   + +       G     E +E V
Sbjct  166  RFLYQQSQE----------------KSAVNVGDQLFLTMMNLTMNMLWGGSVKAENMESV  209

Query  229  ESLFT-VLTHLYAFA----LSDYVPWLRFLDLEGHEKVVSNAMRNVSKYNDPFVDERLMQ  283
             + F  V++ +        +SD+ PWL   DL+G  K     MR  +   D   +  + Q
Sbjct  210  GADFKGVISEMIRLLGEPNVSDFFPWLARFDLQGLVK----QMRVCAHQLDAIFEGAIKQ  265

Query  284  WRNGKMK---EPQDFLDMFIIAKD--TDGKPTLSDEEIKAQVTELMLATVDNPSNAAEWG  338
             +    K   E +DFL   +  KD   + +  ++   +K  +T+++    D  +N  E+
Sbjct  266  MQKLGRKDDDECKDFLQHLMKLKDQEVNSETPITVNHVKGVLTDMVTGGTDTSTNTIEFA  325

Query  339  MAEMINEPSIMQKAVEEIDRVVGKDRLVIESDLPNLNYVKACVKEAFRLHPVAPFNLPHM  398
            MAE+I+ P +M++A +E+D VVGK+ +V ES +   L Y+ A +KE  RLHP  P  +PH
Sbjct  326  MAELISNPKLMKRAQQELDEVVGKENIVEESHITELPYILAIMKETLRLHPTIPLLVPHR  385
```

```
Query  399  STTDTVVDGYFIPKGSHVLISRMGIGRNPSVWDKPHKFDPERHLSTNTCVDLNESDLNII  458
                 VV GY IPK + V I+   I R+P+VW+ P +F PER L   +C D   +D + +
Sbjct  386  PAETAVVGGYAIPKDTKVFINVWSIQRDPNVWENPTEFRPERFLDNKSC-DFTGTDYSFL  444

Query  459  SFSAGRRGCMGVDIGSAMTYMLLARLIQGFTWLPVPGKNKIDISESKNDLFMAK-PLYAV  517
             F +GRR C GV +   M    LA L+  F W  +P    +D+ E    +   K PL A+
Sbjct  445  PFGSGRRICAGVALAERMVLYTLATLLHSFDW-KIPEGCVLDLEEKFGIVLKLKTPLVAL  503

Query  518  ATPRLA  523
              PRL+
Sbjct  504  PVPRLS  509


>XP_009129360.1 PREDICTED: cytochrome P450 81D11-like [Brassica rapa]
Length=500

 Score = 208 bits (530),  Expect = 9e-61, Method: Compositional matrix adjust.
 Identities = 150/521 (29%), Positives = 246/521 (47%), Gaps = 39/521 (7%)

Query  10   FIIGLLLLALTMKRKEKKKTMLISPTRNLSLPPGP-KSWPLIGNLPEILGRNKPVFRWIH  68
            FI  L  L L++K       +  +  R L+LPP P + +P+IG+L  +       F
Sbjct  6    FIPSLFFLVLSLK------LLFGARRRKLNLPPSPTRPFPVIGHLHLLKLPLHRTF---L  56

Query  69   SLMKELN-TDIACIRLANTHVIPVTSPRIAREILKKQDSVFATRPLTMGTEYCSRGYLTV  127
            SL K L+   I  +RL    V  V+S  +A E   K D V A RP  +  +Y      T+
Sbjct  57   SLPKSLDGASIFSLRLGTRLVFVVSSHSVAEECFTKNDIVLANRPEFIFGKYIGYNSSTM  116

Query  128  AVEPQGEQWKKMRRVVASHVTSKKSFQMMLQKRTEEADNLVRYINNRSVKNRGNAFVVID  187
                 G+ W+ +RRV    + S       L  R +E   L+  ++N    N    +  ++
Sbjct  117  VSAAYGDSWRNLRRVGTIEIFSSFRLNSFLSIREDEIRRLIFSLSN----NSQQEYAKVE  172

Query  188  LRLAVRQYSGNVARKMMFGIRHFGKGSEDGSGPGLEEIEHVESLFT-VLTHLYAFALSDY  246
            +R      + N   +M+ G R +G  +ED      +E  HV  L   V     A  ++DY
Sbjct  173  MRTLFMNLTINNILRMVAGKRFYGDETEDD-----DEARHVRQLIADVAVSSGAGNVADY  227

Query  247  VPWLRFLDLEGHEKVVSNAMRNVSKYNDPFVDERLMQWRNGKMKEPQDFLDMFIIAKDTD  306
             P LR +    +EK V      + ++    VDE+    R  K+K     +D  +  ++T
Sbjct  228  FPILRLIT--SYEKQVKKLAGRIDEFLQSLVDEK----RAEKVK-GNTMIDHLLSLQETQ  280

Query  307  GKPTLSDEEIKAQVTELMLATVDNPSNAAEWGMAEMINEPSIMQKAVEEIDRVVGKDRLV  366
                 +D  IK  +  +++A  +      EW MA ++N P ++++A  EI+  +G DRL+
Sbjct  281  -PDYYTDVIIKGIILVMIIAGTNTSGGTLEWAMANLLNHPEVLERARTEIEEQIGSDRLI  339

Query  367  IESDLPNLNYVKACVKEAFRLHPVAPFNLPHMSTTDTVVDGYFIPKGSHVLISRMGIGRN  426
             E D+  L Y++    E  RL+PV P  LPHM++ D +V GY +P+G+ V+++   I R+
Sbjct  340  EEQDIVKLPYLQNITSETLRLYPVVPMLLPHMASEDCIVAGYDVPRGTMVMVNAWAIHRD  399

Query  427  PSVWDKPHKFDPERHLSTNTCVDLNESDLNIISFSAGRRGCMGVDIGSAMTYMLLARLIQ  486
            P++W++P KF PER        +    D  ++SF  GRR C G  +   +  + L  L+Q
Sbjct  400  PNMWEEPEKFKPERF-------EKEGEDKKMLSFGMGRRACPGSGLAQRLVTLALGSLVQ  452

Query  487  GFTWLPVPGKNKIDISESKNDLFM--AKPLYAVATPRLAPH  525
             F W    G+  +D++E++    M  A+PL A+   R   H
Sbjct  453  CFDW-ERDGEKYVDLTEAEKGTIMRKAEPLKAMCRARPIVH  492


>XP_009144655.1 PREDICTED: cytochrome P450 81D1 [Brassica rapa]
Length=501

 Score = 207 bits (527),  Expect = 2e-60, Method: Compositional matrix adjust.
 Identities = 150/522 (29%), Positives = 251/522 (48%), Gaps = 44/522 (8%)

Query  7    MLAFIIGLLLLALTMKRKEKKKTMLISPTRNLSLPPGPKSW-PLIGNLPEILGRNKPVFR  65
            +L  I  L+LL + +K         + P +  +LPP P  W P+IG+L  +     P+ R
Sbjct  9    VLYSIFPLILLIIFLK--------FLKPNKQ-NLPPSPPGWLPVIGHLRLL---KPPIHR  56

Query  66   WIHSLMKELNTD---IACIRLANTHVIPVTSPRIA-REILKKQDSVFATRPLTMGTEYCS  121
             +  L + L+     +  +RL +  V  V+S RIA  E   K D V A RP  +  ++
Sbjct  57   TLRFLTESLHGSGGGVMSLRLGSRLVYVVSSHRIAAEECFGKNDVVLANRPQVIIGKHVG  116
```

```
Query  122  RGYLTVAVEPQGEQWKKMRRVVASHVTSKKSFQMMLQKRTEEADNLVRYINNRSVKNRGN  181
                 +   P G+ W+ +RR+    + S        L  RT+E    VR + +R  ++ G+
Sbjct  117  YNNTNIIAAPYGDHWRNLRRLCTIEIFSTHRLNCFLYVRTDE----VRRLISRLFRSAGS  172

Query  182  AFVVIDLRLAVRQYSGNVARKMMFGIRHFGKGSEDGSGPGLEEIEHVESLFT-VLTHLYA  240
               V++++  +   + N   +MM G R++G+ + D       EE + V  L   V  +  +
Sbjct  173  EKSVVEMKPMLTDLTFNNIMRMMTGKRYYGEETTDE-----EEAKRVRQLVADVGANTSS  227

Query  241  FALSDYVPWLRFLDLEGHEKVVSNAMRNVSKYNDPFVDERLMQWRNGKMKEPQDFLDMFI  300
                DYVP LR      +EK V    +   K+    +D++  Q   G        +D  +
Sbjct  228  GNAVDYVPILRLFS--SYEKRVKELGKKTDKFLQGLIDDKREQQETGNT-----MIDHLL  280

Query  301  IAKDTDGKPTLSDEEIKAQVTELMLATVDNPSNAAEWGMAEMINEPSIMQKAVEEIDRVV  360
            + + +D +   +D+ IK  +  +++A  +  +   EW ++ ++N P +++KA  EID+ V
Sbjct  281  VLQKSDTE-YYTDQIIKGIILVMVIAGTNTSAVTLEWALSNLLNHPDVIRKARTEIDKQV  339

Query  361  GKDRLVIESDLPNLNYVKACVKEAFRLHPVAPFNLPHMSTTDTVVDGYFIPKGSHVLISR  420
            G DRL+ ESDL  L Y+K  V E  RLHP  P  +PHM++ D  V  Y +P+ + +L++
Sbjct  340  GLDRLIEESDLSELPYLKNIVLETLRLHPATPLLVPHMASEDCKVGSYDMPRNTTLLVNA  399

Query  421  MGIGRNPSVWDKPHKFDPERHLSTNTCVDLNESDLNIISFSAGRRGCMGVDIGSAMTYML  480
              I R+P++W  P  F PER           E    +++F  GRR C G  +   +  +
Sbjct  400  WAIHRDPNLWYDPDCFKPERFEKV-------EEAQKLLAFGLGRRACPGSGLAQRIVGLA  452

Query  481  LARLIQGFTWLPVPGKNKIDISE-SKNDLFMAKPLYAVATPR  521
            L  LIQ F W  V G+ ++D+ E + N +  A PL AV   R
Sbjct  453  LGSLIQCFEWERV-GEEEVDMKEGTGNTVPKAVPLQAVCKAR  493


>XP_009111606.1 PREDICTED: cytochrome P450 81D11 [Brassica rapa]
Length=501

 Score = 206 bits (525),  Expect = 4e-60, Method: Compositional matrix adjust.
 Identities = 148/495 (30%), Positives = 239/495 (48%), Gaps = 33/495 (7%)

Query  36   RNLSLPPGP-KSWPLIGNLPEILGRNKPVFRWIHSLMKELNT-DIACIRLANTHVIPVTS  93
            R L+LPP P + +P+IG+   +     P+ R   SL K L+   I  +RL    V+ V+S
Sbjct  26   RKLNLPPSPTRPFPVIGHFHLL---KLPLHRRFLSLSKSLDGGSIFSLRLGTRLVLVVSS  82

Query  94   PRIAREILKKQDSVFATRPLTMGTEYCSRGYLTVAVEPQGEQWKKMRRVVASHVTSKKSF  153
              +A E   K D V A RP  +  ++      T+A    G  W+ +RRV    + S
Sbjct  83   HSVAEECFTKNDIVLANRPEFIVGKHIGYNSTTMAGAAYGGSWRNLRRVGTIEIFSSLRL  142

Query  154  QMMLQKRTEEADNLVRYINNRSVKNRGNAFVVIDLRLAVRQYSGNVARKMMFGIRHFGKG  213
               L  R +E   L+  ++ RS +     F  ++LR      + N   +M+ G R +G
Sbjct  143  NSFLSIRQDEIQRLIFSLSKRSQQE----FAKVELRPLFMSLTINNILRMVAGKRFYGDR  198

Query  214  SEDGSGPGLEEIEHVESLFT-VLTHLYAFALSDYVPWLRFLDLEGHEKVVSNAMRNVSKY  272
            +E+      +E  HV  L   V+    A   +DY P LR++    +EK V       V  +
Sbjct  199  TEND-----DEARHVRQLIAEVVVSGGAGNAADYFPILRWIT--NYEKQVKELAGRVDGF  251

Query  273  NDPFVDERLMQWRNGKMKEPQDFLDMFIIAKDTDGKPTLSDEEIKAQVTELMLATVDNPS  332
                VDE+    R+ K K     +D  +  ++T      +D  IK  +  ++LA  D  +
Sbjct  252  LQSLVDEK----RDEKEK-GNTMIDHLLSLQETQ-PDYYTDVIIKGIILVMILAGTDTSA  305

Query  333  NAAEWGMAEMINEPSIMQKAVEEIDRVVGKDRLVIESDLPNLNYVKACVKEAFRLHPVAP  392
               EW M+ ++N P +++K   EID  +G DRL+ E D+  L Y++  + E  RL+PVAP
Sbjct  306  GTLEWAMSNLLNHPEVLKKDKTEIDEQIGLDRLIEEQDIVKLPYLQNIMSETLRLYPVAP  365

Query  393  FNLPHMSTTDTVVDGYFIPKGSHVLISRMGIGRNPSVWDKPHKFDPERHLSTNTCVDLNE  452
              LPH+++ D +V GY +P+G+ +L++   I R+P +W++P KF PER        +
Sbjct  366  MLLPHLASEDCMVAGYDVPRGAILLVNVWAIHRDPDMWEEPEKFKPER-------FEKEG  418

Query  453  SDLNIISFSAGRRGCMGVDIGSAMTYMLLARLIQGFTWLPVPGKNKIDISESKNDLFM--  510
             D  ++SF  GRR C G  +   +  + L  L+Q F W  V G+  +++ E++    M
Sbjct  419  EDKKLMSFGIGRRACPGSGLAQRLVTLALGSLVQCFEWERV-GEGYVEMKETEKGTIMRK  477

Query  511  AKPLYAVATPRLAPH  525
```

```
             A PL A+   R   H
Sbjct  478  ATPLQAMCRARPIVH  492


>XP_009142712.1 PREDICTED: cytochrome P450 98A3-like [Brassica rapa]
Length=507

 Score = 206 bits (525),  Expect = 4e-60, Method: Compositional matrix adjust.
 Identities = 141/443 (32%), Positives = 220/443 (50%), Gaps = 17/443 (4%)

Query  89   IPVTSPRIAREILKKQDSVFATRPLTMGTEYCSRGYLTVAVEPQGEQWKKMRRVVASHVT  148
            + V+S  +A+E+LK  D   A R     TE  SR    +     G  + K+R+V    +
Sbjct  71   VVVSSAELAKEVLKDHDQKLADRHRNRSTEAFSRNGQDLIWADYGPHYVKVRKVCMLELF  130

Query  149  SKKSFQMMLQKRTEEADNLVRYINNRSVKNRGNAFVVIDLRLAVRQYSGNVARKMMFGIR  208
            + K  + +   R +E   +V  +  R  K   N    I   + +R+Y G VA   +  +
Sbjct  131  TPKRLESLRPIREDEVTAMVESVF-RDCKLPEN----ITKGIQLRKYLGAVAFNNITRLV  185

Query  209  HFGKGSEDGSGPGLEEIEHVESLFTVLTHLYA-FALSDYVPWLRFLDLEGHEKVVSNAMR  267
             FGK   D  G   E+     ++ +    L A  ++++++PWLR++     EK  +
Sbjct  186  -FGKRFVDADGVIDEQGLEFHAIVSNGLKLGASLSIAEHIPWLRWM-CPADEKAFAEHGA  243

Query  268  NVSKYNDPFVDERLMQWRNGKMKEPQDFLDMFIIAKDTDGKPTLSDEEIKAQVTELMLAT  327
                     ++E  +  R       Q F+D  +  KD   +  LS++ I   + +++ A
Sbjct  244  RRDLLTRAIMEEHTLA-RQKFSGPKQHFVDALLTLKD---QYDLSEDTIIGLLWDMITAG  299

Query  328  VDNPSNAAEWGMAEMINEPSIMQKAVEEIDRVVGKDRLVIESDLPNLNYVKACVKEAFRL  387
            +D  + + EW MAEMI  P + QK  EEIDRV+G DR++ E D  +L Y+K  VKE+FRL
Sbjct  300  MDTAAISTEWAMAEMIKNPRVQQKVQEEIDRVIGLDRILTEPDFASLPYLKCVVKESFRL  359

Query  388  HPVAPFNLPHMSTTDTVVDGYFIPKGSHVLISRMGIGRNPSVWDKPHKFDPERHLSTNTC  447
            HP  P  +PH +  D  + GY IPKGS+V ++   +GR+P+ W  P +F PER L  +
Sbjct  360  HPPTPLMVPHRAREDVKIGGYNIPKGSNVHVNVWAVGRDPAAWRNPLEFRPERFLEED--  417

Query  448  VDLNESDLNIISFSAGRRGCMGVDIGSAMTYMLLARLIQGFTWLPVPGKNKIDISESKND  507
            VD+   D  ++ F AGRR C G  +G ++   +L+ L+  F W    G N  +I  S N
Sbjct  418  VDMKGHDFRLLPFGAGRRVCPGAQLGISLVTSMLSHLLHHFVWTLPQGTNTDEIDMSGNP  477

Query  508  ---LFMAKPLYAVATPRLAPHVY  527
                F   P+ AVATPRLA  +Y
Sbjct  478  GIVTFKRTPVQAVATPRLASDLY  500


>XP_009144487.1 PREDICTED: geraniol 8-hydroxylase-like [Brassica rapa]
 XP_009144488.1 PREDICTED: geraniol 8-hydroxylase-like [Brassica rapa]
Length=516

 Score = 206 bits (525),  Expect = 6e-60, Method: Compositional matrix adjust.
 Identities = 140/451 (31%), Positives = 226/451 (50%), Gaps = 27/451 (6%)

Query  81   IRLANTHVIPVTSPRIAREILKKQDSVFATRPLTMGTEYCSRGYLTVAVEPQGEQWKKMR  140
            + L +   + V SP +AREI K QD  F+   + +     + G L +   P G +W+ +R
Sbjct  79   LNLGSKLTVVVNSPSLAREIFKDQDINFSNHDVPLTARIVTYGGLDLVWLPYGAEWRMLR  138

Query  141  RVVASHVTSKKSFQMMLQKRTEEADNLVRYINNRSVKNRGNAFVVIDLRLAVRQYSGNVA  200
            +V    + S+K+     + R +E     R++  +S   +G+A  V D          N+
Sbjct  139  KVCVLKLLSRKTLDSFYELRRKEIRERTRFLYEKS--QQGSAVNVGDQLFLTMM---NLT  193

Query  201  RKMMFG--IRHFGKGSEDGSGPGLEEIEHVESLFTVLTHLYAFALSDYVPWLRFLDLEGH  258
              M++G  +R     +ED    G +E + V S  T L  L    +SD+ PWL   DL+G
Sbjct  194  MNMLWGGSVR-----AEDMESVG-KEFKGVISEITRL--LGEPNVSDFFPWLARFDLQGL  245

Query  259  EKVVSNAMRNVSKYNDPFVD---ERLMQWRNGKMKEPQDFLDMFIIAKDTDGKPT--LSD  313
             K     MR  +   D   D   E++ + R+    E +DFL   +  KD +G     ++
Sbjct  246  VK----KMRVSAHELDAIFDRAIEQMHKLRSSDDGECKDFLQHLMKLKDQEGDSEVPITV  301

Query  314  EEIKAQVTELMLATVDNPSNAAEWGMAEMINEPSIMQKAVEEIDRVVGKDRLVIESDLPN  373
              +KA + ++++   +  +N  E+ MAE+I+ P +M++A +E+D VVGK+ +V ES +
Sbjct  302  NHVKAVLADMVVGGTETSTNTIEFAMAELISNPKLMKRAQQELDEVVGKEHIVEESHITR  361
```

```
Query  374  LNYVKACVKEAFRLHPVAPFNLPHMSTTDTVVDGYFIPKGSHVLISRMGIGRNPSVWDKP  433
            L Y+ A +KE  RLHP  P  +PH  T   VV GY +PK + V I+   I R+P+VW+ P
Sbjct  362  LPYILAIMKETLRLHPTIPLLVPHRPTETAVVGGYTVPKDTKVFINVWSIQRDPNVWENP  421

Query  434  HKFDPERHLSTNTCVDLNESDLNIISFSAGRRGCMGVDIGSAMTYMLLARLIQGFTWLPV  493
             +F PER L   +C D + +D + + F +GRR C GV +   M    LA L+  F W  +
Sbjct  422  TEFCPERFLDNKSC-DFSGTDYSFLPFGSGRRICAGVALAERMVLYTLATLLHSFDW-KI  479

Query  494  PGKNKIDISESKNDLFMAK-PLYAVATPRLA  523
            P    + + E    +   K  L A+  PRL+
Sbjct  480  PEGQVLGLEEKFGIVLKLKTALVALPVPRLS  510


>XP_009137274.1 PREDICTED: geraniol 8-hydroxylase-like [Brassica rapa]
Length=525

 Score = 206 bits (523),  Expect = 1e-59, Method: Compositional matrix adjust.
 Identities = 139/482 (29%), Positives = 243/482 (50%), Gaps = 22/482 (5%)

Query  49   LIGNLPEILGRNKPVFRWIHSLMKELNTDIACIRLANTHVIPVTSPRIAREILKKQDSVF  108
            ++GNLP +   N  +       + + +  +  I L +   I V+SP +ARE+LK  D  F
Sbjct  61   IVGNLPFL---NSDILHTQFQALTQKHGPLMKIHLGSKLAIVVSSPDMAREVLKTHDVTF  117

Query  109  ATRPLTMGTEYCSRGYLTVAVEPQGEQWKKMRRVVASHVTSKKSFQMMLQKRTEEADNLV  168
            A   L    +  + G   +   P G  W+++R++    + +  + +     R EE    +
Sbjct  118  ANHDLPEVGKINTYGGEDILWSPYGTHWRRLRKLCVMKMFTTPTLEASYSTRREETRQTI  177

Query  169  RYINNRSVKNRGNAFVVIDLRLAVRQYSGNVARKMMFGIRHFGKGSEDGSGPGLEEIEHV  228
             +++  +   R  + V +  ++ +  +  NV  +MM+G    G   E+ +  G E    +
Sbjct  178  VHMSEMA---RDGSPVNLGEQIFLSIF--NVVTRMMWGATVEG---EERTSLGNE----L  225

Query  229  ESLFTVLTHLYAFA-LSDYVPWLRFLDLEGHEKVVSNAMRNVSKYNDPFVDERLMQWRNG  287
            ++L + ++ +      SD+ P     D +G  K +   ++ +    D  V E  ++
Sbjct  226  KTLISDISDIEGIQNYSDFFPLFARFDFQGLVKKMKVHVKKLDILFDR-VMESHVKMVGK  284

Query  288  KMKEPQDFLDMFIIAKDTDGKPTLSDEEIKAQVTELMLATVDNPSNAAEWGMAEMINEPS  347
            K +E +DFL   I  KD D K  LS   +K+ + +++L  VD   NA+E+ MAE+++ P
Sbjct  285  KSEEEEDFLQYLIRVKDDDEKAPLSLTHVKSLLMDMVLGGVDTSVNASEFAMAEIVSRPE  344

Query  348  IMQKAVEEIDRVVGKDRLVIESDLPNLNYVKACVKEAFRLHPVAPFNLPHMSTTDTVVDG  407
            + +K  +E+D+VVGKD +V ES LP L Y++A +KE  RLHP  P  +PH ++  +VV G
Sbjct  345  VFKKIRQELDQVVGKDSVVEESHLPKLTYLQAVMKETLRLHPTLPLLVPHRNSETSVVAG  404

Query  408  YFIPKGSHVLISRMGIGRNPSVWDKPHKFDPERHLSTNTCVDLNESDLNIISFSAGRRGC  467
            Y +PK S + I+   I R+P  WD+P++F PER L  +  +D N  D   + F +G R C
Sbjct  405  YTVPKDSKIFINVWAIHRDPKHWDEPNEFKPERFLENS--LDFNGGDFKYLPFGSGGRIC  462

Query  468  MGVDIGSAMTYMLLARLIQGFTWLPVPGKNKIDISESKNDLFMAK-PLYAVATPRLA-PH  525
              +++   +    +A L+  F W   P  +K ++ E    +   K PL A+  PRL+ P
Sbjct  463  AAINMAERLVLFNIASLLHSFDW-KAPKGHKFEVEEKFGLVLKLKSPLVAIPVPRLSDPK  521

Query  526  VY  527
            +Y
Sbjct  522  LY  523


>XP_009145966.1 PREDICTED: cytochrome P450 84A1 [Brassica rapa]
Length=520

 Score = 204 bits (519),  Expect = 5e-59, Method: Compositional matrix adjust.
 Identities = 145/509 (28%), Positives = 244/509 (48%), Gaps = 64/509 (13%)

Query  47   WPLIGNLPEILGRNKPVFRWIHSLMKELNTDIACIRLANTHVIPVTSPRIAREILKKQDS  106
            WP+IGN+  +   ++   R + +L K+    +  +R+   H+  V+SP +A++++L+ QDS
Sbjct  47   WPIIGNMSMM---DQLTHRGLANLAKKYG-GLCHLRMGFLHMYAVSSPDVAKQVLQVQDS  102

Query  107  VFATRPLTMGTEYCSRGYLTVAVEPQGEQWKKMRRVVASHVTSKKSFQMMLQKRTEEADN  166
            VF+ RP T+   Y +     +A   G  W++MR+V    V S+K  +     R +E D
```

```
Sbjct  103   VFSNRPATIAISYLTYDRADMAFAHYGPFWRQMRKVCVMKVFSRKRAESWASVR-DEVDK  161

Query  167   LVRYINNRSVKNRGNAFVVIDLRLAVRQYSGNVARKMMFGIRHFGKGSEDGSGPGLEEIE  226
             ++R +++    N G +  V +   A+ +   N+  +  FG        E G    +  ++
Sbjct  162   MIRSVSS----NVGKSINVGEQIFALTR---NITYRAAFG-----SACEKGQDEFIRILQ  209

Query  227   HVESLFTVLTHLYAFALSDYVPWLRFLDLEGHEKVVSNAMRNVSKYNDPFVDERLMQWRN  286
                 LF       AF ++D++P+  ++D +G  K +  A  ++  + D  +DE +
Sbjct  210   EFSKLFG------AFNVADFIPYFGWIDPQGINKRLVKARNDLDGFIDDIIDEHM-----  258

Query  287   GKMKEPQDFLD----------------------MFIIAKDTDGKPTLSDEEIKAQVTELM  324
              K KE Q+ +D                      +     D      L+ + IKA + ++M
Sbjct  259   -KKKENQNSVDAGDVVDTDMVDDLLAFYSEEAKLVSETADLQNSIKLTRDNIKAIIMDVM  317

Query  325   LATVDNPSNAAEWGMAEMINEPSIMQKAVEEIDRVVGKDRLVIESDLPNLNYVKACVKEA  384
                 +  ++A EW + E++  P  +++  +E+  VVG DR V ESD+  L ++K  +KE
Sbjct  318   FGGTETVASAIEWALTELLRSPEDLKRVQQELAEVVGLDRRVEESDIEKLTFLKCTLKET  377

Query  385   FRLHPVAPFNLPHMSTTDTVVDGYFIPKGSHVLISRMGIGRNPSVWDKPHKFDPERHLST  444
              RLHP  P  L H +  DT +DGYF+PK S V+I+   IGR+P  W     F P R L
Sbjct  378   LRLHPPIPL-LLHETAEDTEIDGYFVPKKSRVMINAFAIGRDPKSWPDAETFRPSRFLEP  436

Query  445   NTCVDLNESDLNIISFSAGRRGCMGVDIGSAMTYMLLARLIQGFTW-LPVPGK-NKIDIS  502
                 D   S+   I F +GRR C G+ +G     + +A ++  FTW LP   K +++D+S
Sbjct  437   GV-PDFKGSNFEFIPFGSGRRSCPGMQLGLYALELAVAHILHCFTWKLPDGMKPSELDMS  495

Query  503   ESKNDLF-----MAKPLYAVATPRLAPHV  526
                 D+F      A  LYAV + RL   V
Sbjct  496   ----DVFGLTAPKATRLYAVPSTRLICSV  520


>XP_009136332.1 PREDICTED: cytochrome P450 81F1 [Brassica rapa]
Length=499

 Score = 203 bits (516),  Expect = 9e-59, Method: Compositional matrix adjust.
 Identities = 144/500 (29%), Positives = 241/500 (48%), Gaps = 33/500 (7%)

Query  30    MLISPTRNLSLPPGPKSWPLIGNLPEILGRNKPVFRWIHSLMKELNTD---IACIRLANT  86
              L S T   +LPPGP S P +G+L  +    KP    IH L++  +     I  +R  +
Sbjct  17    FLFSKTERFNLPPGPPSRPFVGHLHLM----KPP---IHRLLQRYSDKYGPIFSLRFGSR  69

Query  87    HVIPVTSPRIAREILKKQ-DSVFATRPLTMGTEYCSRGYLTVAVEPQGEQWKKMRRVVAS  145
              V+ +TSP +A+E    Q D + ++RPL +  +Y +  + TV   P G+ W+ +RR+ A+
Sbjct  70    RVVVITSPSLAQEAFTGQNDVILSSRPLQLTAKYVAYNHTTVGTAPYGDHWRNLRRICAN  129

Query  146   HVTSKKSFQMMLQKRTEEADNLVRYINNRSV-KNRGNAFVVIDLRLAVRQYSGNVARKMM  204
              + S       L  R +E   ++  ++  +   +    F  ++L   +   + N   +M+
Sbjct  130   EILSNNRITNFLHIRKDEIRRMLTRLSRATTHSDDATRFTHVELEPLLSDLTFNNIVRMV  189

Query  205   FGIRHFGKGSEDGSGPGLEEIEHVESL-FTVLTHLYAFALSDYVPWLRFLDLEGHEKVVS  263
              G  ++G    +      EE E  + L + +  +  A   +DY+P L+    +  ++V
Sbjct  190   TGKTYYGDDVYNK-----EEAELFKKLVYDIAVYSGANHTADYLPVLKLFGNKFEKEV--  242

Query  264   NAMRNVSKYNDPFVDERLMQWRNGKMKEPQDFLDMFIIAKDTDGKPTLSDEEIKAQVTEL  323
                + + K  D  +   L + R  K     + +   +++         +D  IK  +  +
Sbjct  243   ---KALGKSMDDILQRLLDECRRDK---DGNTMVNHLLSLQQQEPEYYTDVIIKGLMMAM  296

Query  324   MLATVDNPSNAAEWGMAEMINEPSIMQKAVEEIDRVVGKDRLVIESDLPNLNYVKACVKE  383
             MLA  +  +   EW M  ++  P +++KA  EID  +GKDRL+ E D+  L Y++  V E
Sbjct  297   MLAGTETSAVTLEWAMTNLVKHPEVLEKARAEIDEKIGKDRLIDEPDVAVLPYLQNVVSE  356

Query  384   AFRLHPVAPFNLPHMSTTDTVVDGYFIPKGSHVLISRMGIGRNPSVWDKPHKFDPERHLS  443
              FRL PVAPF +P   T D  + GY +P+ + VL++   I R+   WD+P +F+P+R
Sbjct  357   TFRLFPVAPFLIPRRPTEDMKIGGYDVPRDTTVLVNAWAIQRDSEFWDEPERFNPDRF--  414

Query  444   TNTCVDLNESDLNIISFSAGRRGCMGVDIGSAMTYMLLARLIQGFTWLPVPGKNKIDISE  503
              N C          ++ F  GRR C G  +G  +  + L  LIQ F W  V G+ +ID+SE
Sbjct  415   DNGCGS-EYYAYKLMPFGNGRRICPGAGLGRRIVTLALGSLIQCFEWESVKGE-EIDMSE  472
```

```
Query  504  SKNDLFMAK--PLYAVATPR  521
            S   L M K  PL A+  PR
Sbjct  473  SAG-LGMRKMDPLRAMCRPR  491
```

>XP_009117291.1 PREDICTED: cytochrome P450 81D11 [Brassica rapa]
Length=497

Score = 202 bits (515), Expect = 1e-58, Method: Compositional matrix adjust.
Identities = 139/488 (28%), Positives = 229/488 (47%), Gaps = 37/488 (8%)

```
Query  38   LSLPPGP-KSWPLIGNLPEILGRNKPVFRWIHSLMKELN-TDIACIRLANTHVIPVTSPR  95
             +LPP P +  PLIG+L  +    +P+ R   +  K L    I  +RL N   + V+S
Sbjct  29   FNLPPSPARPLPLIGHLHLL---KQPLHRTFLTFSKSLGGAPIFSLRLGNCLAVVVSSYS  85

Query  96   IAREILKKQDSVFATRPLTMGTEYCSRGYLTVAVEPQGEQWKKMRRVVASHVTSKKSFQM  155
            +A E   K D V A RP  +  ++    + T+   P G+ W+ +RR+    + S
Sbjct  86   VAEECFTKNDIVLANRPEFIVGKHIEYNFTTMTSAPYGDHWRNLRRISTLEIFSSHKLNS  145

Query  156  MLQKRTEEADNLVRYINNRSVKNRGNAFVVIDLRLAVRQYSGNVARKMMFGIRHFGKGSE  215
             L  RT+E    +R++  R  KN  + F  +++R      + N   +MM G R +G G+E
Sbjct  146  FLSVRTDE----IRHLLLRLSKNSQHGFARVEMRSLFMDLTINNILRMMAGKRFYGDGTE  201

Query  216  DGSGPGLEEIEHVESLFTVLTHLYAFALSDYVPWLRFLDLEGHEKVVSNAMRNVSKYNDP  275
                     + H+  +  V+    A   +DY+P LR+      EK V      V ++
Sbjct  202  QDDNA--RRVRHL--IDEVVAKAGAGNANDYIPILRWFT--NFEKQVKELAGRVDEFLQS  255

Query  276  FVDERLMQWRNGKMKEPQDFLDMFIIAKDTDGKPTLSDEEIKAQVTELMLATVDNPSNAA  335
             VDE+      G        +D  +  ++       +D  +K  +  ++LA  +  +
Sbjct  256  LVDEKRANKEKG-----NTMMDHLLSLQEMQPD-YYTDVTLKGIIIVMILAGTETLTGTL  309

Query  336  EWGMAEMINEPSIMQKAVEEIDRVVGKDRLVIESDLPNLNYVKACVKEAFRLHPVAPFNL  395
            EW M  ++N P +++KA  EID  +G DRL+ E D  +L Y++  V E  RLHP AP  +
Sbjct  310  EWAMLNLLNHPEVLRKARTEIDTKIGFDRLIDEPDTKSLPYLQGIVLETLRLHPAAPTLV  369

Query  396  PHMSTTDTVVDGYFIPKGSHVLISRMGIGRNPSVWDKPHKFDPERHLSTNTCVDLNESDL  455
            PHM++   ++ GY +P+GS +L++   + R+PSVW+ P  F PER        +  +
Sbjct  370  PHMTSDGCMLAGYDVPRGSMLLVNVWAMHRDPSVWEDPEMFKPER-------FENEKEKQ  422

Query  456  NIISFSAGRRGCMGVDIGSAMTYMLLARLIQGFTWLPVPGKNKIDISESKNDLFMAKPLY  515
             ++SF  GRR C GV +   +  + L  ++Q F W    G+  I+  E+        P+
Sbjct  423  KLLSFGIGRRACPGVGLTHRLVTLALGSMVQCFEW-ERTGEEYIETREA--------PMM  473

Query  516  AVATPRLA  523
              ATP LA
Sbjct  474  RPATPLLA  481
```

>XP_009134259.1 PREDICTED: cytochrome P450 76C2-like isoform X1 [Brassica rapa]
Length=516

Score = 202 bits (515), Expect = 2e-58, Method: Compositional matrix adjust.
Identities = 143/495 (29%), Positives = 241/495 (49%), Gaps = 46/495 (9%)

```
Query  49   LIGNLPEILGRNKPVFRWIHSLMKELNTD---IACIRLANTHVIPVTSPRIAREILKKQD  105
            ++GNLP +    KP    +H+  ++L  +   I  + L +   + V +P ++REILK+QD
Sbjct  51   IVGNLPFL----KPD---LHTYFRDLAQEYGPIFKLNLGSKLTVVVNTPSLSREILKEQD  103

Query  106  SVFATRPLTMGTEYCSRGYLTVAVEPQGEQWKKMRRVVASHVTSKKSFQMMLQKRTEEAD  165
              F+ R + +     S G L +   P   +W+ +R+V    + S+K+       R +E
Sbjct  104  INFSNRDVPLTARAISYGGLDIVWLPYSVEWRMLRKVCVLKLLSRKTLDSFYALRRKEIR  163

Query  166  NLVRYINNRSVKNRGNAFVVIDLRLAVRQYSG-NVARKMMFGIRHFGKGSEDGSGPGLEE  224
               R++  +S                 R+ S  NV  ++   + +       GS    EE
Sbjct  164  ERTRFLYEKS-----------------REKSAVNVGDQLFVTMMNLMTNMLWGSSVKAEE  206

Query  225  IEHVESLF----TVLTHLYAFA-LSDYVPWLRFLDLEGHEKVVSNAMRNVSKYNDPFVD-  278
            +E V + F    + +T L     +SD+ PWL   DL+G  K     MR  ++  D   D
Sbjct  207  MESVGTEFKGVVSDITRLLGEPNVSDFFPWLARFDLQGLVK----QMRVYARELDAIFDG  262
```

```
Query  279  --ERLMQWRNGKMKEPQDFLDMFIIAKDTDGKPT--LSDEEIKAQVTELMLATVDNPSNA  334
              E++    +    E +DFL   +  KD +      ++   +KA + ++++   D  +N
Sbjct  263  AIEKMTNLGSKNDGECKDFLQQLMKLKDQEANSEVPITINHVKAVLADMVIGGTDTSTNT  322

Query  335  AEWGMAEMINEPSIMQKAVEEIDRVVGKDRLVIESDLPNLNYVKACVKEAFRLHPVAPFN  394
             E+ MAE+I     M++A  E+D VVGKD +V ES +  L Y+ A +KE+ RL+P  P
Sbjct  323  IEFAMAELIKNQESMKRAQHELDEVVGKDNIVEESHITKLPYIVAIMKESLRLYPTVPLL  382

Query  395  LPHMSTTDTVVDGYFIPKGSHVLISRMGIGRNPSVWDKPHKFDPERHLSTNTCVDLNESD  454
            +PH     TVV GY +PK + + I+   I R+P+VW+ P++F PER L   +C D + +D
Sbjct  383  VPHRPAETTVVGGYTVPKDTKIFINVWSIQRDPNVWENPNEFRPERFLDKKSC-DFHGTD  441

Query  455  LNIISFSAGRRGCMGVDIGSAMTYMLLARLIQGFTWLPVPGKNKIDISESKNDLFMAK-P  513
             + + F +GRR C G+ +   M    LA L+  F W  +P  +  ++ +    +   K P
Sbjct  442  YSFLPFGSGRRICAGLALAERMVQYTLATLLHSFDW-KIPEGHVFNVEDKFVIVLKLKNP  500

Query  514  LYAVATPRLA-PHVY  527
            L A+  PRL+ P +Y
Sbjct  501  LIAMPVPRLSDPDLY  515
```

>XP_009103432.1 PREDICTED: cytochrome P450 81D1-like isoform X1 [Brassica rapa]
Length=498

 Score = 202 bits (513), Expect = 2e-58, Method: Compositional matrix adjust.
 Identities = 141/483 (29%), Positives = 232/483 (48%), Gaps = 26/483 (5%)

```
Query  39   SLPPGPKSWPLIGNLPEILGRNKPVFRWIHSLMKELNTDIACIRLANTHVIPVTSPRIAR  98
            +LPP P  +P+IG+L  +     PV R +    + L   +  +RL +   + VTS   A
Sbjct  26   NLPPSPVGFPVIGHLHLL---KDPVHRCLRDRSQSLGP-VFSLRLGSCRAVVVTSASAAE  81

Query  99   EILKKQ-DSVFATRPLTMGTEYCSRGYLTVAVEPQGEQWKKMRRVVASHVTSKKSFQMML  157
            E L  + D VFA RP+T    Y       V+V P G+ W+ +RR+  + + S    +
Sbjct  82   EFLSHENDVVFANRPITTMYAYVLYSNTGVSVAPYGDHWRHLRRICTTEIFSAARLRESF  141

Query  158  QKRTEEADNLVRYINNRSVKNRGNAFVVIDLRLAVRQYSGNVARKMMFGIRHFGKGSEDG  217
            + R +E  ++++ I+  ++  RGN  V ++LR  +  ++ NV  +M+ G R++G   ED
Sbjct  142  EIRRDEVRSMLQTIHAATL--RGNNSVRVELRPLLSGFTLNVIMRMVAGKRYYG---EDN  196

Query  218  SGPGLEEIEHVESLFTVLTHLYAFA-LSDYVPWLRFLDLEGHEKVVSNAMRNVSKYNDPF  276
            +     E + V  L +    L  F  +  ++P L+  D +G+ K        + K+
Sbjct  197  A-----EAKAVSELISETFELGGFTYVGGFLPILKLFDFDGYVKKSKKIGSKLDKFLQEL  251

Query  277  VDERLMQWRNGKMKEPQDFLDMFIIAKDTDGKPTLSDEEIKAQVTELMLATVDNPSNAAE  336
            VDE   +   GK +     +   +  +++  + + +D+ IK  V  ++ A  D  S   E
Sbjct  252  VDEH--RGNRGKTEFKNTMITHLLTLQESQPE-SYTDQIIKGLVLVMLFAGSDTTSVTLE  308

Query  337  WGMAEMINEPSIMQKAVEEIDRVVGKDR-LVIESDLPNLNYVKACVKEAFRLHPVAPFNL  395
            W MA ++N P ++ K   E++ +V ++R L+ ESD     Y+   + E  RL P AP  +
Sbjct  309  WAMANLLNHPDVLMKVKTELNNLVSRERRLMEESDTSTCTYLDNVISETLRLCPAAPLLV  368

Query  396  PHMSTTDTVVDGYFIPKGSHVLISRMGIGRNPSVWDKPHKFDPERHLSTNTCVDLNESDL  455
            PH S+ D  V GY IP+G+ + I+   I R+P +WD+P  F PER  S
Sbjct  369  PHASSGDCKVAGYDIPRGTWLFINAWAIQRDPKMWDEPEVFKPERFDSEG----WKTQHG  424

Query  456  NIISFSAGRRGCMGVDIGSAMTYMLLARLIQGFTWLPVPGKNKIDISESKNDLFMAKPLY  515
              + F  GRR C G+ +   +  + L  LIQ F W        +D+SE K  L M K L
Sbjct  425  KFLPFGMGRRACPGMGLAQLILSLALGSLIQCFDW-ERDEDVAVDMSEGKG-LTMPKALS  482

Query  516  AVA  518
             VA
Sbjct  483  LVA  485
```

>XP_009142349.1 PREDICTED: cytochrome P450 76C3-like [Brassica rapa]
Length=512

 Score = 202 bits (514), Expect = 2e-58, Method: Compositional matrix adjust.

```
 Identities = 140/489 (29%), Positives = 238/489 (49%), Gaps = 25/489 (5%)

Query  39   SLPPGPKSWPLIGNLPEILGRNKPVFRWIHSLMKELNTDIACIRLANTHVIPVTSPRIAR  98
            +LPPGP   P++GN+ ++        R + +L K +   I  ++L +   I ++SP  A+
Sbjct  37   TLPPGPPKLPVVGNIFQV---GYSPHRSLTTLSK-IYGPIMGLKLGSLTTIVISSPEAAK  92

Query  99   EILKKQDSVFATRPLTMGTEYCSRGYLTVAVEPQGEQWKKMRRVVASHVTSKKSFQMMLQ  158
            E LK QD   + R              + A  P   +W+ +R++   H+ S +    M
Sbjct  93   EALKTQDHHLSARTFNDPVRVFDHHEYSFAWGPPSARWRFLRKITTMHLLSTQRLNAMEP  152

Query  159  KRTEEADNLVRYINNRSVKNRGNAFVVIDLRLAVRQYSGNVARKMMFGIRHFGKGSEDGS  218
             R ++ + L+ +IN    +        +D+  A    + N+    +F        S+
Sbjct  153  LRMKKVEELMSFINKCCEREEA-----VDIARAFFVTALNIISNALFSTDFATHDSKSSH  207

Query  219  GPGLEEIEHVESLFTVLTHLYAFALSDYVPWLRFLDLEGHEKVVSNAMRNVSKYNDPFVD  278
                   E+  ++ +++       + DY P+LRFLDL+G  K  +   + + K    F++
Sbjct  208  -------EYHNTVISLMNVTGKPNVGDYFPFLRFLDLQGTRKEATLCTQRLFKVFQDFIN  260

Query  279  ERLMQWRNGKMKEPQDFLDMF--IIAKDTDGKPTLSDEEIKAQVTELMLATVDNPSNAAE  336
             R+ +  +   ++     DM   ++    + K  LS  EIK  + +L  A  D  S+  E
Sbjct  261  ARMAKRSSQTERKDVSSFDMLNTLLDLTQENKAELSLNEIKHFLQDLFTAGTDTNSSTME  320

Query  337  WGMAEMINEPSIMQKAVEEIDRVVGKDRLVIESDLPN-LNYVKACVKEAFRLHPVAPFNL  395
            W M+E+I  P  M KA  EI + +G++ +V ESD+P+ L Y++A +KE  RLHP AP  +
Sbjct  321  WVMSELIRNPEKMVKAQSEIRQGIGENGVVQESDIPDWLPYLQAILKETLRLHPAAPL-I  379

Query  396  PHMSTTDTVVDGYFIPKGSHVLISRMGIGRNPSVWDKPHKFDPERHLSTNTCVDLNESDL  455
            P  S +D  + G+ IP+ + VL++   IGR+ SVW+ P +F+PER L     +D+   D
Sbjct  380  PRKSESDVHIFGFLIPENASVLVNVWAIGRDSSVWENPMRFEPERFLLRE--IDVKGKDF  437

Query  456  NIISFSAGRRGCMGVDIGSAMTYMLLARLIQGFTWLPVPG--KNKIDISESKN-DLFMAK  512
             +I F AGRR C G+ +      ++LA L+  F W    G     +D++++    L  AK
Sbjct  438  ELIPFGAGRRMCPGMSMALRTMSLVLASLLYSFDWKAQKGVVAENMDMTDAFGVTLRKAK  497

Query  513  PLYAVATPR  521
            PL AV T R
Sbjct  498  PLRAVPTKR  506


>XP_009116769.1 PREDICTED: cytochrome P450 76C3 [Brassica rapa]
Length=497

 Score = 201 bits (512),  Expect = 3e-58, Method: Compositional matrix adjust.
 Identities = 142/488 (29%), Positives = 233/488 (48%), Gaps = 36/488 (7%)

Query  40   LPPGPKSWPLIGNLPEILGRNKPVFRWIHSLMKELNTDIACIRLANTHVIPVTSPRIARE  99
            + PGP   PL+GN+ +I    KP     H  + ++   +  ++L     + ++SP  A+E
Sbjct  30   ISPGPPRLPLLGNILQI--GEKPHRSLDH--LSKIYGSVMTLKLGCLTTVVISSPEAAKE  85

Query  100  ILKKQDSVFATRPLTMGTEYCSRGYLTVAVEPQGEQWKKMRRVVASHVTSKKSFQMMLQK  159
            +LK  D V   R  T           + A  P   +W+ +R++    + S +S
Sbjct  86   VLKTHDHVLCYRISTDPVRATGHHERSFAWLPPFARWRFLRKITTQQLFSTRSLDATKDL  145

Query  160  RTEEADNLVRYINNRSVKNRGNAFVVIDLRLAVRQYSGNVARKMMFGIRHFGKGSEDGSG  219
            R  +   L+ +++  S  +       +++  A    S N+    +F I          +
Sbjct  146  RMSKVQELMSFVDTCSQSSEA-----VNIGRASFITSLNIISNALFSI----------NL  190

Query  220  PGLEEIEHVESLFTVLTHLYAFA----LSDYVPWLRFLDLEGHEKVVSNAMRNVSKYNDP  275
                + E  +    V+  +   A    ++D+ P+L FLDL+G  K     M  + +
Sbjct  191  ANFNDTETTDDFQNVVLRMMEIAGKSNMADFFPFLGFLDLQGTRKEARLCMNKLFRVFQR  250

Query  276  FVDER--LMQWRNGKMKEPQDFLDMFIIAKDTDGKPTLSDEEIKAQVTELMLATVDNPSN  333
            F+D +  L   RN         LD+       + +  L D+ IK  + +L LA  D  S+
Sbjct  251  FIDTKRSLKASRNKDNDMLSSLLDI-----SQEKESELDDDGIKHLLLDLFLAGTDTSSS  305

Query  334  AAEWGMAEMINEPSIMQKAVEEIDRVVGKDRLVIESDLPNLNYVKACVKEAFRLHPVAPF  393
              EW MAE++  P +M KA EEI +++G +  V E D+  L Y++A VKE  RLHP +PF
Sbjct  306  TVEWAMAELLRNPKMMVKAQEEIRQMIGGNDAVRELDIFKLPYLQAVVKETLRLHPPSPF  365
```

```
Query  394  NLPHMSTTDTV-VDGYFIPKGSHVLISRMGIGRNPSVWDKPHKFDPERHLSTNTCVDLNE  452
             +P  S +D V +  + IPK + VL+S   IGR+P+VW+ P +F PER L     +D+
Sbjct  366  LIPRTSESDDVRIFEFIIPKNTQVLVSLWAIGRDPNVWENPTQFKPERFLGRE--IDVKG  423

Query  453  SDLNIISFSAGRRGCMGVDIGSAMTYMLLARLIQGFTWLPVPG--KNKIDISESKN-DLF  509
            +D  +I F AGRR C G+ +G  + +++LA L+ GF W    G     +D+SE+    L
Sbjct  424  NDFELIPFGAGRRICPGLPLGFRIVHLVLASLLYGFDWEYQNGILPENVDMSEAFGVTLH  483

Query  510  MAKPLYAV  517
             A+PL AV
Sbjct  484  KAEPLCAV  491


>XP_009102613.1 PREDICTED: cytochrome P450 76C4-like [Brassica rapa]
Length=504

 Score = 201 bits (511),  Expect = 5e-58, Method: Compositional matrix adjust.
 Identities = 130/446 (29%), Positives = 222/446 (50%), Gaps = 25/446 (6%)

Query  78   IACIRLANTHVIPVTSPRIAREILKKQDSVFATRPLTMGTEYCSRGYLTVA-VEPQGEQW  136
            I  ++    + + VTSP  ARE+LK+ D   + R         +   ++VA + P   +W
Sbjct  71   IMHLKFGRLNTVIVTSPEAAREVLKRHDQTLSGRNSPNSIRSINHQNVSVAWIHPSTARW  130

Query  137  KKMRRVVASHVTSKKSFQMMLQKRTEEADNLVRYINNRSVKNRGNAFVVIDLRLAVRQYS  196
            + +R++ A+H+ S +  +     R ++   L+ +++  S +        +D+  A    +
Sbjct  131  RLLRKLSATHMFSPQRIEATKALRMKKVQELMSFMDESSEREEA-----VDISRASFITT  185

Query  197  GNVARKMMFGIRHFGKGSEDGSGPGLEEIEHVESLFTVLTHLYAFALSDYVPWLRFLDLE  256
             N+   ++F +     GSE  +G         +S+   +    +  L+++ P+L FLDL+
Sbjct  186  LNIISNIIFSVDLCSYGSEISNG-------FHDSVIGGMEAAGSPDLANFFPFLGFLDLQ  238

Query  257  GHEKVVSNAMRNVSKYNDPFVDERLMQ--WRNG-KMKEPQDFLDMFIIAKDTDGKPTLSD  313
            G+ K +      + K    F+D +  +   RN  K    +DFLD  +     D +  L +
Sbjct  239  GNSKRMKFCTERLFKVFRGFIDIKTAEKSLRNDPKDASNRDFLDALLDLTVGD-EAELDN  297

Query  314  EEIKAQVTELMLATVDNPSNAAEWGMAEMINEPSIMQKAVEEIDRVVGKDRLVIESDLPN  373
             +I+  + ++ +A  D  SN  EW MAE++  P  M KA  EI R++G++  V E D+
Sbjct  298  NDIEHLLLDMFIAGTDTSSNTVEWAMAELLTNPKTMVKAQSEIQRIIGQNGFVQEPDISE  357

Query  374  LNYVKACVKEAFRLHPVAPFNLPHMSTTDTVVDGYFIPKGSHVLISRMGIGRNPSVWDKP  433
            L Y++A VKE  RLHP  P  LP  +  D  V GY +PK + VL++   IGR+P+VW+ P
Sbjct  358  LPYIQAVVKETLRLHPAVPLLLPRKAEKDVEVFGYLVPKDAQVLVNVWAIGRDPNVWENP  417

Query  434  HKFDPERHLSTNTCVDLNESDLNIISFSAGRRGCMGVDIGSAMTYMLLARLIQGFTW-LP  492
             +F+P+R L     +D+   D  +  F AGRR C G+ +   M  ++L  L+  F W LP
Sbjct  418  TQFEPDRFLGEE--IDVKGRDYELTPFGAGRRICPGLPLAVKMVSLMLVSLLYSFDWKLP  475

Query  493  VPGKNKIDISESKN-DLFMAKPLYAV  517
                N +D+ E+    L  A PL+ V
Sbjct  476  ----NTVDMEETFGITLHKANPLHVV  497


>XP_009133990.1 PREDICTED: cytochrome P450 81D11-like [Brassica rapa]
Length=499

 Score = 201 bits (510),  Expect = 6e-58, Method: Compositional matrix adjust.
 Identities = 146/495 (29%), Positives = 237/495 (48%), Gaps = 40/495 (8%)

Query  38   LSLPPGPK-SWPLIGNLPEILGRNKPVFRWIHSLMKELN-TDIACIRLANTHVIPVTSPR  95
             +LPP P  S P+IG+L  +     P+ + +HSL + L    I  +RL N     V+S
Sbjct  29   FNLPPTPAWSLPVIGHLHLL---KPPLHQILHSLSQSLGGAPIFRLRLGNRVAFVVSSLS  85

Query  96   IAREILKKQDSVFATRP-LTMG--TEY-CSRGYLTVAVEPQGEQWKKMRRVVASHVTSKK  151
            +A E   K D   A RP  T G   EY C+    T+A    G+ W+ +RR+ A  + S
Sbjct  86   LAEECFTKNDIALADRPKFTFGRLVEYNCT----TMATTSYGDHWRNLRRIGAIEIFSSH  141

Query  152  SFQMMLQKRTEEADNLVRYINNRSVKNRGNAFVVIDLRLAVRQYSGNVARKMMFGIRHFG  211
                 L  R +E  +L+  ++    KN  + F  ++LR     ++ N   +M+ G R +G
Sbjct  142  RLDSFLSIRKDEIRHLILCLS----KNSLHVFAKVELRSLFGNFNINNILRMIAGKRFYG  197
```

```
Query  212  KGSEDGSGPGLEEIEHVESLF-TVLTHLYAFALSDYVPWLRFLDLEGHEKVVSNAMRNVS  270
              +E G     +E + V  L    ++       SDYVP+LR+     +EK V      V
Sbjct  198  DEAEQG-----DEAKRVRQLLDEAVSSAGVGHASDYVPFLRWF--TSYEKGVKKLAVRVD  250

Query  271  KYNDPFVDERLMQWRNGKMKEPQDFLDMFIIAKDTDGKPTLSDEEIKAQVTELMLATVDN  330
            ++    +D++  Q   G        +D  +  ++T+     +D  +K  V  ++ A
Sbjct  251  EFLQGLLDDKRAQKEKGNT-----MIDHLLSLQETE-PDYYTDVTVKGLVVVMIFAGNVT  304

Query  331  PSNAAEWGMAEMINEPSIMQKAVEEIDRVVGKDRLVIESDLPNLNYVKACVKEAFRLHPV  390
             +   EW M  ++N P +++KA  EID  +G DRL+ E D  NL Y++  + E FRL+P
Sbjct  305  LTRTLEWAMLNLLNHPEVLKKAKTEIDTKIGLDRLIDEPDAKNLPYLQCIISEIFRLYPA  364

Query  391  APFNLPHMSTTDTVVDGYFIPKGSHVLISRMGIGRNPSVWDKPHKFDPERHLSTNTCVDL  450
            AP   PH +T D ++ GY  P+G+ ++ +   I R+P++W++P KF PER        +
Sbjct  365  APLLAPHRATEDCIIGGYDFPRGTTLIANVWAIHRDPNIWEEPEKFKPER-------FER  417

Query  451  NESDLNIISFSAGRRGCMGVDIGSAMTYMLLARLIQGFTWLPVPGKNKIDISESKNDLFM  510
               D  ++ F  GRR C G  +   +  + L  +IQ F W  + G+  +DISE+   +
Sbjct  418  KGGDQTLMPFGMGRRACPGSGLAQRVVNLALGSMIQCFDWERI-GEEFVDISEATT-MRP  475

Query  511  AKPLYAVATPRLAPH  525
            A PL A+   R   H
Sbjct  476  ATPLLAMCRARPLVH  490


>XP_009136438.1 PREDICTED: cytochrome P450 81F1-like [Brassica rapa]
Length=498

 Score = 200 bits (508),  Expect = 1e-57, Method: Compositional matrix adjust.
 Identities = 140/499 (28%), Positives = 235/499 (47%), Gaps = 32/499 (6%)

Query  30   MLISPTRNLSLPPGPKSWPLIGNLPEILGRNKPVFRWIHSLMKELNTDIACIRLANTHVI  89
             + S     +LPPGP S P++G+L  +     P+ R + S   +    I  +R  +  V+
Sbjct  17   FIYSEMHRFNLPPGPPSRPIVGHLHLM---KPPIHRLLQSFANKYGP-IFSLRFGSRRVV  72

Query  90   PVTSPRIAREILKKQDSV-FATRPLTMGTEYCSRGYLTVAVEPQGEQWKKMRRVVASHVT  148
             +TS  + +E    Q+ +  ++RP  +  +Y +  Y TV   P G+ W+ +RR+ A  +
Sbjct  73   VITSSSLVQEAFTGQNDINLSSRPFQLTAKYVAYNYTTVGTAPYGDHWRNLRRICALEIL  132

Query  149  SKKSFQMMLQKRTEEAD-NLVRYINNRSVKNRGNAFVVIDLRLAVRQYSGNVARKMMFGI  207
            S       L  R +E    L+R   + +    G+ F  ++L       + N   +M+ G
Sbjct  133  SSNRLTNFLHIRKDEIRIMLMRLSRDTTHSQGGSCFTHVELEPLFSDLTFNNIVRMVTGK  192

Query  208  RHFGKGSEDGSGPGLEEIEHVESL-FTVLTHLYAFALSDYVPWLRFLDLEGHEKVVSNAM  266
            R++G    +      EE E  + L + +  +  A   +DY+P L+    +  E+V
Sbjct  193  RYYGDDVNNK-----EEAELFKKLVYDIAVYSGANHTADYLPVLKLFGNKFEEEV-----  242

Query  267  RNVSKYNDPFVDERLMQWRNGKMKEPQDFLDMFIIAKDTDGKPTLSDEEIKAQVTELMLA  326
            + + K  D  +    L + R  K     + +   +++         SD  IK  +  +MLA
Sbjct  243  KALGKSMDEILQRLLDECRRDK---DGNTMVTHLLSLQEQEPEYYSDVTIKGLMMAMMLA  299

Query  327  TVDNPSNAAEWGMAEMINEPSIMQKAVEEIDRVVGKDRLVIESDLPNLNYVKACVKEAFR  386
              +  +   EW M  ++  P +++KA  EID  +G+ RL+ E D+  L Y++  V E FR
Sbjct  300  GTETSAITLEWAMTNLLRHPDVLKKARSEIDEKIGEGRLIDEPDIAVLPYLQDVVSETFR  359

Query  387  LHPVAPFNLPHMSTTDTVVDGYFIPKGSHVLISRMGIGRNPSVWDKPHKFDPERH--LST  444
            L PVAP  +P   T D  + GY IP+ + V+++   I R+P +WD P +F+P+R     +
Sbjct  360  LFPVAPLLVPRTPTEDMKIGGYDIPRETIVIVNAWAIHRDPELWDDPERFNPDRFKGCGS  419

Query  445  NTCVDLNESDLNIISFSAGRRGCMGVDIGSAMTYMLLARLIQGFTWLPVPGKNKIDISES  504
              C         ++ F  GRR C G  +G  +  + L  LIQ F W  V G+ +ID+SES
Sbjct  420  ELCA------YKLMPFGNGRRVCPGAGLGRRIVTLALGSLIQCFDWENVKGE-EIDMSES  472

Query  505  KNDLFMAK--PLYAVATPR  521
               L M K  PL A+  PR
Sbjct  473  TG-LGMHKLDPLRAMCRPR  490
```

```
>XP_009142347.1 PREDICTED: cytochrome P450 76C2-like [Brassica rapa]
Length=509

 Score = 200 bits (509),  Expect = 1e-57, Method: Compositional matrix adjust.
 Identities = 141/520 (27%), Positives = 254/520 (49%), Gaps = 37/520 (7%)

Query  7    MLAFIIGLLLLALTMKRKEKKKTMLISPTRNLSLPPGPKSWPLIGNLPEILGRNKPVFRW  66
            +  FI+   L+  T +   ++K+   + T     PPGP   P+IGN+  ++G+N
Sbjct  12   LFCFILSCFLIICTTR--SRRKSFEAAT-----PPGPPRLPIIGNI-HLVGKNPH----  59

Query  67   IHSL--MKELNTDIACIRLANTHVIPVTSPRIAREILKKQDSVFATRPLTMGTEYCSRGY  124
             HS   + +    +  ++  + + + +TSP  A+E+L+  D V + R  T       +
Sbjct  60   -HSFANLSKTYGPVMSLKFGSLNTVIITSPEAAKEVLRTHDQVLSWRSSTNSIRSINHHE  118

Query  125  LTVA-VEPQGEQWKKMRRVVASHVTSKKSFQMMLQKRTEEADNLVRYINNRSVKNRGNAF  183
            ++V  + P   +W+ +R++  + + S +  +     R  +   LV ++N  S ++
Sbjct  119  VSVVWLPPSSARWRLLRKLSVTLLFSPQRIEATKALRLNKVKELVSFMNESSERDEP---  175

Query  184  VVIDLRLAVRQYSGNVARKMMFGIRHFGKGSEDGSGPGLEEIEHVESLFTVLTHLYAFAL  243
              +D+  A    + N+   ++F +       + GS    +  E  +++  V+  +
Sbjct  176  --VDISRASFITALNIISNILFSV-------DLGSYDLKKSNEFQDAVIGVMEAIGNPDA  226

Query  244  SDYVPWLRFLDLEGHEKVVSNAMRNVSKYNDPFVDERLMQ--WRNGKMK-EPQDFLDMFI  300
            ++Y P+L FLD++G+ K +  A   + +    F+D ++ +   RN +      DF+D  +
Sbjct  227  ANYFPFLAFLDMQGNRKAMKVASEKLFRVFRGFIDAKIAKRSLRNSRENVSTHDFVDALL  286

Query  301  IAKDTDGKPTLSDEEIKAQVTELMLATVDNPSNAAEWGMAEMINEPSIMQKAVEEIDRVV  360
                 D +  L+  +    + +L  A  D  S+  EW MAE++  P  M +A  EID V+
Sbjct  287  DLTKED-EVELNTNDFVHLLLDLFGAGTDTNSSTVEWAMAELLRSPKTMTRAQAEIDHVI  345

Query  361  GKDRLVIESDLPNLNYVKACVKEAFRLHPVAPFNLPHMSTTDTVVDGYFIPKGSHVLISR  420
            G+   V ESD+  L Y++A VKE FRLHP AP  +P  + +D  V G+ +PK + VL++
Sbjct  346  GQKGFVEESDISELPYLQAVVKEVFRLHPAAPLLVPRKAESDVEVQGFKVPKDTQVLVNV  405

Query  421  MGIGRNPSVWDKPHKFDPERHLSTNTCVDLNESDLNIISFSAGRRGCMGVDIGSAMTYML  480
              IGR+PSVW+ P +F+PER L   T  D+   D  +  F A R+ C G+ +   +  ++
Sbjct  406  WAIGRDPSVWENPTRFEPERFLGKET--DVRGRDYELTPFGARRKICPGLPLAVKIVPLM  463

Query  481  LARLIQGFTW-LPVP-GKNKIDISESKN-DLFMAKPLYAV  517
            LA L+  F W LP       +D+ E+    L    PL+AV
Sbjct  464  LASLLYSFDWKLPNGVASEDLDMDETFGLTLHKTNPLHAV  503


>XP_009129560.1 PREDICTED: cytochrome P450 71B23-like [Brassica rapa]
Length=501

 Score = 199 bits (507),  Expect = 2e-57, Method: Compositional matrix adjust.
 Identities = 133/475 (28%), Positives = 230/475 (48%), Gaps = 31/475 (7%)

Query  35   TRNLSLPPGPKSWPLIGNLPEILGRNKPVFRWIHSLMKELNTD---IACIRLANTHVIPV  91
            T  ++LPPGP+  P+IGNL  + G        +H  +++L+     +  ++     ++ +
Sbjct  26   TSKINLPPGPQKLPIIGNLHNLDG-------LLHICLQKLSKTYGPVMKLQFGFVPIVII  78

Query  92   TSPRIAREILKKQDSVFATRPLTMGTEYCSRGYLTVAVEPQGEQWKKMRRVVASHVTSKK  151
            +S + A E+LK  D    +RP T+ T+  S  +  +    P GE+W+ +R++    + S K
Sbjct  79   SSNKAAEEVLKTHDLDCCSRPETIATKKISYNFKDIGFAPYGEEWRALRKLAVIELFSLK  138

Query  152  SFQMMLQKRTEEADNLVRYINNRSVKNRGNAFVVIDLRLAVRQYSGNVARKMMFGIRHFG  211
                    R EE D LV+ ++  S K         ++L+ A+     ++  ++ FG
Sbjct  139  KLNSFRYIREEENDLLVKKLSEASQKQSP-----VNLKKALFTLVASIICRLAFGQNL--  191

Query  212  KGSEDGSGPGLEEIEHVESLFTVLTHLYAFALSDYVPWLRFLD-LEGHEKVVSNAMRNVS  270
              SE     G+EE+                FA S++ P    +D + G  K +      +
Sbjct  192  HESEFIDEDGMEELASRSEKLQA-----KFAFSNFFPGGWIIDRITGQSKSLEGLFSELD  246

Query  271  KYNDPFVDERLMQWRNGKMKEPQDFLDMFII---AKDTDGKPTLSDEEIKAQVTELMLAT  327
             + +  +D+ L   R+  + E  D +D+ I      +  +G   L+ + IK  ++++ LA
Sbjct  247  AFFNQVLDDHLKPGRS--VLESPDVVDVMIDMMNKQGGEGSFKLTTDHIKGIISDIFLAG  304
```

```
Query  328  VDNPSNAAEWGMAEMINEPSIMQKAVEEIDRVVGKDRL-VIESDLPNLNYVKACVKEAFR  386
            V+  +    W M E++  PS+M+K  +E+  V+G+ R  + E DL  LNY K  +KE FR
Sbjct  305  VNTSATTILWAMTELMRNPSVMKKVQDEVRTVLGESRQRITEEDLNQLNYFKLVIKETFR  364

Query  387  LHPVAPFNLPHMSTTDTVVDGYFIPKGSHVLISRMGIGRNPSVWDKPHKFDPERHLSTNT  446
            LHP AP  LP  + +   V GY IPK + ++I+   I R+P +W  P +F PER   ++
Sbjct  365  LHPTAPLLLPRETMSPIKVQGYDIPKKTQIMINVYAIARDPKLWANPDEFKPERF--SDI  422

Query  447  CVDLNESDLNIISFSAGRRGCMGVDIGSAMTYMLLARLIQGFTWLPVPGKNKIDI  501
             VD    +  ++ F +GRR C G+ +G AM  + L  L+  F W+   G    DI
Sbjct  423  SVDYRGLNFELLPFGSGRRICPGMTMGIAMVELGLLNLLYFFDWVMPEGTTVKDI  477


>XP_009138490.1 PREDICTED: cytochrome P450 81D11-like [Brassica rapa]
Length=492

 Score = 199 bits (505),  Expect = 2e-57, Method: Compositional matrix adjust.
 Identities = 136/481 (28%), Positives = 231/481 (48%), Gaps = 34/481 (7%)

Query  28   KTMLISPTRNLSLPPGPK-SWPLIGNLPEILGRNKPVFRWIHSLMKEL-NTDIACIRLAN  85
            + +L    R L LPP P  S P++G+L        P+ RW  SL K + N  I  +RL N
Sbjct  19   RFLLTKSNRKLKLPPSPAISLPVLGHLHLF---KTPLHRWFLSLSKSIGNAPIFHLRLGN  75

Query  86   THVIPVTSPRIAREILKKQDSVFATRPLTMGTEYCSRGYLTVAVEPQGEQWKKMRRVVAS  145
              +  VTS  IA E   + D V A RP  + +++       +   P G+ W+ +RR+ A
Sbjct  76   RLIYVVTSRSIAEECFTENDVVLANRPKFIVSKHVGYDATHLLSAPYGDHWRNLRRIAAV  135

Query  146  HVTSKKSFQMMLQKRTEEADNLVRYINNRSVKNRGNAFVVIDLRLAVRQYSGNVARKMMF  205
             + S +     L  R +E    +R + +R  ++  + FV ++++  +   + N   +M+
Sbjct  136  ELLSTQRLNSFLYIRKDE----IRRLISRLSRDSFHGFVEVEMKSLLGNLASNNIIRMLA  191

Query  206  GIRHFGKGSEDGSGPGLEEIEHVESLFT-VLTHLYAFALSDYVPWLRFLDLEGHEKVVSN  264
            G R++G+ ++        E + V  L   V+    A   +DY+  +R+     +EK + +
Sbjct  192  GKRYYGEEND--------EAKFVRQLVAEVVISSGAGNPADYLSVVRWF--TNYEKRIKD  241

Query  265  AMRNVSKYNDPFVDERLMQWRNGKMKEPQDFLDMFIIAKDTDGKPTLSDEEIKAQVTELM  324
                   +    VDE+      G     Q  +D  +  ++T      +D+ IK  +  L
Sbjct  242  LGNRFDTFLQRIVDEKRADKEKG-----QTMIDRLLSLQETQ-PDYYTDDIIKGLILSLT  295

Query  325  LATVDNPSNAAEWGMAEMINEPSIMQKAVEEIDRVVGKDRLVIESDLPNLNYVKACVKEA  384
            +   D  +   EW M+ ++N P +++KA  EID  +G DRLV E D+ NL Y++  V E
Sbjct  296  IGGTDTTAVTLEWAMSNLLNYPEVLRKARNEIDDKIGFDRLVDEPDIVNLPYLQNIVSET  355

Query  385  FRLHPVAPFNLPHMSTTDTVVDGYFIPKGSHVLISRMGIGRNPSVWDKPHKFDPERHLST  444
             RL+P  P  LPH+S+ D  + GY +P+G+ VL +   + R+P++W+    F PER
Sbjct  356  LRLYPAVPLLLPHLSSNDFKLAGYDVPRGTMVLTNVWAMQRDPTLWEDAEMFKPERFDKA  415

Query  445  NTCVDLNESDLNIISFSAGRRGCMGVDIGSAMTYMLLARLIQGFTWLPVPGKNKIDISES  504
                   E+D  ++ F  GRR C G  +   +  ++L  L+Q F W  V G   +D++E
Sbjct  416  ------GEAD-KLLPFGMGRRACPGAGLAQRLVSLVLGTLVQCFEWERV-GDELVDMTED  467

Query  505  K  505
            K
Sbjct  468  K  468


>XP_009101907.1 PREDICTED: cytochrome P450 84A1-like [Brassica rapa]
Length=513

 Score = 199 bits (506),  Expect = 3e-57, Method: Compositional matrix adjust.
 Identities = 128/473 (27%), Positives = 224/473 (47%), Gaps = 44/473 (9%)

Query  35   TRNLSLPPGPKSWPLIGNLPEILGRNKPVFRWIHSLMKELNTDIACIRLANTHVIPVTSP  94
            TR    PPGPK +P+IGN+     +N+   R +  L K+    +  +++   H++  ++
Sbjct  26   TRGNPFPPGPKGYPIIGNMKL---KNQLNHRGLAELAKQYGG-LLHLQMGRIHIVAASTA  81

Query  95   RIAREILKKQDSVFATRPLTMGTEYCSRGYLTVAVEPQGEQWKKMRRVVASHVTSKKSFQ  154
             +AREIL+ QD VFA RP  +   Y +     +A    G  W++MR+V    + S+K  +
Sbjct  82   EMAREILQVQDVVFANRPANVAISYLTYNRADMAFANYGPLWRQMRKVCVMKLFSRKRAE  141
```

```
Query  155  MMLQKRTEEADNLVRYINNRSVK--NRGNAFVVIDLRLAVRQYSGNVARKMMFGIRHFGK  212
                 R +E + +V+ +  ++    N G     +   +  R   G+ AR
Sbjct  142  SWASVR-DEINTMVQTLTKQTCSPVNVGELVFALTRNITYRAAFGSFAR-----------  189

Query  213  GSEDGSGPGLEEIEHVESLFTVLTHLYAFALSDYVPWLRFLDLEGHEKVVSNAMRNVSKY  272
               DG    ++ ++    LF       AF +++++PW+++       + + NA +++  +
Sbjct  190  ---DGQDEFVKILQEFSKLFG------AFDITEFLPWMKWFSNRDFSERLENARKSLDGF  240

Query  273  NDPFVDERLMQWRNGKMKE---PQDFLDMFII------------AKDTDGKPTLSDEEIK  317
             D  +D  + +  + K  +     D +D  +             + D+     L+ + IK
Sbjct  241  IDRIIDAHIEKKNSRKQDDDGLEDDMVDELMAFYSVESGENGGKSNDSLSSFKLTRDNIK  300

Query  318  AQVTELMLATVDNPSNAAEWGMAEMINEPSIMQKAVEEIDRVVGKDRLVIESDLPNLNYV  377
            A V ++M    +  ++A EW M E++  P  + K  +E+  V+G +R   ESDL NL Y
Sbjct  301  ALVMDVMFGGTETVASAIEWAMTELMKNPHELVKLQQELADVIGLNREFHESDLENLPYF  360

Query  378  KACVKEAFRLHPVAPFNLPHMSTTDTVVDGYFIPKGSHVLISRMGIGRNPSVWDKPHKFD  437
            +  +KE  RLHP  P  L H +  D+VV GY IP+ S V+I+   IGR+ SVW +P  F
Sbjct  361  RCAMKETLRLHPPIPL-LLHEAAADSVVSGYSIPRDSRVMINVYAIGRDGSVWTEPDAFR  419

Query  438  PERHLSTNTCVDLNESDLNIISFSAGRRGCMGVDIGSAMTYMLLARLIQGFTW  490
            P R + +    D   SD   + F +GRR C G+ +G     + +A ++  F W
Sbjct  420  PGRFMDSKA-PDFKGSDFEFLPFGSGRRSCPGMQLGLYAMELAVAHMLHSFDW  471


>XP_009137862.1 PREDICTED: cytochrome P450 CYP82D47-like [Brassica rapa]
Length=529

 Score = 199 bits (505),  Expect = 6e-57, Method: Compositional matrix adjust.
 Identities = 144/531 (27%), Positives = 261/531 (49%), Gaps = 31/531 (6%)

Query  7    MLAFIIGLLLLALTMKRKEKKKTMLISPTRNLSLPPGPKSWPLIGNLPEILGRNKPVFRW  66
             L F + L+ L   + R +K K          + P  P +WP++G+LP +    KP
Sbjct  7    FLLFALSLIPLCFLILRPKKSKG---------TAPMVPGAWPVVGHLP-MYRSVKPQAHV  56

Query  67   IHSLMKELNTDIACIRLANTHVIPVTSPRIAREILKKQDSVFATRPLTMGTEYCSRGYLT  126
                M ++       ++ + + + + ++S  +ARE+    D  F  R   +  E      L
Sbjct  57   AFGAMADVYGPAFMTKIGSRNALVISSEEVAREVYTVHDK-FLRRQDMIACEILGYDGLL  115

Query  127  VAVEPQGEQWKKMRRVVASHVTSKKSFQMMLQKRTEEADNLVRYINNRSVKNRGNAF---  183
              V PQG  W+++R++V S + S      +  +R  E D   R +  R  ++ G
Sbjct  116  PLVSPQGAYWREIRKIVISQLMSASVVDTLKGRRAGEVDVAFRDLYVRWEQHNGRPHQKG  175

Query  184  VVIDLRLAVRQYSGNVARKMMFGIRHFGKGSEDGSGPGLEEIEHVESL-FTVLTHLYAFA  242
            V++D++      + N+   M+ G R++G      +G    E   +  L    + +   ++
Sbjct  176  VLVDMKPEFINVATNMTVMMVAGKRYYGNSPNCEAG----EARRIGKLILEAVQNFGRYS  231

Query  243  LSDYVPWLRFLDLEGHEKVVSNAMRNVSKYNDPFVDERLMQWRNGKMKEPQDFLDMFIIA  302
            +SD++P+L +L  E  EK +   M+ +++  D   +  + + +N + +  +D+LDM + +
Sbjct  232  ISDFIPYLGWL--EWKEKKI---MKRMAQELDCVFESWIEEHKNKRGESEKDYLDMVLES  286

Query  303  KDTDGKPTLSDEEIKAQVTELMLATVDNPSNAA--EWGMAEMINEPSIMQKAVEEIDRVV  360
             +       SD    A+   L +A   + +  A   W ++ ++N   +++KA EE+DR +
Sbjct  287  IEQHKILGSSDAHKTAKAVCLNMAISGSDAVVAILVWAVSLLVNNQHVLRKAQEELDRTI  346

Query  361  GKDRLVIESDLPNLNYVKACVKEAFRLHPVAPFNLPHMSTTDTVV-DGYF-IPKGSHVLI  418
            G  R+V ESDL +L Y++A VKE FRL+P APF     +T D V+ +G F IP G+HV++
Sbjct  347  GNQRVVEESDLKDLVYLQAIVKETFRLYPPAPFVAYRETTEDFVIANGNFHIPAGTHVMV  406

Query  419  SRMGIGRNPSVWDKPHKFDPERHLSTNTCVDL-NESDLNIISFSAGRRGCMGVDIGSAMT  477
            +   + R+P++W  P +F+PER L+++  VD+  + +  +  F  GRR C    +G+ M
Sbjct  407  NEWKVQRDPTIWPNPEEFEPERFLTSHKEVDVGGKMNHKLFPFGLGRRACPASLLGTKMV  466

Query  478  YMLLARLIQGFTWLPVPGKNKIDISESKNDL-FMAKPLYAVATPRLAPHVY  527
              +LAR +  F  +  P    +D++E  N +   A PL    TPRL   +Y
Sbjct  467  QYILARFLHSFD-VANPSNQNVDMTEDNNLVNLKATPLEVFITPRLHESLY  516
```

```
>XP_009125667.1 PREDICTED: 3,9-dihydroxypterocarpan 6A-monooxygenase [Brassica
rapa]
Length=506

 Score = 198 bits (503),  Expect = 8e-57, Method: Compositional matrix adjust.
 Identities = 154/494 (31%), Positives = 244/494 (49%), Gaps = 45/494 (9%)

Query  38   LSLPPGPKSWPLIGNLPEILGRNKPVFRWIHSLMKELNTDIACIRLANTHVIP---VTSP  94
            LSLPP P + P+IG++  +LG   P+    H  + +L+T    +       IP   V+S
Sbjct  33   LSLPPSPTALPIIGHI-HLLG---PI---AHQALHKLSTHYGPLMYLFIGSIPNVIVSST  85

Query  95   RIAREILKKQDSVFATRPLTMGTEYCSRGYLTVAVEPQGEQWKKMRRVVASHVTSKKSFQ  154
             +A EILK  +  F  RP     +Y + G       P G  WK M+R+    + S ++
Sbjct  86   EMANEILKSNELNFLNRPTMQNVDYLTYGSADFFSAPYGLHWKFMKRICMMELFSSRAID  145

Query  155  MMLQKRTEEADNL-VRYINNRSVKNRGNAFVVIDLRLAVRQYSGNVARKMMFGIRHFGKG  213
                 RTEE   L VR +    ++         +DL   +++ + ++  +MMF  R    G
Sbjct  146  RFANVRTEELRKLLVRVMKKAEIEES------VDLGEQLKELTSSIITRMMFRERRSDSG  199

Query  214  SEDGSGPGLEEIEHVESLFTVLTHLYAFALSDYVPWLRFLDLEGHEKVVSNAMRNVSKYN  273
              +     + E+  +   F V         S+   +LR LDL+G +K + NA     +Y+
Sbjct  200  RREEVIKMVVELNELAGFFNV---------SETFWFLRRLDLQGIKKRLKNAR---ERYD  247

Query  274  DPFVDERLMQWRNGKMKEP----QDFLDMFI-IAKDTDGKPTLSDEEIKAQVTELMLATV  328
               + ER+M+    +  +     +  LD+ + I +D + +  L+ E IK  +  +
Sbjct  248  --VIIERIMKEHESRNHKDAGGVRSMLDILLDIYEDKNSEIKLTRENIKGFIMNIYGGGT  305

Query  329  DNPSNAAEWGMAEMINEPSIMQKAVEEIDRVVGKDRLVIESDLPNLNYVKACVKEAFRLH  388
            D  +   EW ++E+IN P IM+KA +EI++VVG  RLV ESDL NL+Y++A VKE  RLH
Sbjct  306  DTSAITVEWALSELINNPEIMKKAQQEIEQVVGDKRLVQESDLCNLSYIQAVVKETLRLH  365

Query  389  PVAPFNLPHMSTTDTVVDGYFIPKGSHVLISRMGIGRNPSVWDKPHKFDPERHLSTNTCV  448
            P  P      S  +  V GY IP  + V+++   IGR+P+ W+ P +F PER   +   V
Sbjct  366  PGGPI-FVRESNEECAVAGYRIPAKTRVIVNVWAIGRDPNQWEDPLEFKPERFEGSEWKV  424

Query  449  DLNESDLNIISFSAGRRGCMGVDIGSAMTYMLLARLIQGFTWLPVPGKNKIDISE-SKND  507
             ++E    ++SF AGRR C G  +      ++LA +IQ F  L V G  ++++SE S +
Sbjct  425  -MSE---KMVSFGAGRRSCPGEKMVFRFVPLVLAAVIQCFE-LKVKG--RVEMSEGSGSS  477

Query  508  LFMAKPLYAVATPR  521
            L  A PL  V   R
Sbjct  478  LPRATPLVCVPVAR  491


>XP_009151334.1 PREDICTED: cytochrome P450 71A23-like [Brassica rapa]
Length=485

 Score = 197 bits (500),  Expect = 1e-56, Method: Compositional matrix adjust.
 Identities = 136/486 (28%), Positives = 225/486 (46%), Gaps = 40/486 (8%)

Query  42   PGPKSWPLIGNLPEILGRNKPVFRWIHSLMKELNTDIACIRLANTHVIPVTSPRIAREIL  101
            P P   PLIGN+ ++   N P  R + SL +    D+  +       V+  +S   AR++L
Sbjct  31   PSPPRLPLIGNMHQL--GNHP-HRSLLSLSQRYG-DLMLLHFGTVPVLVASSADAARDVL  86

Query  102  KKQDSVFATRPLTMGTEYCSRGYLTVAVEPQGEQWKKMRRVVASHVTSKKSFQMMLQKRT  161
            K  D VFA+RP +   +    G   +A  P GE W++M+ V   H+ S K  +   + R
Sbjct  87   KTHDRVFASRPHSKIYDKLLYGSSNMASAPYGEYWRQMKSVSVIHLLSNKMVRTFREVRQ  146

Query  162  EEADNLVRYINNRSVKNRGNAFVVIDLRLAVRQYSGNVARKMMFGIRHFGKGSEDGSGPG  221
            EE   ++  +  +S      + +   L       + +V  ++  G ++       G+G
Sbjct  147  EEISLMMETVRKQSSSPMNISKITTTL-------TNDVICRVALGQKY-------GAGTD  192

Query  222  LEEIEHVESLFTVLTHLYAFALSDYVPWLRFLD----LEGHEKVVSNAMRNVSKYNDPFV  277
             +E+     +  ++  L  F    YVP L ++D    LEG  +  +N    +
Sbjct  193  FKEL-----IDRLMRQLGTFTFGTYVPSLAWIDWICGLEGKLEKTANDFDKLL-------  240

Query  278  DERLMQWRNGKMKEPQDFLDMFI-IAKDTDGKPTLSDEEIKAQVTELMLATVDNPSNAAE  336
             E+++Q       +  DF D+ + + + +D      +S   IKA + +  +   D  S   E
Sbjct  241  -EKIVQDHEDGAGDKADFADVLLGVQRDKSVGFEVSRMSIKAIILDAFVGGTDTSSTLLE  299
```

```
Query  337  WGMAEMINEPSIMQKAVEEIDRVVGKDRLVIESDLPNLNYVKACVKEAFRLHPVAPFNLP  396
            W M E++N P  +++  EE+  V      V E D+ +++Y++A +KE  RLHP  P  +P
Sbjct  300  WEMQELLNHPKCLKRLQEEVRTVCKGKSSVSEDDIQDMDYLRAVIKETLRLHPPVPLMVP  359

Query  397  HMSTTDTVVDGYFIPKGSHVLISRMGIGRNPSVWD-KPHKFDPERHLSTNTCVDLNESDL  455
            H+ST D  + G+ IP G+ V+I+   +GR  + W     +F PERHL  N+  D    D
Sbjct  360  HISTEDANLRGHHIPAGTQVMINLFAVGREVATWGPDADEFKPERHL--NSSADFRGQDF  417

Query  456  NIISFSAGRRGCMGVDIGSAMTYMLLARLIQGFTWLPVPGKNKIDISESKNDLF-MAKPL  514
             +I F AGRR C G      +  + LA L+ GF W     + K D+ ES   +     PL
Sbjct  418  ELIPFGAGRRMCPGTSFAVVLNEVALANLMLGFDWKSTEDQTKTDVPESIGAVIRRMNPL  477

Query  515  YAVATP  520
            Y +A+P
Sbjct  478  YVIASP  483


>XP_009126851.1 PREDICTED: cytochrome P450 81F1-like [Brassica rapa]
Length=493

 Score = 197 bits (500),  Expect = 1e-56, Method: Compositional matrix adjust.
 Identities = 148/518 (29%), Positives = 245/518 (47%), Gaps = 40/518 (8%)

Query  8    LAFIIGLLLLALTMKRKEKKKTMLISPTRNLSLPPGPKSWPLIGNLPEILGRNKPVFRWI  67
            + F++  +LL L  K       ++ S T++ +LPPGP  +P++GNL  +    KP    +
Sbjct  4    ILFLLPFVLLILAYK------FLISSKTQSFNLPPGPTPFPIVGNLHLV----KPPVHRL  53

Query  68   HSLMKELNTDIACIRLANTHVIPVTSPRIAREILK-KQDSVFATRPLTMGTEYCSRGYLT  126
                 E   DI  +R  +  V+ ++S  + +E      D +   RP  +  +Y +  Y T
Sbjct  54   FRRFAEKYGDIFSLRYGSRQVVVISSLPLVKECFTGDNDVILTNRPHFLTAKYVAYDYTT  113

Query  127  VAVEPQGEQWKKMRRVVASHVTSKKSFQMMLQKRTEEADNLVRYINNRSVKNRGNAFVVI  186
            +   P G+ W+ +RR+ +  + S       L  R++E   L+  ++ R    R     V+
Sbjct  114  IGTAPYGDHWRNLRRICSLEILSSNRLTGFLSVRSDEIRRLLTKLS-RDYNGR-----VV  167

Query  187  DLRLAVRQYSGNVARKMMFGIRHFGKGSEDGSGPGLEEIEHVESLFTVLT-HLYAFALSD  245
            +L   +   + N   +M+ G R++G    +      EE    + L T +  + A    D
Sbjct  168  ELEPLLADLTFNNIVRMVTGRRYYGDQVHNK-----EEANLFKKLVTQINDNSGASHPGD  222

Query  246  YVPWLRFLDLEGHEKVVSNAMRNVSKYNDPFVDERLMQWRNGKMKEPQDFLDMFIIAKDT  305
            Y+P L+       +KV     + + +  D F+   L   R  +  E    L   +  +
Sbjct  223  YLPILKVFGHSYQKKV-----KALGEAMDTFLQRLLDDCR--RDGESNTMLSHLLSLQHE  275

Query  306  DGKPTLSDEEIKAQVTELMLATVDNPSNAAEWGMAEMINEPSIMQKAVEEIDRVVGKDRL  365
              K   SD  IK  +  +MLA  D  +   EW MA ++  P +++KA  EID  +G++RL
Sbjct  276  QPK-YYSDVIIKGLMLSMMLAGTDTAAVTLEWAMANLLKNPEVLKKAKAEIDDKIGQERL  334

Query  366  VIESDLPNLNYVKACVKEAFRLHPVAPFNLPHMSTTDTVVDGYFIPKGSHVLISRMGIGR  425
            V E D+ NL Y++  V E FRL P AP  +P   + D  + GY +P+G+ VL++   I R
Sbjct  335  VDEPDIVNLPYLQNIVLETFRLCPAAPLLVPRSPSEDIKIGGYDMPRGTIVLVNSWAIHR  394

Query  426  NPSVWDKPHKFDPERHLSTNTCVDLNESDLNIISFSAGRRGCMGVDIGSAMTYMLLARLI  485
            +P +WD+P +F PER        + N+    ++ F  GRR C G  +G  M  + L  LI
Sbjct  395  DPKLWDEPERFMPER-FEDKAAANANK----LMMFGNGRRTCPGAALGQRMVSLALGSLI  449

Query  486  QGFTWLPVPGKNKIDISESKNDLFMAK--PLYAVATPR  521
            Q F W  V G++ ID++E+   + M K  PL AV   R
Sbjct  450  QCFDWEKVNGED-IDMTENPG-MAMRKLVPLRAVCHQR  485


>XP_009136105.1 PREDICTED: cytochrome P450 82G1 [Brassica rapa]
Length=513

 Score = 197 bits (501),  Expect = 1e-56, Method: Compositional matrix adjust.
 Identities = 131/454 (29%), Positives = 222/454 (49%), Gaps = 20/454 (4%)

Query  78   IACIRLANTHVIPVTSPRIAREILKKQDSVFATRPLTMGTEYCSRGYLTVAVEPQGEQWK  137
            I  ++L    ++  + P I ++     D V ATRP      Y       +A+ P G+ W+
```

```
Sbjct  73   IFSLKLGFYRLVVTSDPEILKDCFTTNDLVLATRPNIAFGRYVGYNNAALALAPYGDYWR  132

Query  138  KMRRVVASHVTSKKSFQMMLQKRTEEADNLVRYINNRSVKNRGNAFVVIDLRLAVRQYSG  197
            ++R++   H+ S +S +M+   R  E +  ++++   S    G + V ID+      +
Sbjct  133  ELRKITTVHLFSNQSVEMLGHIRYAEVNEFLKHLYEGS---DGTSTVKIDMSFEFLTF--  187

Query  198  NVARKMMFGIR-HFGKGSEDGSGPGLEEIEHVESLFTVLTHLYAFALSDYVPWLRFLDLE  256
            N+  + M G R  FGK          EE  + E+L         F + D +PWL +LD
Sbjct  188  NIILRKMVGKRIGFGKVKS-------EEWRYKEALKRSEYLAAVFMIGDVIPWLGWLDFT  240

Query  257  GHEKVVSNAMRNVSKYNDPFVDERLMQWRNGKMKEPQDFLDMF--IIAKDTDGKPTLSDE  314
               ++ S   + +      +++E L +    +  + +  +D+   I+ +D      + D
Sbjct  241  KIAQMKST-FKELDSVITKWLEEHLEKRSRKEENQEKTIMDLLLDILPEDVVICGHVRDV  299

Query  315  EIKAQVTELMLATVDNPSNAAEWGMAEMINEPSIMQKAVEEIDRVVGKDRLVIESDLPNL  374
             +KA +  L L   D+ S    W ++ ++N PS ++ A EEID  VGK R V ESD+ NL
Sbjct  300  IVKATILVLTLTGSDSTSITLTWAVSLLLNNPSTLKAAQEEIDNSVGKGRWVEESDIRNL  359

Query  375  NYVKACVKEAFRLHPVAPFNLPHMSTTDTVVDGYFIPKGSHVLISRMGIGRNPSVWDKPH  434
             Y++A VKE  RL+P AP      +  D ++  Y + +GS +L++   + R+P +W  P
Sbjct  360  KYLQAIVKETHRLYPPAPLTGIREAREDCLLGEYHVKRGSRLLVNIWKLHRDPKIWHDPE  419

Query  435  KFDPERHLSTNTCVDLNESDLNIISFSAGRRGCMGVDIGSAMTYMLLARLIQGFTWLPVP  494
             F PER +   +  +  +SD   I FS+GRR C G+++G  + +++LARLIQGF  L
Sbjct  420  TFKPERFMEEKSLCE--KSDFEFIPFSSGRRSCPGMNLGLRVVHLVLARLIQGFE-LHKA  476

Query  495  GKNKIDISESKN-DLFMAKPLYAVATPRLAPHVY  527
                +D++E     L    P+  VA PRL P +Y
Sbjct  477  SDEPLDMAEGPGLALPKINPVEVVAMPRLEPELY  510


>XP_009148545.1 PREDICTED: cytochrome P450 71B7-like [Brassica rapa]
Length=507

 Score = 197 bits (501),  Expect = 2e-56, Method: Compositional matrix adjust.
 Identities = 143/519 (28%), Positives = 249/519 (48%), Gaps = 48/519 (9%)

Query  6    PMLAFIIGLLLLALTMKRKEKKKTMLISPTRNLSLPPGPKSWPLIGNLPEILGRNKPVFR  65
            P+  F++   ++A   K  + K            LPPGPK+ P+IGNL  + G+    F
Sbjct  10   PLAIFVVSFFIIAKKHKPSKWK------------LPPGPKTLPIIGNLHNLKGQPHTCF-  56

Query  66   WIHSLMKELNTDIACIRLANTHVIPVTSPRIAREILKKQDSVFATRPLTMGTEYCSRGYL  125
               + + +    +  +R  +  V+ ++S   A E LK QD    +RP T+ T   S  +
Sbjct  57   ---TNLSKTYGPVMLLRFGSVPVVVISSREGAEEALKTQDLECCSRPETVATRMISYNFK  113

Query  126  TVAVEPQGEQWKKMRRVVASHVTSKKSFQMMLQKRTEEADNLVRYINNRSVKNRGNAFVV  185
             +   P GE+WK +R++V   + + K FQ     R EE + LV+ +   ++
Sbjct  114  DIGFAPYGEEWKALRKLVVVELLNMKKFQSFGYIREEENNVLVKKLTEAALTRSP-----  168

Query  186  IDLRLAVRQYSGNVARKMMFGIR-HFGKGSEDGSGPGLEEIEHVESLFTVLTHLYAFALS  244
            ++L+  +     ++  ++ FGI  H  +  ++ +   L  +   E L          A S
Sbjct  169  VNLKKTLFALVASIICRLAFGIDIHKCEFVDEDNAADL--VHKFELLVD------GIAFS  220

Query  245  DYVP---WLRFLD-LEGHEKVVSNAMRNVSKYNDPFVDERLMQWRNGKMKEPQDFLDMFI  300
            D+ P   W+  LD + G  K ++N    +  +    +D  L   R   + +  D +D+ +
Sbjct  221  DFFPGVGWI--LDRVSGQNKTLNNVFSELDTFFQNILDAHLKPGRT--VSDNPDVVDVMV  276

Query  301  ---IAKDTDGKP-TLSDEEIKAQVTELMLATVDNPSNAAEWGMAEMINEPSIMQKAVEEI  356
                 ++ DG    L+ +  K  ++++ LA V+  +    W M E+I  P+IM+K   EI
Sbjct  277  GLMKKQEQDGDSFKLTTDHFKGIISDIFLAGVNTSAITMIWAMTELIRNPNIMKKVQHEI  336

Query  357  DRVVG--KDRLVIESDLPNLNYVKACVKEAFRLHPVAPFNLPHMSTTDTVVDGYFIPKGS  414
               +G  K+RL  + DL +L+Y+K  +KE FRLHP AP  LP  + +D  + GY IPK S
Sbjct  337  RTTLGDNKERLTAD-DLNHLHYLKHVIKETFRLHPAAPLLLPRETMSDIKIQGYDIPKKS  395

Query  415  HVLISRMGIGRNPSVWDKPHKFDPERHLSTNTCVDLNESDLNIISFSAGRRGCMGVDIGS  474
             ++I+   I R+P +W  P +F+P+R +  ++ VD    +  ++ F +GRR C G+++G
Sbjct  396  QMMINVYSIARDPEIWSNPDEFNPDRFI--DSSVDYKGLNFELLPFGSGRRICPGMNMGI  453
```

```
Query  475  AMTYMLLARLIQGFTWLPVPGKNKIDIS-ESKNDLFMAK  512
            A   + L  L+  F W    GK   D+  E    L ++K
Sbjct  454  ATVELGLLNLLYFFDWAVPEGKTIKDMDLEETGSLIISK  492
```

```
>XP_009120246.1 PREDICTED: cytochrome P450 81F1 [Brassica rapa]
Length=491

 Score = 196 bits (497),  Expect = 3e-56, Method: Compositional matrix adjust.
 Identities = 145/491 (30%), Positives = 233/491 (47%), Gaps = 34/491 (7%)

Query  35   TRNLSLPPGPKSWPLIGNLPEILGRNKPVFRWIHSLMKELNTDIACIRLANTHVIPVTSP  94
            +++ +LPPGP  +P++GNL  +    KP    +     E   DI  +R  +  V+ ++S
Sbjct  23   SKSFNLPPGPTPFPIVGNLHLV----KPPVHRLFRRFAEKYGDIFSLRYGSRQVVVISSL  78

Query  95   RIAREILKKQDSVFAT-RPLTMGTEYCSRGYLTVAVEPQGEQWKKMRRVVASHVTSKKSF  153
             + RE    Q+ V  T RP  +  +Y +  Y TV     G+ W+ +RR+ +  + S
Sbjct  79   PLVRECFTGQNDVILTNRPHFLTAKYVAYDYTTVGTAAYGDHWRNLRRICSLEILSSNRL  138

Query  154  QMMLQKRTEEADNLVRYINNRSVKNRGNAFVVIDLRLAVRQYSGNVARKMMFGIRHFGKG  213
               L  R +E   L+  ++      R     V++L   +   + N   +M+ G R++G
Sbjct  139  TGFLSVRKDEIRRLLTKLS------RDYNGQVVELEPLLADLTFNNIVRMVTGRRYYGDQ  192

Query  214  SEDGSGPGLEEIEHVESLFTVLT-HLYAFALSDYVPWLRFLDLEGHEKVVSNAMRNVSKY  272
              +      EE    + L T +  +  A    DY+P L+     G+EK V    + + +
Sbjct  193  VHNK-----EEANLFKKLVTQINDNSGASHPGDYLPILKVFG-HGYEKKV----KALGEA  242

Query  273  NDPFVDERLMQWRNGKMKEPQDFLDMFIIAKDTDGKPTLSDEEIKAQVTELMLATVDNPS  332
             D F+   L   R  +  E    L   +++   D     SD  IK  +  +MLA  D  +
Sbjct  243  MDTFLQRLLDDCR--RDGESNTMLS-HLLSLQVDQPKYYSDVIIKGLMLSMMLAGTDTAA  299

Query  333  NAAEWGMAEMINEPSIMQKAVEEIDRVVGKDRLVIESDLPNLNYVKACVKEAFRLHPVAP  392
               EW MA ++  P +++KA  EID  +G +RLV E D+ NL Y++  V E FRL P AP
Sbjct  300  VTLEWAMASLLKSPEVLKKAKAEIDDKIGHERLVDEPDILNLPYLQNIVSETFRLCPAAP  359

Query  393  FNLPHMSTTDTVVDGYFIPKGSHVLISRMGIGRNPSVWDKPHKFDPERHLSTNTCVDLNE  452
              +P   + D  + GY IP+G+ VL++   I R+P +WD+P +F PER
Sbjct  360  LLVPRSPSEDLKIGGYDIPRGTIVLVNSWAIHRDPRLWDEPERFMPERFEDKEAA-----  414

Query  453  SDLNIISFSAGRRGCMGVDIGSAMTYMLLARLIQGFTWLPVPGKNKIDISESKNDLFMAK  512
            ++  ++ F  GRR C G  +G  M  + L  LIQ F W  V G+ +ID++E+   + M K
Sbjct  415  NNNKLMMFGNGRRTCPGAALGQRMVSLALGSLIQCFDWEKVNGE-EIDMTENSG-MAMRK  472

Query  513  --PLYAVATPR  521
              PL AV   R
Sbjct  473  LVPLRAVCHQR  483
```

```
>XP_009150729.1 PREDICTED: cytochrome P450 71B7-like [Brassica rapa]
Length=502

 Score = 196 bits (497),  Expect = 4e-56, Method: Compositional matrix adjust.
 Identities = 144/505 (29%), Positives = 236/505 (47%), Gaps = 52/505 (10%)

Query  40   LPPGPKSWPLIGNLPEILGRNKPVFRWIHSLMKELNTDIACIRLANTHVIP---VTSPRI  96
            LPPGP+  P+IGNL  + G         H+  + L+     + L     +P   ++S
Sbjct  30   LPPGPQKLPIIGNLHNLNG-------LAHTCFQNLSQKFGPVMLLCLGYVPTVVISSREG  82

Query  97   AREILKKQDSVFATRPLTMGTEYCSRGYLTVAVEPQGEQWKKMRRVVASHVTSKKSFQMM  156
            A E LK  D    +RP T+ T   S  +  +   P GE+WK +R++V   + + K  +
Sbjct  83   AEEALKTHDLECCSRPETVATRMLSYNFKDIGFAPYGEEWKSLRKLVVMELLNAKKLKSF  142

Query  157  LQKRTEEADNLVRYINNRSVKNRGNAFVVIDLRLAVRQYSGNVARKMMFGIRHFGKGSED  216
               R EE D LV+ +  R     G+    ++L  A+     +V  ++ FGI
Sbjct  143  KYIREEENDILVKKL--RECALTGSP---VNLTKALFTLVASVVCRLAFGI---------  188

Query  217  GSGPGLEEIEHV-ESLFTVLTHLY-----AFALSDYVPWLR-FLD-LEGHEKVVSNAMRN  268
                 + + E + E     L H +      FA SD+ P +  F+D + G  K ++N
Sbjct  189  ----DIHKCEFIDEDNVADLVHKFELLIDGFAFSDFFPGVGWFIDQISGQNKTLNNVFSE  244
```

```
Query  269  VSKYNDPFVDERLMQWRNGKMKEPQDFLDMFIIAKDTDGKP----TLSDEEIKAQVTELM  324
            +  +    +DE L     G++ E  D +D+ +   +  GK      L+ +  K  ++++
Sbjct  245  LDTFFQKVLDEHLKP--GGRVSESPDVVDVMVDLMEKQGKDGDSFKLTTDHFKGIISDIF  302

Query  325  LATVDNPSNAAEWGMAEMINEPSIMQKAVEEIDRVVG-KDRLVIESDLPNLNYVKACVKE  383
            LA V+  +    W M E+I  P +M+K  +EI   +G K   + E DL  L+Y K  VKE
Sbjct  303  LAGVNTSAITLAWAMTEVIRNPRVMKKVQDEIRTTLGVKKEKLTEDDLSQLHYFKLVVKE  362

Query  384  AFRLHPVAPFNLPHMSTTDTVVDGYFIPKGSHVLISRMGIGRNPSVWDKPHKFDPERHLS  443
             FRLHP AP  LP  + +D  + GY IP  + ++I+   I R+P +W  P +F+P+R L
Sbjct  363  TFRLHPAAPLLLPRETMSDIKIQGYDIPAKTQMIINVYLIARDPKIWTNPDEFNPDRFLE  422

Query  444  TNTCVDLNESDLNIISFSAGRRGCMGVDIGSAMTYMLLARLIQGFTWLPVPGK--NKIDI  501
            ++  +D    +  ++ F +GRR C G+ +G A   M L  L+  F W    GK  N +D+
Sbjct  423  SS--IDYKGLNYELLPFGSGRRICPGMMMGIANVEMGLLNLLYFFDWGLPEGKTVNDMDL  480

Query  502  SESKNDLFMAKPLYAVATPRLAPHV  526
             E+ + +   K     AT  L P +
Sbjct  481  EETGSIIVSKK-----ATLELVPFI  500
```

>XP_009132142.1 PREDICTED: cytochrome P450 81F1-like [Brassica rapa]
Length=493

Score = 195 bits (495), Expect = 8e-56, Method: Compositional matrix adjust.
Identities = 147/498 (30%), Positives = 239/498 (48%), Gaps = 34/498 (7%)

```
Query  28   KTMLISPTRNLSLPPGPKSWPLIGNLPEILGRNKPVFRWIHSLMKELNTDIACIRLANTH  87
            K +  + T+  +LPPGP  +P++GNL  +     PV R   +   +   +I  +R  +
Sbjct  18   KFLFSTKTKRYNLPPGPTPFPIVGNLHLV---KPPVHRLFRNFAAKYG-EIFSLRYGSRQ  73

Query  88   VIPVTSPRIAREILKKQDSVFAT-RPLTMGTEYCSRGYLTVAVEPQGEQWKKMRRVVASH  146
            V+ ++S  + RE    Q+ V  T RP  +  +Y +    T+     G+ W+ +RR+ +
Sbjct  74   VVVISSLPLVRECFMGQNDVILTNRPHFLTAKYVAYDDTTIGTAAYGDHWRNLRRICSLE  133

Query  147  VTSKKSFQMMLQKRTEEADNLVRYINNRSVKNRGNAFVVIDLRLAVRQYSGNVARKMMFG  206
            + S      +L  R +E   L+  ++      R     V++L   +   + N   +M+ G
Sbjct  134  ILSSHRLTGLLSVRRDEIQRLLTRLS------RDYNGHVVELEPLLADLTFNNIVRMVTG  187

Query  207  IRHFGKGSEDGSGPGLEEIEHVESLFTVLT-HLYAFALSDYVPWLRFLDLEGHEKVVSNA  265
             R++G    +       EE    + L T +  +  A    DY+P L+     G+EK V
Sbjct  188  RRYYGDQVHNE-----EEANLFKKLVTEINDNSGASHPGDYLPILKVFG-HGYEKKV---  238

Query  266  MRNVSKYNDPFVDERLMQWRNGKMKEPQDFLDMFIIAKDTDGKPTLSDEEIKAQVTELML  325
             + + +  D F+   L   R  +  E    L   +++   D     SD  IK  +  +ML
Sbjct  239  -KALGEAMDTFLQRLLDDCR--RDGESNTMLS-HLLSLQLDQPMYYSDVIIKGLMLSMML  294

Query  326  ATVDNPSNAAEWGMAEMINEPSIMQKAVEEIDRVVGKDRLVIESDLPNLNYVKACVKEAF  385
            A  D  +   EW MA ++N P +++KA  EID  +G++RLV E D+ NL Y++  V E F
Sbjct  295  AGTDTAAVTLEWAMANLLNNPEVLKKAKSEIDVKIGQERLVDEPDIVNLPYLQNIVSETF  354

Query  386  RLHPVAPFNLPHMSTTDTVVDGYFIPKGSHVLISRMGIGRNPSVWDKPHKFDPERHLSTN  445
            RL P AP  +P   + D  + GY +P+G+ VL++   I R+P +WD+P KF PER
Sbjct  355  RLCPAAPLLVPRSPSEDLKIGGYDVPRGAIVLVNSWAIHRDPKLWDEPEKFMPER-FEEK  413

Query  446  TCVDLNESDLNIISFSAGRRGCMGVDIGSAMTYMLLARLIQGFTWLPVPGKNKIDISESK  505
               + N+    ++ F  GRR C G  +G  M  + L  LIQ F W  V G+ KID++E+
Sbjct  414  EAANTNK----LMMFGNGRRTCPGAALGQRMVSLALGSLIQCFDWEKVNGE-KIDMTENP  468

Query  506  NDLFMAK--PLYAVATPR  521
              + M K  PL AV   R
Sbjct  469  G-MAMRKLVPLRAVCYQR  485
```

>XP_009152069.1 PREDICTED: cytochrome P450 71B23 [Brassica rapa]
Length=501

Score = 195 bits (495), Expect = 8e-56, Method: Compositional matrix adjust.

```
 Identities = 139/493 (28%), Positives = 234/493 (47%), Gaps = 28/493 (6%)

Query  35   TRNLSLPPGPKSWPLIGNLPEILGRNKPVFRWIHSLMKELNTDIACIRLANTHVIPVTSP  94
            T  L+ PPGP+  P+IGNL  + G      + + +L K +   +  +      ++ ++S
Sbjct  26   TSKLNHPPGPRKLPIIGNLHNLEGLPH---KCLQNLSK-IYGPVMKLHFGFVPIVIISSK  81

Query  95   RIAREILKKQDSVFATRPLTMGTEYCSRGYLTVAVEPQGEQWKKMRRVVASHVTSKKSFQ  154
            + A E+LK  D    +RP T+ T+  S  +  +   P GE+W+ +R++    + + K
Sbjct  82   QAAEEVLKTHDLDCCSRPETVATKKISYNFKDIGFAPYGEEWRSLRKLAVMELLNLKKLN  141

Query  155  MMLQKRTEEADNLVRYINNRSVKNRGNAFVVIDLRLAVRQYSGNVARKMMFGIRHFGKGS  214
                 R EE D  V+ ++  SVK+       ++L  A+     ++  ++ FG       S
Sbjct  142  SFRYIREEENDLFVKKLSEASVKH-----FPVNLTKALFTLIASIVCRLAFGQNL--HES  194

Query  215  EDGSGPGLEEIEHVESLFTVLTHLYAFALSDYVPWLRFLD-LEGHEKVVSNAMRNVSKYN  273
            E     G+EE+                FA S+Y P    +D + G  K +      +  +
Sbjct  195  EFIDEDGMEELASRSEKLQ-----GEFAFSNYFPGGWIVDRITGQSKNLEGLFSELDGFF  249

Query  274  DPFVDERLMQWRNGKMKEPQDFLDMFII---AKDTDGKPTLSDEEIKAQVTELMLATVDN  330
            +  +DE L   R   + E  D +D+ I     +  DG   L+ + IK  ++++ LA V+
Sbjct  250  NQVLDEHLKPGR--IVLESPDVVDVMIDMMNKQGEDGHFQLTTDHIKGVISDIFLAGVNT  307

Query  331  PSNAAEWGMAEMINEPSIMQKAVEEIDRVVG-KDRLVIESDLPNLNYVKACVKEAFRLHP  389
             +    W M E+I  PS+M+K  EE+  V+G K   + E DL  L+Y K  +KE FRLHP
Sbjct  308  SATTILWAMTELIKNPSVMKKVQEEVRTVLGEKKEKITEQDLNQLSYFKLVIKETFRLHP  367

Query  390  VAPFNLPHMSTTDTVVDGYFIPKGSHVLISRMGIGRNPSVWDKPHKFDPERHLSTNTCVD  449
             AP  +P  + +   + GY I K + ++I+   I R+P  W+ P +F PER    ++C+D
Sbjct  368  TAPLLVPRETMSPIKIQGYDILKKNQIMINVYAIARDPKTWENPDEFKPERF--ADSCID  425

Query  450  LNESDLNIISFSAGRRGCMGVDIGSAMTYMLLARLIQGFTWLPVPGKNKIDIS-ESKNDL  508
                +  ++ F +GRR C G+ +G AM  + L  L+  F W+   G    DI  + +  L
Sbjct  426  YRGLNFELLPFGSGRRICPGMTMGIAMVELGLLNLLYFFDWVLPEGTTVKDIDMDEEGAL  485

Query  509  FMAK--PLYAVAT  519
             + K  PL  V T
Sbjct  486  IIGKKVPLELVPT  498


>XP_009136992.1 PREDICTED: cytochrome P450 81D11 [Brassica rapa]
Length=492

 Score = 194 bits (494),  Expect = 9e-56, Method: Compositional matrix adjust.
 Identities = 139/500 (28%), Positives = 232/500 (46%), Gaps = 44/500 (9%)

Query  28   KTMLISPTRNLSLPPGPK-SWPLIGNLPEILGRNKPVFRWIHSLMKEL-NTDIACIRLAN  85
            K +L    R L+LPP P  S PLIG+L  +     P+ R   SL   + N  I  +RL N
Sbjct  19   KILLTRSKRKLNLPPSPAISLPLIGHLHLL---KPPLHRSFRSLSISIGNAPIFQLRLGN  75

Query  86   THVIPVTSPRIAREILKKQDSVFATRPLTMGTEYCSRGYLTVAVEPQGEQWKKMRRVVAS  145
              V  ++S  +A E     D V A RP  + ++Y        +     G+ W+ +RR+ A
Sbjct  76   RLVYVISSRSMAEECFTGNDVVLANRPKFIVSKYVGYNATHLIAASYGDHWRNLRRIAAV  135

Query  146  HVTSKKSFQMMLQKRTEEADNLVRYINNRSVKNRGNAFVVIDLRLAVRQYSGNVARKMMF  205
             + S +     L  R +E   L+  ++  S+     + +V ++++  +   + N   +M
Sbjct  136  ELFSTQRLNAFLYIRKDEIQRLISRLSRDSL----HGYVEVEMKSLLANLASNNIIRMAA  191

Query  206  GIRHFGKGSEDGSGPGLEEIEHVESLFT-VLTHLYAFALSDYVPWLRFLDLEGHEKVVSN  264
            G R++G+ ++        E + V  L + V+T   A   +DY+  +R+     +EK + N
Sbjct  192  GKRYYGEEND--------EAKFVRQLVSEVVTSAGAGNPADYLSIVRWF--TNYEKRIKN  241

Query  265  AMRNVSKYNDPFVDERLMQWRNGKMKEPQDFLDMFIIAKDTDGKPTLSDEEIKAQVTELM  324
                   +    VDE+      G      +  +D  +  ++T      +D+ IK  +  L
Sbjct  242  LGNRFDAFLQRIVDEKRADKEKG-----ETMIDRLLSLQETQ-PDYYTDDIIKGLILTLT  295

Query  325  LATVDNPSNAAEWGMAEMINEPSIMQKAVEEIDRVVGKDRLVIESDLPNLNYVKACVKEA  384
            +   D  +   EW ++ ++N P +++KA  EID  +G  RLV E D+ NL Y++  V E
Sbjct  296  IGGTDTSAVTLEWALSNLLNHPEVLKKARAEIDDKIGFGRLVDEPDIANLPYLQNIVSET  355
```

```
Query  385  FRLHPVAPFNLPHMSTTDTVVDGYFIPKGSHVLISRMGIGRNPSVWDKPHKFDPERHLST  444
             RL+P  P  LPH+S+ D  V GY +P+G+ VL +   + R+P +W+ P  F PER
Sbjct  356  LRLYPAVPLLLPHVSSDDCKVAGYDVPRGTMVLTNVWAMHRDPMLWEDPELFKPERFEKE  415

Query  445  NTCVDLNESDLNIISFSAGRRGCMGVDIGSAMTYMLLARLIQGFTWLPVPGKNKIDISES  504
                 L       + F  GRR C G  +   +  ++LA L+Q F W  V G+  +D++E
Sbjct  416  GEAEKL-------LPFGMGRRACPGAGLAQRLVSLVLATLVQCFEWERV-GEELVDMTED  467

Query  505  KNDLFMAKPLYAVATPRLAP  524
            K           V  P+L P
Sbjct  468  K----------GVTLPKLVP  477
```

>XP_009138491.1 PREDICTED: cytochrome P450 81D11-like [Brassica rapa]
Length=498

Score = 194 bits (493), Expect = 2e-55, Method: Compositional matrix adjust.
Identities = 139/465 (30%), Positives = 224/465 (48%), Gaps = 28/465 (6%)

```
Query  62   PVFRWIHSLMKEL-NTDIACIRLANTHVIPVTSPRIAREILKKQDSVFATRPLTMGTEYC  120
            P+ R   S+ + L +  I  +RL N  V   +S  IA E   K D V A RP  +  ++
Sbjct  50   PIHRTFLSISQSLGDAPIFSLRLGNRLVFVNSSHSIAEECFTKNDVVLANRPNFILAKHV  109

Query  121  SRGYLTVAVEPQGEQWKKMRRVVASHVTSKKSFQMMLQKRTEEADNLVRYINNRSVKNRG  180
            +  Y TV     G+ W+ +RR+ +  + S       L  R +E    +R +  R  +N
Sbjct  110  AYDYTTVIAASYGDHWRNLRRIGSLEIFSNHRLNSFLSIRKDE----IRRLILRLSRNFS  165

Query  181  NAFVVIDLRLAVRQYSGNVARKMMFGIRHFGKGSEDGSGPGLEEIEHVESLFTVLTHLY-  239
              +  ++++  +   + N   +M+ G R++G G E+       E + V  L   +  L
Sbjct  166  QEYAKVEMKSMLSDLTFNNIIRMVAGKRYYGDGVEEDP-----EAKRVRQLIADVVALAG  220

Query  240  AFALSDYVPWLRFLDLEGHEKVVSNAMRNVSKYNDPFVDERLMQWRNGKMKEPQDFLDMF  299
            A    DY+P+LR++    +E  V      + ++    VDE+    R+ K K     +D
Sbjct  221  AGNAVDYLPFLRWV--SDYENRVMKLAGRLDEFLQGLVDEK----RDAKEK-GNTMVDHL  273

Query  300  IIAKDTDGKPTLSDEEIKAQVTELMLATVDNPSNAAEWGMAEMINEPSIMQKAVEEIDRV  359
            +  ++T      +D  IK  +  L+LA  D  +   EW ++ ++N P +++KA  EID
Sbjct  274  LSLQETQ-PDYFTDRIIKGNMVALILAGTDTSAVTLEWALSNLLNYPDVLKKARNEIDCK  332

Query  360  VGKDRLVIESDLPNLNYVKACVKEAFRLHPVAPFNLPHMSTTDTVVDGYFIPKGSHVLIS  419
            VG DRL+ ESD+ NL Y++  V E  RL+P  P  LPH+++ D  V GY +P G+ +L +
Sbjct  333  VGLDRLIDESDISNLPYLQNIVCETLRLYPSVPMLLPHVASEDCKVAGYDMPSGTILLTN  392

Query  420  RMGIGRNPSVWDKPHKFDPERHLSTNTCVDLNESDLNIISFSAGRRGCMGVDIGSAMTYM  479
               I R+P +W+ P  F PER           ES+  ++SF  GRR C G  +   +  +
Sbjct  393  AWAIHRDPQLWEDPTSFKPERFEKE------GESN-KLMSFGLGRRACPGSGLAHRLVNL  445

Query  480  LLARLIQGFTWLPVPGKNKIDISESKNDLF-MAKPLYAVATPRLA  523
             L  LIQ   W  + G+ K+D+SE K      AKPL A+   R A
Sbjct  446  TLGSLIQCLEWERI-GEEKVDMSERKGGTMPKAKPLEAMCRARTA  489
```

>XP_009116856.1 PREDICTED: cytochrome P450 78A9 [Brassica rapa]
Length=532

Score = 193 bits (490), Expect = 8e-55, Method: Compositional matrix adjust.
Identities = 141/483 (29%), Positives = 232/483 (48%), Gaps = 37/483 (8%)

```
Query  42   PGPKSWPLIGNLPEILGRNKPVFRWIHSLMKELNTD-IACIRLANTHVIPVTSPRIAREI  100
            PGP+  P +G++   L  N    R I +  ++     +    L +T VI   +P +A+EI
Sbjct  68   PGPRGLPFVGSMS--LMSNALAHRCIAAAAEKFGAKRLMAFSLGDTRVIVTCNPDVAKEI  125

Query  101  LKKQDSVFATRPLTMGTEYCSRGYLTVAVEPQGEQWKKMRRVVASHVTSKKSFQMMLQKR  160
            L     VFA RP+   + Y       +   P G  W+ +R++ ++H+ S K  +    +R
Sbjct  126  L--NSPVFADRPVK-ESAYSLMFNRAIGFAPYGVYWRTLRKIASNHLFSPKQIKRSETQR  182

Query  161  TEEADNLVRYINNRSVKNRGNAFVVIDLRLAVRQYSGNVARKMMFGIRHFGKGSEDGSGP  220
               A+ +V+ +  +S  N G  F     R  ++  S N    MM  +  FGK  E
Sbjct  183  RVIANQIVKCLEKQSSGNEGLCFA----RDLIKTASLN---SMMCSV--FGKEYE-----  228
```

```
Query  221  GLE-EIEHVESLFTVLTHLY----AFALSDYVPWLRFLDLEGHEKVVSNAMRNVSKYNDP  275
             LE E + V  L  ++   Y        +D++PWL   D +      SN +  V++
Sbjct  229  -LEHEHDEVNELRGLVEEGYDLLGTLNWTDHLPWLSEFDPQRIRSRCSNLVPKVNR----  283

Query  276  FVDERLMQWRNGKMKEPQDFLDMFIIAKDTDGKPTLSDEEIKAQVTELMLATVDNPSNAA  335
            FV+  +   R+     P DF+D+ +     DG+  LSD ++ A + E++    D  +
Sbjct  284  FVNRIISDHRDQTRDSPSDFVDVLL---SLDGQDKLSDPDMVAVLWEMIFRGTDTVAVLI  340

Query  336  EWGMAEMINEPSIMQKAVEEIDRVVGKDRLVIESDLPNLNYVKACVKEAFRLHPVAPF-N  394
            EW +A M+  P I      E+D+ VG+ R V ESD+ +L Y+ A VKE  RLHP  P  +
Sbjct  341  EWVLARMVLHPDIQSMVHNELDQNVGRSRTVEESDVASLTYLTAVVKEVLRLHPPGPLLS  400

Query  395  LPHMSTTDTVVDGYFIPKGSHVLISRMGIGRNPSVWDKPHKFDPERHLSTNTCVDLN--E  452
               ++ TDT++DG  +P G+  +++   I  +P VW+ P +F PER ++    V+ +
Sbjct  401  WARLAITDTIIDGRRVPAGTTAMVNMGAIAHDPQVWENPLEFKPERFVAKEGEVEFSVLG  460

Query  453  SDLNIISFSAGRRGCMGVDIGSAMTYMLLARLIQGFTWLPVPGKNKIDISES-KNDLFMA  511
            SDL +  F +GRR C G ++G +     +A L+  F W    G+  +D+SE  +    MA
Sbjct  461  SDLRLAPFGSGRRVCPGKNLGLSTVMYWIATLMHEFEWFAPTGEKTVDLSEKLRLSCEMA  520

Query  512  KPL  514
             PL
Sbjct  521  NPL  523


>XP_009110601.1 PREDICTED: cytochrome P450 71B2-like [Brassica rapa]
Length=502

 Score = 191 bits (486),  Expect = 2e-54, Method: Compositional matrix adjust.
 Identities = 137/491 (28%), Positives = 230/491 (47%), Gaps = 25/491 (5%)

Query  38   LSLPPGPKSWPLIGNLPEILGRNKPVFRWIHSLMKELNTDIACIRLANTHVIPVTSPRIA  97
            L+LPP P S P+IGNL    G      R+ HSL  +    +  +RL    V+ ++S   A
Sbjct  29   LNLPPSPSSLPIIGNLHHFAGCPS---RYFHSLSIKYGP-VMLLRLGFLRVVVISSSEAA  84

Query  98   REILKKQDSVFATRPLTMGTEYCSRGYLTVAVEPQGEQWKKMRRVVASHVTSKKSFQMML  157
             E+LK  D    +RP T+ +   S G+  V   P GE W++MR++V   + S K  Q
Sbjct  85   EEVLKTHDMECCSRPNTLVSGKLSYGFKDVNFAPYGEYWREMRKLVVIELFSLKKVQSFR  144

Query  158  QKRTEEADNLVRYINNRSVKNRGNAFVVIDLRLAVRQYSGNVARKMMFGIRHFGKG---S  214
              R EE+  +V+ ++  ++K        +DL       + ++  ++  G+     G
Sbjct  145  YIREEESYLMVKKVSESALKRSP-----VDLNKTFFSLTASIICRVALGLNFHESGFVID  199

Query  215  EDGSGPGLEEIEHVESLFTVLTHLYAFALSDYVPWLRFLDLEGHEKVVSNAMRNVSKYND  274
            ++     + E+  V   FT  +  +  AL  +V WL     + H+K+ +  +  +  +
Sbjct  200  QEKIEDLVTEVGEVLGTFT-FSDYFPGALGSFVDWL----FQRHKKI-NKVVEELDAFYQ  253

Query  275  PFVDERLMQWRNGKMKEPQDFLDMFIIAKDTDGKPTLSDEEIKAQVTELMLATVDNPSNA  334
              +DE L       +      LDM I  + ++    L  + +KA + ++ LA +D  +
Sbjct  254  HVIDEHLKAEGRKNLDIVSLMLDM-IDKQGSEDYFKLDMDNVKAIIMDVFLAGIDTGATT  312

Query  335  AEWGMAEMINEPSIMQKAVEEIDRVVGKDR-LVIESDLPNLNYVKACVKEAFRLHPVAPF  393
              W M E++  P++M+KA E I   +G  R  + E DL  ++Y+   +KE FRLHP  PF
Sbjct  313  MIWAMTELVRNPNVMKKAQENIRATLGHKRERITEEDLGKVDYMTFIIKETFRLHPPVPF  372

Query  394  NLPHMSTTDTVVDGYFIPKGSHVLISRMGIGRNPSVWDKPHKFDPERHLSTNTCVDLNES  453
             LP  + +   + GY IP  + + ++   IGR+P  W  P  F PER    ++ VD
Sbjct  373  LLPRETMSHVKIQGYDIPPKTQIKVNVWTIGRDPKRWTDPEDFIPERF--ADSSVDFRGQ  430

Query  454  DLNIISFSAGRRGCMGVDIGSAMTYMLLARLIQGFTWLPVPGKN--KIDISESKNDLFMA  511
               ++ F +GRR C  + +G+A   + L  L+  F W    G N  + D+ E+ N  ++
Sbjct  431  HFELLPFGSGRRICPAMAMGTATVELGLMNLLYFFDWGLPDGMNIEEFDMEEADNPTYVK  490

Query  512  K-PLYAVATPR  521
            K PL  V   R
Sbjct  491  KLPLQLVPFQR  501
```

>XP_009138745.1 PREDICTED: LOW QUALITY PROTEIN: cytochrome P450 78A9-like
[Brassica
rapa]
Length=562

Score = 192 bits (489), Expect = 2e-54, Method: Compositional matrix adjust.
Identities = 139/479 (29%), Positives = 224/479 (47%), Gaps = 28/479 (6%)

```
Query  42   PGPKSWPLIGNLPEILGRNKPVFRWIHSLMKELNTD-IACIRLANTHVIPVTSPRIAREI  100
            PGP+  P +G++   L  N    R I +  ++     +    L +T VI   +P +A+EI
Sbjct  94   PGPRGLPFVGSMS--LMSNALAHRCIAATAEKFGAKRLMAFSLGDTRVIVTCNPDVAKEI  151

Query  101  LKKQDSVFATRPLTMGTEYCSRGYLTVAVEPQGEQWKKMRRVVASHVTSKKSFQMMLQKR  160
            L     VFA RP+   + Y       +   P G  W+ +RR+ + H+ S K  +    +R
Sbjct  152  L--NSPVFADRPVK-ESAYSLMFNRAIGFAPYGVYWRTLRRIASYHLFSPKQIKRSETQR  208

Query  161  TEEADNLVRYINNRSVKNRGNAFVVIDLRLAVRQYSGNVARKMMFGIRHFGKGSEDGSGP  220
               A+ +V+ +  +S  N G  F     R  ++  S N     +FG  +  +   D
Sbjct  209  RVIANQIVKCLEKQSSGNEGLCFA----RDLIKTASLNSXMCSVFGKEYELEHEHDEVNE  264

Query  221  GLEEIEHVESLFTVLTHLYAFALSDYVPWLRFLDLEGHEKVVSNAMRNVSKYNDPFVDER  280
                +E    L   L        +D++PWL   D +G   + S     V K N  FV+
Sbjct  265  LRGLVEEGYDLLGTLN------WTDHLPWLSEFDPQG---IRSRCFSLVPKVNR-FVNRI  314

Query  281  LMQWRNGKMKEPQDFLDMFIIAKDTDGKPTLSDEEIKAQVTELMLATVDNPSNAAEWGMA  340
            +   R+     P DF+D+ +     DG   LSD ++ A + E++    D  +   EW +A
Sbjct  315  ISDHRDQTRDSPSDFVDVLL---SLDGPNKLSDPDMVAVLWEMIFRGTDTVAVLIEWILA  371

Query  341  EMINEPSIMQKAVEEIDRVVGKDRLVIESDLPNLNYVKACVKEAFRLHPVAP-FNLPHMS  399
             M+    I      E+D+VVG+ R V ESD+ +L Y+ A +KE  RLHP  P  +   ++
Sbjct  372  RMVFHQDIQTTVHNELDQVVGRSRAVEESDVASLTYLTAVIKEVLRLHPPGPLLSWARLA  431

Query  400  TTDTVVDGYFIPKGSHVLISRMGIGRNPSVWDKPHKFDPERHLSTNTCVDLN--ESDLNI  457
             TDT++DG  +P G+  +++   I  +P VW+ P +F+PER ++    VD +   SDL +
Sbjct  432  ITDTIIDGRRVPAGTTAMVNMWAIAHDPHVWENPLEFEPERFVAKEGDVDFSVLGSDLRL  491

Query  458  ISFSAGRRGCMGVDIGSAMTYMLLARLIQGFTWL-PVPGKNKIDISES-KNDLFMAKPL  514
              F +GRR C G ++G       +A L+  F WL P      +D+SE  +    MA PL
Sbjct  492  APFGSGRRVCPGKNLGLTTVTFWIATLLHEFEWLAPSSDDKTVDLSEKLRLSCEMANPL  550
```

>XP_009118796.1 PREDICTED: cytochrome P450 78A6-like [Brassica rapa]
Length=539

Score = 192 bits (487), Expect = 3e-54, Method: Compositional matrix adjust.
Identities = 148/492 (30%), Positives = 233/492 (47%), Gaps = 35/492 (7%)

```
Query  36   RNLSLPPGPKSWPLIGNLPEILGRNKPVFRWIHSLMKELNTD-IACIRLANTHVIPVTSP  94
            +N ++ PGP+ +PL+G++   L  +    + I  +    N   +    L +T VI    P
Sbjct  73   KNKNVIPGPRGFPLVGSMS--LRSSHVAHQRIADVAAMNNAKRLMAFSLGDTKVIVTCHP  130

Query  95   RIAREILKKQDSVFATRPLTMGTEYCSRGYLTVAVEPQGEQWKKMRRVVASHVTSKKSFQ  154
             +A+EIL    SVFA RP+   T Y       +   P G  W+ +RR+ ++H+ + K  +
Sbjct  131  EVAKEIL--NSSVFADRPVD-ETAYGLMFNRAMGFAPNGTYWRMLRRLGSNHLFNPKQIK  187

Query  155  MMLQKRTEEADNLVRYINNRSVKNRGNAFVVIDLRLAVRQYSGNVARKMMFGIRHFGKGS  214
               ++R   A  +V    N    N GN F V DL       +       M G+  FGK
Sbjct  188  RSEEQRRVIATRMV----NAFTSNAGNVFGVRDLLKTASLCN-------MMGLV-FGKEY  235

Query  215  EDGSGPGLEEIEHVESLFTVLTHLYA-FALSDYVPWLRFLDLEGHEKVVSNAMRNVSKYN  273
            E  +   +E  E+++SL       L      +D++PWL  LD +      S  +
Sbjct  236  ELETNNNVES-EYLKSLVEEGYDLLGTLNWTDHLPWLAGLDFQQIRFRCSQLV-------  287

Query  274  DPFVDERLMQWRNGKMKEPQDFLDMFIIAKDTDGKPTLSDEEIKAQVTELMLATVDNPSN  333
             P V++ L +  +       +FLD   +     G   LS+ ++ A + E++    D  +
Sbjct  288  -PKVNQLLSRIIHEHRTAACNFLD---VLHSLQGSEKLSESDMVAVLWEMIFRGTDTVAV  343

Query  334  AAEWGMAEMINEPSIMQKAVEEIDRVVGKDRLVIESDLPNLNYVKACVKEAFRLHPVAP-  392
              EW +A ++  P I     EE+DRVVG+ R V ESDLP+L Y+ A +KE  RLHP  P
```

```
Sbjct  344  LMEWVLARIVMHPKIQSTVHEELDRVVGRSRAVDESDLPSLTYLTAMIKEVLRLHPPGPL  403

Query  393  FNLPHMSTTDTVVDGYFIPKGSHVLISRMGIGRNPSVWDKPHKFDPERHLSTNTCVDLN-  451
              +   +S  DT VDGY +P G+  +++   I R+P VW+ P +F PER ++     + +
Sbjct  404  LSWARLSIADTTVDGYHVPAGTTAMVNMWAIARDPHVWENPLEFKPERFVAKEGEAEFSV  463

Query  452  -ESDLNIISFSAGRRGCMGVDIGSAMTYMLLARLIQGFTWLPVPGKNKIDISES-KNDLF  509
              SDL +  F +G+R C G ++G        +A+L+  F WLP    N  D+SE  +
Sbjct  464  FGSDLRLAPFGSGKRVCPGKNLGLTTVTFWVAKLLHEFEWLPSVNANPPDLSEVLRLSCE  523

Query  510  MAKPLYAVATPR  521
            MA PL     PR
Sbjct  524  MACPLVVDVRPR  535


>XP_009109368.1 PREDICTED: cytochrome P450 81F1-like [Brassica rapa]
Length=496

 Score = 191 bits (484),  Expect = 3e-54, Method: Compositional matrix adjust.
 Identities = 145/520 (28%), Positives = 244/520 (47%), Gaps = 37/520 (7%)

Query  7    MLAFIIGLLLLALTMKRKEKKKTMLISPTRNLSLPPGP-KSWPLIGNLPEILGRNKPVFR  65
            M  ++I +L LAL +      K +  S  +  +LPP P  + P++G+   I     PV R
Sbjct  1    MFYYVI-ILSLALFLV---AYKLIFSSKKQRFNLPPSPPYALPILGHHLLI---KPPVHR  53

Query  66   WIHSLMKELNTDIACIRLANTHVIPVTSPRIAREILKKQDSVF-ATRPLTMGTEYCSRGY  124
              H L K     I  +R+     + ++S  +A E    Q+ V  + RP  +  +Y +  Y
Sbjct  54   LFHRLSKTYGP-IFSLRVGYRRTVVISSSSLASECFTGQNDVLISNRPCFLTAKYVAYNY  112

Query  125  LTVAVEPQGEQWKKMRRVVASHVTSKKSFQMMLQKRTEEADNLVRYINNRSVKNRGNAFV  184
             TV   P G+ W+ +RRV +  + S        L  R +E   ++  ++    K
Sbjct  113  TTVGTAPYGDHWRHLRRVCSLEILSSNRLTNFLHIRKDEIRRMLTRLSREVDKE------  166

Query  185  VIDLRLAVRQYSGNVARKMMFGIRHFGKGSEDGSGPGLEEIEHVESLFTVLTHLY-AFAL  243
             I+L   +   + N   +M+ G R++G    +       EE    + L   +     A
Sbjct  167  -IELEPLLSDLTFNNIVRMVTGKRYYGDQVHNE-----EEANLFKKLVADVNDCSGARHP  220

Query  244  SDYVPWLRFLDLEGHEKVVSNAMRNVSKYNDPFVDERLMQWRNGKMKEPQDFLDMFIIAK  303
             DY+P+L+      +KV     ++V +  D  +   L + R  K     + +   +++
Sbjct  221  GDYLPFLKIFGGSFEKKV-----KSVGEAMDEILQRLLDECRRDK---GGNTMVNHLLSL  272

Query  304  DTDGKPTLSDEEIKAQVTELMLATVDNPSNAAEWGMAEMINEPSIMQKAVEEIDRVVGKD  363
                    +D  IK  +  +M+A  D  +   EW MA ++N P  ++KA +EID  +G++
Sbjct  273  QQQEPEYYTDVTIKGLMLGMMIAGTDTSAVTLEWAMACLLNHPESLEKAKQEIDEKIGQE  332

Query  364  RLVIESDLPNLNYVKACVKEAFRLHPVAPFNLPHMSTTDTVVDGYFIPKGSHVLISRMGI  423
            RL+ E DL  L Y++  V E FRL+P AP  +P  +T D  V GY +P+G+ V+++   I
Sbjct  333  RLIDEPDLEKLPYLQNIVSETFRLYPAAPLLVPRSTTDDIKVGGYDVPRGTMVMVNAWAI  392

Query  424  GRNPSVWDKPHKFDPERHLSTNTCVDLNESDLNIISFSAGRRGCMGVDIGSAMTYMLLAR  483
             R+PS+W++P KF PER         D+++    ++ F  GRR C G  +G  +  + L
Sbjct  393  HRDPSLWNEPEKFKPERFNIGEGGEDVHK----LMPFGNGRRACPGTGLGQRIVTLALGS  448

Query  484  LIQGFTWLPVPGKNKIDISESKNDLFMAK-PLYAVATPRL  522
            LIQ F W  V    KID++E+        K PL+A+ + RL
Sbjct  449  LIQCFDWEKV-NDEKIDMTETPGMAMRKKEPLWALCSSRL  487


>XP_009118025.1 PREDICTED: cytochrome P450 71B7 [Brassica rapa]
Length=507

 Score = 191 bits (484),  Expect = 4e-54, Method: Compositional matrix adjust.
 Identities = 132/471 (28%), Positives = 222/471 (47%), Gaps = 31/471 (7%)

Query  40   LPPGPKSWPLIGNLPEILGRNKPVFRWIHSLMKELNTDIACIRLANTHVIPVTSPRIARE  99
            LPPGPK+ P+IGNL  + G     FR +     +    +  +R     V+ ++S   A E
Sbjct  32   LPPGPKTLPIIGNLHNLNGLPHACFRNLSQTYGQ----VMLLRFGFVPVVVISSREGAEE  87

Query  100  ILKKQDSVFATRPLTMGTEYCSRGYLTVAVEPQGEQWKKMRRVVASHVTSKKSFQMMLQK  159
```

```
             LK QD    +RP T+ T   S  +  +   P GE+WK +R++V   + + K FQ
Sbjct  88   ALKTQDLECCSRPETVATRMISYNFKDIGFAPYGEEWKALRKLVVVELLNMKKFQSFSYI  147

Query  160  RTEEADNLVRYINNRSVKNRGNAFVVIDLRLAVRQYSGNVARKMMFGI--RHFGKGSEDG  217
            R EE + LV+ +   ++ +       ++L+  +     ++  ++ FGI         ED
Sbjct  148  REEENNLLVKKLMESALTHSP-----VNLKKTLFTLVASIVCRLAFGIDIHKCEFVDEDN  202

Query  218  SGPGLEEIEHVESLFTVLTHLYAFALSDYVPWLRFL--DLEGHEKVVSNAMRNVSKYNDP  275
                + + E V             A SD+ P + +L   + G  K ++N    +  +
Sbjct  203  VADLVHKFELVVD---------GIAFSDFFPGVGWLIDRVSGQNKTLNNVFSELDTFFQN  253

Query  276  FVDERLMQWRNGKMKEPQDFLDMFI---IAKDTDGKP-TLSDEEIKAQVTELMLATVDNP  331
             +D+ L   R   +    D +D+ +     ++ DG    L+ +  K  ++++ LA V+
Sbjct  254  ILDDHLKPGRT--VSGNPDVVDVMVDLMKKQEKDGDSFKLTTDHFKGIISDIFLAGVNTS  311

Query  332  SNAAEWGMAEMINEPSIMQKAVEEIDRVVG-KDRLVIESDLPNLNYVKACVKEAFRLHPV  390
                 W M E+I  P +M+K   EI   +G K + +   DL +L+Y K  VKE FRLHP
Sbjct  312  VITLIWAMTELIRNPRVMEKVQREIRTTLGDKKKSLTADDLNHLHYFKLVVKETFRLHPA  371

Query  391  APFNLPHMSTTDTVVDGYFIPKGSHVLISRMGIGRNPSVWDKPHKFDPERHLSTNTCVDL  450
            AP  LP  + +   + GY IP+ S ++I+   I R+P +W  P +F+P+R L  ++ VD
Sbjct  372  APLLLPRETMSQVKIQGYDIPEKSQMMINIYSIARDPKLWTNPDEFNPDRFL--DSSVDY  429

Query  451  NESDLNIISFSAGRRGCMGVDIGSAMTYMLLARLIQGFTWLPVPGKNKIDI  501
               +  ++ F +GRR C G+++G A   + L  L+  F W    GK   DI
Sbjct  430  RGLNFELLPFGSGRRICPGMNMGIATVELGLLNLLYFFNWGLPEGKTVKDI  480


>XP_009132738.1 PREDICTED: trans-cinnamate 4-monooxygenase-like [Brassica rapa]
Length=503

 Score = 190 bits (483),  Expect = 4e-54, Method: Compositional matrix adjust.
 Identities = 141/505 (28%), Positives = 236/505 (47%), Gaps = 29/505 (6%)

Query  1    MLDSTPMLAFIIGLLLLALTMKRKEKKKTMLISPTRNLSLPPGPKSWPLIGNLPEILGRN  60
            +L   P++A  + ++L  +  K   KK          L LPPGP   P+ GN  E+   N
Sbjct  4    LLLEKPLIAVFLAVVLAKMISKLCSKK----------LKLPPGPIPIPVFGNWLEV--GN  51

Query  61   KPVFRWIHSLMKELNTDIACIRLANTHVIPVTSPRIAREILKKQDSVFATRPLTMGTEYC  120
                R +    K+   D+  +R+    ++ ++SP +A+E+L+ Q   F +R   +  +
Sbjct  52   DLNHRNLVDYAKKFG-DLFHLRMGQRDIVVISSPDLAKEVLQTQGVEFGSRYRNIVYDIF  110

Query  121  SRGYLTVAVEPQGEQWKKMRRVVASHVTSKKSFQMMLQKRTEEADNLVRYIN-NRSVKNR  179
            +     +     GE W+KMRR++     + K  Q   +    EA ++V  +  N     +
Sbjct  111  TGKGQDMVFTVYGEHWRKMRRIMTVPFFTNKVVQQNREGWEFEAASVVEEVKKNPDAATK  170

Query  180  GNAFVVIDLRLAVRQYSGNVARKMMFGIRHFGKGSEDGSGPGLEEIEHVESLFTVLTHLY  239
            G   +V   RL +  Y+ N+ R M      FGK  E    P L  ++ +    + LT  +
Sbjct  171  G---IVARKRLQLMMYN-NMFRVM------FGKRFESEDDPLLLRLKFLNGERSRLTQSF  220

Query  240  AFALSDYVPWLRFLDLEGHEKVVSNAM-RNVSKYNDPFVDERLMQWRNGKMKEPQDFLDM  298
             +   D++P LR   L G+ K   +   R ++ +   FVDER             +  +D
Sbjct  221  EYNYGDFIPILRPF-LRGYLKSCQDVKERRLALFKKYFVDERKEIASAKPTGSVKYAIDH  279

Query  299  FIIAKDTDGKPTLSDEEIKAQVTELMLATVDNPSNAAEWGMAEMINEPSIMQKAVEEIDR  358
             + A++   K  ++ + +   V  + +A ++    + EWG+AE++N P I  K   EID
Sbjct  280  ILEAEE---KGEINADNVLYIVENINVAAIETTLWSIEWGIAELVNHPEIQSKLRNEIDT  336

Query  359  VVGKDRLVIESDLPNLNYVKACVKEAFRLHPVAPFNLPHMSTTDTVVDGYFIPKGSHVLI  418
            V+G    V E DL  L Y++  +KE  RL    P  +PHM+  D  + GY IP  S +L+
Sbjct  337  VLGPGVQVTEPDLHKLPYLQVVLKETLRLRMGVPLLVPHMNLKDAKLAGYDIPAESKILV  396

Query  419  SRMGIGRNPSVWDKPHKFDPERHLSTNTCVDLNESDLNIISFSAGRRGCMGVDIGSAMTY  478
            +   +G N   W KP +F PER L     V+ N +D   + F  GRR C G+ +   +
Sbjct  397  NAWWLGNNSESWKKPEEFRPERFLEEEAHVEANGNDFRYLPFGLGRRSCPGIVLALPILG  456

Query  479  MLLARLIQGFTWLPVPGKNKIDISE  503
            + + RL+Q F   P PG++K+D +E
Sbjct  457  ITIGRLVQNFELFPPPGQSKVDTTE  481
```

```
>XP_009107632.1 PREDICTED: cytochrome P450 83B1 [Brassica rapa]
Length=511

 Score = 189 bits (481),  Expect = 1e-53, Method: Compositional matrix adjust.
 Identities = 137/493 (28%), Positives = 223/493 (45%), Gaps = 22/493 (4%)

Query  33   SPTRNLSLPPGPKSWPLIGNLPEILGRNKPVFRWIHSLMKELNTDIACIRLANTHVIPVT  92
            S  ++L LPPGPK  P+IGNL ++   N   F +    + +L   I  +++    +  ++
Sbjct  34   STKKSLRLPPGPKGLPIIGNLHQMEKFNPQHFLF---RLSKLYGPIFTMKIGGRRLAVIS  90

Query  93   SPRIAREILKKQDSVFATRPLTMGTEYCSRGYLTVAVEPQGEQWKKMRRVVASHVTSKKS  152
            S  +A+E+LK QD  F  RPL  G +  S     +        +++MR++   ++ S
Sbjct  91   SAELAKELLKTQDLNFTARPLLKGQQTMSYQGRELGFGQYTAYYREMRKMCMVNLFSPNR  150

Query  153  FQMMLQKRTEEADNLVRYINNRSVKNRGNAFVVIDLRLAVRQYSGNVARKMMFGIRHFGK  212
                   R EE   ++  I  ++    G     +DL   +  ++  V  +  FG R+
Sbjct  151  VASFRPVREEECQRMMDKIY-KAADQSGT----VDLSELLLSFTNCVVCRQAFGKRYNEY  205

Query  213  GSEDGSGPGLEEIEHVESLFTVLTHLYAFALSDYVPWLRFLD-LEGHEKVVSNAMRNVSK  271
            G+E            +  L+     L     SD  P+  FLD L G    +  A + +
Sbjct  206  GTEMK--------RFINILYETQALLGTLFFSDLFPYFGFLDNLTGLNARLKRAFKELDT  257

Query  272  YNDPFVDERLMQWRNGKMKEPQDFLDMFI-IAKDTDGKPTLSDEEIKAQVTELMLATVDN  330
            Y    +DE L   R     E + F+D+ + I KD       + E +KA + ++++   D
Sbjct  258  YLQELLDETLDPSR--PKPETESFIDLLMQIYKDQPFSIKFTHENVKAMILDIVVPGTDT  315

Query  331  PSNAAEWGMAEMINEPSIMQKAVEEIDRVVGKDRLVIESDLPNLNYVKACVKEAFRLHPV  390
             +    W M  +I  P  M+KA +E+  VVG    V E D+PNL Y+KA +KE+ RL PV
Sbjct  316  AAAVVVWAMTYLIKYPEAMKKAQDEVRNVVGDKGYVSEEDIPNLPYLKAVIKESLRLEPV  375

Query  391  APFNLPHMSTTDTVVDGYFIPKGSHVLISRMGIGRNPSVW-DKPHKFDPERHLSTNTCVD  449
             P  L    +  D  + GY IP  + + ++    + R+ + W D P++F PER ++    VD
Sbjct  376  IPILLHRETIADAKIGGYDIPAKTIIQVNAWAVSRDTAAWGDNPNEFIPERFMNEQKGVD  435

Query  450  LNESDLNIISFSAGRRGCMGVDIGSAMTYMLLARLIQGFTWLPVPGKNKIDIS-ESKNDL  508
                D  ++ F +GRR C  + +G AM  +  A L+  F W    G    DI  +    L
Sbjct  436  FKGQDFELLPFGSGRRMCPAMHLGVAMVEIPFANLLYRFDWSLPKGIKPEDIKMDVMTGL  495

Query  509  FMAKPLYAVATPR  521
             M K  + V  PR
Sbjct  496  AMHKKDHLVLAPR  508


>XP_009138804.1 PREDICTED: cytochrome P450 76C4-like [Brassica rapa]
Length=500

 Score = 189 bits (480),  Expect = 1e-53, Method: Compositional matrix adjust.
 Identities = 137/454 (30%), Positives = 219/454 (48%), Gaps = 30/454 (7%)

Query  78   IACIRLANTHVIPVTSPRIAREILKKQDSVFATRPLTMGTEYCSRGYLTVAVEPQGEQWK  137
            +  +RL +   + ++SP  ARE+LK  D V + R +    +   +      +     +W+
Sbjct  64   VMSLRLGSLTTVIISSPEAAREVLKTHDQVLSGRIILDPIQSIHQDVSMAWLPSTSPRWR  123

Query  138  KMRRVVASHVTSKKSFQMMLQKRTEEADNLVRYINNRSVKNRGNAFVVIDLRLAVRQYSG  197
              R++ A+ + S +        R ++ + LV +I+   +  RG +   I++  A    S
Sbjct  124  LWRKISATQMFSPQCLDATKTVRMKKVNELVTFISE--ICERGES---INIARASFVTSL  178

Query  198  NVARKMMFGIRHFGKGSEDGSGPGLEEIEHVESLFTVLTHLYAFALSDYVPWLRFLDLEG  257
            N+    +F        ++ GS      +E  ES+  ++  +    L++Y P + FLDL+G
Sbjct  179  NLISNTLFS-------TDLGSYDSKISMELQESVVRIMETIGKPNLANYFPLIGFLDLQG  231

Query  258  ---HEKVVSNAMRNVSKYNDPFVDERLMQWRNGKM-KEPQDFLDMFIIAKDTDGKPTLSD  313
                 KV S+ +  V +    F+D R     N K  +   D LD  +     +G P L
Sbjct  232  IRKEMKVCSDVLFQVFQ---GFIDAR----NNEKTTRNESDLLDSLMDLVKENG-PELKV  283

Query  314  EEIKAQVTELMLATVDNPSNAAEWGMAEMINEPSIMQKAVEEIDRVVGKDRLVIESDLPN  373
             EIK  + +L LA  D  S   EW MAE++  P  M KA  E+D VVG + +V ES + +
```

```
Sbjct  284  NEIKHFIFDLFLAGTDTNSTTVEWAMAELLRNPKTMAKAQAEMDDVVGLNGVVQESHISD  343

Query  374  LNYVKACVKEAFRLHPVAPFNLPHMSTTDTVVDGYFIPKGSHVLISRMGIGRNPSVWDKP  433
            L Y++A VKE  RLHP  P   PH + ++  + G+ +PK + VL++   IGR+  +W+
Sbjct  344  LPYLQALVKETLRLHPPGPLLGPHKAESNAEILGFLVPKNAQVLVNAWAIGRDSGIWENA  403

Query  434  HKFDPERHLSTNTCVDLNESDLNIISFSAGRRGCMGVDIGSAMTYMLLARLIQGFTWLPV  493
             +F+PER L     +DL   D  +I F AGRR C G+ +      ++LA L+  F W
Sbjct  404  EQFEPERFL-VGREIDLKGRDFELIPFGAGRRICPGMSLAMKTVSLILASLLHSFQWKLQ  462

Query  494  PG--KNKIDISESKN-DLFMAKPLYAVATPRLAP  524
             G     +D+ ES    L  A PLYAV  P L P
Sbjct  463  NGVLPEDLDMDESFGLSLHKANPLYAV--PVLKP  494


>XP_009138803.1 PREDICTED: cytochrome P450 76C4-like [Brassica rapa]
Length=499

 Score = 188 bits (478),  Expect = 3e-53, Method: Compositional matrix adjust.
 Identities = 135/454 (30%), Positives = 222/454 (49%), Gaps = 29/454 (6%)

Query  78   IACIRLANTHVIPVTSPRIAREILKKQDSVFATRPLTMGTEYCSRGYLTVAVEPQGE-QW  136
            +  +R  +   + ++SP  ARE+LK  D V + R +            +++A  P    +W
Sbjct  65   VMSLRFGSLTTVIISSPEAAREVLKTHDQVLSGRIILDPIRSIDHQDVSMAWLPSTSPRW  124

Query  137  KKMRRVVASHVTSKKSFQMMLQKRTEEADNLVRYINNRSVKNRGNAFVVIDLRLAVRQYS  196
            +  R++ A+H+ S +        R ++ + LV ++  R +  RG +   +D+  A    S
Sbjct  125  RLWRKISATHMFSLQCLDATKSVRMKKVNELVTFM--REICERGES---VDIARASFVTS  179

Query  197  GNVARKMMFGIRHFGKGSEDGSGPGLEEIEHVESLFTVLTHLYAFALSDYVPWLRFLDLE  256
             N+     F        ++ GS      +E  ES+  ++  +    L++Y P + FLD++
Sbjct  180  LNIISNTFFS-------TDLGSYDPRTSMELQESVVRIMETIGKPNLANYFPLIGFLDMQ  232

Query  257  G---HEKVVSNAMRNVSKYNDPFVDERLMQWRNGKMKEPQ-DFLDMFIIAKDTDGKPTLS  312
            G     KV S+ +  V +    F+D R    +N K  + + D LD  +     +G   L+
Sbjct  233  GIRKEMKVCSDILFTVFQ---GFIDAR----KNEKSSQNEIDLLDSLMNLVKENGS-ELN  284

Query  313  DEEIKAQVTELMLATVDNPSNAAEWGMAEMINEPSIMQKAVEEIDRVVGKDRLVIESDLP  372
              +IK  + ++ L   D  S   EW MAE++  P  M KA  E+D VVG + +V ESD+
Sbjct  285  VNDIKHFLYDMFLGGTDTNSTVVEWAMAELLRNPKTMAKAQAEMDDVVGPNGVVQESDIS  344

Query  373  NLNYVKACVKEAFRLHPVAPFNLPHMSTTDTVVDGYFIPKGSHVLISRMGIGRNPSVWDK  432
            +L Y++A VKE  RLHP  P   PH + T+  V G+ +PK + VL++   IGR+ S+W+
Sbjct  345  DLPYLQAVVKETLRLHPPGPLLAPHKAETNVEVLGFLVPKNAQVLVNVWYIGRDSSIWEN  404

Query  433  PHKFDPERHLSTNTCVDLNESDLNIISFSAGRRGCMGVDIGSAMTYMLLARLIQGFTWLP  492
              +F+PER LS    +D+   D  +I F AGRR C G+ +      ++LA L+  F W
Sbjct  405  AERFEPERFLSGRE-IDVKGRDFELIPFGAGRRICPGMSVAMKTVPLILASLLHSFHWKL  463

Query  493  VPG--KNKIDISESKN-DLFMAKPLYAVATPRLA  523
              G     +D+ ES    L    PLYAV   + A
Sbjct  464  QNGVLPEGLDMDESFGLTLHKTNPLYAVPVKKRA  497


>XP_009148546.1 PREDICTED: cytochrome P450 71B7-like [Brassica rapa]
Length=502

 Score = 187 bits (476),  Expect = 5e-53, Method: Compositional matrix adjust.
 Identities = 132/485 (27%), Positives = 232/485 (48%), Gaps = 42/485 (9%)

Query  32   ISPTRNLSLPPGPKSWPLIGNLPEILGRNKPVFRWIHSLMKELNTD---IACIRLANTHV  88
            + P+   +LPPGPK  P+IGNL  + G         +H  +++L+     +  ++     +
Sbjct  21   LKPSSRWNLPPGPKKLPIIGNLHNLQG-------MLHLCLRDLSQTYGPVMLLKFGFVRM  73

Query  89   IPVTSPRIAREILKKQDSVFATRPLTMGTEYCSRGYLTVAVEPQGEQWKKMRRVVASHVT  148
            + +TS   A E+LK  D    +RP T+ +   S  +  +   P GE+WK +R++    +
Sbjct  74   VVITSKEAAEEVLKTLDLECCSRPETVSSRTVSYNFKDIGFAPYGEEWKALRKLSVVEIF  133

Query  149  SKKSFQMMLQKRTEEADNLVRYINNRSVKNRGNAFVVIDLRLAVRQYSGNVARKMMFG--  206
```

```
            S K  Q     R EE D LV+ ++  +     +    ++L+  +     ++  ++ FG
Sbjct  134  STKKIQSFRYIREEENDLLVKKLSECA-----STRFPVNLKKTLFTLVASIVCRLAFGQD  188

Query  207  IRHFGKGSEDGSGPGLEEIEHVESLFTVLTHLYAFALSDYVP----WLRFLD-LEGHEKV  261
            +       ED     +++ E V         L + A SD+ P    W+  LD + G  K
Sbjct  189  LHKCEFIDEDSIAELVQKSEMV---------LASSAFSDFFPGGTGWV--LDKITGQNKK  237

Query  262  VSNAMRNVSKYNDPFVDERLMQWRNGKMKEPQDFLDMFI---IAKDTDGKP-TLSDEEIK  317
            +++    +  +    +D+ L   R   + +  D +D+ I     ++ DG    L+ + +K
Sbjct  238  LNSVFSELDAFFQNILDDHLRPGRT--VLDSPDVVDVMIDMMKKQERDGDSFKLTTDHLK  295

Query  318  AQVTELMLATVDNPSNAAEWGMAEMINEPSIMQKAVEEIDRVVG-KDRLVIESDLPNLNY  376
              ++++ LA V+  +    WGM E+I  P +M+K  +EI   +G K   V E D+  L+Y
Sbjct  296  GIISDIFLAGVNTSAMTLIWGMTELIRNPRVMKKVQKEIRTTLGDKKEKVTEEDVNKLHY  355

Query  377  VKACVKEAFRLHPVAPFNLPHMSTTDTVVDGYFIPKGSHVLISRMGIGRNPSVWDKPHKF  436
             K  VKE FRLHP AP  LP  + ++  + GY IP  + ++++   I R+P +W  P +F
Sbjct  356  FKLMVKELFRLHPAAPLLLPRETLSNIKIQGYDIPAKTQIMVNVYSIARDPKLWTNPDEF  415

Query  437  DPERHLSTNTCVDLNESDLNIISFSAGRRGCMGVDIGSAMTYMLLARLIQGFTWLPVPGK  496
            +P+R L  +  VD    +  ++ F +GRR C G+ +G A   + L  L+  F W    GK
Sbjct  416  NPDRFL--DMSVDYRGLNFELLPFGSGRRICPGMTMGVATVELGLLNLLYFFDWALPEGK  473

Query  497  NKIDI  501
               DI
Sbjct  474  TVKDI  478


>XP_009143151.1 PREDICTED: cytochrome P450 76C3-like [Brassica rapa]
Length=511

 Score = 187 bits (476),  Expect = 5e-53, Method: Compositional matrix adjust.
 Identities = 128/445 (29%), Positives = 218/445 (49%), Gaps = 22/445 (5%)

Query  78   IACIRLANTHVIPVTSPRIAREILKKQDSVFATRPLTMGTEYCSRGYLTVAVEPQGEQWK  137
            I  ++L  +  + ++SP  A+E LK  D V + R              +V   P   +W+
Sbjct  75   IMSLKLGRSTAVVISSPEAAKEALKTHDHVMSARTFNDPIRAFDHHKHSVVWIPASARWR  134

Query  138  KMRRVVASHVTSKKSFQMMLQKRTEEADNLVRYINNRSVKNRGNAFVVIDLRLAVRQYSG  197
             +++++  ++ S ++   +   R  + + L+  +N  +   RG A   ID+  A    S
Sbjct  135  FLKKIIVQNLLSPQNLDGIQSIRIRKVEELLSLVN--TFCERGEA---IDMARASFITSF  189

Query  198  NVARKMMFGIRHFGKGSEDGSGPGLEEIEHVESLFTVLTHLYAFALSDYVPWLRFLDLEG  257
            N+    +F +      + + +    E  E V  L  +     A    D+  +LRFLDL+G
Sbjct  190  NIISNALFSV---DLATYNSNSSSFEFHETVVHLMEICGKPNA---GDFFRFLRFLDLQG  243

Query  258  HEKVVSNAMRNVSKYNDPFVDERLMQWRNGKMKEPQDFLDMFIIAKDTDGKPTLSDEEIK  317
              K  +  +  + +    F+D+R+ +  +       D LD  +  K  + +  ++  ++K
Sbjct  244  SRKESTLCIEKLFRVFQEFIDDRVAKRLSQTGASSNDMLDALLDIKQQN-QEEITINDMK  302

Query  318  AQVTELMLATVDNPSNAAEWGMAEMINEPSIMQKAVEEIDRVVGKDRLVIESDLPNLNYV  377
                +L +A  D  S+  EW M E++  P  M +A  EI +V+G++ +V ESD+  L+Y+
Sbjct  303  HLFLDLFVAGTDTNSSTMEWAMTELLRNPEKMVRAQSEIRQVIGENGVVQESDISKLSYL  362

Query  378  KACVKEAFRLHPVAPFNLPHMSTTDTVVDGYFIPKGSHVLISRMGIGRNPSVWDKPHKFD  437
             A VKE  RLHP AP  +P  S +D  + G+F+PK S VL++   +GR+ +VW+ P KF+
Sbjct  363  LAIVKETLRLHPPAPL-IPRKSESDVQIFGFFVPKNSQVLVNVWAMGRDSNVWENPMKFE  421

Query  438  PERHLSTNTCVDLNESDLNIISFSAGRRGCMGVDIGSAMTYMLLARLIQGFTWL----PV  493
            PER L     +D+   D  ++ F +GRR C G+ +    T M+LA L+  F W      V
Sbjct  422  PERFLLRE--IDVRGKDFELLPFGSGRRMCPGISMSLKTTPMVLASLLYSFDWKLQDGIV  479

Query  494  PGKNKIDISESKN-DLFMAKPLYAV  517
            PG   +D+SE     L  AKPL  V
Sbjct  480  PG--NMDMSEVFGLTLHKAKPLCIV  502


>XP_009150199.1 PREDICTED: cytochrome P450 71A26 [Brassica rapa]
Length=511
```

```
 Score = 186 bits (472),  Expect = 2e-52, Method: Compositional matrix adjust.
 Identities = 135/484 (28%), Positives = 230/484 (48%), Gaps = 36/484 (7%)

Query  41   PPGPKSWPLIGNLPEILGRNKPVFRWIHSLMKELNTDIACIRLANTHVIPVTSPRIAREI  100
            P  P S PLIGNL + LG +    R +   +      +  +   +  V+ V+S   A+E+
Sbjct  56   PSSPPSLPLIGNLHQ-LGHHP--HRSL-CFLSHRYGPLMLLHFGSVPVLVVSSMEAAKEV  111

Query  101  LKKQDSVFATRPLTMGTEYCSRGYLTVAVEPQGEQWKKMRRVVASHVTSKKSFQMMLQKR  160
            LK  D VFA+RP +   +         VA  P GE W++M+ V   H+ S K  +     R
Sbjct  112  LKTHDRVFASRPRSKIFQKLLYDGQDVAAAPYGEYWRQMKSVCVLHLLSNKMVRSFRNVR  171

Query  161  TEEADNLVRYINNRSVKNRGNAFVVIDLRLAVRQYSGNVARKMMFGIRHFGKGSEDGSGP  220
             EE   ++  I       R  + + ++L   +   + +V  ++  G ++   G E
Sbjct  172  EEEMSLMMEKI-------RKASSLPVNLSELLANLTNDVICRVALGRKY---GCETDFKE  221

Query  221  GLEEIEHVESLFTVLTHLYAFALSDYVPWLRFLD-LEGHEKVVSNAMRNVSKYNDPFVDE  279
             +E +  +  +F+V T         YVPWL ++D + G +  +     +V    D F++
Sbjct  222  LMERLTRLLGVFSVGT---------YVPWLAWIDWIRGLDSQLEKLRNDV----DEFLER  268

Query  280  RLMQWRNGKMKEPQDFLDMFI-IAKDTDGKPTLSDEEIKAQVTELMLATVDNPSNAAEWG  338
             L    +G   +  DF+D+ + I ++      +    IKA + ++ +   D      EW
Sbjct  269  VLQDHEDGDGGDRTDFVDVLLKIQREKSVGFDIDRVSIKAIILDVFVGGTDTSYTLMEWV  328

Query  339  MAEMINEPSIMQKAVEEIDRVV-GKDRLVIESDLPNLNYVKACVKEAFRLHPVAPFNLPH  397
            M E++  P  +++  EE+  +  GK  ++ E D+ ++NY+KA +KE  RLHP  P  +PH
Sbjct  329  MTELLRHPECLRRLQEEVRTICKGKSSVLEEEDIQDMNYLKAVIKETLRLHPPLPLMVPH  388

Query  398  MSTTDTVVDGYFIPKGSHVLISRMGIGRNPSVWD-KPHKFDPERHLSTNTCVDLNESDLN  456
             ST D  +  Y IP G+ V+I+   IGR    W     +F PERHL  ++ VD    D
Sbjct  389  ESTHDVRLRNYRIPAGTQVMINAWAIGREVETWGPDAEEFRPERHL--HSSVDFRGQDFE  446

Query  457  IISFSAGRRGCMGVDIGSAMTYMLLARLIQGFTWLPVPGKNKIDISESKN-DLFMAKPLY  515
            +I F AGRR C  +     +  ++LA L+  F W  +  +++ +++ES    +    PLY
Sbjct  447  LIPFGAGRRICPAISFAVVLNEVVLANLVHQFGW--ISTEDQAEVAESTGIAIHRMFPLY  504

Query  516  AVAT  519
            A+A+
Sbjct  505  AIAS  508


>XP_009118033.1 PREDICTED: cytochrome P450 71B2-like [Brassica rapa]
Length=508

 Score = 186 bits (471),  Expect = 3e-52, Method: Compositional matrix adjust.
 Identities = 134/461 (29%), Positives = 217/461 (47%), Gaps = 25/461 (5%)

Query  35   TRNLSLPPGPKSWPLIGNLPEILGRNKPVFRWIHSLMKELNTDIACIRLANTHVIPVTSP  94
            T   +LPP P S P+IGNL  + G      R  H+L  +    +  +RL    V+ ++S
Sbjct  26   TSKFNLPPSPSSLPIIGNLHHLSGLPH---RCFHNLSLKYGP-VMLLRLGFVPVVVISSS  81

Query  95   RIAREILKKQDSVFATRPLTMGTEYCSRGYLTVAVEPQGEQWKKMRRVVASHVTSKKSFQ  154
              A  +LK  D    +RP T+GT   S G+  ++  P G  W++MR++    + S K  Q
Sbjct  82   EAAEAVLKTHDLECCSRPKTVGTGKLSYGFKDISFSPYGAYWREMRKIAVIELLSLKKVQ  141

Query  155  MMLQKRTEEADNLVRYINNRSVKNRGNAFVVIDLRLAVRQYSGNVARKMMFGIRHFGKGS  214
                 R EE D +V+ ++  ++          +DL       + ++  ++  G ++F
Sbjct  142  SFRYIREEEVDYVVKKVSESALTQSP-----VDLSKTFFSLTASIICRVALG-QNF---H  192

Query  215  EDGSGPGLEEIEHVESLFTVLTHLYAFALSDYVPWL--RFLD-LEGHEKVVSNAMRNVSK  271
             DG      E IE + +   +   L  F  SD+ P    RFLD L    K ++ A + +
Sbjct  193  VDGFVIDQERIEELVTDGAI--ALGTFTFSDFFPGGAGRFLDWLFRRNKKINRAFKELDA  250

Query  272  YNDPFVDERLMQWRNGKMKEPQDFLDMFIIAKDTDG-KPTLSDEEIKAQVTELMLATVDN  330
            +    +D+ L +    K K+    L   I  +D D  KP++  + +KA V ++ LA +D
Sbjct  251  FYQHVIDDHL-KPEGRKNKDIVSLLLDMIDKEDADSFKPSM--DNLKAIVMDVFLAGIDT  307

Query  331  PSNAAEWGMAEMINEPSIMQKAVEEIDRVVGKDR-LVIESDLPNLNYVKACVKEAFRLHP  389
             S    W M E++  P +M+KA E I   +G  R  + E DL  + Y+   +KE FRLHP
```

```
Sbjct  308  SSITMIWAMTELVRNPRVMKKAQENIRITLGAKREKITEDDLGKVEYLSLIIKETFRLHP  367

Query  390  VAPFNLPHMSTTDTVVDGYFIPKGSHVLISRMGIGRNPSVWDKPHKFDPERHLSTNTCVD  449
              PF +P  + +   + GY IP  + + ++   IGR+P  W  P  F PER    N+ VD
Sbjct  368  PLPFIIPRETMSHIKIQGYDIPPKTQIQVNVWAIGRDPKRWTDPEDFIPERF--ANSSVD  425

Query  450  LNESDLNIISFSAGRRGCMGVDIGSAMTYMLLARLIQGFTW  490
                   ++ F +GRR C  + +G+A   + L  L+  F W
Sbjct  426  FRGQHFELLPFGSGRRMCPAMPMGAATVELGLMNLLYFFDW  466
```

>XP_009129563.1 PREDICTED: LOW QUALITY PROTEIN: cytochrome P450 71B19-like [Brassica
rapa]
Length=502

```
 Score = 186 bits (471),  Expect = 3e-52, Method: Compositional matrix adjust.
 Identities = 138/504 (27%), Positives = 232/504 (46%), Gaps = 43/504 (9%)

Query  8    LAFIIGLLLLALTMKRKEKKKTMLISPTRNLSLPPGPKSWPLIGNLPEILGRNKPVFRWI  67
              F+  L L+ L  K K+ K           +LPP P ++P+IGNL +I    +   R +
Sbjct  8    FCFVTFLTLIFLVKKIKQSK----------WNLPPTPPTFPVIGNLHQI---GELPHRSL  54

Query  68   HSLMKELNTDIACIRLANTHVIPVTSPRIAREILKKQDSVFATRPLTMGTEYCSRGYLTV  127
             SL +     +  I      ++ +TS   A E+L+  D    +RP  +GT   SR +  V
Sbjct  55   QSLAQRFGP-VMLIHFGFVPLVVITSKEAAEEVLRTHDLDCCSRPKLVGTRLLSRDFKDV  113

Query  128  AVEPQGEQWKKMRRVVASHVTSKKSFQMMLQKRTEEADNLVRYINNRSVKNRGNAFVVID  187
               P GE+WK+ R+     +   ++ Q     R EE + LVR ++  +V          +D
Sbjct  114  GFTPYGEEWKERRKFAVRELFXSENVQSFRHIREEECNLLVRKLSESAVDR-----TPVD  168

Query  188  LRLAVRQYSGNVARKMMFGIRHFGKGSEDGSGPGLEEIEHVESLFTVLTHLYAFALSDYV  247
            L   +   + ++  ++  G     +   +      EEIE  E +F   T L +F  SD+
Sbjct  169  LSKTLFWLTASILFRVALG-----QDFHESKFIDKEEIE--ELVFEAETALASFTCSDFF  221

Query  248  P-----WLRFLD-LEGHEKVVSNAMRNVSKYNDPFVDERLMQWRNGKMKEPQDFLDMFII  301
            P     WL  +D   G  K +++    +       +D+ +     G+ K+ +D +D  +
Sbjct  222  PVAGLGWL--VDWFSGQHKRLNDVYLKLDTLFQLVIDDHM---NPGRTKDHEDIIDAMLD  276

Query  302  AKDTDGKP---TLSDEEIKAQVTELMLATVDNPSNAAEWGMAEMINEPSIMQKAVEEIDR  358
                 GK     L+ + IK  +  + LA +D  +    W M E+  +P +M+K  +EI
Sbjct  277  VIHKQGKNDSLKLTVDHIKGFLANIFLAGIDTGAITMIWAMTELAKKPKLMKKVQDEIRD  336

Query  359  VVGKDRLVI-ESDLPNLNYVKACVKEAFRLHPVAPFNLPHMSTTDTVVDGYFIPKGSHVL  417
             +G ++  I E D+  + Y+K  +KE FRLHP AP  LP  + +   V GY I   + +L
Sbjct  337  CLGNNKETITEEDVDKVPYLKLVIKETFRLHPAAPLILPRETMSHMKVQGYDILPKTRIL  396

Query  418  ISRMGIGRNPSVWDKPHKFDPERHLSTNTCVDLNESDLNIISFSAGRRGCMGVDIGSAMT  477
            ++   IGR+P +W +P +F+PER + ++  VD       ++ F +GRR C G+ +G A
Sbjct  397  VNTWAIGRDPKLWTEPEEFNPERFIDSH--VDYRGQHYELLPFGSGRRMCPGMPMGIATV  454

Query  478  YMLLARLIQGFTWLPVPGKNKIDI  501
             + L  L+  F W    G    DI
Sbjct  455  ELGLLNLLYFFDWRVPEGMTHKDI  478
```

>NP_001288861.1 trans-cinnamate 4-monooxygenase [Brassica rapa]
Length=505

```
 Score = 185 bits (469),  Expect = 5e-52, Method: Compositional matrix adjust.
 Identities = 130/471 (28%), Positives = 226/471 (48%), Gaps = 19/471 (4%)

Query  36   RNLSLPPGPKSWPLIGNLPEILGRNKPVFRWIHSLMKELNTDIACIRLANTHVIPVTSPR  95
            + L LPPGP   P+ GN  ++   +    R +    K+   D+  +R+   +++ V+SP
Sbjct  29   KKLKLPPGPMPIPIFGNWLQV--GDDLNHRNLVDYAKKFG-DLFLLRMGQRNLVVVSSPN  85

Query  96   IAREILKKQDSVFATRPLTMGTEYCSRGYLTVAVEPQGEQWKKMRRVVASHVTSKKSFQM  155
            + +E+L  Q   F +R   +  +  +     +     GE W+KMRR++     + K  Q
Sbjct  86   LTKEVLHTQGVEFGSRTRNVVFDIFTGKGQDMVFTVYGEHWRKMRRIMTVPFFTNKVVQQ  145
```

```
Query  156  MLQKRTEEADNLVRYINNRSVKNRGNAFVVIDLRLAVRQYSGNVARKMMFGIRHFGKGSE  215
              +    EA ++V  +     KN  +A   I LR  ++    N   ++MF  R     SE
Sbjct  146  NREGWEFEAASVVEDVK----KNPDSATKGIVLRKRLQLMMYNNMFRIMFDRRF---DSE  198

Query  216  DGSGPGLEEIEHVESLFTVLTHLYAFALSDYVPWLRFLDLEGHEKVVSNAM-RNVSKYND  274
            D   P    ++ +    + L   + +   D++P LR   L G+ K+  +   R ++ +
Sbjct  199  DD--PLFIRLKALNGERSRLAQSFEYNYGDFIPILRPF-LRGYLKICQDVKDRRLALFKK  255

Query  275  PFVDER--LMQWRNGKMKEPQDFLDMFIIAKDTDGKPTLSDEEIKAQVTELMLATVDNPS  332
             FVDER  +  W+    +  +  +D  + A+    K  ++++ +   V  + +A ++
Sbjct  256  YFVDERKQIASWKPTGSEGLKCAIDHILEAQQ---KGEINEDNVLYIVENINVAAIETTL  312

Query  333  NAAEWGMAEMINEPSIMQKAVEEIDRVVGKDRLVIESDLPNLNYVKACVKEAFRLHPVAP  392
             + EWG+AE++N P I  K   EID V+G    V E +L  L Y++A ++E  RL    P
Sbjct  313  WSIEWGIAELVNHPEIQTKLRNEIDTVLGPGVQVTEPELHKLPYLQAVIEETLRLRMAIP  372

Query  393  FNLPHMSTTDTVVDGYFIPKGSHVLISRMGIGRNPSVWDKPHKFDPERHLSTNTCVDLNE  452
              +PHM+  D  + GY IP  S +L++   +  NP  W KP +F PER       V+ N
Sbjct  373  LLVPHMNLNDAKLAGYDIPAESKILVNAWWLANNPESWKKPEEFRPERFFEEEAHVEANG  432

Query  453  SDLNIISFSAGRRGCMGVDIGSAMTYMLLARLIQGFTWLPVPGKNKIDISE  503
            +D   + F  GRR C G+ +   +  + + R++Q F  LP PG++K+D SE
Sbjct  433  NDFRYVPFGVGRRSCPGIILALPILGITIGRMVQNFELLPPPGQSKLDTSE  483


>XP_009142434.1 PREDICTED: cytochrome P450 78A6 [Brassica rapa]
Length=527

 Score = 185 bits (470),  Expect = 5e-52, Method: Compositional matrix adjust.
 Identities = 147/481 (31%), Positives = 226/481 (47%), Gaps = 35/481 (7%)

Query  42   PGPKSWPLIGNLPEILGRNKPVFRWIHSLMKELNTD-IACIRLANTHVIPVTSPRIAREI  100
            PGP+ +P +G++   L  +    R I  + +      +    L  T VI   +P +A+EI
Sbjct  69   PGPRGFPFVGSMS--LMSSTLAHRRIADVAERFGAKRLLAFSLGETRVIVTCNPDVAKEI  126

Query  101  LKKQDSVFATRPLTMGTEYCSRGYLTVAVEPQGEQWKKMRRVVASHVTSKKSFQMMLQKR  160
            L     VFA RP+   + Y       +   P G  W+ +RR+ ++H+ S K  +    +R
Sbjct  127  LNS--PVFADRPVK-ESAYSLMFNRAIGFAPHGVYWRTLRRIASNHLFSPKQIKRAETQR  183

Query  161  TEEADNLVRYINNRSVKNRGNAFVVIDLRLAVRQYSGNVARKMMFGIRHFGKGSE-DGSG  219
               A  +V  +  +S    G  FV    R  ++  S N    MM  +  FG+  E D
Sbjct  184  RVIASQMVGLLEKQSTN--GVCFV----RELLKTASLN---NMMCSV--FGQEYELDQDH  232

Query  220  PGLEEIEHVESLFTVLTHLYAFALSDYVPWLRFLDLEGHEKVVSNAMRNVSKYNDPFVDE  279
              L E+  VE  + +L  L     +D++PWL   D +      S  +  V+++    V E
Sbjct  233  SELREL--VEEGYDLLGTL---NWTDHLPWLSEFDPQRIRTRCSALVPKVNRFVSRIVSE  287

Query  280  RLMQWRNGKMKEPQDFLDMFIIAKDTDGKPTLSDEEIKAQVTELMLATVDNPSNAAEWGM  339
                 RN     P+DF+D+ +      G   LSD +I A + E++    D  +   EW +
Sbjct  288  H----RNQTGDSPRDFVDVLL---SLHGSDHLSDPDIIAVLWEMIFRGTDTVAVLIEWIL  340

Query  340  AEMINEPSIMQKAVEEIDRVVGKDRLVIESDLPNLNYVKACVKEAFRLHPVAP-FNLPHM  398
            A ++  P I      E+D VVGK R V ESDL +L Y+ A VKE  R+HP  P  +   +
Sbjct  341  ARIVLHPDIQSTVQSELDLVVGKSRAVDESDLASLPYLTAVVKEVLRMHPPGPLLSWARL  400

Query  399  STTDTVVDGYFIPKGSHVLISRMGIGRNPSVWDKPHKFDPERHLSTNTCVDLN--ESDLN  456
            + TDT+VDG FIP G+  +++   I  +P VW  P +F PER ++    V+ +   SDL
Sbjct  401  AITDTIVDGRFIPAGTTAMVNMWAIAHDPHVWVDPLEFRPERFVTKEGEVEFSVLGSDLR  460

Query  457  IISFSAGRRGCMGVDIGSAMTYMLLARLIQGFTWLPVPGKNKIDISES-KNDLFMAKPLY  515
            +  F +GRR C G ++G        A L+  F W  V   N +D+SE  +    MA PL
Sbjct  461  LAPFGSGRRTCPGKNLGLTTVTFWTATLLHEFEW-GVSDGNGVDLSEKLRLSCEMANPLA  519

Query  516  A  516
            A
Sbjct  520  A  520
```

```
>XP_009140526.1 PREDICTED: cytochrome P450 705A5-like [Brassica rapa]
Length=530

 Score = 184 bits (466),  Expect = 2e-51, Method: Compositional matrix adjust.
 Identities = 130/458 (28%), Positives = 229/458 (50%), Gaps = 35/458 (8%)

Query  81    IRLANTHVIPVTSPRIAREILKKQDSVFATR-PLTMG--TEYCSRGYLTVAVEPQGEQWK  137
             +R+ +  ++ V+SP +A EI K  D+  + R P+ +     + S GY+     P G+ W+
Sbjct  79    LRIFHVPIVLVSSPTVAYEIFKAHDTNVSYRGPIAIDECIVFGSSGYIRA---PSGDYWR  135

Query  138   KMRRVVASHVTSKKSFQMMLQKRTEEADNLVRYINNRSVKNRGNAFVVIDLRLAVRQYSG  197
              M++++ +     ++ +     R  E +   R + ++++K          +RL
Sbjct  136   FMKKIIMAKALGPQALERTRGVRLVELERFHRNLLDKAMKKESVEVGEEAMRLV-----N  190

Query  198   NVARKMMFGIRHFGKGSEDGSGPGLEEIEHVESLFTVLTHLYAFALSDYVPWLRFLDLEG  257
             N   KM  G   F     DG      ++  +   FT L H +  A   + P    L+  G
Sbjct  191   NTLGKMSMG-SSFSVEDNDGG-----KVCELSVAFTSLCHKFCVAQVFHKP----LEKLG  240

Query  258   HEKVVSNAMRNVSKYNDPFVDERLMQWRNGKMKEPQ--DFLDMFIIA-KDTDGKPTLSDE  314
                +  + M    ++ +    E+++     K++E Q  +F+D  + + +  + +  ++ +
Sbjct  241   ISFLKKDVMEVSHRFEEHL--EKILAKYEEKVEEHQGAEFMDALLESYQGENAEYKMTRK  298

Query  315   EIKAQVTELMLATVDNPSNAAEWGMAEMINEPSIMQKAVEEIDRVVGKDRLVIESDLPNL  374
             +IKA   EL +   D+ S+   W MAE+IN P I+++  EEID VVGK+RLV E+DL NL
Sbjct  299   QIKALFAELFVGAGDSSSSTTRWAMAEIINNPKILERLREEIDSVVGKNRLVQETDLTNL  358

Query  375   NYVKACVKEAFRLHPVAPFNLPHMSTTDTVVDGYFIPKGSHVLISRMGIGRNPSVWDKPH  434
              Y++A VKEA RLHPV    +P        + G++IP+G+ + ++   I R+P  W+ P
Sbjct  359   PYLQAVVKEALRLHPVGAV-VPREFQEGCTIGGFYIPEGTSLAVNSYAIMRDPDSWEDPC  417

Query  435   KFDPERHLSTNTC---VDLNESDLNIISFSAGRRGCMGVDIGSAMTYMLLARLIQGFTWL  491
             KF PER L+++      +  E  L  ++F AGRRGC G ++GS      +  ++Q F W
Sbjct  418   KFKPERFLTSSRSWKEEERKEQALKFLAFGAGRRGCPGSNLGSTFVGTAVGVMVQCFDW-  476

Query  492   PVPGKNKIDISESKNDLF---MAKPLYAVATPRLAPHV  526
              + G +K+++ E+    F   +AKPL    +PR   H+
Sbjct  477   EIEG-DKVNMEEASGLRFFMALAKPLKCTPSPRNMNHL  513


>XP_009109371.1 PREDICTED: cytochrome P450 81F1-like [Brassica rapa]
Length=501

 Score = 183 bits (465),  Expect = 2e-51, Method: Compositional matrix adjust.
 Identities = 122/448 (27%), Positives = 220/448 (49%), Gaps = 30/448 (7%)

Query  62    PVFRWIHSLMKELNTDIACIRLANTHVIPVTSPRIAREILKKQ-DSVFATRPLTMGTEYC  120
             PV R  H L K  +  I  +RL     + ++S  +ARE      D V + RP  + ++Y
Sbjct  49    PVHRLFHGLAKT-HGPIFYLRLGTRRAVVISSSALARECFTGHNDVVVSNRPRFLTSKYI  107

Query  121   SRGYLTVAVEPQGEQWKKMRRVVASHVTSKKSFQMMLQKRTEEADNLVRYINNRSVKNRG  180
             +  Y T+A  P G+ W+ +R++ +  + S K     L  R EE   ++  ++  ++ N
Sbjct  108   AYNYTTIATTPYGDHWRNLRKICSLEIVSSKRLANFLHIRKEEIHRMLTRLSRDALINNE  167

Query  181   NAFVVIDLRLAVRQYSGNVARKMMFGIRHFGKGSEDGSGPGLEEIEHVESLFTVLTHLY-  239
                  ++L       + N   +M+ G  ++G+ + D +     E +  + L   +T
Sbjct  168   -----VELESLFYDLTFNNIVRMVTGKIYYGEDASDKA-----EADTFKKLIAYITSTSG  217

Query  240   AFALSDYVPWLRFL--DLEGHEKVVSNAMRNVSKYNDPFVDERLMQWRNGKMKEPQDFLD  297
             A    +Y+P+L+      E   K V  AM  +         +RL+    G  K+    ++
Sbjct  218   ARHPGEYLPFLKIFGRSFEKKVKAVGEAMDAIL--------QRLLDECRGN-KDGNTMVN  268

Query  298   MFIIAKDTDGKPTLSDEEIKAQVTELMLATVDNPSNAAEWGMAEMINEPSIMQKAVEEID  357
               +  +  D +   S+  IK  +  +M A  +  +   EW MA ++N P +++K   EID
Sbjct  269   HLLSLQQQDPE-YYSEVIIKGLMLGIMFAASETSAVTIEWAMASLLNHPELLEKLKLEID  327

Query  358   RVVGKDRLVIESDLPNLNYVKACVKEAFRLHPVAPFNLPHMSTTDTVVDGYFIPKGSHVL  417
               +G+DRL+ E+D+PNL Y++  V E  RL+P AP  +P ++  D  + GY +P+ + V+
Sbjct  328   EKIGQDRLIEETDIPNLPYLQNVVSETLRLYPAAPLLVPRLTVEDIKIGGYDVPRETMVM  387
```

```
Query  418  ISRMGIGRNPSVWDKPHKFDPERHLSTNTCVDLNESDLN-IISFSAGRRGCMGVDIGSAM  476
            ++   I R+P +W +P +F+P+R    N   +  + D+  +++F +GRR C G  + + +
Sbjct  388  VNAWSIHRDPELWTEPERFNPDRF---NGGGEGEKDDVRMLVTFGSGRRMCPGAGLANKI  444

Query  477  TYMLLARLIQGFTWLPVPGKNKIDISES  504
              + L  LIQ F W  V GK KID++E
Sbjct  445  VTLALGSLIQCFDWGRVNGK-KIDMTEG  471


>XP_009136769.1 PREDICTED: cytochrome P450 81F1 [Brassica rapa]
Length=499

 Score = 182 bits (462),  Expect = 4e-51, Method: Compositional matrix adjust.
 Identities = 135/474 (28%), Positives = 221/474 (47%), Gaps = 36/474 (8%)

Query  56   ILGRNKPVFRWIHSLMKELNTD---IACIRLANTHVIPVTSPRIAREILKKQ-DSVFATR  111
            ILG +  +   +H L + L+     I  +R  +   + ++S  +A E    Q D + + R
Sbjct  41   ILGHHHLLKPPVHRLFQRLSKTHGPIFSLRFGSRRTVVISSSSLATECFTGQNDVLLSNR  100

Query  112  PLTMGTEYCSRGYLTVAVEPQGEQWKKMRRVVASHVTSKKSFQMMLQKRTEEADNLVRYI  171
            P  +  +Y +  Y TV   P G+ W+ +RR+ +  + S        L  R +E   ++  +
Sbjct  101  PCFLTAKYVAYNYTTVGTSPYGDHWRNLRRICSLEILSSNRLTNFLHIRKDEIRRMLTRL  160

Query  172  NNRSVKNRGNAFVVIDLRLAVRQYSGNVARKMMFGIRHFGKGSEDGSGPGLEEIEHVESL  231
            + R V N+      I+L   +   + N   +M+ G R++G    +      EE    + L
Sbjct  161  S-REVANKE-----IELEPLLSDLTFNNIVRMVTGKRYYGDEVHNE-----EEANLFKKL  209

Query  232  FTVLTHLY-AFALSDYVPWLRFL--DLEGHEKVVSNAMRNVSKYNDPFVDERLMQWRNGK  288
               +     A    DY+P+L+      E   K V  AM       D  +   L + R  +
Sbjct  210  VADVNDCSGARHPGDYLPFLKIFGGSFEKKVKAVGEAM-------DDILQRLLDECR--R  260

Query  289  MKEPQDFLDMFIIAKDTDGKPTLSDEEIKAQVTELMLATVDNPSNAAEWGMAEMINEPSI  348
             K+    ++  +  +  + +   +D  IK  +  +M+A  D  +   EW MA ++N P
Sbjct  261  DKDGNTMVNHLLTLQQQEPE-YYTDVTIKGLMLGMMIAGTDTSAVTLEWAMACLLNHPES  319

Query  349  MQKAVEEIDRVVGKDRLVIESDLPNLNYVKACVKEAFRLHPVAPFNLPHMSTTDTVVDGY  408
            M+KA +EI   +G+DRL+ E DL NL Y++  V E FRL+P AP  +P     D  V GY
Sbjct  320  MEKAKQEIHEKIGQDRLIDEPDLANLPYLQNIVSETFRLYPAAPLLVPRSPMEDIKVGGY  379

Query  409  FIPKGSHVLISRMGIGRNPSVWDKPHKFDPERHLSTNTCVDLNESDLNIISFSAGRRGCM  468
             +P+G+ V+++   I R+PS+W +P KF PER  S    V        ++ F  GRR C
Sbjct  380  DVPRGTMVMVNAWAIHRDPSLWSEPEKFKPERFNSGGEDVH------KLMPFGNGRRSCP  433

Query  469  GVDIGSAMTYMLLARLIQGFTWLPVPGKNKIDISESKNDLFMAK-PLYAVATPR  521
            G  +G  +  + L  LIQ F W  V G+ KID++E+       K PL A+   R
Sbjct  434  GAGLGQRIVTLALGSLIQCFDWEKVNGE-KIDMTETPGMAMRKKEPLRALCRSR  486


>XP_009103856.1 PREDICTED: cytochrome P450 71B5 [Brassica rapa]
Length=500

 Score = 182 bits (462),  Expect = 4e-51, Method: Compositional matrix adjust.
 Identities = 130/463 (28%), Positives = 235/463 (51%), Gaps = 38/463 (8%)

Query  40   LPPGPKSWPLIGNLPEILGRNKPVFRWIHSLMKELNTDIACIRLANTHVIPV---TSPRI  96
            LPPGP   P+IGNL +         R++H  + +++ +   + L +  V+PV   +S
Sbjct  28   LPPGPTGLPIIGNLHQF-------GRFLHKSLHKISQEYGPVMLLHFGVVPVIIVSSKEG  80

Query  97   AREILKKQDSVFATRPLTMGTEYCSRGYLTVAVEPQGEQWKKMRRVVASHVTSKKSFQMM  156
            A E+LK  D    +RP T+GT   +  +  V   P GE W++MR+++   + S+K  +
Sbjct  81   AEEVLKTHDLETCSRPKTVGTGLFTYNFKDVGFAPFGENWREMRKIMVLELFSQKKLKSF  140

Query  157  LQKRTEEADNLVRYINNRSVKNRGNAFVVIDLRLAVRQYSGNVARKMMFGIRHFGKGSED  216
               R EE++ LV+ ++N + +   ++   +DLR  +  Y+ ++  ++ FG        ++
Sbjct  141  RYIREEESELLVKKVSNSANEKPTSS---VDLRKVIFSYAASIICRLAFG--------QN  189

Query  217  GSGPGLEEIEHVESL-FTVLTHLYAFALSDYVPWLRFLD-LEGHEKVVSNAMRNVSKYND  274
                   ++E VE L     T+L + +L+D+ P    +D + G    ++ A   ++ + +
Sbjct  190  FHECDFVDMERVEELVLESETNLGSLSLADFFPAGWLIDRISGQHSRLNKAFAKLTTFFE  249
```

```
Query  275  PFVDERLMQWRNGKMKEPQDFLDMFIIAKDTDGKPT------LSDEEIKAQVTELMLATV  328
              +D+ L      K  +PQD  D+  +  D   KP       ++D+ ++  ++++ LA V
Sbjct  250  HVIDDHL------KTGQPQDHSDIISVMLDMINKPNKVGSFQVTDDHLRGVMSDVFLAGV  303

Query  329  DNPSNAAEWGMAEMINEPSIMQKAVEEIDRVVGKDR-LVIESDLPNLNYVKACVKEAFRL  387
            +  +    W M E+   P +M+K  EEI   +G ++  + E DL  + Y+K  ++E FRL
Sbjct  304  NAGAITMIWTMTELSRHPVVMKKLQEEIRATLGPNKERITEEDLEKVEYLKLVIEETFRL  363

Query  388  HPVAPFNLPHMSTTDTVVDGYFIPKGSHVLISRMGIGRNPSVWDKPHKFDPERHLSTNTC  447
            HP AP  LP ++ +D  + GY IPK + + I+   IGR+P  + KP +F PER +   +
Sbjct  364  HPPAPLLLPRLTMSDIKIQGYNIPKNTMIQINTYTIGRDPKNYTKPEEFIPERFV--DNP  421

Query  448  VDLNESDLNIISFSAGRRGCMGVDIGSAMTYMLLARLIQGFTW  490
            ++       ++ F AGRR C G+  G AM  + L  L+  F W
Sbjct  422  IEYKGKHFELLPFGAGRRVCPGMATGIAMVELCLLSLLYFFDW  464
```

>XP_009140379.1 PREDICTED: cytochrome P450 71B2-like [Brassica rapa]
Length=502

```
 Score = 182 bits (462),  Expect = 5e-51, Method: Compositional matrix adjust.
 Identities = 145/496 (29%), Positives = 238/496 (48%), Gaps = 35/496 (7%)

Query  38   LSLPPGPKSWPLIGNLPEILGRNKPVFRWIHSLMKELNTDIACIRLANTHVIPVTSPRIA  97
             +LPP P S P+IGNL  + G      R  H+L K+    +  +RL +  V+ ++S   A
Sbjct  29   FNLPPSPSSLPVIGNLHHLAGLPH---RCFHNLSKKYGP-VMLLRLGSVPVVVISSSEAA  84

Query  98   REILKKQDSVFATRPLTMGTEYCSRGYLTVAVEPQGEQWKKMRRVVASHVTSKKSFQMML  157
              +LK  D    +RP T+GT   S G+  +A+   GE W+KMR++    + S K  Q
Sbjct  85   EAVLKAHDLECCSRPKTLGTGKFSYGFKDIALSQYGEYWRKMRKLAVIELFSLKRVQSFR  144

Query  158  QKRTEEADNLVRYINNRSVKNRGNAFVVIDLRLAVRQYSGNVARKMMFGIRHFGKGSEDG  217
              R EE   +V+ ++  +++         +DL       + ++  ++  G ++F   +EDG
Sbjct  145  YIREEEVGLVVKKVSESALRQSP-----VDLSKTFFSLTASIICRVALG-QNF---NEDG  195

Query  218  SGPGLEEIEHVESLFTVLTH-LYAFALSDYVPWL--RFLD-LEGHEKVVSNAMRNVSKYN  273
                +   E ++ L T  T  +  F   D+ P    RFLD L    K ++     +  +
Sbjct  196  F---VINQERIQELITEATEAIGTFTFYDFFPGALGRFLDWLFQRHKKINKVFEELDAFY  252

Query  274  DPFVDERL-MQWRNGKMKEPQDFLDMFIIAKDTDGKP---TLSDEEIKAQVTELMLATVD  329
               +D+ L ++ R    K+P D + + +   D  G      L+ + +KA + +L LA VD
Sbjct  253  QHVIDDHLTLEGR----KDP-DIVSLMLDMIDKQGNEDSFKLNIDNVKAILMDLFLAGVD  307

Query  330  NPSNAAEWGMAEMINEPSIMQKAVEEIDRVVG-KDRLVIESDLPNLNYVKACVKEAFRLH  388
              +    W M+E++  P  ++KA E+I   +G K  ++ E DL  ++Y+   +KE FRLH
Sbjct  308  TSAVTMIWAMSELVRNPRALKKAQEKIRTTLGEKKEIITEDDLGKVDYLTLIIKETFRLH  367

Query  389  PVAPFNLPHMSTTDTVVDGYFIPKGSHVLISRMGIGRNPSVWDKPHKFDPERHLSTNTCV  448
            P  PF LP  + +   + GY IP  + + I+   IGR+P  W  P +F PER   T++ V
Sbjct  368  PALPFILPRETMSHVKIQGYDIPPKTQIQINVWTIGRDPKRWTDPKEFIPERF--TDSSV  425

Query  449  DLNESDLNIISFSAGRRGCMGVDIGSAMTYMLLARLIQGFTWLPVPGKN--KIDISESKN  506
            D       ++ F +GRR C  + +G A   + L  L+  F W    G N   ID+ E+ N
Sbjct  426  DFRGQHFELLPFGSGRRMCPAMPMGVATVELGLMNLLYFFDWELPDGINFGDIDMEETGN  485

Query  507  DLFMAK-PLYAVATPR  521
               + K PL  V   R
Sbjct  486  ISIVKKVPLQLVPLQR  501
```

>XP_009110600.1 PREDICTED: cytochrome P450 71B2-like [Brassica rapa]
Length=502

```
 Score = 181 bits (460),  Expect = 1e-50, Method: Compositional matrix adjust.
 Identities = 138/491 (28%), Positives = 238/491 (48%), Gaps = 36/491 (7%)

Query  29   TMLISPTRN--LSLPPGPKSWPLIGNLPEILGRNKPVFRWIHSLMKELNTDIACIRLANT  86
            ++ +  T+N   +LPP P S+P+IGNL  + G      R  H+L  +    +  +RL +
```

```
Sbjct  18   SIFLKKTKNSKFNLPPSPSSFPIIGNLHHLAGLPH---RCFHNLSIKYGP-VVLLRLGSV  73

Query  87   HVIPVTSPRIAREILKKQDSVFATRPLTMGTEYCSRGYLTVAVEPQGEQWKKMRRVVASH  146
             V+ ++S   A  +LK  D    +RP T+GT   S G+  +     G  W++MR++
Sbjct  74   PVVVISSSEAAEAVLKTHDLECCSRPKTVGTGKLSYGFKDITFSQYGAYWREMRKLAVIE  133

Query  147  VTSKKSFQMMLQKRTEEADNLVRYINNRSVKNRGNAFVVIDLRLAVRQYSGNVARKMMFG  206
            + S K  Q     R +E   +V+ ++  S+         +DL       + ++  ++  G
Sbjct  134  LFSLKKVQSFRYIREDEVGFVVKKVSEASLTQSP-----VDLSKTFFTLTASIICRVALG  188

Query  207  IRHFGKGSEDGSGPGLEEIEHVESLFTVLT-HLYAFALSDYVPWL--RFLDL--EGHEKV  261
             ++F +           + E +E L T  T  L  F  SD+ P +  RFLDL  + H+++
Sbjct  189  -QNFHESD------FFIDQEKIEELVTEATVALGDFTFSDFFPGVFGRFLDLLFQRHKRI  241

Query  262  VSNAMRNVSKYNDPFVDERLMQWRNGKMKEPQDFLDMFIIAKDTDGKP---TLSDEEIKA  318
             S     +  +    +D+ L      + ++ QD + M +   D  G      L+ + +KA
Sbjct  242  -SKVFEELDAFYQHVIDDHL----KPEGRKNQDIVSMMLDMIDEQGDDDSFKLNMDNVKA  296

Query  319  QVTELMLATVDNPSNAAEWGMAEMINEPSIMQKAVEEIDRVVGKDR-LVIESDLPNLNYV  377
             + ++ LA +D  +    W MAE++  P +M+KA E I   +G +R  + E D+  ++Y+
Sbjct  297  ILMDVFLAGIDTSAVTMIWAMAELVKNPRVMKKAQENIRTTLGLNRERITEDDIGKVDYL  356

Query  378  KACVKEAFRLHPVAPFNLPHMSTTDTVVDGYFIPKGSHVLISRMGIGRNPSVWDKPHKFD  437
            K  VKE FRLHP  PF +P  + +   + GY IP  + + I+   IGR+P  W  P +F+
Sbjct  357  KLIVKEIFRLHPALPFIIPRETMSHVKIQGYDIPPKTQIQINVWTIGRDPERWTDPEEFN  416

Query  438  PERHLSTNTCVDLNESDLNIISFSAGRRGCMGVDIGSAMTYMLLARLIQGFTW-LPVPGK  496
            PER   T++ VD       ++ F +GRR C  + +G A   + L  L+  F W LP   K
Sbjct  417  PERF--TDSSVDFRGQHYELLPFGSGRRMCPAMPMGVANVELALMNLLYFFDWGLPDGMK  474

Query  497  -NKIDISESKN  506
              ++D+ E+ N
Sbjct  475  VGELDMEEAGN  485


>NP_001306250.1 cytochrome P450 81F1-like [Brassica rapa]
Length=501

 Score = 181 bits (459),  Expect = 1e-50, Method: Compositional matrix adjust.
 Identities = 121/447 (27%), Positives = 219/447 (49%), Gaps = 27/447 (6%)

Query  62   PVFRWIHSLMKELNTDIACIRLANTHVIPVTSPRIAREILKKQ-DSVFATRPLTMGTEYC  120
            PV R  H L K  +  I  +RL     + ++S  +A+E      D V + RP  + ++Y
Sbjct  48   PVHRLFHRLAKA-HGPIFYLRLGTRRAVVISSSALAKECFTGHNDVVVSNRPRFLTSKYI  106

Query  121  SRGYLTVAVEPQGEQWKKMRRVVASHVTSKKSFQMMLQKRTEEADNLVRYINNRSVKNRG  180
            +  Y T+A  P G+ W+ +R++ +  + S K     L  R EE   ++  ++  ++ N
Sbjct  107  AYNYTTIATTPYGDHWRNLRKICSLEIVSSKRLANFLHIRKEEIHRMLTRLSRDALINNE  166

Query  181  NAFVVIDLRLAVRQYSGNVARKMMFGIRHFGKGSEDGSGPGLEEIEHVESLFTVLTHLY-  239
                 ++L       + N   +M+ G  ++G+ + D +     E +  + L   +T
Sbjct  167  -----VELESLFYDLTFNNIVRMVTGKIYYGEDASDKA-----EADTFKKLIAYITSTSG  216

Query  240  AFALSDYVPWLRFL--DLEGHEKVVSNAMRNVSKYNDPFVDERLMQWRNGKMKEPQDFLD  297
            A    +Y+P+L+      E   K V  AM  +         +RL+    G  K+    ++
Sbjct  217  ARHPGEYLPFLKIFGRSFEKKVKAVGEAMDAIL--------QRLLDECRGN-KDGNTMVN  267

Query  298  MFIIAKDTDGKPTLSDEEIKAQVTELMLATVDNPSNAAEWGMAEMINEPSIMQKAVEEID  357
              +  +  D +   S+  IK  +  +M A  +  +   EW MA ++N P +++K   EID
Sbjct  268  HLLSLQQQDPE-YYSEVIIKGLMLGIMFAASETSAVTIEWAMASLLNHPELLEKLKLEID  326

Query  358  RVVGKDRLVIESDLPNLNYVKACVKEAFRLHPVAPFNLPHMSTTDTVVDGYFIPKGSHVL  417
              +G+DRL+ E+D+PNL Y++  V E FRL+P AP  +P ++  D  V GY +P+ + V+
Sbjct  327  EKIGQDRLIEETDIPNLPYLQNVVSETFRLYPAAPLLVPRLTEEDIKVGGYDVPRETMVM  386

Query  418  ISRMGIGRNPSVWDKPHKFDPERHLSTNTCVDLNESDLNIISFSAGRRGCMGVDIGSAMT  477
            ++   I R+P +W +P +F+P+R        D ++    +I+F +GRR C G  + + +
Sbjct  387  VNAWTIHRDPELWTEPERFNPDRFNGERGEGDKDDVR-TLITFGSGRRMCPGAGLANKIV  445
```

```
Query  478  YMLLARLIQGFTWLPVPGKNKIDISES  504
             + L  LIQ F W  V G+ +ID++E
Sbjct  446  TLALGSLIQCFDWGRVNGE-EIDMTEG  471


>XP_009137084.1 PREDICTED: cytochrome P450 81D1 [Brassica rapa]
Length=513

 Score = 181 bits (458),  Expect = 2e-50, Method: Compositional matrix adjust.
 Identities = 137/497 (28%), Positives = 238/497 (48%), Gaps = 33/497 (7%)

Query  37   NLSLPPGPK-SWPLIGNLPEILGR---NKPVFRWIHSLMKELNTDIACIRLANTHVIPVT  92
            N +LPP P+  +P+IG+L  +L +   ++ + R  HSL       +  +RL +   + V+
Sbjct  27   NKNLPPSPRVCFPIIGHL-HLLKQPLLHRTLLRLSHSL-----GPVFSLRLGSRLAVIVS  80

Query  93   SPRIARE-ILKKQDSVFATRP-LTMGTEYCSRGYLTVAVEPQGEQWKKMRRVVASHVTSK  150
            SP  A E  L K D V A RP  TMG +Y +  Y ++   P G+ W+ +RR+ A  V S
Sbjct  81   SPAAAEECFLTKNDIVLANRPRFTMG-KYVAYDYTSMVTAPYGDHWRNLRRITALEVFST  139

Query  151  KSFQMMLQKRTEEADNLVRYINNRSVKNRGNAFVVIDLRLAVRQYSGNVARKMMFGIRHF  210
                   +   +E   L++ +   SV+        ++LR  +   + NV  +MM G R
Sbjct  140  HRLNGSAEILQDEVKRLLQKLYGLSVQRPAK----VELRTLLTGLTLNVIMRMMTGKRFC  195

Query  211  GKGSEDGSGPGLEEIEHVESLFTVLTHLYAFALSDYVPWLRFLDLEGHEKVVSNAMRNVS  270
                ED  G     +E  E +  +L    A   +D++P L++ D + + K        +
Sbjct  196  ---EEDEGGKEKISLEFQELVAEILELSSAGNPADFLPALQWYDYKDYIKRAKKVGEKMD  252

Query  271  KYNDPFVDERLMQWRNGKMKEPQDFLDMFIIAKDTDGKPTLSDEEIKAQVTELMLATVDN  330
                 F+DE   +   G+++     +   + +++ +     +D  IK  +  +++   D
Sbjct  253  SLLQGFLDEH--RANKGRLEFTNTMIAHLLDSQEKEPH-YYNDVTIKGLILMMVIGGTDT  309

Query  331  PSNAAEWGMAEMINEPSIMQKAVEEIDRVV-----GKDRLVIESDLPNLNYVKACVKEAF  385
             +   EW M+ ++N P +++   + ID  +         RL+ E DL N+NY+   V E
Sbjct  310  SALTVEWAMSNLLNHPQVLETTRQNIDTHIVPSSSSNRRLLKEDDLVNMNYLNCVVSETL  369

Query  386  RLHPVAPFNLPHMSTTDTVVDGYFIPKGSHVLISRMGIGRNPSVWDKPHKFDPERHLSTN  445
            RL PVAP  +PH S++D V+ G+ +P+ + VL++   I R+PSVWD P  F PER    +
Sbjct  370  RLCPVAPLMVPHFSSSDCVIGGFDVPRDTIVLVNLWAIHRDPSVWDDPTSFKPERFEDRD  429

Query  446  TCVDLNESDLNIISFSAGRRGCMGVDIGSAMTYMLLARLIQGFTWLPVPGKNKIDISESK  505
                L + +  ++ F  GRR C G+ + + +  ++L  +IQ F W    G   +D++E
Sbjct  430  ---QLGQYNGKMMPFGLGRRVCPGLGLANRVVGLVLGSMIQCFEWESGSG-GPVDMTEGP  485

Query  506  N-DLFMAKPLYAVATPR  521
               +  A+PL     PR
Sbjct  486  GLSMPKAEPLVVTCRPR  502


>XP_009119678.1 PREDICTED: cytochrome P450 78A6 [Brassica rapa]
Length=540

 Score = 181 bits (458),  Expect = 3e-50, Method: Compositional matrix adjust.
 Identities = 134/472 (28%), Positives = 231/472 (49%), Gaps = 31/472 (7%)

Query  36   RNLSLPPGPKSWPLIGNLPEILGRNKPVFRWIHSLMKELNTD-IACIRLANTHVIPVTSP  94
            +N ++ PGP+ +PL+G++   L  ++   + I ++    N   +    L +T V+    P
Sbjct  73   KNKNIIPGPRGFPLVGSMS--LRSSRVAHQRIAAVAAMRNAKRLMAFSLGDTKVVVTCHP  130

Query  95   RIAREILKKQDSVFATRPLTMGTEYCSRGYLTVAVEPQGEQWKKMRRVVASHVTSKKSFQ  154
             +A+EIL    SVFA RP+   T Y       +   P G  W+ +RR+ ++H+ + K  +
Sbjct  131  DVAKEIL--NSSVFADRPVD-ETAYGLMFNRAMGFAPNGAYWRTLRRLGSNHLFNPKQIK  187

Query  155  MMLQKRTEEADNLVRYINNRSVKNRGNAFVVIDLRLAVRQYSGNVARKMMFGIRHFGKGS  214
               ++R   A  +V    N   +N   AF V DL   ++  S +    ++FG ++  + +
Sbjct  188  RSEEQRRVIATQMV----NVFARNAETAFGVRDL---LKTASLSNMMGLVFGKQYELESN  240

Query  215  EDGSGPGLEEIEHVESLFTVLTHLYAFALSDYVPWLRFLDLEGHEKVVSNAMRNVSKYND  274
             +     L+ +  VE  + +L  L     +D++PWL  LD +      S  +
Sbjct  241  NNVESECLKGL--VEEGYDLLGTL---NWTDHLPWLAGLDFQQVRFRCSQLV--------  287
```

```
Query  275  PFVDERLMQWRNGKMKEPQDFLDMFIIAKDTDGKPTLSDEEIKAQVTELMLATVDNPSNA  334
            P V++ L +  +        FLD  ++         LS+ ++ A + E++    D  +
Sbjct  288  PKVNQLLSRIIHEHRALTCSFLD--VLQHSLHDSEKLSESDMVAVLWEMIFRGTDTVAVL  345

Query  335  AEWGMAEMINEPSIMQKAVEEIDRVVGKDRLVIESDLPNLNYVKACVKEAFRLHPVAP-F  393
             EW +A ++  P +     +E+DRVVG+ + V ESDLP+L Y+ A +KE  RLHP  P
Sbjct  346  IEWVLARIVMHPKVQSTVHDELDRVVGRSKAVDESDLPSLTYLTAMIKEVLRLHPPGPLL  405

Query  394  NLPHMSTTDTVVDGYFIPKGSHVLISRMGIGRNPSVWDKPHKFDPERHLSTNTCVDLN--  451
            +   +S TDT VDGY +P G+  +++   I R+P VW+ P +F PER ++     + +
Sbjct  406  SWARLSITDTTVDGYHVPAGTTAMVNMWAIARDPHVWENPLEFKPERFVAKEGEAEFSVL  465

Query  452  ESDLNIISFSAGRRGCMGVDIGSAMTYMLLARLIQGFTWLPVPGKNKIDISE  503
             SDL +  F +G+R C G ++G A     +A L+  F WL     N  D+SE
Sbjct  466  GSDLRLAPFGSGKRVCPGKNLGLATVSFWVATLLHEFEWLSSVNANPPDLSE  517


>XP_009126913.1 PREDICTED: cytochrome P450 71B2-like [Brassica rapa]
Length=512

 Score = 180 bits (456),  Expect = 4e-50, Method: Compositional matrix adjust.
 Identities = 132/487 (27%), Positives = 224/487 (46%), Gaps = 29/487 (6%)

Query  32   ISPTRNLSLPPGPKSWPLIGNLPEILGRNKPVFRWIHSLMKELNTDIACIRLANTHVIPV  91
            I   +   LPP P + P+IGNL ++ G      R  H L  +    +  + L     + +
Sbjct  26   IKENKTFYLPPSPPTLPIIGNLHQLSGLPH---RCFHHLSIKYGP-VVLLHLGFVPTVVI  81

Query  92   TSPRIAREILKKQDSVFATRPLTMGTEYCSRGYLTVAVEPQGEQWKKMRRVVASHVTSKK  151
            +S   A E+L+  D    +RP T+ T   S G+  ++    GE W++MR++    + S K
Sbjct  82   SSSEAAEEVLRTNDLGCCSRPKTVATGKLSYGFKDISFAQYGEYWREMRKLAVVELFSLK  141

Query  152  SFQMMLQKRTEEADNLVRYINNRSVKNRGNAFVVIDLRLAVRQYSGNVARKMMFGIRHFG  211
                    R EE   +V+ ++  S+K        +DL       + ++  ++  G ++F
Sbjct  142  KVHSFKNIREEEVGFMVKKVSESSLKQSP-----VDLNKTFFSLTASIICRVALG-QNFN  195

Query  212  KGSEDGSGPGLEEIEHVESLFTVLTHLYAFALSDYVP--WLRFLD-LEGHEKVVSNAMRN  268
            +     SG  +E+    E +   L  L +F  SD  P    R LD L G  K ++
Sbjct  196  E-----SGFVIEQDRIEELVRDALVALGSFTCSDVFPGGLGRLLDWLFGGHKRINKVFEE  250

Query  269  VSKYNDPFVDERLMQWRNGK--MKEPQDFLDMFIIAKDTDGKP---TLSDEEIKAQVTEL  323
            +  +    +D+ L     GK  +    D + + +   +  GK     L+   IK  +  +
Sbjct  251  LDAFYQHVIDDHLKPEAAGKKAIDSTADIVALLLDMMEKQGKKDYFKLNISNIKGVLMNI  310

Query  324  MLATVDNPSNAAEWGMAEMINEPSIMQKAVEEIDRVVGKDR-LVIESDLPNLNYVKACVK  382
             LA +D  +    W M E++  P +M++A EEI   +G ++  + E D+  + Y+K  +K
Sbjct  311  FLAGIDTGAITMIWAMTELVRNPKVMRRAQEEIRTTLGLNKEKITEEDVEKVGYLKLIIK  370

Query  383  EAFRLHPVAPFNLPHMSTTDTVVDGYFIPKGSHVLISRMGIGRNPSVWDKPHKFDPERHL  442
            E FRLHP AP  LP  + +   ++GY IP  + + ++    IGR+P  W  P +F PER
Sbjct  371  ETFRLHPAAPLLLPRETMSHVKINGYDIPPKTQIQLNVWAIGRDPRRWTDPGEFIPERF-  429

Query  443  STNTCVDLNESDLNIISFSAGRRGCMGVDIGSAMTYMLLARLIQGFTW-LP--VPGKNKI  499
              N+ VD      +++ F +GRR C G+ +  A   + L  L+  F W LP  +  +  I
Sbjct  430  -ANSSVDFRGQHFDLLPFGSGRRSCPGMSMALASVELGLLSLLYFFDWKLPEGMVSEEDI  488

Query  500  DISESKN  506
            DI E+ N
Sbjct  489  DIEEAGN  495


>XP_009103433.1 PREDICTED: cytochrome P450 81D1-like isoform X2 [Brassica rapa]
Length=466

 Score = 179 bits (453),  Expect = 4e-50, Method: Compositional matrix adjust.
 Identities = 135/482 (28%), Positives = 218/482 (45%), Gaps = 56/482 (12%)

Query  39   SLPPGPKSWPLIGNLPEILGRNKPVFRWIHSLMKELNTDIACIRLANTHVIPVTSPRIAR  98
            +LPP P  +P+IG+L  +     PV R +    + L   +  +RL +   + VTS   A
```

```
Sbjct  26   NLPPSPVGFPVIGHLHLL---KDPVHRCLRDRSQSLGP-VFSLRLGSCRAVVVTSASAAE  81

Query  99   EILKKQ-DSVFATRPLTMGTEYCSRGYLTVAVEPQGEQWKKMRRVVASHVTSKKSFQMML  157
            E L  + D VFA RP+T    Y       V+V P G+ W+ +RR+  + + S    +
Sbjct  82   EFLSHENDVVFANRPITTMYAYVLYSNTGVSVAPYGDHWRHLRRICTTEIFSAARLRESF  141

Query  158  QKRTEEADNLVRYINNRSVKNRGNAFVVIDLRLAVRQYSGNVARKMMFGIRHFGKGSEDG  217
            + R +E  ++++ I+  ++  RGN  V ++LR  +  ++ NV  +M+ G R++G   ED
Sbjct  142  EIRRDEVRSMLQTIHAATL--RGNNSVRVELRPLLSGFTLNVIMRMVAGKRYYG---EDN  196

Query  218  SGPGLEEIEHVESLFTVLTHLYAFALSDYVPWLRFLDLEGHEKVVSNAMRNVSKYNDPFV  277
            +     E + V  L +    L  F                                   V
Sbjct  197  A-----EAKAVSELISETFELGGFTYE-------------------------------LV  220

Query  278  DERLMQWRNGKMKEPQDFLDMFIIAKDTDGKPTLSDEEIKAQVTELMLATVDNPSNAAEW  337
            DE   +   GK +     +   +  +++  + + +D+ IK  V  ++ A  D  S   EW
Sbjct  221  DEH--RGNRGKTEFKNTMITHLLTLQESQPE-SYTDQIIKGLVLVMLFAGSDTTSVTLEW  277

Query  338  GMAEMINEPSIMQKAVEEIDRVVGKDR-LVIESDLPNLNYVKACVKEAFRLHPVAPFNLP  396
             MA ++N P ++ K   E++ +V ++R L+ ESD     Y+   + E  RL P AP  +P
Sbjct  278  AMANLLNHPDVLMKVKTELNNLVSRERRLMEESDTSTCTYLDNVISETLRLCPAAPLLVP  337

Query  397  HMSTTDTVVDGYFIPKGSHVLISRMGIGRNPSVWDKPHKFDPERHLSTNTCVDLNESDLN  456
            H S+ D  V GY IP+G+ + I+   I R+P +WD+P  F PER  S
Sbjct  338  HASSGDCKVAGYDIPRGTWLFINAWAIQRDPKMWDEPEVFKPERFDSEG----WKTQHGK  393

Query  457  IISFSAGRRGCMGVDIGSAMTYMLLARLIQGFTWLPVPGKNKIDISESKNDLFMAKPLYA  516
             + F  GRR C G+ +   +  + L  LIQ F W        +D+SE K  L M K L
Sbjct  394  FLPFGMGRRACPGMGLAQLILSLALGSLIQCFDW-ERDEDVAVDMSEGKG-LTMPKALSL  451

Query  517  VA  518
            VA
Sbjct  452  VA  453


>XP_009145675.1 PREDICTED: cytochrome P450 705A20-like [Brassica rapa]
Length=513

 Score = 179 bits (455),  Expect = 5e-50, Method: Compositional matrix adjust.
 Identities = 145/534 (27%), Positives = 251/534 (47%), Gaps = 50/534 (9%)

Query  5    TPMLAFIIGLLLLALTMKRKEKKKTMLISPTRNLSLPPGPKSWPLIGNLPEILGRNKPVF  64
            T +L     LL  +L  K+ ++       P      PP P S P+IG+L  +L
Sbjct  14   TFILLCFFSLLCYSLFFKKTKE-------PRVGCDFPPSPPSLPVIGHLHLLLST-----  61

Query  65   RWIHSLMKELNTDIA---CIRLANTHVIPVTSPRIAREILKKQDSVFATRPLTMGTEYCS  121
              +H  +++++++      +R+ NT +I V+S  +A EI +  D   ++R +    +
Sbjct  62   -LVHKSLQKISSNYGPFLHLRIFNTSIILVSSASVAYEIFRAHDVNVSSRGVPAVDDSLL  120

Query  122  RGYLTVAVEPQGEQWKKMRRVVASHVTSKKSFQMMLQKRTEEADNLVRYINNRSVKNRGN  181
             G   V   P G+ WK M++++ + +   ++ +   Q R   AD + R+     + N+
Sbjct  121  FGSSGVLNAPCGDYWKFMKKLMVTKLLGPQAQE---QSRGIRADEINRFYGK--LLNKAR  175

Query  182  AFVVIDLRLAVRQYSGNVARKMMFGIRHFGKGSEDGSGPGLEEIEHVESLFTVLTHLYAF  241
                +D+         N+   M  G R F +  EDG      E E ++ L T  + L
Sbjct  176  KKESVDVGKEAMNLVNNIMCMMSMG-RRFSE--EDG------EAERLKDLVTEWSGLIKR  226

Query  242  ALSDYVPWLRFLDLEGHEKVVSNAMRNVSKYNDPFVDERLMQWRNGKMKEP-----QDFL  296
                   +L  L     EK+  +  +N         DE L +   G  +EP     +D +
Sbjct  227  M------FLAVLFRRQLEKIGISLFKNEIMRVSNRCDEMLERVLVGHKEEPDKDQGKDMM  280

Query  297  DMFIIA-KDTDGKPTLSDEEIKAQVTELMLATVDNPSNAAEWGMAEMINEPSIMQKAVEE  355
            D+ + A +D   +  ++   IKA   EL+   +D  S    W MAE+IN P++++K  +E
Sbjct  281  DVLLSAYEDKKAEYKITMNHIKAFFVELLFGAIDTSSTTILWAMAEIINNPNVLEKLRKE  340

Query  356  IDRVVGKDRLVIESDLPNLNYVKACVKEAFRLHPVAPFNLPHMSTTDTVVDGYFIPKGSH  415
            +D +VGK RL+ E+D+PNL Y++A VKE  RLHP  P  +P     +  + G++IP+ +
Sbjct  341  LDSIVGKTRLIQETDIPNLPYLQAVVKETLRLHPPGPL-VPREFQKECEIGGFYIPEKTR  399
```

```
Query  416  VLISRMGIGRNPSVWDKPHKFDPERHLSTNTCVDLNESD---LNIISFSAGRRGCMGVDI  472
            ++++   I R+P +W+ P KF PER L+++     +E     L  + F +GRRGC G  +
Sbjct  400  LVVNVYDIMRDPDLWEDPLKFMPERFLASSKSGQEDERKEKILKYLPFGSGRRGCPGSAL  459

Query  473  GSAMTYMLLARLIQGFTWLPVPGKNKIDISESKND--LFMAKPLYAVATPRLAP  524
            G  +    +  ++ GF W  + G +K+++ E      L MA PL      R  P
Sbjct  460  GYIVVGTAIGVIVHGFEW-RIDG-DKVNMEEVMEGVILTMAHPLKFTPITRYVP  511


>XP_009104755.1 PREDICTED: cytochrome P450 98A8-like [Brassica rapa]
Length=498

 Score = 179 bits (454),  Expect = 6e-50, Method: Compositional matrix adjust.
 Identities = 140/500 (28%), Positives = 233/500 (47%), Gaps = 42/500 (8%)

Query  39   SLPPGPKSWPLIGNLPEILGRNKPVFRWIHSLMK--ELNTDIACIRLANTHVIPVTSPRI  96
            ++PPGPK   L+GN+ ++    KP   W HS  +  E    I  +       + V+S  +
Sbjct  27   NIPPGPKPRFLLGNIHQL----KP--HWTHSFSEWSETYGPIISVWFGTNLSVVVSSSDL  80

Query  97   AREILKKQDSVFATRPLTMGTEYCSRGYLTVAVEPQGEQWKKMRRVVASHVTSKKSFQMM  156
            A+++LK  D     R             +     P    + K+R++    + S KS +
Sbjct  81   AKQVLKDNDHQLCNRERVAKMTQSGNDLVWSDYSPH---YVKLRKLCTLELFSLKSIESF  137

Query  157  LQKRTEEADNLVRYINNR----SVKNRGNAFVVIDLRLAVRQYSGNVARKMMFGIRHFGK  212
               R  E  ++V  +       S  ++    VV+   LA      N   K+M G     K
Sbjct  138  RSLRETETRSMVESVYKDVMIDSTDDQTRKPVVVRKYLAAVVL--NTISKLMIG-----K  190

Query  213  GSEDGSGPGLEEIEHVESLFTVLTHLYAFALSDYVPWLRFLD--LEGHEKVVSNAMRNVS  270
                  G   +EI H E   +      + +L D + WL+++   L   +  +++  R
Sbjct  191  EFSTEEGKEFKEIVHKEHYLS-----GSGSLLDVIWWLKWVSKWLVSDKDFMAHMDRRTK  245

Query  271  KYNDPFVDERLMQWRNGKMKEPQDFLDMFIIAKDTDGKPTLSDEEIKAQVTELMLATVDN  330
             +    + E      +  +++ + F+   ++ K+   K  LS+E +   +  +  A  D
Sbjct  246  WFRGAIMVEE-----DVAVEDHEGFVRKLLVLKE---KKELSEETVHGLIWNMFTAGSDT  297

Query  331  PSNAAEWGMAEMINEPSIMQKAVEEIDRVVGKDRLVIESDLPNLNYVKACVKEAFRLHPV  390
             +   EW MAEMI  P++ +KA +E+D VVG +RL+ E+D+P L Y++  VKEA RLHP
Sbjct  298  TAVVLEWAMAEMIRCPNVQRKAQQELDAVVGSERLMTEADIPKLPYLQCVVKEALRLHPS  357

Query  391  APFNLPHMSTTDTVVDGYFIPKGSHVLISRMGIGRNPSVWDKPHKFDPERHLSTNTCVDL  450
             P  LPH ++    + GY +PKG+ V +    IGR+P  W  P +F PER L  +T  D+
Sbjct  358  TPTMLPHKASETVWLGGYKVPKGTTVYVHVKEIGRDPVNWINPKEFRPERFLLEDT--DV  415

Query  451  NESDLNIISFSAGRRGCMGVDIGSAMTYMLLARLIQGFTW-LPVPGKNKIDISESKNDL-  508
               D  ++ F +GRR C    +   +  +++  L+Q F+W  P+P +  ID+SE    L
Sbjct  416  KGRDFRVLPFGSGRRVCPAAQLSMNLMALVMGNLLQCFSWSSPIPPET-IDMSEIPGLLC  474

Query  509  FMAKPLYAVATPRLAPHVYP  528
             M  P+ A+A+PR A  V P
Sbjct  475  VMKTPVEALASPRAATRVIP  494


>XP_009141067.1 PREDICTED: trans-cinnamate 4-monooxygenase-like [Brassica rapa]
Length=505

 Score = 179 bits (454),  Expect = 6e-50, Method: Compositional matrix adjust.
 Identities = 123/434 (28%), Positives = 202/434 (47%), Gaps = 24/434 (6%)

Query  77   DIACIRLANTHVIPVTSPRIAREILKKQDSVFATRPLTMGTEYCSRGYLTVAVEPQGEQW  136
            D+   ++   +++ V+SP + +E+L  Q   F +R   M  +  +     +     GE W
Sbjct  67   DLFLFKMGQRNIVVVSSPDLTKEVLHTQGVEFGSRHRNMVYDIFTGKGQDMVFTVYGEHW  126

Query  137  KKMRRVVASHVTSKKSFQMMLQKRTEEADNLVRYIN-NRSVKNRGNAFVVIDLRLAVRQY  195
            +KMRR++     + K  Q   +    EA ++V  +  N     +G   +V+  RL +  Y
Sbjct  127  RKMRRIMTVPFFTNKVVQQNHEGWEFEAASVVEDVKKNPDAATKG---IVVRKRLQLMVY  183

Query  196  SGNVARKMMFGIRHFGKGSEDGSGPGLEEIEHVESLFTVLTHLYAFALSDYVPWLRFLDL  255
            +       MFGI  FGK  E    P    ++ +      LT  + +   D++P LR   L
Sbjct  184  NN------MFGI-MFGKRFESEDDPLFLRLKFLNGERGRLTQSFEYNYGDFIPILRPF-L  235
```

```
Query  256   EGHEKVVSNAM-RNVSKYNDPFVDERLM-----QWRNGKMKEPQDFLDMFIIAKDTDGKP  309
              G+ K+  +   R  + +   FVD+R          G +K   D +       +   K
Sbjct  236   RGYLKICQDVKDRRFALFKKYFVDDRKQVPSAKPTGGGGLKCAIDHI------LEAQKKG  289

Query  310   TLSDEEIKAQVTELMLATVDNPSNAAEWGMAEMINEPSIMQKAVEEIDRVVGKDRLVIES  369
              ++++ +   V  + +A ++    + EWG+AE++N P I  K   EID V+G    V E
Sbjct  290   EINEDNVLYIVENINVAAIETTLTSMEWGIAELVNHPEIQSKLRNEIDTVLGPGVHVTEP  349

Query  370   DLPNLNYVKACVKEAFRLHPVAPFNLPHMSTTDTVVDGYFIPKGSHVLISRMGIGRNPSV  429
              L  L Y++A +KE  R     P  +PHM+  D  + GY IP  S +LI+   +  NP
Sbjct  350   ALHKLPYLQAVIKETIRRRMAVPLLVPHMNLKDAKLAGYDIPAESRILINAWWLANNPDS  409

Query  430   WDKPHKFDPERHLSTNTCVDLNESDLNIISFSAGRRGCMGVDIGSAMTYMLLARLIQGFT  489
             W+ P +F PER L     V+ N +D   + F  GRR C G+ +   +  + + RL+Q F
Sbjct  410   WNSPEEFRPERFLEEEAHVEANGNDFRYVPFGVGRRSCPGIILALPILGITIGRLVQNFE  469

Query  490   WLPVPGKNKIDISE  503
              LP PG++KID SE
Sbjct  470   LLPPPGQSKIDTSE  483
```

>XP_009152073.1 PREDICTED: cytochrome P450 71B19-like [Brassica rapa]
Length=502

```
 Score = 179 bits (453),  Expect = 7e-50, Method: Compositional matrix adjust.
 Identities = 141/520 (27%), Positives = 237/520 (46%), Gaps = 48/520 (9%)

Query  7     MLAFIIGLLLLALTMKRKEKKKTMLISPTRNLSLPPGPKSWPLIGNLPEILGRNKPVFRW  66
             +L F +  L+  +  ++K K+           +LPP P  +P+IGNL +I G
Sbjct  5     LLCFCLITLVTLILFEKKIKQS--------KWNLPPSPPKFPVIGNLHQIGGLP------  50

Query  67    IHSLMKELNTDIACIRLANTHVIPV---TSPRIAREILKKQDSVFATRPLTMGTEYCSRG  123
              H  ++ L       + L +   +PV   +S   A E+L+  D    +RP  +GT   SR
Sbjct  51    -HRSLERLARKYGPVMLLHFGFVPVVVVSSREAAEEVLRTHDLDCCSRPKLVGTRLLSRD  109

Query  124   YLTVAVEPQGEQWKKMRRVVASHVTSKKSFQMMLQKRTEEADNLVRYINNRSVKNRGNAF  183
             +  +A  P GE+WK+ R++    +   K  Q     R EE + +V+ ++  +V
Sbjct  110   FKDIAFTPYGEEWKERRKLAVRELFCLKKVQSFRYIREEECNFMVKKLSESAVCRSP---  166

Query  184   VVIDLRLAVRQYSGNVARKMMFGIRHFGKGSEDGSGPGLEEIEHVESLFTVLTHLYAFAL  243
               +DL  A+   + ++  ++  G     +   +      E+IE  E +F   T L +F
Sbjct  167   --VDLSKALFWLTASILFRVALG-----QNFNESKFIDKEKIE--ELVFEAETALGSFTC  217

Query  244   SDYVP-----WLRFLD-LEGHEKVVSNAMRNVSKYNDPFVDERLMQWRNGKMKEPQDFLD  297
             SD  P     WL  +D L G  K +++    +       +D+ L     G+ K+ QD +D
Sbjct  218   SDSFPVAGLGWL--VDWLSGQHKRLNDVFFKLDDLFQRVIDDHL---STGRSKDHQDIVD  272

Query  298   MF---IIAKDTDGKPTLSDEEIKAQVTELMLATVDNPSNAAEWGMAEMINEPSIMQKAVE  354
                  I  +  +G   L+ + I+  +  + LA +D  +    W M E+   P++M+K
Sbjct  273   SMLDMIHKQGQNGSLNLTVDHIRGVLLNIFLAGIDTGAITMIWAMTELARNPNLMKKVQR  332

Query  355   EIDRVVGKDRLVI-ESDLPNLNYVKACVKEAFRLHPVAPFNLPHMSTTDTVVDGYFIPKG  413
             EI   +G ++  I E D+  + Y+K  +KE FRLH   P  LP  +     V GY IP
Sbjct  333   EIRDALGNNKKTITEEDVEKVPYLKMVIKETFRLHHAVPLLLPRETMVHIKVQGYNIPPK  392

Query  414   SHVLISRMGIGRNPSVWDKPHKFDPERHLSTNTCVDLNESDLNIISFSAGRRGCMGVDIG  473
             + +L++   IGR+P +W  P +F+PER +  N+ VD       ++ F +GRR C G+ +G
Sbjct  393   TQILVNAGAIGRDPKLWTNPEEFNPERFI--NSPVDYRGQYFELLPFGSGRRICPGMPMG  450

Query  474   SAMTYMLLARLIQGFTWLPVPGKNKIDI-SESKNDLFMAK  512
              A   + L  L+  F W    G    DI +E    L + K
Sbjct  451   MATVELGLLNLLYFFDWSLPDGMTHEDIDTEEAGTLTIVK  490
```

>XP_009142048.1 PREDICTED: 3,9-dihydroxypterocarpan 6A-monooxygenase [Brassica rapa]
Length=518

```
 Score = 179 bits (454),  Expect = 9e-50, Method: Compositional matrix adjust.
 Identities = 146/514 (28%), Positives = 242/514 (47%), Gaps = 34/514 (7%)

Query  10   FIIGLLLLALTMKRKEKKKTMLISPTRNLSLPPGPKSWPLIGNLPEILGRNKPVFRWIHS  69
            F+   +L A       KK+    S T    LP  P + PLIG+L  ++G+  PV     S
Sbjct  15   FVFAFMLNAFFKWFSSKKQ----SSTAATKLPQSPSALPLIGHL-HLIGKVLPV--SFQS  67

Query  70   LMKELNTDIACIRLANTHVIPVTSPRIAREILKKQDSVFATRPLTMGTEYCSRGYLTVAV  129
            L ++ +  +  IRL  +  + V+S  +AREI K Q+  F++RP     EY         +
Sbjct  68   LARK-HGPLMEIRLGASKCVVVSSSSVAREIFKDQELNFSSRPEFGSAEYFKYRGSRFVL  126

Query  130  EPQGEQWKKMRRVVASHVTSKKSFQMMLQKRTEEADNLVRYINNRSVKNRGNAFVVIDLR  189
               G+ W+ M+++  + + +    +     R EE   LV      SV       +  DL
Sbjct  127  AQYGDYWRFMKKLCMTKLLAVPQLEKFSDIREEEKLKLVE-----SVSKCCREGLPCDLS  181

Query  190  LAVRQYSGNVARKMMFGIRHFGKGSEDGSGPGLEEIEHVESLFTVLTHLYAFALSDYVPW  249
                 Y+ N+  +M    R  G  +E       EEI  +  + T L      ++ D +
Sbjct  182  SMFVSYTNNIICRMAMSTRCSGTDNE------AEEIRKL--VKTCLELAGKVSVGDVLGP  233

Query  250  LRFLDLEGHEKVVSNAMRNVSKYNDPFVDER-LMQWRNGKMKEPQDFLDMFI-IAKDTDG  307
            L+ LD  G+ K +   M    +  +  + ER  M  +   M+  +D LDM +   +D
Sbjct  234  LKVLDFSGNGKKLVAVMEKYDQLVEKIMKEREAMGMKKEGMR--KDILDMLLETYRDPTA  291

Query  308  KPTLSDEEIKAQVTELMLATVDNPSNAAEWGMAEMINEPSIMQKAVEEIDRVVGKDRLVI  367
            +  ++  ++K+ + ++ +A  D  + A +W M E+IN P    K  EEI+ VVG  RLV
Sbjct  292  ELKITRNDMKSFLLDVFMAGTDTSAAAMQWAMGELINNPQAFNKLREEIETVVGSKRLVK  351

Query  368  ESDLPNLNYVKACVKEAFRLHPVAPFNLPHMSTTDTVVDGYFIPKGSHVLISRMGIGRNP  427
            ESD+PNL Y++A ++E  RLHP AP  +   +  D  V+G  I   + VL++   I R+P
Sbjct  352  ESDVPNLPYLRAVLRETLRLHPSAPLIIRECA-ADCQVNGCLIKSKTRVLVNVYAIMRDP  410

Query  428  SVWDKPHKFDPERHLSTNT------CVDLNESDLNIISFSAGRRGCMGVDIGSAMTYMLL  481
             +W +  +F PER L ++        ++    +   + F +GRRGC G  +   + +  +
Sbjct  411  ELWKEAEQFIPERFLESSQEKIGEHQMEFKGQNFRYLPFGSGRRGCPGASLAMNVMHAGV  470

Query  482  ARLIQGFTWLPVPGKNKIDISE-SKNDLFMAKPL  514
              L+Q F W  V G+ K+D+S+ S     MA+PL
Sbjct  471  GSLVQRFDWKCVDGE-KVDLSQGSGFAAEMARPL  503


>XP_009152068.1 PREDICTED: cytochrome P450 71B3-like [Brassica rapa]
Length=519

 Score = 179 bits (453),  Expect = 1e-49, Method: Compositional matrix adjust.
 Identities = 135/516 (26%), Positives = 239/516 (46%), Gaps = 39/516 (8%)

Query  7    MLAFIIGLLLLALTMKRKEKKKTMLISPTRNLSLPPGPKSWPLIGNLPEILGRNKPVFRW  66
            ++ +    LL +L   +K       I  +R  +LPP P  +P+IGNL ++ G    + R
Sbjct  22   IILYFFSFLLFSLIFVKK-------IKESRQ-NLPPSPPKFPIIGNLHQLRGL---LHRC  70

Query  67   IHSLMKELNTDIACIRLANTHVIPVTSPRIAREILKKQDSVFATRPLTMGTEYCSRGYLT  126
            +H + K+ +  +  +RL    ++ ++S   A+E LK  D    TRP T  +   SR
Sbjct  71   LHDISKK-HGPVLLLRLGFVQMVVISSSEAAQEALKTHDLECCTRPKTNASWTFSRDGQN  129

Query  127  VAVEPQGEQWKKMRRVVASHVTSKKSFQMMLQKRTEEADNLVRYINNRSVKNRGNAFVVI  186
            +A  P GE W+++R++   +  S K  +     R EE D +V+ +  + +  R +    +
Sbjct  130  IAFAPYGEAWRELRKLSVLNFFSAKKVRSFRYIREEENDLMVKKL--KELAQRKSP---V  184

Query  187  DLRLAVRQYSGNVARKMMFGIRHFGKGSEDGSGPGLEEIEHVESLFTVLTHLYAFALSDY  246
            DL   +   +G++  +  FG R       D         E ++ L   +    + + SD
Sbjct  185  DLSQTLFCLAGSIIFRAAFGQRFEENKHVDK--------ERIKELMFEVQRTASLSSSDL  236

Query  247  VP----WLRFLDLEGHEKVVSNAMRNVSKYNDPFVDERLMQWRNGKMKEPQDFLDMFII-  301
            +P    W  +  L G  + V+     V    +  +D+ L    +   K+  D +D  +
Sbjct  237  LPAGLGWFMYY-LSGQHRRVNQVFVEVDTLLNHIIDDHLKNPEDKTNKDRPDIVDSILDI  295

Query  302  --AKDTDGKPTLSDEEIKAQVTELMLATVDNPSNAAEWGMAEMINEPSIMQKAVEEIDRV  359
               ++ D    L+ + +K  + ++ LA VD  +    W MAE++  P +M+KA +EI
Sbjct  296  MHKQEQDDSFKLNFDNLKGIMQDIYLAGVDTSAITMIWAMAELVRNPRVMKKAQDEIRTC  355
```

```
Query  360  VG---KDRLVIESDLPNLNYVKACVKEAFRLHPVAPFNLPHMSTTDTVVDGYFIPKGSHV  416
             G   K+R + E D+  L Y+K  +KE  RLHP AP  LP  + +   + GY IP  + +
Sbjct  356  FGIKQKER-IEEEDVDKLQYLKLVIKETLRLHPAAPLLLPRETMSQIKIQGYDIPPKTLL  414

Query  417  LISRMGIGRNPSVWDKPHKFDPERHLSTNTCVDLNESDLNIISFSAGRRGCMGVDIGSAM  476
            +++   IGR+P  W+ P +F PER +  +  VD     + ++ F +GRR C G+    A
Sbjct  415  VVNAWSIGRDPKHWEDPEEFIPERFI--DCSVDYKGRGVEMLRFGSGRRMCPGMASAIAT  472

Query  477  TYMLLARLIQGFTWLPVPGKNKIDISESKNDLFMAK  512
              + L  L+  F W     +  ID+ E+ +   + K
Sbjct  473  VELGLLNLLYYFDWRLPEEETDIDMEEAGDTTVIKK  508
```

>XP_009121600.1 PREDICTED: cytochrome P450 71B4-like [Brassica rapa]
Length=512

```
 Score = 178 bits (452),  Expect = 2e-49, Method: Compositional matrix adjust.
 Identities = 138/484 (29%), Positives = 228/484 (47%), Gaps = 45/484 (9%)

Query  39   SLPPGPKSWPLIGNLPEILGRNKPVFRWIHSLMKELNTDIACIRLANTHVIPV---TSPR  95
            +LPP P   P IGNL ++ G        +H  + +L+     + L +   +PV   +S
Sbjct  31   NLPPSPPKLPFIGNLHQLQG-------LLHRRLLDLSKKHGPVMLLHLGFVPVLVASSSE  83

Query  96   IAREILKKQDSVFATRPLTMGTEYCSRGYLTVAVEPQGEQWKKMRRVVASHVTSKKSFQM  155
             A E+LK  D    TRP  +G +  SR    +     GE+W+++R++      + K  Q
Sbjct  84   AAEEVLKTHDLECCTRPKALGMQTFSRNGKDIGFSSYGEEWRELRKLAVLEFFNAKKVQS  143

Query  156  MLQKRTEEADNLVRYINNRSVKNRGNAFVVIDLRLAVRQYSGNVARKMMFGIRHFGKGSE  215
                R EE D  ++ +   ++K+       +DL   +   + ++  +  FG  +F
Sbjct  144  FRYIREEENDLTIKKLTESALKHSP-----VDLSKTLFSLTASIVFRSAFGQNYF-----  193

Query  216  DGSGPGLEEIEHVESLFTVLTHLYAFALSDYVP-----WLRFLDLEGHEKVVSNAMRNVS  270
            +      E+IE  E +F  L ++ +F  SD+ P     W     L  H++     +RNV
Sbjct  194  ENKRISKEKIE--ELMFEALANM-SFKFSDFFPVGGIGWFIDFVLGEHKR-----LRNVF  245

Query  271  KYNDPFVDERLMQWRNG-KMKEPQDFLDMF---IIAKDTDGKPTLSDEEIKAQVTELMLA  326
               D FV +     ++G    +  D +D+    I  ++ D    L+ + +  +++L LA
Sbjct  246  LEVDSFVRKVADDHKHGVTTPDRPDIVDVMLDMIKKQEQDESFKLTTDHLHGVISDLFLA  305

Query  327  TVDNPSNAAEWGMAEMINEPSIMQKAVEEID---RVVGKDRLVIESDLPNLNYVKACVKE  383
             VD  S    W MAE+I  P +M+KA EEI    R+  ++RL  E DL   +Y+K  VKE
Sbjct  306  GVDTSSIIMIWAMAELIRNPRVMKKAQEEIRTSIRIKPEERLA-EEDLDKAHYLKLVVKE  364

Query  384  AFRLHPVAPFNLPHMSTTDTVVDGYFIPKGSHVLISRMGIGRNPSVWDKPHKFDPERHLS  443
              RLHP AP  LP  + +   + GY IP  + ++++   +G++P  W  P +F PER +
Sbjct  365  TLRLHPAAPLLLPRETMSPIKIQGYDIPPKTLLIVNAWALGQDPKHWMNPEEFIPERFM-  423

Query  444  TNTCVDLNESDLNIISFSAGRRGCMGVDIGSAMTYMLLARLIQGFTW-LPVPGKNKIDIS  502
             +  VD       ++ FS+GRR C G+  G A   + L  L+  F W LP   K  +D+
Sbjct  424  -DCPVDYKGHSFEMLPFSSGRRMCPGMAFGIATVELGLLNLLYFFDWKLPEESKG-MDME  481

Query  503  ESKN  506
            ES +
Sbjct  482  ESGD  485
```

>XP_009128027.1 PREDICTED: cytochrome P450 78A5-like [Brassica rapa]
 XP_009128028.1 PREDICTED: cytochrome P450 78A5-like [Brassica rapa]
Length=532

```
 Score = 179 bits (453),  Expect = 2e-49, Method: Compositional matrix adjust.
 Identities = 140/493 (28%), Positives = 237/493 (48%), Gaps = 37/493 (8%)

Query  42   PGPKSWPLIGNLPEILGRNKPVFRWIHSLMKELNTD-IACIRLANTHVIPVTSPRIAREI  100
            PGP   P+IG L   +  N    R + S+    N   +    + +T  +  + P  A+E+
Sbjct  65   PGPAGLPIIGLLLAFV-NNASTHRILASIANSCNAKALMAFSVGSTRFVITSEPETAKEL  123

Query  101  LKKQDSVFATRPLTMGTEYCSRGYLTVAVEPQGEQWKKMRRVVASHVTSKKSFQMMLQKR  160
```

```
            L    S FA RPL   + Y    + ++   P G+ W+++RR+ ++H+ S K      + R
Sbjct  124  LNS--SAFADRPLNE-SAYELLFHRSMGFAPFGDYWRELRRISSTHLFSPKRISGFAESR  180

Query  161  TEEADNLVRYINNRSVKNRGNAFVVIDLRLAVRQYSGNVARKMMFG-IRHFGKGSEDGSG  219
             +  +++V  IN+ ++ + G     +++R  +   S N     +FG   +F  G+
Sbjct  181  RKIGNSMVEDINS-AMASYGE----VEIRRILHFGSLNNVMSTVFGRTFYFNDGTN----  231

Query  220  PGLEEIEHVESLFTVLTHLYAFALSDYVPWLRFLDLEGHEKVVSNAMRNVSKYNDPFVDE  279
                E+EH  S    L  L  F   D+ P  R+LDL+G  +   + +  V+ +    +D+
Sbjct  232  ----ELEHFVSEGYEL--LGIFNWGDHFPGARWLDLQGVRRRCRSLVGKVNVFVGNIIDD  285

Query  280  RLMQWRNGKMKEPQ-----DFLDMFIIAKDTDGKPTLSDEEIKAQVTELMLATVDNPSNA  334
             + +      +E +     DF+D+ +      G   LS+ ++ A + E++    D  +
Sbjct  286  HISKRSLHDSQEEESTNEDDFVDVLL---GMQGNSKLSNSDMIAVLWEMIFRGTDTVAIL  342

Query  335  AEWGMAEMINEPSIMQKAVEEIDRVVGKD-RLVIESDLPNLNYVKACVKEAFRLHPVAPF  393
             EW +A MI  P I  KA  EID +VG+  R V +SDL  L Y++A VKE  R+HP  P
Sbjct  343  LEWILARMILHPDIQAKAQAEIDVIVGESGRQVSDSDLSKLPYLRAIVKETLRMHPPGPL  402

Query  394  -NLPHMSTTDTVVDGYFIPKGSHVLISRMGIGRNPSVWDKPHKFDPERHLST--NTCVDL  450
                 ++  DT +  +FIP G+  +++   I  +  VW + H++ P+R L    +    +
Sbjct  403  LAWARLTIHDTQIGTHFIPAGTTAMVNMWAITHDEKVWPEAHEYKPDRFLGAPESGNFPI  462

Query  451  NESDLNIISFSAGRRGCMGVDIGSAMTYMLLARLIQGFTWLPVPGKNKIDISES-KNDLF  509
              SDL +  F AGRR C G  +G A   + LA+L+  F W+P     ++D+SE+ K  L
Sbjct  463  MGSDLRLAPFGAGRRVCPGKSMGIATVELWLAQLLGRFKWVPC---GEVDLSETLKLSLE  519

Query  510  MAKPLYAVATPRL  522
            M  PL   A PR+
Sbjct  520  MKNPLVCKAIPRV  532


>XP_009151311.1 PREDICTED: cytochrome P450 71A23-like [Brassica rapa]
Length=485

 Score = 177 bits (450),  Expect = 2e-49, Method: Compositional matrix adjust.
 Identities = 132/487 (27%), Positives = 226/487 (46%), Gaps = 42/487 (9%)

Query  42   PGPKSWPLIGNLPEILGRNKPVFRWIHSLMKELNTDIACIRLANTHVIPV---TSPRIAR  98
            P P   PLIGN+ + LG+      + H  ++ L+       L +   +PV   +S   AR
Sbjct  31   PSPPGLPLIGNMHQ-LGQ------YPHQSLRSLSQHYGPFMLLHFGTVPVLVASSADAAR  83

Query  99   EILKKQDSVFATRPLTMGTEYCSRGYLTVAVEPQGEQWKKMRRVVASHVTSKKSFQMMLQ  158
            +ILK  D VFA+RP +   +    G   +A  P GE W++M+ +   H+ S K  +
Sbjct  84   DILKTHDRVFASRPHSKIYDKLLYGSRNLASAPYGEYWRQMKSLSVLHLLSNKMVRTFRD  143

Query  159  KRTEEADNLVRYINNRSVKNRGNAFVVIDLRLAVRQYSGNVARKMMFGIRHFGKGSEDGS  218
             R EE   ++      +++ +G++   ++L   +   + +V  ++  G ++       G+
Sbjct  144  VRQEEISLMME-----TIRKQGSS--PMNLSKIMMTCTSDVICRVALGRKY-------GA  189

Query  219  GPGLEEIEHVESLFTVLTHLYAFALSDYVPWLRFLDLEGHEKVVSNAMRNVSKYNDPF--  276
               L+E+        ++  L  F    +VP L ++D       +    R + K  + F
Sbjct  190  ETDLKELTD-----RLVRQLGTFTFGSFVPCLSWIDW------ICGLERQLEKTANDFDE  238

Query  277  VDERLMQWRNGKMKEPQDFLDMFI-IAKDTDGKPTLSDEEIKAQVTELMLATVDNPSNAA  335
            + E+++Q          DF D+ + + +D      +S   IKA + +  +   D  S
Sbjct  239  ILEKVVQDHEDGDGGKADFADVLLALQRDKSVGFEVSRMSIKAIILDAFVGGTDTSSTLL  298

Query  336  EWGMAEMINEPSIMQKAVEEIDRVVGKDRLVIESDLPNLNYVKACVKEAFRLHPVAPFNL  395
            EW M+E+++ P  +++  EE+  V      V E D+ ++ Y+KA +KE  RLHP  P  +
Sbjct  299  EWEMSELLSHPECLKRLQEEVRTVSKGKSSVSEDDIQDMYYLKAVIKETLRLHPPFPLTV  358

Query  396  PHMSTTDTVVDGYFIPKGSHVLISRMGIGRNPSVWD-KPHKFDPERHLSTNTCVDLNESD  454
            PH+ST D  + GY IP G+ V+I+   +GR  + W      F PERHL  N+ VD    D
Sbjct  359  PHVSTEDVNLRGYHIPAGTQVMINLYAVGREVATWGPDADDFKPERHL--NSPVDFLGQD  416

Query  455  LNIISFSAGRRGCMGVDIGSAMTYMLLARLIQGFTWLPVPGKNKIDISESKNDLFMAK-P  513
              +I F AGRR C G+   + +  + LA L+ G  W     + +  + ES   +     P
Sbjct  417  FELIPFGAGRRMCPGISFAAVLNEVALANLMLGIDWQSTEDQTENHVPESIGVVIRRMFP  476
```

```
Query  514   LYAVATP  520
             L   A+P
Sbjct  477   LIVTASP  483


>XP_009111243.1 PREDICTED: cytochrome P450 705A20-like [Brassica rapa]
Length=513

 Score = 177 bits (449),  Expect = 4e-49, Method: Compositional matrix adjust.
 Identities = 130/446 (29%), Positives = 209/446 (47%), Gaps = 26/446 (6%)

Query  81    IRLANTHVIPVTSPRIAREILKKQDSVFATRPLTMGTEYCSRGYLTVAVEPQGEQWKKMR  140
             IR+ N  +I V+S  +A EI K  D   +TR L    E    G   +   P G+ WK M+
Sbjct  79    IRIFNAPIILVSSASVAYEIFKSHDVNVSTRALAAIDESLVFGSYGIINAPYGDWKFMK  138

Query  141   RVVASHVTSKKSFQMMLQKRTEEADNLVRYINNRSVKNRGNAFVVIDLRLAVRQYSGNVA  200
             +++A+ +   +S +  L  R EE     R +  ++ KN G     +++     +   N
Sbjct  139   KLIATKLLRPQSLERSLGIRAEEIQRFYRSLLEKARKNEG-----VEISKEAMKLINNTL  193

Query  201   RKMMFGIRHFGKGSEDGSGPGLEEIEHVESLFTVLTHLYAFALSDYVPWLRFLDLEGHE-  259
              +M  G R F +  E+G      E E V  L   +   YA     ++  L    L+
Sbjct  194   CRMSMG-RSFSE--ENG------EAEKVRGL---VGESYALTKKMFLAALLRRPLKKLRI  241

Query  260   KVVSNAMRNVSKYNDPFVDERLMQWRNGKMKEPQ---DFLDMFIIA-KDTDGKPTLSDEE  315
              +    + +VS   D  + ER++     K+ E Q   D +D+ + A  D + +  ++  +
Sbjct  242   PLFKKEIMSVSDRLDELL-ERIIVEHTEKLDEKQQDKDMMDVLLAAYGDEEAEYKITMNQ  300

Query  316   IKAQVTELMLATVDNPSNAAEWGMAEMINEPSIMQKAVEEIDRVVGKDRLVIESDLPNLN  375
             IKA   EL +   D      +W MAE+++ P+++++  EEID  VG  RL+ E+DLPNL
Sbjct  301   IKAFFVELFVGATDTSVQTTQWTMAEILDNPNVLERLREEIDSAVGSSRLIQETDLPNLP  360

Query  376   YVKACVKEAFRLHPVAPFNLPHMSTTDTVVDGYFIPKGSHVLISRMGIGRNPSVWDKPHK  435
             Y++A VKE  RLHP  P  L         + GY+IP+ + ++I+   + R+P  W+ P +
Sbjct  361   YLQAVVKEGLRLHPPGPL-LVRTFQERCEIKGYYIPEKTTLVINAYAVMRDPDSWEDPDE  419

Query  436   FDPERHLSTNTCVDLNESDLNIISFSAGRRGCMGVDIGSAMTYMLLARLIQGFTWLPVPG  495
             F PER LS     D  E     + F +GRRGC G ++         +  ++Q F W    G
Sbjct  420   FKPERFLSPKE--DEKELAFKYLPFGSGRRGCPGGNLSQIFVGTAVGVMVQCFEWKIEGG  477

Query  496   KNKIDISESKNDLFMAKPLYAVATPR  521
             K  ++ S    +L M  PL  V   R
Sbjct  478   KVNMEESFEGMNLSMVHPLKCVPVAR  503


>XP_009142616.1 PREDICTED: cytochrome P450 78A6-like [Brassica rapa]
Length=528

 Score = 177 bits (450),  Expect = 4e-49, Method: Compositional matrix adjust.
 Identities = 141/480 (29%), Positives = 221/480 (46%), Gaps = 32/480 (7%)

Query  42    PGPKSWPLIGNLPEILGRNKPVFRWIHSLMKELNTDIACIRLANTHVIPVTSPRIAREIL  101
             PGP+ +P +G++  ++       R   +  +     +    L  T VI   +P +A+EIL
Sbjct  69    PGPRGFPFVGSM-SLMSSTLAHHRIAEAAERYGAKRLMAFSLGETRVIVTCNPDVAKEIL  127

Query  102   KKQDSVFATRPLTMGTEYCSRGYLTVAVEPQGEQWKKMRRVVASHVTSKKSFQMMLQKRT  161
                  +FA RP+   + Y       +   P G  W+ +RR+ ++H+ S K  +    +
Sbjct  128   NS--PIFADRPVK-ESAYSLMFNRAIGFAPHGVYWRTLRRIASNHLFSPKQIKGAETQSR  184

Query  162   EEADNLVRYINNRSVKNRGNAFVVIDLRLAVRQYSGNVARKMMFGIRHFGKGSE-DGSGP  220
               A  +V  +  +S  N   A   +   L     S      MM  +  FG+  E D
Sbjct  185   VIASQMVGLLEKQSSTN---ALCFVRELLKTASLS-----NMMCSV--FGQEYELDQDHV  234

Query  221   GLEEIEHVESLFTVLTHLYAFALSDYVPWLRFLDLEGHEKVVSNAMRNVSKYNDPFVDER  280
              L E+  VE  + +L  L     +D++PWL   D    +++ S     V K N  FV
Sbjct  235   ELREL--VEEGYDLLGEL---NWTDHLPWLSEFD---PQRIRSRCSALVPKVNR-FVSRI  285

Query  281   LMQWRNGKMKEPQDFLDMFIIAKDTDGKPTLSDEEIKAQVTELMLATVDNPSNAAEWGMA  340
             + + R      P+DF+D+ +      G   LSD +I A + E++    D  +   EW +A
```

```
Sbjct  286  ISEHRRQTGDSPRDFVDVLL---SLHGSDQLSDPDIIAVLWEMIFRGTDTVAVLVEWILA  342

Query  341  EMINEPSIMQKAVEEIDRVVGKDRLVIESDLPNLNYVKACVKEAFRLHPVAP-FNLPHMS  399
             M+  P I      E+D VVGK R V ESDL +L Y+ A VKE  R+HP  P  +   ++
Sbjct  343  RMVLHPDIQSTVQNELDSVVGKSRAVDESDLVSLPYLTAMVKEVLRMHPPGPLLSWARLA  402

Query  400  TTDTVVDGYFIPKGSHVLISRMGIGRNPSVWDKPHKFDPERHLSTNTCVDLN--ESDLNI  457
             TDT+VDG  +P G+  +++   I  +P VW  P +F PER ++    V+ +   SDL +
Sbjct  403  ITDTIVDGCLVPAGTTAMVNMWAIAHDPHVWVDPLEFKPERFVAKEGEVEFSVLGSDLRL  462

Query  458  ISFSAGRRGCMGVDIGSAMTYMLLARLIQGFTWLPVPGKNKIDISES-KNDLFMAKPLYA  516
              F +GRR C G ++G        A L+  F W    G N +D+SE  +    MA PL A
Sbjct  463  APFGSGRRICPGKNLGLTTVTFWTATLLHEFEWGSSVG-NGVDLSEKLRLSCEMATPLAA  521


  Database: NCBI Protein Reference Sequences
    Posted date:  Aug 30, 2016  10:23 PM
  Number of letters in database: 19,254,814
  Number of sequences in database:  46,093

Lambda      K        H
   0.320    0.136    0.406
Gapped
Lambda      K        H
   0.267   0.0410    0.140
Matrix: BLOSUM62
Gap Penalties: Existence: 11, Extension: 1
Number of Sequences: 46093
Number of Hits to DB: 1530
Number of extensions: 4
Number of successful extensions: 4
Number of sequences better than 100: 2
Number of HSP's better than 100 without gapping: 0
Number of HSP's gapped: 2
Number of HSP's successfully gapped: 2
Length of query: 529
Length of database: 19254814
Length adjustment: 107
Effective length of query: 422
Effective length of database: 14322863
Effective search space: 6044248186
Effective search space used: 6044248186
T: 21
A: 40
X1: 16 (7.4 bits)
X2: 38 (14.6 bits)
X3: 64 (24.7 bits)
S1: 41 (20.4 bits)
S2: 56 (26.2 bits)
ka-blk-alpha gapped: 1.9
ka-blk-alpha ungapped: 0.7916
ka-blk-alpha_v gapped: 42.6028
ka-blk-alpha_v ungapped: 4.96466
ka-blk-sigma gapped: 43.6362
```

c. Blast search of cinnamoyl-CoA reductase

BLASTP 2.5.0+
Reference: Stephen F. Altschul, Thomas L. Madden, Alejandro A. Schaffer, Jinghui Zhang, Zheng Zhang, Webb Miller, and David J. Lipman (1997), "Gapped BLAST and PSI-BLAST: a new generation of protein database search programs", Nucleic Acids Res. 25:3389-3402.

Reference for compositional score matrix adjustment: Stephen F. Altschul, John C. Wootton, E. Michael Gertz, Richa Agarwala, Aleksandr Morgulis, Alejandro A. Schaffer, and Yi-Kuo Yu (2005) "Protein database searches using compositionally adjusted substitution matrices", FEBS J. 272:5101-5109.

RID: X1HGNWT8014

Database: NCBI Protein Reference Sequences
67,628,579 sequences; 25,829,655,146 total letters
Query= ABR15768.1 phenylacetaldehyde reductase [Solanum lycopersicum]

Length=328

Sequences producing significant alignments:		Score (Bits)	E Value
XP_009120886.1	PREDICTED: cinnamoyl-CoA reductase 1 isoform X...	518	0.0
XP_009144901.1	PREDICTED: cinnamoyl-CoA reductase 1-like isof...	509	0.0
XP_009107209.1	PREDICTED: cinnamoyl-CoA reductase 1 [Brassica...	507	0.0
XP_009114927.1	PREDICTED: cinnamoyl-CoA reductase 1-like [Bra...	506	0.0
XP_009120887.1	PREDICTED: cinnamoyl-CoA reductase 1 isoform X...	456	3e-163
XP_009144902.1	PREDICTED: cinnamoyl-CoA reductase 2-like isof...	398	2e-140
XP_009118318.1	PREDICTED: cinnamoyl-CoA reductase 1-like [Bra...	358	5e-124
XP_009127680.1	PREDICTED: cinnamoyl-CoA reductase 1-like isof...	358	7e-124
XP_009148244.1	PREDICTED: cinnamoyl-CoA reductase 1-like [Bra...	349	2e-120
XP_009148245.1	PREDICTED: cinnamoyl-CoA reductase 1-like [Bra...	343	8e-118
XP_009148246.1	PREDICTED: cinnamoyl-CoA reductase 1-like isof...	342	2e-117
XP_009148247.1	PREDICTED: tetraketide alpha-pyrone reductase ...	316	8e-108
XP_009127681.1	PREDICTED: cinnamoyl-CoA reductase 1-like isof...	303	2e-102
XP_009120358.1	PREDICTED: cinnamoyl-CoA reductase 1 [Brassica...	300	7e-101
XP_009109293.1	PREDICTED: tetraketide alpha-pyrone reductase ...	291	3e-97
XP_009117788.1	PREDICTED: cinnamoyl-CoA reductase 1 [Brassica...	291	3e-97
XP_009102146.1	PREDICTED: tetraketide alpha-pyrone reductase ...	290	3e-97
XP_009149037.1	PREDICTED: cinnamoyl-CoA reductase 1 [Brassica...	290	7e-97
XP_009121757.1	PREDICTED: tetraketide alpha-pyrone reductase ...	262	5e-86
XP_009105396.1	PREDICTED: tetraketide alpha-pyrone reductase ...	261	8e-86
XP_009129149.1	PREDICTED: cinnamoyl-CoA reductase 1 [Brassica...	240	2e-77
XP_009141331.1	PREDICTED: cinnamoyl-CoA reductase 1 [Brassica...	227	1e-72
XP_009115319.1	PREDICTED: tetraketide alpha-pyrone reductase ...	226	1e-71
XP_009115320.1	PREDICTED: tetraketide alpha-pyrone reductase ...	208	3e-65
XP_009106316.1	PREDICTED: cinnamoyl-CoA reductase 2 [Brassica...	199	1e-61
XP_009137647.1	PREDICTED: dihydroflavonol-4-reductase-like is...	197	2e-60
XP_009137646.1	PREDICTED: dihydroflavonol-4-reductase-like is...	196	8e-60
XP_009105139.1	PREDICTED: anthocyanidin reductase [Brassica r...	184	2e-55
XP_009113238.1	PREDICTED: anthocyanidin reductase-like [Brass...	183	4e-55
XP_009115321.1	PREDICTED: tetraketide alpha-pyrone reductase ...	180	2e-54
XP_009144935.1	PREDICTED: LOW QUALITY PROTEIN: bifunctional d...	169	5e-50
XP_009142332.1	PREDICTED: vestitone reductase-like [Brassica ...	162	4e-47
XP_009137924.1	PREDICTED: cinnamoyl-CoA reductase 2 [Brassica...	153	5e-44
XP_009140504.1	PREDICTED: cinnamoyl-CoA reductase 2 [Brassica...	150	9e-43
XP_009143137.1	PREDICTED: vestitone reductase [Brassica rapa]	149	7e-42
XP_009125995.1	PREDICTED: cinnamoyl-CoA reductase 2-like isof...	123	4e-32
XP_009121639.1	PREDICTED: cinnamoyl-CoA reductase 2 [Brassica...	120	7e-31
XP_009137735.1	PREDICTED: tetraketide alpha-pyrone reductase ...	110	1e-28

```
XP_009125996.1  PREDICTED: cinnamoyl-CoA reductase 2-like isof...  112     3e-28
XP_009141386.1  PREDICTED: uncharacterized protein At2g34460, ...  45.8    2e-05
XP_009141385.1  PREDICTED: uncharacterized protein At2g34460, ...  45.8    2e-05
XP_009108619.1  PREDICTED: uncharacterized protein ycf39 [Bras...  36.6    0.023
XP_009137405.1  PREDICTED: UDP-glucose 4-epimerase 2 [Brassica...  35.4    0.066
XP_009137528.1  PREDICTED: UDP-glucose 4-epimerase 2 [Brassica...  30.4    2.0
XP_009150492.1  PREDICTED: protein TIC 62, chloroplastic [Bras...  29.3    6.4

ALIGNMENTS
>XP_009120886.1 PREDICTED: cinnamoyl-CoA reductase 1 isoform X1 [Brassica rapa]
Length=330

 Score = 518 bits (1334),  Expect = 0.0, Method: Compositional matrix adjust.
 Identities = 241/324 (74%), Positives = 282/324 (87%), Gaps = 0/324 (0%)

Query  5    AKTVCVTGASGYIASWLVKFLLHSGYNVKASVRDPNDPKKTQHLLSLGGAKERLHLFKAN  64
             K VCVTGASGYIASWLVKFLL  GY VKASVRDP+DPKKTQHL+SL GAKERLHLFKA+
Sbjct  7    GKVVCVTGASGYIASWLVKFLLSRGYTVKASVRDPSDPKKTQHLVSLDGAKERLHLFKAD  66

Query  65   LLEEGSFDAVVDGCEGVFHTASPFYYSVTDPQAELLDPAVKGTLNLLGSCAKAPSVKRVV  124
            LLEEGSFD+ +DGCEGVFHTASPFY+ V DPQAEL+DPAVKGTLN+L SC KA SVKRVV
Sbjct  67   LLEEGSFDSAIDGCEGVFHTASPFYHDVKDPQAELIDPAVKGTLNVLNSCTKASSVKRVV  126

Query  125  LTSSIAAVAYSGQPRTPEVVVDESWWTSPDYCKEKQLWYVLSKTLAEDAAWKFVKEKGID  184
            +TSS+AAVAY+G+PRTP+V VDE+W++ P+ CK  ++WYVLSKTLAEDAAWKF KEK +D
Sbjct  127  VTSSMAAVAYNGKPRTPDVTVDETWFSDPEVCKTSKMWYVLSKTLAEDAAWKFAKEKDLD  186

Query  185  MVVVNPAMVIGPLLQPTLNTSSAAVLSLVNGAETYPNSSFGWVNVKDVANAHILAFENPS  244
            +V +NPAMVIGPLLQPTLNTS+AA+L+ +NGA+T+ NS+FGWVNVKDVANAHI A+E PS
Sbjct  187  IVTINPAMVIGPLLQPTLNTSAAAILNFINGAKTFSNSTFGWVNVKDVANAHIQAYEVPS  246

Query  245  ANGRYLMVERVAHYSDILKILRDLYPTMQLPEKCADDNPLMQNYQVSKEKAKSLGIEFTT  304
            ANGRY MVERV H+S+I+ ILR LYP +QLPE CAD+NP +  YQVSKEK KS+GI++
Sbjct  247  ANGRYCMVERVLHHSEIVNILRQLYPNLQLPESCADENPFVPTYQVSKEKIKSIGIDYIP  306

Query  305  LEESIKETVESLKEKKFFGGSSSM  328
            LE SIKETVESLKEK F    +S+
Sbjct  307  LEVSIKETVESLKEKGFINVRASI  330


>XP_009144901.1 PREDICTED: cinnamoyl-CoA reductase 1-like isoform X1 [Brassica
rapa]
Length=325

 Score = 509 bits (1310),  Expect = 0.0, Method: Compositional matrix adjust.
 Identities = 235/317 (74%), Positives = 276/317 (87%), Gaps = 0/317 (0%)

Query  6    KTVCVTGASGYIASWLVKFLLHSGYNVKASVRDPNDPKKTQHLLSLGGAKERLHLFKANL  65
            KTVCVTGASGYIASW+VK LL  GY VKASVRDPNDP+KT+HLL+L GAKERL LFKANL
Sbjct  7    KTVCVTGASGYIASWIVKLLLLRGYTVKASVRDPNDPRKTEHLLALEGAKERLQLFKANL  66

Query  66   LEEGSFDAVVDGCEGVFHTASPFYYSVTDPQAELLDPAVKGTLNLLGSCAKAPSVKRVVL  125
            LEEGSFD+ +DGC+GVFHTASPFY+ V DPQAELLDPAVKGT+N+L +C K PSVKRVVL
Sbjct  67   LEEGSFDSAIDGCQGVFHTASPFYHDVKDPQAELLDPAVKGTINVLSTCLKTPSVKRVVL  126

Query  126  TSSIAAVAYSGQPRTPEVVVDESWWTSPDYCKEKQLWYVLSKTLAEDAAWKFVKEKGIDM  185
            TSSIA+VA++G PRTPE +VDESW+  P+YC+  +LWYVLSKTLAE+AAWKF KE  + +
Sbjct  127  TSSIASVAFNGMPRTPETIVDESWFADPEYCRAAKLWYVLSKTLAENAAWKFAKENDLQL  186

Query  186  VVVNPAMVIGPLLQPTLNTSSAAVLSLVNGAETYPNSSFGWVNVKDVANAHILAFENPSA  245
            V +N AMVIGPLLQPTLNTS+AAVLSL+ GA+T+PN++FGWVNVKDVANAHI AFENP+A
Sbjct  187  VSINAAMVIGPLLQPTLNTSAAAVLSLIKGAQTFPNATFGWVNVKDVANAHIQAFENPTA  246

Query  246  NGRYLMVERVAHYSDILKILRDLYPTMQLPEKCADDNPLMQNYQVSKEKAKSLGIEFTTL  305
            NGRY +VERVAHYS+++ IL DLYP  QLPEKCAD+   +  Y+VSKEKA+SLG+EF  L
Sbjct  247  NGRYCLVERVAHYSEVVNILHDLYPDFQLPEKCADEKIYIPTYKVSKEKAESLGVEFVPL  306

Query  306  EESIKETVESLKEKKFF  322
            E SIKETVESL++K F
```

```
Sbjct  307  EVSIKETVESLQDKGFI  323
```

>XP_009107209.1 PREDICTED: cinnamoyl-CoA reductase 1 [Brassica rapa]
Length=325

Score = 507 bits (1306), Expect = 0.0, Method: Compositional matrix adjust.
Identities = 234/317 (74%), Positives = 275/317 (87%), Gaps = 0/317 (0%)

```
Query  6    KTVCVTGASGYIASWLVKFLLHSGYNVKASVRDPNDPKKTQHLLSLGGAKERLHLFKANL  65
            KTVCVTGASGYIASW+VK LL  GY VKASVRDPNDP+KT+HLL+L GA+ERL LFKANL
Sbjct  7    KTVCVTGASGYIASWIVKLLLLRGYTVKASVRDPNDPRKTEHLLALEGAEERLQLFKANL  66

Query  66   LEEGSFDAVVDGCEGVFHTASPFYYSVTDPQAELLDPAVKGTLNLLGSCAKAPSVKRVVL  125
            LEEGSFD+ +DGC+GVFHTASPFY+ V DPQAELLDPAVKGT+N+L +C K PSVKRVVL
Sbjct  67   LEEGSFDSAIDGCQGVFHTASPFYHDVKDPQAELLDPAVKGTINVLSTCLKTPSVKRVVL  126

Query  126  TSSIAAVAYSGQPRTPEVVVDESWWTSPDYCKEKQLWYVLSKTLAEDAAWKFVKEKGIDM  185
            TSSIAAVA++G PRTP+ +VDE+W+  PDYC+  +LWYVLSKTLAE+AAWKF KE  + +
Sbjct  127  TSSIAAVAFNGMPRTPDTIVDETWFADPDYCRAAKLWYVLSKTLAENAAWKFAKENDMQL  186

Query  186  VVVNPAMVIGPLLQPTLNTSSAAVLSLVNGAETYPNSSFGWVNVKDVANAHILAFENPSA  245
            V +N AMVIGPLLQPTLNTS+AAVLSL+ GA+T+PN++FGWVNVKDVANAHI AFENP A
Sbjct  187  VSINAAMVIGPLLQPTLNTSAAAVLSLIKGAQTFPNATFGWVNVKDVANAHIQAFENPEA  246

Query  246  NGRYLMVERVAHYSDILKILRDLYPTMQLPEKCADDNPLMQNYQVSKEKAKSLGIEFTTL  305
            NGRY +VERVAHYS+++ IL DLYP  QLPEKCAD+   +  Y+VSKEKA+SLG+EF  L
Sbjct  247  NGRYCLVERVAHYSEVVNILHDLYPDFQLPEKCADEKIFIPTYKVSKEKAESLGVEFVPL  306

Query  306  EESIKETVESLKEKKFF  322
            E SIKETVESL++K F
Sbjct  307  EVSIKETVESLQDKGFI  323
```

>XP_009114927.1 PREDICTED: cinnamoyl-CoA reductase 1-like [Brassica rapa]
Length=325

Score = 506 bits (1304), Expect = 0.0, Method: Compositional matrix adjust.
Identities = 234/317 (74%), Positives = 275/317 (87%), Gaps = 0/317 (0%)

```
Query  6    KTVCVTGASGYIASWLVKFLLHSGYNVKASVRDPNDPKKTQHLLSLGGAKERLHLFKANL  65
            KTVCVTGASGYIASW+VK LL  GY VKASVRDPNDP+KT+HLL+L GA+ERL LFKANL
Sbjct  7    KTVCVTGASGYIASWIVKLLLLRGYTVKASVRDPNDPRKTEHLLALEGAEERLQLFKANL  66

Query  66   LEEGSFDAVVDGCEGVFHTASPFYYSVTDPQAELLDPAVKGTLNLLGSCAKAPSVKRVVL  125
            LEEGSFD+ +DGC+GVFHTASPFY+ V DPQAELLDPAVKGT+N+L +C K PSVKRVVL
Sbjct  67   LEEGSFDSAIDGCQGVFHTASPFYHDVKDPQAELLDPAVKGTINVLSTCLKTPSVKRVVL  126

Query  126  TSSIAAVAYSGQPRTPEVVVDESWWTSPDYCKEKQLWYVLSKTLAEDAAWKFVKEKGIDM  185
            TSSIAAVA++G PRTP+ +VDESW+  P+YC+  +LWYVLSKTLAE+AAWKF KE  + +
Sbjct  127  TSSIAAVAFNGMPRTPDTIVDESWFADPEYCRAAKLWYVLSKTLAENAAWKFAKENDMQL  186

Query  186  VVVNPAMVIGPLLQPTLNTSSAAVLSLVNGAETYPNSSFGWVNVKDVANAHILAFENPSA  245
            V +N AMVIGPLLQPTLNTS+AAVLSL+ GA+T+PN++FGWVNVKDVANAHI AFENP A
Sbjct  187  VSINAAMVIGPLLQPTLNTSAAAVLSLIKGAQTFPNATFGWVNVKDVANAHIQAFENPDA  246

Query  246  NGRYLMVERVAHYSDILKILRDLYPTMQLPEKCADDNPLMQNYQVSKEKAKSLGIEFTTL  305
            NGRY +VERVAHYS+++ IL DLYP  QLPEKCAD+   +  Y+VSKEKA+SLG+EF  L
Sbjct  247  NGRYCLVERVAHYSEVVNILHDLYPDFQLPEKCADEKIFIPTYKVSKEKAESLGVEFVPL  306

Query  306  EESIKETVESLKEKKFF  322
            E SIKETVESL++K F
Sbjct  307  EVSIKETVESLQDKGFI  323
```

>XP_009120887.1 PREDICTED: cinnamoyl-CoA reductase 1 isoform X2 [Brassica rapa]
Length=279

Score = 456 bits (1174), Expect = 3e-163, Method: Compositional matrix adjust.

```
 Identities = 209/273 (77%), Positives = 244/273 (89%), Gaps = 0/273 (0%)

Query  5    AKTVCVTGASGYIASWLVKFLLHSGYNVKASVRDPNDPKKTQHLLSLGGAKERLHLFKAN  64
             K VCVTGASGYIASWLVKFLL  GY VKASVRDP+DPKKTQHL+SL GAKERLHLFKA+
Sbjct  7    GKVVCVTGASGYIASWLVKFLLSRGYTVKASVRDPSDPKKTQHLVSLDGAKERLHLFKAD  66

Query  65   LLEEGSFDAVVDGCEGVFHTASPFYYSVTDPQAELLDPAVKGTLNLLGSCAKAPSVKRVV  124
            LLEEGSFD+ +DGCEGVFHTASPFY+ V DPQAEL+DPAVKGTLN+L SC KA SVKRVV
Sbjct  67   LLEEGSFDSAIDGCEGVFHTASPFYHDVKDPQAELIDPAVKGTLNVLNSCTKASSVKRVV  126

Query  125  LTSSIAAVAYSGQPRTPEVVVDESWWTSPDYCKEKQLWYVLSKTLAEDAAWKFVKEKGID  184
            +TSS+AAVAY+G+PRTP+V VDE+W++ P+ CK  ++WYVLSKTLAEDAAWKF KEK +D
Sbjct  127  VTSSMAAVAYNGKPRTPDVTVDETWFSDPEVCKTSKMWYVLSKTLAEDAAWKFAKEKDLD  186

Query  185  MVVVNPAMVIGPLLQPTLNTSSAAVLSLVNGAETYPNSSFGWVNVKDVANAHILAFENPS  244
            +V +NPAMVIGPLLQPTLNTS+AA+L+ +NGA+T+ NS+FGWVNVKDVANAHI A+E PS
Sbjct  187  IVTINPAMVIGPLLQPTLNTSAAAILNFINGAKTFSNSTFGWVNVKDVANAHIQAYEVPS  246

Query  245  ANGRYLMVERVAHYSDILKILRDLYPTMQLPEK  277
            ANGRY MVERV H+S+I+ ILR LYP +QLPE+
Sbjct  247  ANGRYCMVERVLHHSEIVNILRQLYPNLQLPER  279


>XP_009144902.1 PREDICTED: cinnamoyl-CoA reductase 2-like isoform X2 [Brassica
rapa]
Length=270

 Score = 398 bits (1023),  Expect = 2e-140, Method: Compositional matrix adjust.
 Identities = 199/317 (63%), Positives = 232/317 (73%), Gaps = 55/317 (17%)

Query  6    KTVCVTGASGYIASWLVKFLLHSGYNVKASVRDPNDPKKTQHLLSLGGAKERLHLFKANL  65
            KTVCVTGASGYIASW+VK LL  GY VKASVRDPNDP+KT+HLL+L GAKERL LFKANL
Sbjct  7    KTVCVTGASGYIASWIVKLLLLRGYTVKASVRDPNDPRKTEHLLALEGAKERLQLFKANL  66

Query  66   LEEGSFDAVVDGCEGVFHTASPFYYSVTDPQAELLDPAVKGTLNLLGSCAKAPSVKRVVL  125
            LEEGSFD+ +DGC+GVFHTASPFY+ V DPQAELLDPAVKGT+N+L +C K PSVKRVVL
Sbjct  67   LEEGSFDSAIDGCQGVFHTASPFYHDVKDPQAELLDPAVKGTINVLSTCLKTPSVKRVVL  126

Query  126  TSSIAAVAYSGQPRTPEVVVDESWWTSPDYCKEKQLWYVLSKTLAEDAAWKFVKEKGIDM  185
            TSSIA+VA++                                                M
Sbjct  127  TSSIASVAFNA------------------------------------------------M  138

Query  186  VVVNPAMVIGPLLQPTLNTSSAAVLSLVNGAETYPNSSFGWVNVKDVANAHILAFENPSA  245
            V+       GPLLQPTLNTS+AAVLSL+ GA+T+PN++FGWVNVKDVANAHI AFENP+A
Sbjct  139  VI-------GPLLQPTLNTSAAAVLSLIKGAQTFPNATFGWVNVKDVANAHIQAFENPTA  191

Query  246  NGRYLMVERVAHYSDILKILRDLYPTMQLPEKCADDNPLMQNYQVSKEKAKSLGIEFTTL  305
            NGRY +VERVAHYS+++ IL DLYP  QLPEKCAD+   +  Y+VSKEKA+SLG+EF  L
Sbjct  192  NGRYCLVERVAHYSEVVNILHDLYPDFQLPEKCADEKIYIPTYKVSKEKAESLGVEFVPL  251

Query  306  EESIKETVESLKEKKFF  322
            E SIKETVESL++K F
Sbjct  252  EVSIKETVESLQDKGFI  268


>XP_009118318.1 PREDICTED: cinnamoyl-CoA reductase 1-like [Brassica rapa]
Length=322

 Score = 358 bits (920),  Expect = 5e-124, Method: Compositional matrix adjust.
 Identities = 174/321 (54%), Positives = 228/321 (71%), Gaps = 0/321 (0%)

Query  1    MSVTAKTVCVTGASGYIASWLVKFLLHSGYNVKASVRDPNDPKKTQHLLSLGGAKERLHL  60
            M+   K VCVTGASGYIASW+VK LL  GY VKA+VRDP D KKT HLL+L GA+ERL L
Sbjct  1    MNGGGKVVCVTGASGYIASWIVKLLLLRGYTVKATVRDPKDQKKTDHLLALDGARERLQL  60

Query  61   FKANLLEEGSFDAVVDGCEGVFHTASPFYYSVTDPQAELLDPAVKGTLNLLGSCAKAPSV  120
            FKA+LLEEGSF+  +DGC+ VFHTASP     TDPQAEL++PAVKGT+N+L +C K  SV
Sbjct  61   FKASLLEEGSFEHAIDGCDAVFHTASPVKIIATDPQAELIEPAVKGTINVLTTCTKVSSV  120
```

```
Query  121  KRVVLTSSIAAVAYSGQPRTPEVVVDESWWTSPDYCKEKQLWYVLSKTLAEDAAWKFVKE  180
            KRV+LTSS+A +     P  P V++DE+ ++ P  C+E++ WY+LSKTLAE+AAW F K+
Sbjct  121  KRVILTSSMATLLSPNFPLGPNVLLDETTFSDPSVCEEEKQWYILSKTLAENAAWTFAKD  180

Query  181  KGIDMVVVNPAMVIGPLLQPTLNTSSAAVLSLVNGAETYPNSSFGWVNVKDVANAHILAF  240
              +D+VV+NP +VIGP+LQPT+N S   V+  + G  T+       V+V+DVA AHI A
Sbjct  181  NNLDLVVMNPGLVIGPVLQPTINFSVDVVIDFIKGKNTFNRKHHRLVDVRDVALAHIKAL  240

Query  241  ENPSANGRYLMVERVAHYSDILKILRDLYPTMQLPEKCADDNPLMQNYQVSKEKAKSLGI  300
            E PSANGRY++   +    +I KILR+ +P + +  +  D +     Y+V+ EK KSLGI
Sbjct  241  ETPSANGRYIIDAPIVTTEEIEKILREFFPDLCIAHENEDIDLNSMAYEVNVEKVKSLGI  300

Query  301  EFTTLEESIKETVESLKEKKF  321
            EFT  E S+++TV SLKEK
Sbjct  301  EFTPTETSLRDTVLSLKEKHL  321


>XP_009127680.1 PREDICTED: cinnamoyl-CoA reductase 1-like isoform X1 [Brassica
rapa]
Length=325

 Score = 358 bits (919),  Expect = 7e-124, Method: Compositional matrix adjust.
 Identities = 181/322 (56%), Positives = 231/322 (72%), Gaps = 3/322 (1%)

Query  1    MSVTAKTVCVTGASGYIASWLVKFLLHSGYNVKASVRDPNDPKKTQHLLSLGGAKERLHL  60
            M+   K VCVTGASGYIASW+VK LL  GY V+A+VRDPN+ KKT HLL L GAKERL L
Sbjct  1    MNGEGKVVCVTGASGYIASWIVKLLLQRGYTVRATVRDPNNQKKTDHLLQLDGAKERLSL  60

Query  61   FKANLLEEGSFDAVVDGCEGVFHTASPFYYSVTDPQAELLDPAVKGTLNLLGSCAKAPSV  120
            F ANLLEEGSF   +DGCE VFHTASP   +  DPQAEL++PAVKGTLN+L +C K  SV
Sbjct  61   FGANLLEEGSFQHAIDGCEAVFHTASPVLLTAEDPQAELIEPAVKGTLNVLKTCVKMSSV  120

Query  121  KRVVLTSSIAAVAYSGQPRTPEVVVDESWWTSPDYCKEKQLWYVLSKTLAEDAAWKFVKE  180
            KRV+LTSS+AAV     P  P  VVDE+ ++ P +C++++ WY LSKTLAED A KF K
Sbjct  121  KRVILTSSMAAVIAHASPTGPNGVVDETMFSDPSFCEQRKQWYALSKTLAEDEACKFAKA  180

Query  181  KGIDMVVVNPAMVIGPLLQPTLNTSSAAVLSLVNGAETYPNSSFGWVNVKDVANAHILAF  240
              +D++V+NP +VIGP+LQPTLN S   V+ L  G + + + S+ +V+V+DV+ AHI A
Sbjct  181  NEMDLIVMNPGLVIGPILQPTLNFSVGVVVELTKGKDPFMSKSYRFVDVRDVSLAHIKAL  240

Query  241  ENPSANGRYLMVERV-AHYSDILKILRDLYPTMQL-PEKCADDNPL-MQNYQVSKEKAKS  297
            E PSANGRY++   V A   DI K+LR+  P + +  +K  +D  L +  Y+VS EK +S
Sbjct  241  ETPSANGRYIIDGPVIATLKDIEKVLREFVPDLCIGDDKNNEDIDLDLVTYKVSVEKVRS  300

Query  298  LGIEFTTLEESIKETVESLKEK  319
            LGIEFT  E S+++TV SLKEK
Sbjct  301  LGIEFTPTETSLRDTVLSLKEK  322


>XP_009148244.1 PREDICTED: cinnamoyl-CoA reductase 1-like [Brassica rapa]
Length=322

 Score = 349 bits (896),  Expect = 2e-120, Method: Compositional matrix adjust.
 Identities = 168/319 (53%), Positives = 228/319 (71%), Gaps = 0/319 (0%)

Query  1    MSVTAKTVCVTGASGYIASWLVKFLLHSGYNVKASVRDPNDPKKTQHLLSLGGAKERLHL  60
            M+V  K VCVTGASGYIASW+VK LL  GY V+A+VR+P D  KT+H+L+L GAKERL L
Sbjct  1    MNVGGKVVCVTGASGYIASWIVKLLLLRGYTVRATVRNPTDAAKTEHILALEGAKERLKL  60

Query  61   FKANLLEEGSFDAVVDGCEGVFHTASPFYYSVTDPQAELLDPAVKGTLNLLGSCAKAPSV  120
            FKA+LLEE SF+  ++ C+ VFHTASP  + VT+PQ EL+DPA+KGT+N+L +C K  SV
Sbjct  61   FKADLLEECSFEQAIECCDAVFHTASPVKFIVTNPQTELIDPALKGTMNVLNTCKKTSSV  120

Query  121  KRVVLTSSIAAVAYSGQPRTPEVVVDESWWTSPDYCKEKQLWYVLSKTLAEDAAWKFVKE  180
            KRV++TSS+AAV     P  P  VVDES+++ P  C E + WY LSKTLAE+ AW+F KE
Sbjct  121  KRVIVTSSMAAVIVRQPPLEPNDVVDESFFSDPSVCMETEYWYPLSKTLAENVAWQFSKE  180

Query  181  KGIDMVVVNPAMVIGPLLQPTLNTSSAAVLSLVNGAETYPNSSFGWVNVKDVANAHILAF  240
             G+DMV +NP  +IGPLLQPTLN S   ++ ++NG   + +  + +V+V+DVA AH+ A
```

```
Sbjct  181  NGMDMVFINPGFIIGPLLQPTLNFSVEMIVDMINGKNPFNSIYYRFVDVRDVALAHVKAL  240

Query  241  ENPSANGRYLMVERVAHYSDILKILRDLYPTMQLPEKCADDNPLMQNYQVSKEKAKSLGI  300
            E PSANGRY+M        +I +I+R+L+P M + +   +       Y+V  EK K+LG+
Sbjct  241  ETPSANGRYIMDGASMTIYEIKEIMRELFPDMCVADTKEEGEIKEIIYKVCVEKVKNLGV  300

Query  301  EFTTLEESIKETVESLKEK  319
            EFT L+ S+++T+ SLKEK
Sbjct  301  EFTPLKSSLRDTIISLKEK  319


>XP_009148245.1 PREDICTED: cinnamoyl-CoA reductase 1-like [Brassica rapa]
Length=322

 Score = 343 bits (879),  Expect = 8e-118, Method: Compositional matrix adjust.
 Identities = 170/324 (52%), Positives = 228/324 (70%), Gaps = 10/324 (3%)

Query  1    MSVTAKTVCVTGASGYIASWLVKFLLHSGYNVKASVRDPNDPKKTQHLLSLGGAKERLHL  60
            M+   K VCVTGASGYIASW+VK LL  GY V+A+V++P D  +T+HLL+L GAKERL L
Sbjct  1    MNGGGKVVCVTGASGYIASWIVKLLLLRGYTVRATVQNPTDTAETEHLLALEGAKERLKL  60

Query  61   FKANLLEEGSFDAVVDGCEGVFHTASPFYYSVTDPQAELLDPAVKGTLNLLGSCAKAPSV  120
            FKA+LLE+ SF+  ++GC+ VFHTASP  + VTDPQ EL+DPAVKGTLN+L +C K  SV
Sbjct  61   FKADLLEDCSFEKAIEGCDAVFHTASPVKFIVTDPQTELIDPAVKGTLNVLNTCKKTSSV  120

Query  121  KRVVLTSSIAAVAYSGQPRTPEVVVDESWWTSPDYCKEKQLWYVLSKTLAEDAAWKFVKE  180
            KRV++TSS AAV     P  P  VVDE++++ P  C E++LWY LSKTLAE+ AW+F K+
Sbjct  121  KRVIVTSSTAAVLVRQPPLEPNDVVDETFFSDPSVCMERKLWYPLSKTLAENVAWQFAKD  180

Query  181  KGIDMVVVNPAMVIGPLLQPTLNTSSAAVLSLVNGAETYPNSSFGWVNVKDVANAHILAF  240
             G+DMVVVNP  +IGPLLQPTLN S   ++ +V G   +    + +V+V+DVA AH+ A
Sbjct  181  NGMDMVVVNPGFIIGPLLQPTLNFSVEIIVDMVKGKNPFNCRYYSFVDVRDVALAHVKAL  240

Query  241  ENPSANGRYLMVERVAHYSDILKILRDLYPTMQLPEKCADDNP---LMQ--NYQVSKEKA  295
            E PSANGRY++       + I + +R+L+P +     C DD     LM   N  +  +K
Sbjct  241  ETPSANGRYIISGPSVTINHIKETMRELFPKL-----CIDDTNGEGLMDGVNCTICVDKV  295

Query  296  KSLGIEFTTLEESIKETVESLKEK  319
            K+LG+EFT L+ S+++T+ SLKEK
Sbjct  296  KNLGVEFTPLKSSLRDTIISLKEK  319


>XP_009148246.1 PREDICTED: cinnamoyl-CoA reductase 1-like isoform X1 [Brassica
rapa]
Length=326

 Score = 342 bits (877),  Expect = 2e-117, Method: Compositional matrix adjust.
 Identities = 173/325 (53%), Positives = 230/325 (71%), Gaps = 8/325 (2%)

Query  1    MSVTAKTVCVTGASGYIASWLVKFLLHSGYNVKASVRDPNDPKKTQHLLSLGGAKERLHL  60
            M+   K VCVTGASGYIASW+VK LL  GY V+A+VR+P D  KT+HLL+L GAKERL L
Sbjct  1    MNCGGKVVCVTGASGYIASWIVKLLLLRGYTVRATVRNPTDKAKTEHLLALEGAKERLQL  60

Query  61   FKANLLEEGSFDAVVDGCEGVFHTASPFYYSVTDPQAELLDPAVKGTLNLLGSCAKAPSV  120
            FKA+LLEE SF+  + GC+ VFHTASP  Y VTDPQ EL+DPAVKGT+N+L +C K  SV
Sbjct  61   FKADLLEECSFEQAIQGCDAVFHTASPVKYIVTDPQTELIDPAVKGTINVLNTCKKTSSV  120

Query  121  KRVVLTSSIAAVAYSGQPRTPEVVVDESWWTSPDYCKEKQLWYVLSKTLAEDAAWKFVKE  180
            KRV+LTSS AAV    +P  P  VVDE++++ P  C E +LWY LSKTLAE+AAW+F K+
Sbjct  121  KRVILTSSTAAVLV--RPLEPNDVVDETFFSDPSVCTELKLWYPLSKTLAENAAWQFTKD  178

Query  181  KGIDMVVVNPAMVIGPLLQPTLNTSSAAVLSLVNGAETYPNSSFGWVNVKDVANAHILAF  240
             G+DMVV+ P  VIGPLLQPTLN S   ++ ++NG   +   ++ +V+V+DVA AH+ A
Sbjct  179  NGMDMVVIIPGFVIGPLLQPTLNFSDGFIVDMINGKNPFNCINYRFVDVRDVALAHVKAL  238

Query  241  ENPSANGRYLMV-ERVAHYSDILKILRDLYPTMQLPEKCADD--NPLMQN---YQVSKEK  294
            E PSANGRYL+    +    +I + +R+L+P + + +   +     +M     Y+V  EK
Sbjct  239  EIPSANGRYLIDGPSMMTIYEIRETMRELFPDLCIADMNGESEMKDIMTKEIIYEVCVEK  298
```

```
Query  295  AKSLGIEFTTLEESIKETVESLKEK  319
             K+LGIEFT L+ S+ +T+ SLK+K
Sbjct  299  VKNLGIEFTPLKSSLTDTIISLKDK  323
```

>XP_009148247.1 PREDICTED: tetraketide alpha-pyrone reductase 1-like isoform X2 [Brassica rapa]
Length=288

 Score = 316 bits (810), Expect = 8e-108, Method: Compositional matrix adjust.
 Identities = 155/281 (55%), Positives = 203/281 (72%), Gaps = 3/281 (1%)

```
Query  1    MSVTAKTVCVTGASGYIASWLVKFLLHSGYNVKASVRDPNDPKKTQHLLSLGGAKERLHL  60
            M+   K VCVTGASGYIASW+VK LL  GY V+A+VR+P D  KT+HLL+L GAKERL L
Sbjct  1    MNCGGKVVCVTGASGYIASWIVKLLLLRGYTVRATVRNPTDKAKTEHLLALEGAKERLQL  60

Query  61   FKANLLEEGSFDAVVDGCEGVFHTASPFYYSVTDPQAELLDPAVKGTLNLLGSCAKAPSV  120
            FKA+LLEE SF+  + GC+ VFHTASP  Y VTDPQ EL+DPAVKGT+N+L +C K  SV
Sbjct  61   FKADLLEECSFEQAIQGCDAVFHTASPVKYIVTDPQTELIDPAVKGTINVLNTCKKTSSV  120

Query  121  KRVVLTSSIAAVAYSGQPRTPEVVVDESWWTSPDYCKEKQLWYVLSKTLAEDAAWKFVKE  180
            KRV+LTSS AAV    +P  P  VVDE++++ P  C E +LWY LSKTLAE+AAW+F K+
Sbjct  121  KRVILTSSTAAVLV--RPLEPNDVVDETFFSDPSVCTELKLWYPLSKTLAENAAWQFTKD  178

Query  181  KGIDMVVVNPAMVIGPLLQPTLNTSSAAVLSLVNGAETYPNSSFGWVNVKDVANAHILAF  240
             G+DMVV+ P  VIGPLLQPTLN S   ++ ++NG   +   ++ +V+V+DVA AH+ A
Sbjct  179  NGMDMVVIIPGFVIGPLLQPTLNFSDGFIVDMINGKNPFNCINYRFVDVRDVALAHVKAL  238

Query  241  ENPSANGRYLMV-ERVAHYSDILKILRDLYPTMQLPEKCAD  280
            E PSANGRYL+    +    +I + +R+L+P + + +   D
Sbjct  239  EIPSANGRYLIDGPSMMTIYEIRETMRELFPDLCIADISFD  279
```

>XP_009127681.1 PREDICTED: cinnamoyl-CoA reductase 1-like isoform X2 [Brassica rapa]
Length=293

 Score = 303 bits (775), Expect = 2e-102, Method: Compositional matrix adjust.
 Identities = 163/322 (51%), Positives = 207/322 (64%), Gaps = 35/322 (11%)

```
Query  1    MSVTAKTVCVTGASGYIASWLVKFLLHSGYNVKASVRDPNDPKKTQHLLSLGGAKERLHL  60
            M+   K VCVTGASGYIASW+VK LL  GY V+A+VRDPN+ KKT HLL L GAKERL L
Sbjct  1    MNGEGKVVCVTGASGYIASWIVKLLLQRGYTVRATVRDPNNQKKTDHLLQLDGAKERLSL  60

Query  61   FKANLLEEGSFDAVVDGCEGVFHTASPFYYSVTDPQAELLDPAVKGTLNLLGSCAKAPSV  120
            F ANLLEEGSF   +DGCE VFHTASP   +  DPQAEL++PAVKGTLN+L +C K  SV
Sbjct  61   FGANLLEEGSFQHAIDGCEAVFHTASPVLLTAEDPQAELIEPAVKGTLNVLKTCVKMSSV  120

Query  121  KRVVLTSSIAAVAYSGQPRTPEVVVDESWWTSPDYCKEKQLWYVLSKTLAEDAAWKFVKE  180
            KRV+LTSS+AAV     P  P  VVDE+ ++ P +C++++ WY LSKTLAED A
Sbjct  121  KRVILTSSMAAVIAHASPTGPNGVVDETMFSDPSFCEQRKQWYALSKTLAEDEA------  174

Query  181  KGIDMVVVNPAMVIGPLLQPTLNTSSAAVLSLVNGAETYPNSSFGWVNVKDVANAHILAF  240
                                         V+ L  G + + + S+ +V+V+DV+ AHI A
Sbjct  175  -------------------------CVVVELTKGKDPFMSKSYRFVDVRDVSLAHIKAL  208

Query  241  ENPSANGRYLMVERV-AHYSDILKILRDLYPTMQL-PEKCADDNPL-MQNYQVSKEKAKS  297
            E PSANGRY++   V A   DI K+LR+  P + +  +K  +D  L +  Y+VS EK +S
Sbjct  209  ETPSANGRYIIDGPVIATLKDIEKVLREFVPDLCIGDDKNNEDIDLDLVTYKVSVEKVRS  268

Query  298  LGIEFTTLEESIKETVESLKEK  319
            LGIEFT  E S+++TV SLKEK
Sbjct  269  LGIEFTPTETSLRDTVLSLKEK  290
```

>XP_009120358.1 PREDICTED: cinnamoyl-CoA reductase 1 [Brassica rapa]
Length=323

 Score = 300 bits (767), Expect = 7e-101, Method: Compositional matrix adjust.

```
 Identities = 160/325 (49%), Positives = 218/325 (67%), Gaps = 6/325 (2%)

Query  1    MSVTAKTVCVTGASGYIASWLVKFLLHSGYNVKASVRDPNDPKKTQHLLSLGGAKERLHL  60
            MS   + VCVTGASG I SWLV  LL  GY+V A+V++  D K+T+HL +L GA  RLHL
Sbjct  1    MSTEREVVCVTGASGCIGSWLVHLLLLRGYSVHATVKNLQDEKETKHLEALEGAATRLHL  60

Query  61   FKANLLEEGSFDAVVDGCEGVFHTASPFYYS-VTDPQAELLDPAVKGTLNLLGSCAKAPS  119
            F+ +LL+  +  A V+GC GVFH ASP     V DP+ +LLDPAVKGTLN+L + AK
Sbjct  61   FEMDLLKYDTVSAAVNGCAGVFHLASPCIVDEVQDPEKQLLDPAVKGTLNVL-TAAKEAG  119

Query  120  VKRVVLTSSIAAVAYSGQPRTP-EVVVDESWWTSPDYCKEKQLWYVLSKTLAEDAAWKFV  178
            VKRVV+TSSI+A+  S  P  P + + +E  W   D+CK+  LWY LSKTLAE AAW+F
Sbjct  120  VKRVVVTSSISAITPS--PNWPADKIKNEDCWADEDFCKQNGLWYPLSKTLAEKAAWEFA  177

Query  179  KEKGIDMVVVNPAMVIGPLLQPTLNTSSAAVLSLVNGA-ETYPNSSFGWVNVKDVANAHI  237
            +EKG+D+VVVNP  V+GP++ P++N S   +  L+ G  ETY N   G V+ KDVA AHI
Sbjct  178  EEKGLDVVVVNPGTVMGPVIPPSINASMLMLQRLLEGCTETYENFFMGSVHFKDVALAHI  237

Query  238  LAFENPSANGRYLMVERVAHYSDILKILRDLYPTMQLPEKCADDNPLMQNYQVSKEKAKS  297
            L +ENPSA GR+L VE ++HY D +  + +LYP   +P+   +  P +   + + +K
Sbjct  238  LVYENPSAKGRHLCVEAISHYGDFVAKVAELYPNYSVPKLPRETQPGLLRAKNAAKKLME  297

Query  298  LGIEFTTLEESIKETVESLKEKKFF  322
            LG+EF+++E+ IK+ VESLK K +
Sbjct  298  LGLEFSSMEDIIKDGVESLKSKGYI  322


>XP_009109293.1 PREDICTED: tetraketide alpha-pyrone reductase 1-like [Brassica
rapa]
Length=326

 Score = 291 bits (744),  Expect = 3e-97, Method: Compositional matrix adjust.
 Identities = 151/324 (47%), Positives = 206/324 (64%), Gaps = 5/324 (2%)

Query  1    MSVTAKTVCVTGASGYIASWLVKFLLHSGYNVKASVRDPNDPKKTQHLLSLGGAKERLHL  60
            M  T   VCVTGASG++ASWLVK LL  GY V  +VRDP + KK  HL  L GAKERL L
Sbjct  1    MDQTKGKVCVTGASGFLASWLVKRLLLEGYEVTGTVRDPGNEKKLAHLWKLEGAKERLRL  60

Query  61   FKANLLEEGSFDAVVDGCEGVFHTASPFYYSVTDPQAELLDPAVKGTLNLLGSCAKAPSV  120
             KA+L+E+GSFD  + GC GVFHTASP     ++P+ E+L PA++GTLN+L SC K  S+
Sbjct  61   VKADLMEDGSFDNAIMGCHGVFHTASPVLKPTSNPEEEILKPAIEGTLNVLRSCRKNQSL  120

Query  121  KRVVLTSSIAAVAYSGQPRTPEVVVDESWWTSPDYCKEKQLWYVLSKTLAEDAAWKFVKE  180
            KRVVLTSS + V        P + +DES WTS + CK  Q+WY LSKTLAE AAWKF +E
Sbjct  121  KRVVLTSSSSTVRIRDD-FDPNIPLDESVWTSVELCKRFQVWYALSKTLAEQAAWKFCEE  179

Query  181  KGIDMVVVNPAMVIGPLLQPTLNTSSAAVLSLVNG-AETYP-NSSFGWVNVKDVANAHIL  238
              ID+V V P+ ++GP L P L ++++ VL L+ G  E +  +   G++++ DVA  HIL
Sbjct  180  NSIDLVTVLPSFLVGPSLPPDLCSTASDVLGLLKGETEKFQWHGQMGYIHIDDVARTHIL  239

Query  239  AFENPSANGRYLMVERVAHYSDILKILRDLYPTMQLPEKCADDNPLMQNYQVSKEKAKSL  298
             FE  +A GRY+   +V    +++  L   YP++ +P++    N L  +Y +   K KSL
Sbjct  240  VFEQEAAKGRYICSSKVVSLEELVSFLSTRYPSLPIPKRFKKLNRL--HYDLDTSKIKSL  297

Query  299  GIEFTTLEESIKETVESLKEKKFF  322
            G+EF  LEE   + + S  E+ +
Sbjct  298  GLEFKPLEEMFDDCIASFVEQGYL  321


>XP_009117788.1 PREDICTED: cinnamoyl-CoA reductase 1 [Brassica rapa]
Length=343

 Score = 291 bits (745),  Expect = 3e-97, Method: Compositional matrix adjust.
 Identities = 162/317 (51%), Positives = 221/317 (70%), Gaps = 9/317 (3%)

Query  5    AKTVCVTGASGYIASWLVKFLLHSGYNVKASVRDPNDPKKTQHLLSLGGAKERLHLFKAN  64
             KTVCVTGA GYIASW+VK LL  GY VK +VR+P+DPK T HL  L GAKERL L KA+
Sbjct  10   GKTVCVTGAGGYIASWIVKLLLERGYTVKGTVRNPDDPKNT-HLRELEGAKERLILCKAD  68
```

```
Query  65   LLEEGSFDAVVDGCEGVFHTASPFYYSVTDPQAELLDPAVKGTLNLLGSCAKAPSVKRVV  124
            L +  +  A +DGC+GVFHTASP    VTD   ++++PAV G   ++ + A+A  VKRVV
Sbjct  69   LQDYDALKAAIDGCDGVFHTASP----VTDDPEQMVEPAVNGAKFVINAAAEA-KVKRVV  123

Query  125  LTSSIAAVAYSGQPRTPEVVVDESWWTSPDYCKEKQLWYVLSKTLAEDAAWKFVKEKGID  184
            +TSSI AV Y    R PE VVDES W+  ++CK  + WY   K +AE AAW+  +EKG+D
Sbjct  124  ITSSIGAV-YMDPNRDPEAVVDESCWSDLEFCKNTKNWYCYGKMVAEQAAWETAEEKGVD  182

Query  185  MVVVNPAMVIGPLLQPTLNTSSAAVLSLVNG-AETYPNSSFGWVNVKDVANAHILAFENP  243
            +VV+NP +V+GP LQPT+N S   VL  + G A+TY N +  +V+V+DVA AH+L +E P
Sbjct  183  LVVLNPVLVLGPPLQPTINASLFHVLKYLTGSAKTYANLTQAYVDVRDVALAHVLVYEAP  242

Query  244  SANGRYLMVERVAHYSDILKILRDLYPTMQLPEKCADD-NPLMQNYQVSKEKAKSLGIEF  302
            SA+GRYL+ E   H  ++++IL  L+P   LP KC D+ NP  + Y+ + +K K LG+EF
Sbjct  243  SASGRYLLAETALHRGEVVEILAKLFPEYPLPTKCKDEKNPRAKPYKFTNQKIKDLGLEF  302

Query  303  TTLEESIKETVESLKEK  319
            T+ ++S+ +TV+SL+EK
Sbjct  303  TSTKQSLYDTVKSLQEK  319


>XP_009102146.1 PREDICTED: tetraketide alpha-pyrone reductase 1 [Brassica rapa]
Length=328

 Score = 290 bits (743),  Expect = 3e-97, Method: Compositional matrix adjust.
 Identities = 150/317 (47%), Positives = 207/317 (65%), Gaps = 5/317 (2%)

Query  8    VCVTGASGYIASWLVKFLLHSGYNVKASVRDPNDPKKTQHLLSLGGAKERLHLFKANLLE  67
            VCVTGASG++ASWLVK LL  GY V  +VRDP + KK  HL  L GAKERL L KA+L+E
Sbjct  8    VCVTGASGFLASWLVKRLLLEGYEVIGTVRDPGNEKKLAHLRKLEGAKERLRLVKADLME  67

Query  68   EGSFDAVVDGCEGVFHTASPFYYSVTDPQAELLDPAVKGTLNLLGSCAKAPSVKRVVLTS  127
            +GSFD  + GC+GVFHTASP     ++P+ E+L PA++GTLN+L SC K  S+KRVVLTS
Sbjct  68   DGSFDKAIMGCQGVFHTASPVLKPTSNPEEEILKPAIEGTLNVLRSCMKNQSLKRVVLTS  127

Query  128  SIAAVAYSGQPRTPEVVVDESWWTSPDYCKEKQLWYVLSKTLAEDAAWKFVKEKGIDMVV  187
            S + V        P + +DES WTS + CK  Q+WY LSKTLAE AAWKF +E GID+V
Sbjct  128  SSSTVRIRDD-FDPNIPLDESVWTSVELCKRFQVWYALSKTLAEQAAWKFCEENGIDLVT  186

Query  188  VNPAMVIGPLLQPTLNTSSAAVLSLVNG-AETYP-NSSFGWVNVKDVANAHILAFENPSA  245
            V P+ ++GP L P L ++++ VL L+ G  E +  +   G+V++ DVA+ HIL FE+ +A
Sbjct  187  VLPSFLVGPSLPPDLCSTASDVLGLLKGETEKFQWHGQMGYVHIDDVASTHILVFEHEAA  246

Query  246  NGRYLMVERVAHYSDILKILRDLYPTMQLPEKCADDNPLMQNYQVSKEKAKSLGIEFTTL  305
             GRY+         +++  L   YP++ +P++    N L  ++  S  K KSLG++F +L
Sbjct  247  QGRYICSSNFVSLEELVSFLSTRYPSLPIPKRFEKLNRLHYDFDTS--KIKSLGLKFKSL  304

Query  306  EESIKETVESLKEKKFF  322
            EE   + + S  EK +
Sbjct  305  EEMFDDCIASFVEKGYL  321


>XP_009149037.1 PREDICTED: cinnamoyl-CoA reductase 1 [Brassica rapa]
Length=341

 Score = 290 bits (743),  Expect = 7e-97, Method: Compositional matrix adjust.
 Identities = 162/320 (51%), Positives = 221/320 (69%), Gaps = 9/320 (3%)

Query  2    SVTAKTVCVTGASGYIASWLVKFLLHSGYNVKASVRDPNDPKKTQHLLSLGGAKERLHLF  61
            S   KTVCVTGA GYIASW+VK LL  GY VK +VR+P+DPK T HL  L GAKERL L
Sbjct  7    SPAGKTVCVTGAGGYIASWIVKLLLERGYTVKGTVRNPDDPKNT-HLRELEGAKERLILC  65

Query  62   KANLLEEGSFDAVVDGCEGVFHTASPFYYSVTDPQAELLDPAVKGTLNLLGSCAKAPSVK  121
            KA+L +  +    +DGC+GVFHTASP    VTD   ++++PAV G   ++ + A+A  VK
Sbjct  66   KADLQDYEALKTAIDGCDGVFHTASP----VTDDPEQMVEPAVNGAKFVINAAAEA-KVK  120

Query  122  RVVLTSSIAAVAYSGQPRTPEVVVDESWWTSPDYCKEKQLWYVLSKTLAEDAAWKFVKEK  181
            RVV+TSSI AV Y    R PE VVDES W+  ++CK  + WY   K +AE AAW+  KEK
Sbjct  121  RVVITSSIGAV-YMDPNRDPEAVVDESCWSDLEFCKNTKNWYCYGKMVAEQAAWETAKEK  179
```

```
Query  182  GIDMVVVNPAMVIGPLLQPTLNTSSAAVLSLVNG-AETYPNSSFGWVNVKDVANAHILAF  240
            G+D+VV+NP +V+GP LQPT+N S   VL  + G A+TY N +  +V+V+DVA AH+L +
Sbjct  180  GVDLVVLNPVLVLGPPLQPTINASLFHVLKYLTGSAKTYANLTQAYVDVRDVALAHVLVY  239

Query  241  ENPSANGRYLMVERVAHYSDILKILRDLYPTMQLPEKCADD-NPLMQNYQVSKEKAKSLG  299
            E P+A+GRYL+ E   H  ++++IL  L+P   LP KC D+ NP  + Y+ + +K K LG
Sbjct  240  EAPTASGRYLLAESALHRGEVVEILAKLFPEYPLPTKCKDENNPRAKPYKFTNQKIKDLG  299

Query  300  IEFTTLEESIKETVESLKEK  319
            +EFT+ ++S+ +TV+SL+EK
Sbjct  300  LEFTSTKQSLYDTVKSLQEK  319


>XP_009121757.1 PREDICTED: tetraketide alpha-pyrone reductase 2 [Brassica rapa]
Length=321

 Score = 262 bits (669),  Expect = 5e-86, Method: Compositional matrix adjust.
 Identities = 139/318 (44%), Positives = 202/318 (64%), Gaps = 13/318 (4%)

Query  10   VTGASGYIASWLVKFLLHSGYNVKASVRDPNDPKKTQHLLSLGGAKERLHLFKANLLEEG  69
            VTG +G+IAS+++K LL  G+ V+ +VR+P D +K   L  L GAKERL +FKA+L  +G
Sbjct  6    VTGGTGFIASYIIKSLLELGHTVRTTVRNPQDEEKVGFLWELKGAKERLKMFKADLTVDG  65

Query  70   SFDAVVDGCEGVFHTASPFYYSVT-DPQAELLDPAVKGTLNLLGSCAK-APSVKRVVLTS  127
            SFD  V+G +GVFHTASP       + Q  L+DP +KGT N++ SCAK   ++KR+VLTS
Sbjct  66   SFDEAVNGVDGVFHTASPVLVPQDHNIQETLVDPIIKGTTNVMNSCAKPKTTLKRIVLTS  125

Query  128  SIAAVAYSGQPRTPEVVVDESWWTSPDYCKEKQLWYVLSKTLAEDAAWKFVKEKGIDMVV  187
            S +++ Y     T    ++ES W+ PDYCK   LWY  +KTL E  AW+  +EKG+++VV
Sbjct  126  SCSSIRYRFDA-TKASPLNESHWSDPDYCKRFNLWYAYAKTLGEKEAWRIAEEKGLNLVV  184

Query  188  VNPAMVIGPLLQPTLNTSSAAVLSLVNG-AETYPNSSFGWVNVKDVANAHILAFENPSAN  246
            VNP+ V+GPLL P   ++   +L++V G A  YPN + G+V++ DV  AH+LA E P A+
Sbjct  185  VNPSFVVGPLLGPKPTSTLLYILAIVKGLAGEYPNLTVGFVHIDDVVAAHVLAMEEPKAS  244

Query  247  GRYLMVERVAHYSDILKILRDLYPTMQLPEKCA----DDNPLMQNYQVSKEKAKSLGI-E  301
            GR +    VAH+S+I+++LR+ YP   L  KC+    D+NP    + +   K   LG
Sbjct  245  GRIVCSSSVAHWSEIIELLRNKYPNYPLENKCSNKEGDNNP----HSMDTRKIHELGFAS  300

Query  302  FTTLEESIKETVESLKEK  319
            F +L E   + + S +EK
Sbjct  301  FKSLLEMFDDCIRSFQEK  318


>XP_009105396.1 PREDICTED: tetraketide alpha-pyrone reductase 2-like [Brassica
rapa]
Length=321

 Score = 261 bits (667),  Expect = 8e-86, Method: Compositional matrix adjust.
 Identities = 136/314 (43%), Positives = 199/314 (63%), Gaps = 5/314 (2%)

Query  10   VTGASGYIASWLVKFLLHSGYNVKASVRDPNDPKKTQHLLSLGGAKERLHLFKANLLEEG  69
            VTG +G+IAS+++K LL  G+ V+ +VR+P D +K   L  L GAKERL +FKA+L  +G
Sbjct  6    VTGGTGFIASYIIKSLLELGHTVRTTVRNPQDEEKVGFLWELKGAKERLKMFKADLTVDG  65

Query  70   SFDAVVDGCEGVFHTASPFYYSVT-DPQAELLDPAVKGTLNLLGSCAKAP-SVKRVVLTS  127
            SF+  V+G +GVFHTASP       + Q  L+DP +KGT N++ SCAK+  ++KR+VLTS
Sbjct  66   SFNEAVNGVDGVFHTASPVLVPQDHNIQETLVDPIIKGTTNVMNSCAKSKTTLKRIVLTS  125

Query  128  SIAAVAYSGQPRTPEVVVDESWWTSPDYCKEKQLWYVLSKTLAEDAAWKFVKEKGIDMVV  187
            S +++ Y     T    ++ES W+ PDYCK   LWY  +KTL E  AW+  +EKG+++VV
Sbjct  126  SCSSIRYCFDA-TKASPLNESHWSDPDYCKRFNLWYAYAKTLGEKEAWRIAEEKGLNLVV  184

Query  188  VNPAMVIGPLLQPTLNTSSAAVLSLVNG-AETYPNSSFGWVNVKDVANAHILAFENPSAN  246
            VNP+ V+GPLL P   ++   +L++V G A  YPN + G+V++ DV  AH+LA E P A+
Sbjct  185  VNPSFVVGPLLGPKPTSTLLYILAIVKGLAGEYPNLTVGFVHIDDVVAAHVLAMEEPKAS  244

Query  247  GRYLMVERVAHYSDILKILRDLYPTMQLPEKCADDNPLMQNYQVSKEKAKSLGI-EFTTL  305
```

```
             GR +    VAH+S+I+ +LR+ YP   L  KC+D       + +   K   LG   F +L
Sbjct  245  GRIVCSSSVAHWSEIIVLLRNKYPNYPLESKCSDKEGDNNPHSMDTRKMHELGFASFKSL  304

Query  306  EESIKETVESLKEK  319
             E   + + S +EK
Sbjct  305  PEMFDDCIRSFQEK  318
```

```
>XP_009129149.1 PREDICTED: cinnamoyl-CoA reductase 1 [Brassica rapa]
Length=321

 Score = 240 bits (612),  Expect = 2e-77, Method: Compositional matrix adjust.
 Identities = 131/321 (41%), Positives = 203/321 (63%), Gaps = 7/321 (2%)

Query  6    KTVCVTGASGYIASWLVKFLLHSGYN-VKASVRDPNDPKKTQHLLSLGGAKERLHLFKAN  64
            +TVCVTGA+G+I SW+++ LL +GY  + AS+   +DP      L          + +F+A+
Sbjct  4    ETVCVTGANGFIGSWIIRTLLDNGYTKIHASIYPGSDPTHLLKLPRPDDTNTEIKIFEAD  63

Query  65   LLEEGSFDAVVDGCEGVFHTASPFYYSV-TDPQAELLDPAVKGTLNLLGSCAKAPSVKRV  123
            LL+  +    VDGC GVFH ASP       DP+ EL++PAVKGT+N+L + A   +V+RV
Sbjct  64   LLDPDAIARAVDGCAGVFHVASPCTLDPPEDPEKELVEPAVKGTINVLLA-ANRFNVRRV  122

Query  124  VLTSSIAAVAYSGQPRTPE-VVVDESWWTSPDYCKEKQLWYVLSKTLAEDAAWKFVKEKG  182
            V+TSSI+A+  +  P  PE   VDES WT  DYCK  Q WY +SKTLAE AAW+F ++
Sbjct  123  VITSSISALVTN--PNWPEGKPVDESSWTDLDYCKSMQKWYPISKTLAEKAAWEFSEKHR  180

Query  183  IDMVVVNPAMVIGPLLQPTLNTSSAAVLSLVNGA-ETYPNSSFGWVNVKDVANAHILAFE  241
             ++V ++P+  +GPLLQP+LN S A +L L+ G+ ET  +   G V+V+DVA AH++ FE
Sbjct  181  TNVVTIHPSTCLGPLLQPSLNASCAVLLQLLQGSTETQEHHWLGVVHVRDVAKAHVMLFE  240

Query  242  NPSANGRYLMVERVAHYSDILKILRDLYPTMQLPEKCADDNPLMQNYQVSKEKAKSLGIE  301
             P A+GR+L    +  +S+   ++  L+P   + +   +  P +   + + ++   LG+
Sbjct  241  TPEASGRFLCSNGIYQFSEFAALVSKLFPEFDVHKFDKETQPGLTPCKDAAKRLIELGMV  300

Query  302  FTTLEESIKETVESLKEKKFF  322
            FT +E+++KETV+S+++K F
Sbjct  301  FTPVEDAVKETVQSIRDKGFL  321
```

```
>XP_009141331.1 PREDICTED: cinnamoyl-CoA reductase 1 [Brassica rapa]
Length=321

 Score = 227 bits (579),  Expect = 1e-72, Method: Compositional matrix adjust.
 Identities = 136/326 (42%), Positives = 196/326 (60%), Gaps = 10/326 (3%)

Query  1    MSVTAK-TVCVTGASGYIASWLVKFLLHSGYNVKASVRDPNDPKKTQHLLSLGGAKERLH  59
            MSV AK  VCVTGA G++ASW+V  LL   Y V  +VRDP D +K  HL  L  A ++L
Sbjct  1    MSVAAKGKVCVTGAGGFLASWVVDLLLSKDYFVHGTVRDP-DNEKYSHLKKLEKAGDKLK  59

Query  60   LFKANLLEEGSFDAVVDGCEGVFHTASPF-YYSVTDPQAELLDPAVKGTLNLLGSCAKAP  118
            L KA+LL+  S  + + GC GVFH ASP    SV +P+ E++ PAV GTLN+L +C +A
Sbjct  60   LVKADLLDYPSLQSAIAGCIGVFHVASPVPSSSVPNPEVEVMSPAVDGTLNVLKACVEA-  118

Query  119  SVKRVVLTSSIAAVAYSGQPRTPEVVVDESWWTSPDYCKEKQLWYVLSKTLAEDAAWKFV  178
            +VKRVV  SS AA+  +    + + V+DES W+  ++CK  + WY  SKT AE  A++F
Sbjct  119  NVKRVVYVSSAAALMMNPN-WSKDRVIDESCWSDLEFCKRTENWYCASKTQAESEAFEFA  177

Query  179  KEKGIDMVVVNPAMVIGPLLQP-TLNTSSAAVLSLVN-GAETYPNSSFGWVNVKDVANAH  236
            K  GI +V + P MV GP+LQ  T+N S+ A+  L+  G E+  N     V+V+DVA A
Sbjct  178  KRTGISLVSICPTMVFGPVLQQHTVNASTLALAKLLKEGFESRENQVRLIVDVRDVAQAL  237

Query  237  ILAFENPSANGRYLMVERVAHYSDILKILRDLYPTMQLPEKCADDNPLMQNYQVSKEKAK  296
            +L +E P A GRY+     A   D+++ L+ LYP    P+   +   + +  ++ EK +
Sbjct  238  LLVYEKPEAEGRYICTAHKAKEKDVVEKLKSLYPNYNYPKSYVE---VEERSTMTSEKLQ  294

Query  297  SLGIEFTTLEESIKETVESLKEKKFF  322
             LG  F  LEE++ ++VES ++ K
Sbjct  295  KLGWSFRPLEETLVDSVESYRKAKIL  320
```

```
>XP_009115319.1 PREDICTED: tetraketide alpha-pyrone reductase 2-like [Brassica
rapa]
Length=363

 Score = 226 bits (577),  Expect = 1e-71, Method: Compositional matrix adjust.
 Identities = 126/314 (40%), Positives = 188/314 (60%), Gaps = 5/314 (2%)

Query  10   VTGASGYIASWLVKFLLHSGYNVKASVRDPNDPKKTQHLLSLGGAKERLHLFKANLLEEG  69
            VTG + +IAS +VK LL  G++V+ +VRD +D +K + L  L GAKERL +F+A+L  EG
Sbjct  48   VTGGTSFIASHVVKALLDLGHSVRTTVRDSSDEEKVRFLWELKGAKERLKIFEADLTVEG  107

Query  70   SFDAVVDGCEGVFHTASPFYYSVTDPQAE-LLDPAVKGTLNLLGSCAKAP-SVKRVVLTS  127
            SFD  V G +GVFH AS     +     E L+D  + GT NL+ SC K+  +VKR+VLTS
Sbjct  108  SFDEAVKGVDGVFHIASRVTVCLDKNDLEKLVDRDINGTRNLMNSCEKSRNTVKRIVLTS  167

Query  128  SIAAVAYSGQPRTPEVVVDESWWTSPDYCKEKQLWYVLSKTLAEDAAWKFVKEKGIDMVV  187
            S  AV Y     T    ++ES ++  DYC+  ++WY  +KTL E  AW    EK +D+VV
Sbjct  168  SSTAVRYRYDA-TEASPLNESHYSDLDYCRNFKIWYGYAKTLGEKEAWTIAAEKNLDLVV  226

Query  188  VNPAMVIGPLLQPTLNTSSAAVLSLVNGAE-TYPNSSFGWVNVKDVANAHILAFENPSAN  246
            V P+  IGP+L P   +S   +LS++ G    YPN + G+V+++DV  A ILA E P A+
Sbjct  227  VIPSFCIGPILSPEPTSSPLILLSIIKGVRGDYPNVTGGFVHIEDVVAAQILAMEEPKAS  286

Query  247  GRYLMVERVAHYSDILKILRDLYPTMQLPEKCADDNPLMQNYQVSKEKAKSLGI-EFTTL  305
            GR++    VAH+S+I+++LR  YP      KC+ +      + +   K + LG+  F +L
Sbjct  287  GRFICSSSVAHWSEIIEMLRPKYPLYPFETKCSSEEGKDMPHSLDTTKIRELGLPPFKSL  346

Query  306  EESIKETVESLKEK  319
             E   + ++  ++K
Sbjct  347  AEMFDDCIKCFQDK  360


>XP_009115320.1 PREDICTED: tetraketide alpha-pyrone reductase 2 isoform X1
[Brassica
rapa]
Length=321

 Score = 208 bits (530),  Expect = 3e-65, Method: Compositional matrix adjust.
 Identities = 122/314 (39%), Positives = 178/314 (57%), Gaps = 5/314 (2%)

Query  10   VTGASGYIASWLVKFLLHSGYNVKASVRDPNDPKKTQHLLSLGGAKERLHLFKANLLEEG  69
            VTG + +IAS +VK LL  GY V+ +VRD +  +K + L  L GAKERL +F+A+L  +G
Sbjct  6    VTGGTSFIASHVVKALLDFGYFVRTTVRDSSYEEKVRFLWELKGAKERLKIFEADLTVDG  65

Query  70   SFDAVVDGCEGVFHTASPFYYSVTDPQAE-LLDPAVKGTLNLLGSCAKAP-SVKRVVLTS  127
            SFD  V G +GVFH AS     + D   E L D  + GT NL+ SC K+  +VKR+VLTS
Sbjct  66   SFDDAVKGVDGVFHIASRISVCLDDNDLEKLADRDIYGTRNLMNSCEKSRNTVKRIVLTS  125

Query  128  SIAAVAYSGQPRTPEVVVDESWWTSPDYCKEKQLWYVLSKTLAEDAAWKFVKEKGIDMVV  187
            S  +V YS    T   +  S W+  +Y +   +WY  +KTL E  AW    EK +++VV
Sbjct  126  SSTSVRYSYDA-TKASPLKGSHWSDLEYFRSFSIWYGYAKTLVEKEAWLIAAEKNLNLVV  184

Query  188  VNPAMVIGPLLQPTLNTSSAAVLSLVNGAE-TYPNSSFGWVNVKDVANAHILAFENPSAN  246
            V P+  IGP+L P   +S +  LS++ G    YPN   G+V++ DVA A ILA + P A+
Sbjct  185  VIPSFCIGPILSPKPTSSPSIFLSIIKGVHGEYPNFRGGFVHIDDVAAAQILAMKEPKAS  244

Query  247  GRYLMVERVAHYSDILKILRDLYPTMQLPEKCADDNPLMQNYQVSKEKAKSLGI-EFTTL  305
            GR +    VAH+S+I+++L+  YP      KC  +      + +   K   LG   F +L
Sbjct  245  GRIICSSSVAHWSEIIEMLKPKYPLYPFETKCGSEEGRDMPHSLDTRKIHELGFGSFKSL  304

Query  306  EESIKETVESLKEK  319
             E   + ++  ++K
Sbjct  305  AEMFDDCIKCFQDK  318


>XP_009106316.1 PREDICTED: cinnamoyl-CoA reductase 2 [Brassica rapa]
Length=329
```

```
 Score = 199 bits (507),  Expect = 1e-61, Method: Compositional matrix adjust.
 Identities = 126/324 (39%), Positives = 189/324 (58%), Gaps = 19/324 (6%)

Query  6    KTVCVTGASGYIASWLVKFLLHSGYNVKASVRDPNDPKKTQHLLSLGGAKERLHLFKANL  65
            + +CVTG+ GYIASWLVK LL  GY V  +VRDP+D +K  HL  L  A + L LFKA+L
Sbjct  5    QRICVTGSGGYIASWLVKSLLSRGYTVHGTVRDPSD-RKNDHLKKLDDASKNLKLFKADL  63

Query  66   LEEGSFDAVVDGCEGVFHTASPF-YYSVTDPQAELLDPAVKGTLNLLGSCAKAPSVKRVV  124
             +     + + GC GVFH A P  +  V   + +L+ PA+ GT N+L +C +A   K VV
Sbjct  64   FDYEGLSSAISGCYGVFHIAGPVPFEDVPLTEEQLIKPALTGTKNVLEACTEAKVKKVVV  123

Query  125  LTSSIAAVAYSGQPRTPEVVVDESWWTSPDYCKEKQ---LWYVLSKTLAEDAAWKFVKEK  181
            ++S  A V     PR  +V VDES W+   Y   ++    +Y L+KTL E  A ++ +
Sbjct  124  VSSIAAVVYNPKWPR--DVDVDESCWSDTQYLYSREGYWSYYFLAKTLMEREAIEWSRTS  181

Query  182  GIDMVVVNPAMVIGPLLQPTLNTSSAAVLSLVNGAE-TYPNSSFGWVNVKDVANAHILAF  240
              D+V V P++VIGP LQ TLN+SS  +L+ + G   +  +     V+V+DVA+A +L +
Sbjct  182  SADVVTVCPSVVIGPRLQSTLNSSSLGLLNFIKGGVISLLSDQLYLVDVRDVADALLLVY  241

Query  241  ENPSANGRYLMVERVAHYSDILKILRDLYPTMQLP-------EKCADDNPLMQNYQVSKE  293
            EN  A GRY+      H +D+++ L ++YP  + P       EK  ++N L    ++S E
Sbjct  242  ENQEAKGRYICNSHSLHNNDLMEKLMNMYPKRKFPKSFSERKEKQVNENIL----RISSE  297

Query  294  KAKSLGIEFTTLEESIKETVESLK  317
            K + LG +F +LEE+I ++V S +
Sbjct  298  KLEKLGWKFRSLEETIDDSVVSFE  321


>XP_009137647.1 PREDICTED: dihydroflavonol-4-reductase-like isoform X2 [Brassica
rapa]
Length=350

 Score = 197 bits (501),  Expect = 2e-60, Method: Compositional matrix adjust.
 Identities = 121/332 (36%), Positives = 186/332 (56%), Gaps = 26/332 (8%)

Query  5    AKTVCVTGASGYIASWLVKFLLHSGYNVKASVRDPNDPKKTQHLLSLGGAKERLHLFKAN  64
            A T CVTGASGYI SWLVK LL  GY V A++RD     K+Q+  S     ERL +F+++
Sbjct  10   ATTYCVTGASGYIGSWLVKSLLERGYTVHATLRDL---AKSQYFQSKWRGNERLRIFRSD  66

Query  65   LLEEGSFDAVVDGCEGVFHTASPFYYSVTDPQAEL--------LDPAVKGTLNLLGSCAK  116
            L + GSFD  V GC+GVFH A+   + ++  Q  L        +DPA+KG  N+LGSC K
Sbjct  67   LQDGGSFDDAVKGCDGVFHVAASMEFDISPDQVNLESYVQSKVIDPAIKGVRNVLGSCLK  126

Query  117  APSVKRVVLTSSIAAVAYSGQPRTPEVVVDESWWTSPDYCKEKQLWYVLSKTLAEDAAWK  176
            + SVKRVV TSSI+ +    +      +VDE+  T  D+  + ++ YVLSK ++E+ A++
Sbjct  127  SNSVKRVVFTSSISTLTAKDENERWRSIVDETCKTPIDHVLKTKV-YVLSKLVSEEEAFR  185

Query  177  FVKEKGIDMVVVNPAMVIGPLLQPTLNTSSAAVLSLVNG---------AETYPNSSFGWV  227
            + KE G+D+V V    V GP   P+L +S   +LS + G         A      S G V
Sbjct  186  YAKESGMDLVSVITTTVSGPFCTPSLPSSLQVLLSPITGDSKLFGILSAVNKRMGSIGLV  245

Query  228  NVKDVANAHILAFENPSANGRYL-MVERVAHYSDILKIL--RDLYPTMQLPEKCADDNPL  284
            +++D+  AH+   E P A G+Y+  V+ +  +  +L       L    +L +   +   L
Sbjct  246  HIEDICMAHLFLMEEPKAEGQYICCVDNIDMHELMLNHFSKEHLCKVHKLDDDLEERQSL  305

Query  285  MQNYQVSKEKAKSLGIEFT-TLEESIKETVES  315
            M+   +S +K K LG E+  +++E I++T+++
Sbjct  306  MKP-MISSKKLKDLGFEYKYSIDEIIRQTIDA  336


>XP_009137646.1 PREDICTED: dihydroflavonol-4-reductase-like isoform X1 [Brassica
rapa]
Length=354

 Score = 196 bits (497),  Expect = 8e-60, Method: Compositional matrix adjust.
 Identities = 122/335 (36%), Positives = 186/335 (56%), Gaps = 28/335 (8%)

Query  5    AKTVCVTGASGYIASWLVKFLLHSGYNVKASVRDPNDPKKTQHLLSLGGAKERLHLFKAN  64
            A T CVTGASGYI SWLVK LL  GY V A++RD     K+Q+  S     ERL +F+++
```

```
Sbjct  10   ATTYCVTGASGYIGSWLVKSLLERGYTVHATLRDL---AKSQYFQSKWRGNERLRIFRSD  66

Query  65   LLEEGSFDAVVDGCEGVFHTASPFYYSVTDPQAEL--------LDPAVKGTLNLLGSCAK  116
            L + GSFD  V GC+GVFH A+   + ++  Q  L        +DPA+KG  N+LGSC K
Sbjct  67   LQDGGSFDDAVKGCDGVFHVAASMEFDISPDQVNLESYVQSKVIDPAIKGVRNVLGSCLK  126

Query  117  APSVKRVVLTSSIAAVAYSGQPRTPEVVVDESWWTSPDYCKEKQL--W-YVLSKTLAEDA  173
            + SVKRVV TSSI+ +    +      +VDE+  T  D+  + +   W YVLSK ++E+
Sbjct  127  SNSVKRVVFTSSISTLTAKDENERWRSIVDETCKTPIDHVLKTKASGWVYVLSKLVSEEE  186

Query  174  AWKFVKEKGIDMVVVNPAMVIGPLLQPTLNTSSAAVLSLVNG---------AETYPNSSF  224
            A+++ KE G+D+V V    V GP   P+L +S   +LS + G         A      S
Sbjct  187  AFRYAKESGMDLVSVITTTVSGPFCTPSLPSSLQVLLSPITGDSKLFGILSAVNKRMGSI  246

Query  225  GWVNVKDVANAHILAFENPSANGRYL-MVERVAHYSDILKIL--RDLYPTMQLPEKCADD  281
            G V+++D+  AH+   E P A G+Y+  V+ +  +  +L        L    +L +   +
Sbjct  247  GLVHIEDICMAHLFLMEEPKAEGQYICCVDNIDMHELMLNHFSKEHLCKVHKLDDDLEER  306

Query  282  NPLMQNYQVSKEKAKSLGIEFT-TLEESIKETVES  315
              LM+   +S +K K LG E+  +++E I++T+++
Sbjct  307  QSLMKP-MISSKKLKDLGFEYKYSIDEIIRQTIDA  340


>XP_009105139.1 PREDICTED: anthocyanidin reductase [Brassica rapa]
Length=338

 Score = 184 bits (466),  Expect = 2e-55, Method: Compositional matrix adjust.
 Identities = 117/332 (35%), Positives = 187/332 (56%), Gaps = 23/332 (7%)

Query  6    KTVCVTGASGYIASWLVKFLLHSGYNVKASVRDPNDPKKTQHLLSLGGAKERLHLFKANL  65
            K VCV G +G +AS L+  LL SGY V  +VRDP + KK   L  L    + L++FKA+L
Sbjct  11   KKVCVIGGTGNLASILIDRLLQSGYEVNTTVRDPENEKKMAVLRVLQEQGD-LNIFKADL  69

Query  66   LEEGSFDAVVDGCEGVFHTASPFYYSVTDPQAELLDPAVKGTLNLLGSCAKAPSVKRVVL  125
             +EGSF++ V GCE VFH A+P  ++  DP+ ++++PA++G +N+L SC  + SVKRV+
Sbjct  70   TDEGSFNSPVSGCEYVFHVATPISFTSQDPEKDMINPAIQGVINVLKSCLNSNSVKRVIY  129

Query  126  TSSIAAVAYSGQPRTPEVVVDESWWTSPDYCKEKQLW---YVLSKTLAEDAAWKFVKEKG  182
            TSS AAV+ +     P +V+ E  W+  D+ ++++ +   Y +SK LAE AA++F +E
Sbjct  130  TSSAAAVSINNLS-GPGLVMTEENWSDIDFLRKEKPFNWAYPISKVLAEKAAYQFAQENK  188

Query  183  IDMVVVNPAMVIG-PLLQPTLNTSSAAVLSLVNGAETYPNS---------SFGWVNVKDV  232
            ID+V V PA++ G  L+     +S +  +SL+   E + N+         S  +++V D+
Sbjct  189  IDLVTVVPALIAGNTLIDDPPPSSLSLSMSLITRKEMHLNALKEMQKLSGSISFIHVDDL  248

Query  233  ANAHILAFENPSANGRYLMVERVAHYSDILKILRDLYPTMQLP---EKCADDNPLMQNYQ  289
            A AH+   E  +A+GRY+      +  ++   LR  YP   +    E+C     L
Sbjct  249  ACAHLFLAEKETASGRYICCSYNTNIPELADFLRKRYPRYNVLSEFEECLSTAKLT----  304

Query  290  VSKEKAKSLGIEFT-TLEESIKETVESLKEKK  320
            +S EK  + G +F   +EE   + VE  K  +
Sbjct  305  LSSEKLINEGFQFEHDIEEMYDQMVEHFKTNR  336


>XP_009113238.1 PREDICTED: anthocyanidin reductase-like [Brassica rapa]
Length=345

 Score = 183 bits (464),  Expect = 4e-55, Method: Compositional matrix adjust.
 Identities = 109/276 (39%), Positives = 162/276 (59%), Gaps = 14/276 (5%)

Query  6    KTVCVTGASGYIASWLVKFLLHSGYNVKASVRDPNDPKKTQHLLSLGGAKERLHLFKANL  65
            K  CV G +G +AS L+K LL SGY V  +VRDP + KK  HL  L    + L +FKA+L
Sbjct  14   KKACVIGGTGNLASILIKHLLQSGYKVNTTVRDPENEKKMAHLKVLQELGD-LKIFKADL  72

Query  66   LEEGSFDAVVDGCEGVFHTASPFYYSVTDPQAELLDPAVKGTLNLLGSCAKAPSVKRVVL  125
             +EGSF + + GCE VFH A+P  ++  DP+ +++ PAV+G +N+L SC K+ S+KRV+
Sbjct  73   TDEGSFTSPISGCEYVFHVATPISFTSQDPEKDMIKPAVRGVINVLKSCLKSNSIKRVIY  132

Query  126  TSSIAAVAYSGQPRTPEVVVDESWWTSPDY-CKEKQL-W-YVLSKTLAEDAAWKFVKEKG  182
```

```
            TSS AAV+ +     P +V+ E  W+  D+  KEK   W Y +SKTLAE  A+K+ +E
Sbjct  133  TSSAAAVSINNLSE-PGLVMTEENWSDVDFLTKEKPFNWGYPVSKTLAEKEAYKYAEENK  191

Query  183  IDMVVVNPAMVIGPLLQPTLNTSSAAVLSLVNGAETY---------PNSSFGWVNVKDVA  233
            ID+V V PA++ G  L     +S +  +SL+ G E +          + S  +++V D+A
Sbjct  192  IDLVTVVPALIAGNSLLSDPPSSLSLSMSLITGKEMHLSGLKEMQKLSGSISFIHVDDLA  251

Query  234  NAHILAFENPSANGRYLMVERVAHYSDILKILRDLY  269
             AH+   E  +A+GRY+      +  +I   LR  Y
Sbjct  252  RAHMFLAEKETASGRYICCYYNTNVPEIADFLRRRY  287


>XP_009115321.1 PREDICTED: tetraketide alpha-pyrone reductase 2 isoform X2
[Brassica
rapa]
Length=294

 Score = 180 bits (456),  Expect = 2e-54, Method: Compositional matrix adjust.
 Identities = 114/313 (36%), Positives = 164/313 (52%), Gaps = 30/313 (10%)

Query  10   VTGASGYIASWLVKFLLHSGYNVKASVRDPNDPKKTQHLLSLGGAKERLHLFKANLLEEG  69
            VTG + +IAS +VK LL  GY V+ +VRD +  +K + L  L GAKERL +F+A+L  +G
Sbjct  6    VTGGTSFIASHVVKALLDFGYFVRTTVRDSSYEEKVRFLWELKGAKERLKIFEADLTVDG  65

Query  70   SFDAVVDGCEGVFHTASPFYYSVTDPQAE-LLDPAVKGTLNLLGSCAKAP-SVKRVVLTS  127
            SFD  V G +GVFH AS     + D   E L D  + GT NL+ SC K+  +VKR+VLTS
Sbjct  66   SFDDAVKGVDGVFHIASRISVCLDDNDLEKLADRDIYGTRNLMNSCEKSRNTVKRIVLTS  125

Query  128  SIAAVAYSGQPRTPEVVVDESWWTSPDYCKEKQLWYVLSKTLAEDAAWKFVKEKGIDMVV  187
            S  +V YS    T    +  S W+  +Y +   +WY  +KTL E  AW    EK +++VV
Sbjct  126  SSTSVRYSYDA-TKASPLKGSHWSDLEYFRSFSIWYGYAKTLVEKEAWLIAAEKNLNLVV  184

Query  188  VNPAMVIGPLLQPTLNTSSAAVLSLVNGAETYPNSSFGWVNVKDVANAHILAFENPSANG  247
            V P+  IGP+L P                   P +        DVA A ILA + P A+G
Sbjct  185  VIPSFCIGPILSPK------------------PTN--------DVAAAQILAMKEPKASG  218

Query  248  RYLMVERVAHYSDILKILRDLYPTMQLPEKCADDNPLMQNYQVSKEKAKSLGI-EFTTLE  306
            R +    VAH+S+I+++L+  YP      KC  +      + +   K   LG   F +L
Sbjct  219  RIICSSSVAHWSEIIEMLKPKYPLYPFETKCGSEEGRDMPHSLDTRKIHELGFGSFKSLA  278

Query  307  ESIKETVESLKEK  319
            E   + ++  ++K
Sbjct  279  EMFDDCIKCFQDK  291


>XP_009144935.1 PREDICTED: LOW QUALITY PROTEIN: bifunctional dihydroflavonol
4-reductase/flavanone 4-reductase [Brassica rapa]
Length=337

 Score = 169 bits (429),  Expect = 5e-50, Method: Compositional matrix adjust.
 Identities = 108/337 (32%), Positives = 176/337 (52%), Gaps = 32/337 (9%)

Query  7    TVCVTGASGYIASWLVKFLLHSGYNVKASVRDPNDPKKTQHLLSLGGAKERLHLFKANLL  66
            T C TGA+GY+ SWLVK       ++   +RD     K+Q+  +     ERL LF+ +L
Sbjct  2    TYCATGANGYVGSWLVK-----SKSINGMLRDL---AKSQYFQTKWRGNERLRLFRVDLQ  53

Query  67   EEGSFDAVVDGCEGVFHTASPFYYSVTDP--------QAELLDPAVKGTLNLLGSCAKAP  118
            ++GSFD  + GC+GVFH A+   + ++          Q +++DPA+KG  N+LGSC K+
Sbjct  54   DDGSFDDAIKGCDGVFHIAASMEFDISPNHVNLESYVQGKVIDPAIKGVRNVLGSCLKSK  113

Query  119  SVKRVVLTSSIAAVAYSGQPRTPEVVVDESWWTSPDYCKEKQ-------LWYVLSKTLAE  171
            SVKRVV TSSI+ +    +      +V E+     D   + +       L YVLSK ++E
Sbjct  114  SVKRVVFTSSISTLTSKDENERWRSIVGETCKIPIDRVLKTKASGWINTLIYVLSKLISE  173

Query  172  DAAWKFVKEKGIDMVVVNPAMVIGPLLQPTLNTSSAAVLSLVNGAETYPNSSFGWVNVKD  231
            + ++++ KE+G+D+V V P  V GP L P+L +S    LS +  A      S G V+V+D
Sbjct  174  EESFRYEKERGLDLVSVIPTTVSGPFLTPSLPSSLQVXLSPIT-AVNKRMGSIGLVHVQD  232

Query  232  VANAHILAFENPSANGRYLMVERVAHYSDIL------KILRDLYPTMQLPEKCADDNPLM  285
```

```
                 +  AH+   E P A G+Y+         D+L       K    L+   ++ +   ++ P +
Sbjct  233  ICIAHLFLMEEPKAEGQYICCVDNIDMHDLLLNHTFTKNFTRLFSVYRVDDD-LEERPGL  291

Query  286  QNYQVSKEKAKSLGIEFT-TLEESIKETVESLKEKKF  321
                +S +K + LG ++   +EE I  T+++    +F
Sbjct  292  MKPMISSKKLRKLGFQYKYGIEEIIXSTIDASINIRF  328


>XP_009142332.1 PREDICTED: vestitone reductase-like [Brassica rapa]
Length=358

 Score = 162 bits (411),  Expect = 4e-47, Method: Compositional matrix adjust.
 Identities = 104/306 (34%), Positives = 158/306 (52%), Gaps = 11/306 (4%)

Query  8    VCVTGASGYIASWLVKFLLHSGYNVKASVR-DPNDPKK-TQHLLSLGGAKERLHLFKANL  65
            VCVTG +G+IASWL+  LL  GY+V+A+VR +P   KK   +L  L  A ERL +F A L
Sbjct  32   VCVTGGTGFIASWLIMRLLQRGYSVRATVRTNPEGSKKDISYLTELPFASERLKIFTAEL  91

Query  66   LEEGSFDAVVDGCEGVFHTASPFYYSVTDPQAELLDPAVKGTLNLLGSCAKAPSVKRVVL  125
             E  SF   ++GC+ VFH A P   +  + +  +    V+G + +L SC  A +VKR
Sbjct  92   NEPESFKPAIEGCKAVFHVAHPMDPTSNETEETVTKRTVQGLMGILKSCLDAKTVKRFFY  151

Query  126  TSSIAAVAYSGQPRTPEVVV--DESWWTSPDYC---KEKQLW--YVLSKTLAEDAAWKFV  178
            TSS   V Y              DES W+  +     KEK++   YV+SK  AE  A +F
Sbjct  152  TSSAVTVFYGVGSGVGGNGGVVDESVWSDVEVFRNQKEKRVSSSYVVSKMAAEMTALEFG  211

Query  179  KEKGIDMVVVNPAMVIGPLLQPTLNTSSAAVLSLVNG--AETYPNSSFGWVNVKDVANAH  236
             + G+++V +   +V+GP +  +L +S    L+++ G   E Y   ++  V++ DVA A
Sbjct  212  GKNGLEVVTLVIPLVVGPFISQSLPSSVFISLAMIFGNYKEKYLFDTYNMVHIDDVARAM  271

Query  237  ILAFENPSANGRYLMVERVAHYSDILKILRDLYPTMQLPEKCADDNPLMQNYQVSKEKAK  296
            I   E P A GRY+      +  ++ + L   +P  QLP        + +   +S +K +
Sbjct  272  IFLLEKPIAKGRYICSSVEMNVDEVFEFLSTRFPQFQLPSVDLKSYRVEKRMSLSSKKLR  331

Query  297  SLGIEF  302
            S G EF
Sbjct  332  SAGFEF  337


>XP_009137924.1 PREDICTED: cinnamoyl-CoA reductase 2 [Brassica rapa]
Length=303

 Score = 153 bits (386),  Expect = 5e-44, Method: Compositional matrix adjust.
 Identities = 87/298 (29%), Positives = 161/298 (54%), Gaps = 23/298 (8%)

Query  7    TVCVTGASGYIASWLVKFLLHSGYNVKASVRDPNDPKKTQHLLSLGGAKERLHLFKANLL  66
              CV  AS Y+  W++K LL  GY+V A++R   D +  + +  +   +ERL ++  ++L
Sbjct  9    CCCVLDASTYVGFWILKKLLSRGYSVHAAIRKNGDSEIEETIREMEATEERLVVYDVDVL  68

Query  67   EEGSFDAVVDGCEGVFHTA-SPFYYSVTDPQAELLDPAVKGTLNLLGSCAKAPSVKRVVL  125
            +  S    +  C  VF    SP  Y   +     +D  V+G +N++ +C +  S++++V
Sbjct  69   DYQSILVSLKTCNAVFCCLDSPEGYDEKE-----VDLEVRGAINVVEACGRTESIEKIVF  123

Query  126  TSSIAAVAYSGQPRTPEVVVDESWWTSPDYCKEKQLWYVLSKTLAEDAAWKFVKEKGIDM  185
            +SS+ A  +     T + V DE  W+  D+C+ K+LW+ L+K L+E AAW    ++ ++M
Sbjct  124  SSSLTASIWRDNIGTQKDV-DEKCWSDQDFCRNKKLWHALAKMLSEKAAWALAMDRRLNM  182

Query  186  VVVNPAMVIGPLL-----QPTLNTSSAAVLSLVNGAETYPNSSFGWVNVKDVANAHILAF  240
            V +NP +++GP +     +PT++         L   A+ Y N    +V+VK +A+ HI A+
Sbjct  183  VSINPGLIVGPSVAQYNPRPTMSY-------LKGAAQMYENGVLAYVDVKFLADVHIRAY  235

Query  241  ENPSANGRYLMVERVAHY-SDILKILRDLYPTMQLPEKCADDNPLMQNYQVSKEKAKS  297
            E+ SA GRY    ++ +   + LK++  L P + +P +      +MQ  +V +E+ ++
Sbjct  236  EDVSACGRYFCFNQIVNTEEEALKLVESLSPLIPMPPR---YESVMQGSEVYEERLRN  290


>XP_009140504.1 PREDICTED: cinnamoyl-CoA reductase 2 [Brassica rapa]
Length=341
```

```
 Score = 150 bits (380),  Expect = 9e-43, Method: Compositional matrix adjust.
 Identities = 87/293 (30%), Positives = 163/293 (56%), Gaps = 17/293 (6%)

Query  9    CVTGASGYIASWLVKFLLHSGYNVKASVRDPNDPKKTQHLLSLGGAKERLHLFKANLLEE  68
            CV  AS Y+  W++K LL  GY+V+A++R   +    + + ++   +ERL ++  ++L+
Sbjct  49   CVLDASTYVGFWILKKLLTRGYSVRAAIRKNGESILEEKIRNMQATEERLVVYDVDVLDY  108

Query  69   GSFDAVVDGCEGVFHTASPFYYSVTDPQA--EL-LDPAVKGTLNLLGSCAKAPSVKRVVL  125
             S    ++ C  VF         + +P+   EL +D  V+G +N++ +CA+  S+ ++V
Sbjct  109  QSILVSLNNCNAVF-------CCLDNPEGYDELEVDLEVRGAINVVEACARTESIDKIVF  161

Query  126  TSSIAAVAYSGQPRTPEVVVDESWWTSPDYCKEKQLWYVLSKTLAEDAAWKFVKEKGIDM  185
            +SS+ A  ++    T + V DE  W+  D+C +K+LW+ L+KT +E AAW    ++ ++M
Sbjct  162  SSSLTAAIWTDNIGTQKDV-DEKSWSDLDFCLKKKLWHALAKTQSEKAAWALAMDRMVNM  220

Query  186  VVVNPAMVIGPLLQPTLNTSSAAVLSLVNGAETYPNSSFGWVNVKDVANAHILAFENPSA  245
            V VNP +++GP +  T +     +  L   A+ Y N    +V+V+ VA+ HI  FE+ SA
Sbjct  221  VSVNPGLIVGPSV--TQHNPRPTMSFLKGAAQMYENGVLAYVDVEFVADVHIRVFEDTSA  278

Query  246  NGRYLMVERVAHY-SDILKILRDLYPTMQLPEKCADDNPLMQNYQVSKEKAKS  297
             GRY    ++ +   + LK++  L P + +P +   +   MQ  +V +E+ ++
Sbjct  279  CGRYFCFNQIVNTEEEALKLVEILSPLIPMPPRYEKE---MQGSEVYEERLRN  328


>XP_009143137.1 PREDICTED: vestitone reductase [Brassica rapa]
Length=352

 Score = 149 bits (375),  Expect = 7e-42, Method: Compositional matrix adjust.
 Identities = 104/306 (34%), Positives = 158/306 (52%), Gaps = 11/306 (4%)

Query  8    VCVTGASGYIASWLVKFLLHSGYNVKASVR-DPNDPKK-TQHLLSLGGAKERLHLFKANL  65
            VCVTG +G++ASWL+  LL  GY+V A+VR +P   KK   +L  L  A ERL +F A+L
Sbjct  26   VCVTGGTGFVASWLIMRLLQRGYSVHATVRTNPEGNKKDISYLTELPFASERLKIFTADL  85

Query  66   LEEGSFDAVVDGCEGVFHTASPFYYSVTDPQAELLDPAVKGTLNLLGSCAKAPSVKRVVL  125
             E  SF   ++GC+ VFH A P   +  + +  +    V+G + +L S   + +VKR
Sbjct  86   NEPESFKPAIEGCKAVFHVAHPMDPNSNETEEIVTKRTVQGLMGILKSSVDSKTVKRFFY  145

Query  126  TSSIAAVAYSGQPRTPEVV--VDESWWTSPDYCK---EKQLW--YVLSKTLAEDAAWKFV  178
            TSS   V Y            VDES W+  +  +   EK++   YV+SK  AE AA +F
Sbjct  146  TSSAVTVFYGVGSGAGGNGGEVDESVWSDVEVFRNQEEKRVSSSYVVSKMAAETAALEFG  205

Query  179  KEKGIDMVVVNPAMVIGPLLQPTLNTSSAAVLSLVNG--AETYPNSSFGWVNVKDVANAH  236
               G+++V V   +V+GP +  +L +S    L+++ G   E Y   ++  V++ DVA A
Sbjct  206  GNNGLEVVTVVLPLVVGPFICQSLPSSVFISLAMIFGNYKEKYLFDTYNMVHIDDVARAM  265

Query  237  ILAFENPSANGRYLMVERVAHYSDILKILRDLYPTMQLPEKCADDNPLMQNYQVSKEKAK  296
            I   E P A GRY+          +I ++L   +P  QLP         + +   +S +K +
Sbjct  266  IFLLERPVAKGRYICSSVEMKIDEIFELLSTKFPQFQLPSVDLKSYRVEKRMSLSSKKLR  325

Query  297  SLGIEF  302
            S G EF
Sbjct  326  SAGFEF  331


>XP_009125995.1 PREDICTED: cinnamoyl-CoA reductase 2-like isoform X1 [Brassica
rapa]
Length=367

 Score = 123 bits (309),  Expect = 4e-32, Method: Compositional matrix adjust.
 Identities = 85/304 (28%), Positives = 139/304 (46%), Gaps = 31/304 (10%)

Query  6    KTVCVTGASGYIASWLVKFLLHSGYNVKASVRDPNDPKKTQHL------LSLGGAKERLH  59
            + VCVTG   Y+   +VK LL  GY+V+  V  P D +K   +       S G    R+
Sbjct  52   RLVCVTGGVSYLGRAIVKRLLVHGYSVRIVVDCPEDKEKVSEMEADAETASFGN---RIT  108

Query  60   LFKANLLEEGSFDAVVDGCEGVFHTASPFYYSVTDPQA------ELLDPAVKGTLNLLGS  113
               + L E  S     DGC+GVFHTA+       DP         + +   K + N++ +
Sbjct  109  SVVSRLTETESLIKAFDGCDGVFHTAA-----FVDPAGISGYSKSMAELEAKVSENVIEA  163
```

```
Query  114  CAKAPSVKRVVLTSSIAAVAYSGQPRTP--EVVVDESWWTSPDYCKEKQLWYVLSKTLAE  171
            C +  SV++ V TSS+ A A            +++E  W+    C   +LWY L K  AE
Sbjct  164  CTRTGSVRKCVFTSSLLACALKDNSLNDLDHSIINEESWSDEQLCVHNKLWYALGKLKAE  223

Query  172  DAAWKFVKEKGIDMVVVNPAMVIGPLLQPTLNTSSAAVLSLVNGA-ETYPNSSFGWVNVK  230
             AAW+    KG+ +  + PA++ GP        +S + L+ + GA + Y N     ++V
Sbjct  224  KAAWRIADSKGLKLATICPALITGP---DFFQLNSTSTLAYLKGAKDMYRNRLLATMDVN  280

Query  231  DVANAHILAFE---NPSANGRYLMVERVAHYSDILKILRDLYPTMQLPEKCADDNPLMQN  287
             +A  H+  +E   N +A GRY+  + +       K+ +D+    Q+ + C   +   +N
Sbjct  281  RLAKVHVRLWEGLGNKTAFGRYICFDTILSKDGAEKLAKDIGA--QIEKICGSSDDSEEN  338

Query  288  YQVS  291
             + S
Sbjct  339  AETS  342


>XP_009121639.1 PREDICTED: cinnamoyl-CoA reductase 2 [Brassica rapa]
Length=368

 Score = 120 bits (300),  Expect = 7e-31, Method: Compositional matrix adjust.
 Identities = 84/306 (27%), Positives = 138/306 (45%), Gaps = 25/306 (8%)

Query  6    KTVCVTGASGYIASWLVKFLLHSGYNVKASVRDPNDPKKTQHL---LSLGGAKERLHLFK  62
            + VCVTG   Y+   +VK LL  GY V+  +    D +K   +           R+
Sbjct  52   RLVCVTGGVSYLGRAIVKRLLVHGYTVRIVIDCEEDKEKVSEMEADAETASFSNRITSVV  111

Query  63   ANLLEEGSFDAVVDGCEGVFHTASPFYYSVTDPQA------ELLDPAVKGTLNLLGSCAK  116
            + L E  S     DGC GVFHTA+       DP         + +   K + N+  +C +
Sbjct  112  SRLTEIESLVKAFDGCAGVFHTAA-----FVDPAGISGYSKSMAELEAKVSENVTEACTR  166

Query  117  APSVKRVVLTSSIAAVAYSGQ--PRTPEVVVDESWWTSPDYCKEKQLWYVLSKTLAEDAA  174
              SV++ V TSS+ A A            V++E  W+   +C   +LWY L K  AE AA
Sbjct  167  TGSVRKCVFTSSLLACALQENFYNDLDHSVINEESWSDEQFCINNKLWYALGKLKAEKAA  226

Query  175  WKFVKEKGIDMVVVNPAMVIGPLLQPTLNTSSAAVLSLVNGA-ETYPNSSFGWVNVKDVA  233
            W+    KG+ +  + PA++ GP      + +S + L+ + GA E Y N     ++V  +A
Sbjct  227  WRIADSKGLKLATICPALITGP---DFFHRNSTSTLAYLKGAKEMYSNGLLATMDVNRLA  283

Query  234  NAHILAFE---NPSANGRYLMVERVAHYSDILKILRDLYPTMQLPEKCADDNPLMQNYQV  290
             AH+  +E   + +A GRY+  + +       K+ +D+   +Q+ + C  ++    N +
Sbjct  284  KAHVRVWEGLGDKTAFGRYICFDTILSKDGAQKLAKDI--GVQVEKICGSNSDSNANAET  341

Query  291  SKEKAK  296
            S    K
Sbjct  342  STRNLK  347


>XP_009137735.1 PREDICTED: tetraketide alpha-pyrone reductase 2-like [Brassica
rapa]
 XP_009140144.1 PREDICTED: tetraketide alpha-pyrone reductase 2-like [Brassica
rapa]
 XP_009140389.1 PREDICTED: tetraketide alpha-pyrone reductase 2-like [Brassica
rapa]
Length=203

 Score = 110 bits (274),  Expect = 1e-28, Method: Compositional matrix adjust.
 Identities = 65/143 (45%), Positives = 88/143 (62%), Gaps = 3/143 (2%)

Query  17   IASWLVKFLLHSGYNVKASVRDPNDPKKTQHLLSLGGAKERLHLFKANLLEEGSFDAVVD  76
            IAS +VK LL  G++V+ +VRD +D +K + L  L GAKERL +F+A+L  EGSFD  +
Sbjct  43   IASHVVKALLDLGHSVRTTVRDSSDEEKVRFLWELKGAKERLKIFEADLTVEGSFDEAIK  102

Query  77   GCEGVFHTASPFYYSVTDPQAE-LLDPAVKGTLNLLGSCAKAP-SVKRVVLTSSIAAVAY  134
            G +GVFH AS     V     E L+D  + GT NL+ SC K+  +VKR+VLTSS  AV Y
Sbjct  103  GVDGVFHIASRVTLCVDSNDLEKLVDRDINGTRNLMNSCEKSRNTVKRIVLTSSSTAVRY  162

Query  135  SGQPRTPEVVVDESWWTSPDYCK  157
```

```
                 T    ++ES ++  DY K
Sbjct  163  RYDA-TEASPLNESHYSDLDYSK  184


>XP_009125996.1 PREDICTED: cinnamoyl-CoA reductase 2-like isoform X2 [Brassica
rapa]
Length=350

 Score = 112 bits (280),  Expect = 3e-28, Method: Compositional matrix adjust.
 Identities = 80/303 (26%), Positives = 130/303 (43%), Gaps = 46/303 (15%)

Query  6    KTVCVTGASGYIASWLVKFLLHSGYNVKASVRDPNDPKKTQHL------LSLGGAKERLH  59
            + VCVTG   Y+   +VK LL  GY+V+  V  P D +K   +       S G    R+
Sbjct  52   RLVCVTGGVSYLGRAIVKRLLVHGYSVRIVVDCPEDKEKVSEMEADAETASFGN---RIT  108

Query  60   LFKANLLEEGSFDAVVDGCEGVFHTASPFYYSVTDPQA------ELLDPAVKGTLNLLGS  113
               + L E  S     DGC+GVFHTA+       DP         + +   K + N++ +
Sbjct  109  SVVSRLTETESLIKAFDGCDGVFHTAA-----FVDPAGISGYSKSMAELEAKVSENVIEA  163

Query  114  CAKAPSVKRVVLTSSIAAVAYSGQPRTP--EVVVDESWWTSPDYCKEKQLWYVLSKTLAE  171
            C +  SV++ V TSS+ A A            +++E  W+    C   +LWY L K  AE
Sbjct  164  CTRTGSVRKCVFTSSLLACALKDNSLNDLDHSIINEESWSDEQLCVHNKLWYALGKLKAE  223

Query  172  DAAWKFVKEKGIDMVVVNPAMVIGPLLQPTLNTSSAAVLSLVNGAETYPNSSFGWVNVKD  231
             AAW+    KG+ +  + PA++ G                     + Y N     ++V
Sbjct  224  KAAWRIADSKGLKLATICPALITGA-------------------KDMYRNRLLATMDVNR  264

Query  232  VANAHILAFE---NPSANGRYLMVERVAHYSDILKILRDLYPTMQLPEKCADDNPLMQNY  288
            +A  H+  +E   N +A GRY+  + +       K+ +D+    Q+ + C   +   +N
Sbjct  265  LAKVHVRLWEGLGNKTAFGRYICFDTILSKDGAEKLAKDIGA--QIEKICGSSDDSEENA  322

Query  289  QVS  291
            + S
Sbjct  323  ETS  325


>XP_009141386.1 PREDICTED: uncharacterized protein At2g34460, chloroplastic
isoform
X2 [Brassica rapa]
Length=289

 Score = 45.8 bits (107),  Expect = 2e-05, Method: Compositional matrix adjust.
 Identities = 57/193 (30%), Positives = 81/193 (42%), Gaps = 36/193 (19%)

Query  6    KTVCVTGASGYIASWLVKFLLHSGYNVKASVRDPNDPKKTQHLLSLGGAKERLHLFKANL  65
            K V V GA+G     +V+ LL  G+ VKA VRD +  K +            LH  KA++
Sbjct  55   KKVFVAGATGQTGKRIVEQLLSRGFAVKAGVRDVDKAKTSFR------QDPSLHFVKADV  108

Query  66   LE--EGSFDAVVDGCEGVFHTASPFYYSVTDPQAELLDP---AVKGTLNLLGSCAKAPSV  120
             E  E   +A+ D    V   A+ F      P  +L  P      GT++L+ +C K   V
Sbjct  109  TEGSEKLAEAIGDDSHAVI-CATGFR-----PGFDLFAPWKIDNFGTVSLVDACKKQ-GV  161

Query  121  KRVVLTSSI-AAVAYSGQPRTPEVVVDESWWTSPDYCKEKQLWYVLSKTL-AEDAAWKFV  178
             R VL SSI    A +GQ   P  +                   V   TL A+  A K++
Sbjct  162  NRFVLVSSILVNGAATGQILNPAYI----------------FLNVFGLTLVAKLQAEKYI  205

Query  179  KEKGIDMVVVNPA  191
            K  G++  +V P
Sbjct  206  KRSGVNYTIVRPG  218


>XP_009141385.1 PREDICTED: uncharacterized protein At2g34460, chloroplastic
isoform
X1 [Brassica rapa]
Length=289

 Score = 45.8 bits (107),  Expect = 2e-05, Method: Compositional matrix adjust.
 Identities = 57/193 (30%), Positives = 81/193 (42%), Gaps = 36/193 (19%)
```

```
Query  6    KTVCVTGASGYIASWLVKFLLHSGYNVKASVRDPNDPKKTQHLLSLGGAKERLHLFKANL  65
            K V V GA+G     +V+ LL  G+ VKA VRD +  K +            LH  KA++
Sbjct  55   KKVFVAGATGQTGKRIVEQLLSRGFAVKAGVRDVDKAKTSFR------QDPSLHFVKADV  108

Query  66   LE--EGSFDAVVDGCEGVFHTASPFYYSVTDPQAELLDP---AVKGTLNLLGSCAKAPSV  120
             E  E   +A+ D    V   A+ F      P  +L  P      GT++L+ +C K   V
Sbjct  109  TEGSEKLAEAIGDDSHAVI-CATGFR-----PGFDLFAPWKIDNFGTVSLVDACKKQ-GV  161

Query  121  KRVVLTSSI-AAVAYSGQPRTPEVVVDESWWTSPDYCKEKQLWYVLSKTL-AEDAAWKFV  178
             R VL SSI    A +GQ   P  +                   V   TL A+  A K++
Sbjct  162  NRFVLVSSILVNGAATGQILNPAYI----------------FLNVFGLTLVAKLQAEKYI  205

Query  179  KEKGIDMVVVNPA  191
            K  G++  +V P
Sbjct  206  KRSGVNYTIVRPG  218


>XP_009108619.1 PREDICTED: uncharacterized protein ycf39 [Brassica rapa]
Length=395

 Score = 36.6 bits (83),  Expect = 0.023, Method: Compositional matrix adjust.
 Identities = 51/248 (21%), Positives = 96/248 (39%), Gaps = 46/248 (19%)

Query  3    VTAKTVCVTGASGYIASWLVKFLLHSGYNVKASVRDPNDPKKTQHLLSLGGAKERLHLFK  62
            V   ++ V GA+G +   +V+  L  GY+V+  VR    P     L   G       +
Sbjct  77   VRPTSILVVGATGTLGRQIVRRALDEGYDVRCLVRP--RPAPADFLRDWGAT-----VVN  129

Query  63   ANLLEEGSFDAVVDGCEGVFHTASPFYYSVTDPQAELLDPAVKGTLNLLGSCAKAPSVKR  122
            A+L +  +  A + G   V   A+        P+  +     +G + L+  CAKA  +++
Sbjct  130  ADLSKPETIPATLVGVHTVIDCAT------GRPEEPIKTVDWEGKVALI-QCAKAMGIQK  182

Query  123  VVLTSSIAAVAYSGQPRTPEVVVDESWWTSPDYCKEKQLWYVLSKTLAEDAAWKFVKEKG  182
             V  S           + PEV + E       YC E                 KF++E G
Sbjct  183  YVFYS------IHNCDKHPEVPLMEI-----KYCTE-----------------KFLQESG  214

Query  183  IDMVVVNPAMVIGPLLQPTLNTSSAAVLSLVNGAETYPNSSFGWVNVKDVANAHILAFEN  242
            ++ + +     +   +Q  +   +  +L   +   T   +   +++ +D+A   ++A  N
Sbjct  215  LNHITIR----LCGFMQGLIGQYAVPILEEKSVWGTDAPTRVAYMDTQDIARLTLIALRN  270

Query  243  PSANGRYL  250
               NG+ L
Sbjct  271  EKVNGKLL  278


>XP_009137405.1 PREDICTED: UDP-glucose 4-epimerase 2 [Brassica rapa]
Length=348

 Score = 35.4 bits (80),  Expect = 0.066, Method: Compositional matrix adjust.
 Identities = 67/265 (25%), Positives = 106/265 (40%), Gaps = 42/265 (16%)

Query  5    AKTVCVTGASGYIASWLVKFLLHSGYN-VKASVRDPNDPKKTQHLLSLGGAK-ERLHLFK  62
            AK + VTG SGYI S  V  LL+ GY+ V     D +     + +  L G   +RL   +
Sbjct  2    AKNILVTGGSGYIGSHTVLQLLNGGYSAVVVDNLDNSSAVSLERVKKLAGQNGDRLSFHQ  61

Query  63   ANL---------LEEGSFDAVVD--GCEGVFHTAS-PFYYSVTDPQAELLDPAVKGTLNL  110
             +L           E  FDAV+   G + V  +   P  Y          +  + GT+ L
Sbjct  62   VDLRDRPALEKIFSETKFDAVIHFAGLKAVGESVEKPLLY---------YNNNIVGTITL  112

Query  111  LGSCAKAPSVKRVVLTSSIAAVAYSGQ-PRTPEVVVDESWWTSPDYCKEKQLWYVLSKTL  169
            L   A+    K +V +SS     +  + P T E  +     T+P Y + K     + + +
Sbjct  113  LEVMAQY-GCKNLVFSSSATVYGWPKEVPCTEESPISA---TNP-YGRTKLFIEEICRDV  167

Query  170  AE-DAAWKFVKEKGIDMVVVNPAMVIG--PL-----LQPTLNTSSAAVLS--LVNGAETY  219
               D  WK +  +  + V  +P+  IG  PL     L P +   +        V G +
Sbjct  168  HRSDPEWKIILLRYFNPVGAHPSGYIGEDPLGIPNNLMPYIQQVAVGRRPHLTVFGTDYK  227

Query  220  PNSSFG---WVNVKDVANAHILAFE  241
                 G   +++V D+A+ HI A
Sbjct  228  TKDGTGVRDYIHVIDLADGHIAALR  252
```

```
>XP_009137528.1 PREDICTED: UDP-glucose 4-epimerase 2 [Brassica rapa]
Length=348

 Score = 30.4 bits (67),  Expect = 2.0, Method: Compositional matrix adjust.
 Identities = 28/83 (34%), Positives = 37/83 (45%), Gaps = 15/83 (18%)

Query  6   KTVCVTGASGYIASWLVKFLLHSGYNVKASVRDPNDPKKT---QHLLSLGGAK-ERLHLF  61
           K V VTG +GYI +  V  LL+ GY+V   V D +D       Q +  L G    RL
Sbjct  3   KNVLVTGGAGYIGTHTVLQLLNGGYSV--VVVDNHDNSSVIALQRVKKLAGDNGNRLSFH  60

Query  62  KANL---------LEEGSFDAVV  75
           + +L           E  FDAV+
Sbjct  61  QVDLRDRPALEKIFSETKFDAVI  83


>XP_009150492.1 PREDICTED: protein TIC 62, chloroplastic [Brassica rapa]
Length=511

 Score = 29.3 bits (64),  Expect = 6.4, Method: Compositional matrix adjust.
 Identities = 13/32 (41%), Positives = 19/32 (59%), Gaps = 0/32 (0%)

Query  7    TVCVTGASGYIASWLVKFLLHSGYNVKASVRD  38
            TV V GA+G     + + LL  G++V+A V D
Sbjct  95   TVFVAGATGQAGIRIAQTLLQRGFSVRAGVPD  126


  Database: NCBI Protein Reference Sequences
    Posted date:  Aug 27, 2016  10:35 PM
  Number of letters in database: 19,207,319
  Number of sequences in database:  45,977

Lambda      K        H
   0.315    0.130    0.383
Gapped
Lambda      K        H
   0.267   0.0410    0.140
Matrix: BLOSUM62
Gap Penalties: Existence: 11, Extension: 1
Number of Sequences: 45977
Number of Hits to DB: 236
Number of extensions: 0
Number of successful extensions: 0
Number of sequences better than 100: 0
Number of HSP's better than 100 without gapping: 0
Number of HSP's gapped: 0
Number of HSP's successfully gapped: 0
Length of query: 328
Length of database: 19207319
Length adjustment: 103
Effective length of query: 225
Effective length of database: 14471688
Effective search space: 3256129800
Effective search space used: 3256129800
T: 21
A: 40
X1: 16 (7.3 bits)
X2: 38 (14.6 bits)
X3: 64 (24.7 bits)
S1: 40 (20.0 bits)
S2: 53 (25.0 bits)
ka-blk-alpha gapped: 1.9
ka-blk-alpha ungapped: 0.7916
ka-blk-alpha_v gapped: 42.6028
ka-blk-alpha_v ungapped: 4.96466
ka-blk-sigma gapped: 43.6362
```
